# Alternative Access to Functionalized 2,8-Ethanonoradamantane Derivatives

**DOI:** 10.3390/molecules22060906

**Published:** 2017-05-31

**Authors:** Pelayo Camps, Tània Gómez, Ane Otermin, Mercè Font-Bardia

**Affiliations:** 1 Laboratori de Química Farmacèutica (Unitat Associada al CSIC), Facultat de Farmàcia i Ciències de la Alimentació and Institut de Biomedicina (IBUB), Universitat de Barcelona, Av. Joan XXIII 27-31, Barcelona 08028, Spain; tania_gomeznadal@hotmail.com (T.G.); aneotermin@gmail.com (A.O.); 2 Departament de Mineralogia, Petrologia i Geologia Aplicada, Universitat de Barcelona, Martí Franquès s/n, Barcelona 08028, Spain; mercef@ccit.ub.edu; 3 Unitat de Difracció de RX, Centres Científics i Tecnològics de la Universitat de Barcelona (CCiTUB), Solé i Sabarís 1-3, Barcelona 08028, Spain

**Keywords:** polycycles, intramolecular cycloaddition, retrocycloaddition, domino reactions, X-ray diffraction

## Abstract

7a-(Methoxycarbonyl)-*N*-methyl-1,3a,5,6,7,7a-hexahydro-4*H*-1,4,6-(epiethane[1,1,2]triyl)indene-4,9-dicarboximide has been prepared through a modification of a previous synthetic sequence, in which the benzyloxymethyl hydroxyl protecting group has been replaced by methoxymethyl, to avoid the apparent formation of a benzyl ester derivative as a side product. The overall yield of the new synthetic sequence is comparable to the previous one. Two advantages of the new procedure are: (a) no benzyl ester was formed and (b) a stereoisomeric mixture of *syn*- and *anti*-alcohols at the beginning of the synthetic sequence could be separated and the rest of the synthesis could be carried out with the main *syn*-stereoisomer instead of the corresponding stereoisomeric mixture as it was the case in the previous process. Additionally, several functional 2,8-ethanonoradamantane derivatives have been prepared.

## 1. Introduction

**Scheme 1:**
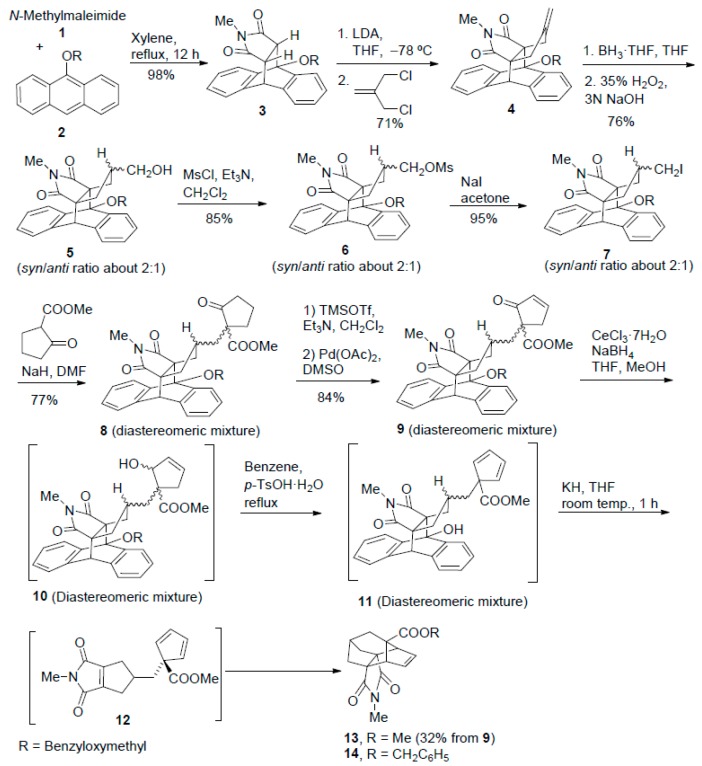
Previously improved synthesis of compound **13**.

**Scheme 2:**
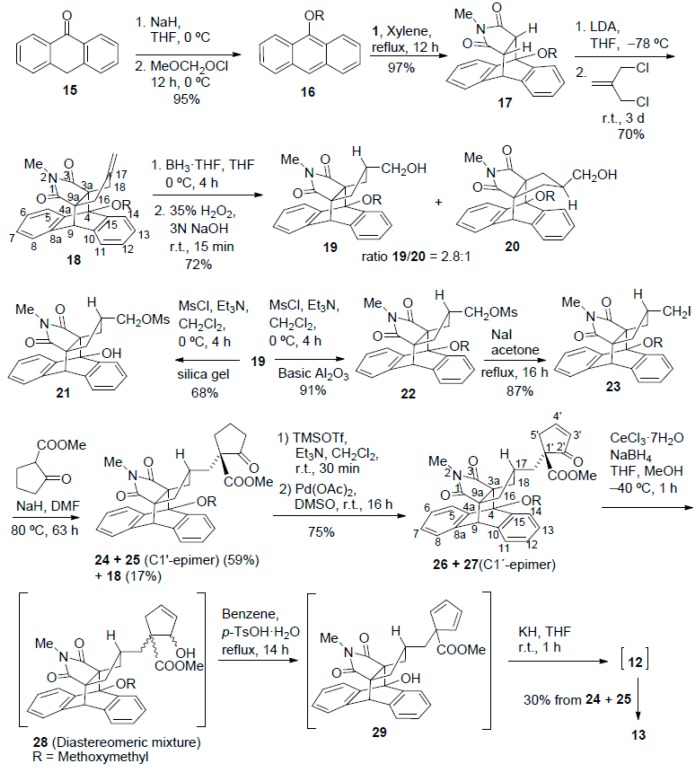
Alternative synthesis of tetracycle **13**, using methoxymethyl as a hydroxyl-protecting group.

Some time ago, the synthesis of the functionalized polycycle **13** as a new scaffold for the preparation of compounds with potential biological activity was described [1]. Later on, improvements of the synthesis of **13** were performed [2,3], the highly-optimized synthetic route to this compound is shown in Scheme 1. During purification of this compound, implying a sublimation process, the presence of a benzyl ester derivative, probably **14**, in the non-sublimed residue was suggested by ^1^H-NMR. To solve this problem, an alternative synthesis of polycycle **13** was planned in which the methoxymethyl hydroxyl-protecting group would be used instead of the benzyloxymethyl one (Scheme 2).

## 2. Results and Discussion

The reaction of anthrone with methoxymethyl chloride, using NaH as the base, and following a procedure similar to that used to prepare anthracene **2** [4,5], gave 9-methoxymethylanthracene **16**. The reaction of *N*-methylmaleimide **1** with anthracene **16** gave the corresponding Diels-Alder adduct **17**. The reaction of **17** with 3-chloro-2-chloromethyl-1-propene using lithium diisopropylamide as the base gave the methylenecyclopentane derivative **18** in 70% yield. Hydroboration of **18** with the borane-THF complex in THF followed by hydrogen peroxide oxidation of the intermediate boranes under strongly basic conditions gave a stereoisomeric mixture of alcohols **19** and **20** in a ratio **19**/**20** = 2.8:1 (^1^H-NMR). Worthy of note, this mixture was separated by silica gel column chromatography and each stereoisomer could be fully characterized. In the previous synthetic sequence using the benzyloxymethyl hydroxyl protecting group, the stereoisomeric mixture of alcohols **5** could not be separated and, consequently, the next steps in that synthetic sequence were carried out with the corresponding stereoisomeric mixtures. In the present work, however, the synthetic sequence of Scheme 2 was carried out with the main stereoisomeric racemate *syn*-alcohol **19**.

**Scheme 3:**
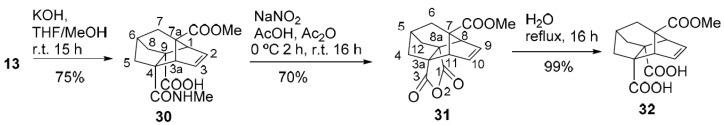
Several functional group transformations from imide ester **13**.

As previously observed for the main *syn*-stereoisomer of **5** [2], the ^1^H-NMR data of the *syn*-alcohol **19** suggests that exists mainly in the shown envelope conformation, the 16-H_n_ (δ = 0.92 ppm) and 18-H_n_ (δ = 1.14 ppm) protons appearing quite shielded by the aromatic ring as triplets (^2^*J*_(HH)_ = ^3^*J*_(HH)_ = 12.8 Hz) due to the similar value of the geminal and vicinal (dihedral angle close to 180°) coupling constants. The 16-H_x_ (δ = 2.16 ppm) and 18-H_x_ (δ = 2.28 ppm) protons of alcohol **19** are not so affected by the aromatic ring. In the case of the minor *anti*-stereoisomer **20**, 17-H is the more shielded proton (δ = 1.09–1.20 ppm). In this case, the 16-H_n_ (δ = 1.71 ppm) and 18-H_n_ (δ = 2.12 ppm) protons are not significantly affected by the aromatic ring. However, the vicinal coupling constants of 16-H_x_ (δ = 1.83–1.89 ppm, ^3^*J*_(H,H)_ = 6.8 Hz) and 18-H_x_ (δ = 1.79–1.85 ppm, ^3^*J*_(H,H)_ = 6.8 Hz), suggest the contribution of other conformations apart from that shown in Scheme 3, for which a higher value of the above vicinal coupling constants would be expected.

**Figure 1:**
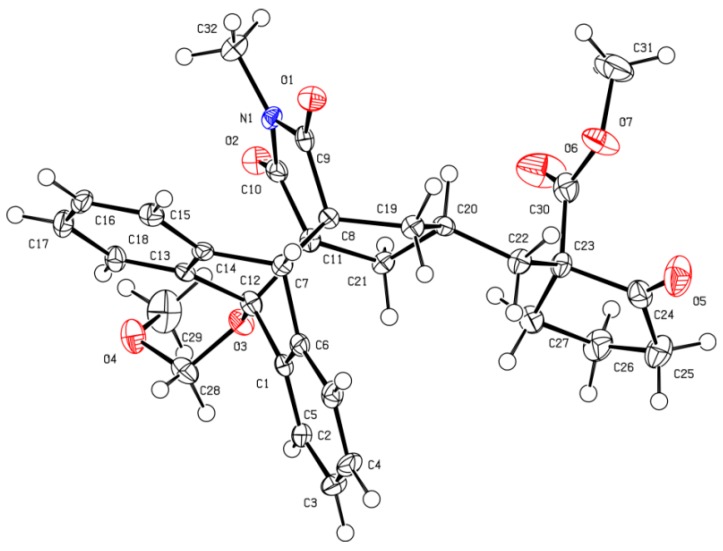
ORTEP representation of compound **24**.

The mesylation of alcohol **19** under the usual conditions gave, after purification of the crude product by silica gel column chromatography, mesylate **21**, in which the methoxymethyl-protecting group had been hydrolyzed. However, purification of the crude mesylate by basic aluminum oxide column chromatography gave the desired mesylate **22** in high yield. The reaction of mesylate **22** with powdered sodium iodide in refluxing acetone gave iodide **23** in good yield. In one occasion, the methoxymethyl group of compound **23** was hydrolyzed on standing (^1^H-NMR), thus, it is recommended not to stock this compound. In the sequence of Scheme 1, in which the benzyloxymethyl was the hydroxyl-protecting group, no hydrolysis at the level of the corresponding mixtures of mesylates **6** or iodides **7** was ever observed. Reaction of iodide **23** with the sodium salt of methyl 2-oxocyclopentanecarboxylate in DMF gave an essentially 1:1 steroisomeric mixture of the substitution product **24** and the C1′ epimer **25** in 59% yield, after column chromatography. Some elimination product **18** was also isolated in 17% yield. After repeated crystallization of this mixture from EtOAc/hexane, stereoisomer **24** was obtained in pure form. The structure of **24** was established by X-ray diffraction analysis (Figure 1). Although compounds **17** to **27** of this synthetic sequence are racemic, the unit cell of the crystal used for the X-ray analysis of **24** contained four molecules of the same enantiomer whose absolute configuration could not be established from the X-ray data [6]. Reaction of the stereoisomeric mixture of keto esters **24** and **25** with trimethylsilyl triflate gave the corresponding mixture of trimethylsilyl enol ethers that was oxidized without purification with Pd(OAc)_2_ in DMSO [7] to give the stereoisomeric mixture of cyclopentenones **26** and **27**, in 75% overall yield. For characterization purposes, the same transformation was carried out with pure stereoisomer **24**, thus, obtaining pure stereoisomer **26**.

Transformation of the stereoisomeric mixture of **26** and **27** to polycycle **13** was carried out without isolating any of the intermediates, as was the case in the sequence of Scheme 1. Thus, reduction of the **26** and **27** mixture with NaBH_4_ in the presence of CeCl_3_·7H_2_O (Luche conditions) [8] gave the diastereomeric mixture of allylic alcohols **28** which on heating in refluxing benzene in the presence of a catalytic amount of *p*-toluenesulfonic acid was dehydrated with simultaneous deprotection of the methoxymethyl group to compound **29**. The oxide anion accelerated retro Diels-Alder reaction of **29** leading to anthrone and maleimide **12** was carried out as usual by treatment with KH in THF [9]. Maleimide **12** experience an intramolecular Diels-Alder reaction producing imide **13** in 30% overall yield from the mixture of **24** and **25**. The overall yield of the synthetic sequence of Scheme 2 (5.1%) is comparable to that of Scheme 1 (7.2%). As expected, no formation of **14** was observed in this case and, worthy of note, the sequence of Scheme 2 made possible the characterization of all of the intermediates as pure racemates. The lower stability of the methoxymethyl hydroxyl-protecting group compared with the benzyloxymethyl required using neutral aluminum oxide instead of silica gel during the column chromatography purification.

Additionally, the imide function of compound **13** has been transformed into the corresponding diacid (compound **32**, Scheme 3), a transformation that is worth of mention. Thus, basic hydrolysis of **13** took place smoothly at room temperature leading cleanly to the amide acid **30**. This result was surprising taking into account that both α-carbon atoms of the imide function are quaternary. In fact, only a closely-related example of this kind of hydrolysis has been previously described, although in this case the reaction was carried out at 50 °C [10]. Under these conditions the ester function, whose α-carbon atom is also quaternary, was not hydrolyzed. We consider that the easy hydrolysis of imide **13** might take place as follow: (i) intramolecular retro-Diels-Alder to revert to maleimide **12**; (ii) fast basic hydrolysis of maleimide **12**, in accord with our previous experience with related maleimides [2]; and (iii) intramolecular Diels-Alder reaction to give **30**. Under more forcing basic conditions the ester function of **30** was fully hydrolyzed while only partial hydrolysis of the amide function was observed. However, amide acid **30** was transformed into anhydride **31** by the reaction with NaNO_2_ in a 1:1 mixture of AcOH and Ac_2_O at room temperature. It is known that *N*-alkyl-*N*-nitrosoamides decompose thermally to alkyl esters [11,12]. In the present case, the expected ester from the decomposition of the *N*-methyl-*N*-nitrosoamide, on reaction with the neighbor carboxylate under the reaction conditions would give anhydride **31**. Alternatively, addition of the carboxylate group to the carbonyl of the neighbor *N*-nitrosoamide followed by elimination of the *N*-methyl-*N*-nitrosoamide anion would give anhydride **31**. Following a related procedure [13], heating anhydride **31** in water under reflux, diacid **32** was obtained in high yield.

## 3. Materials and Methods

### General

Melting points were determined in open capillary tubes with a MFB 595010M Gallenkamp melting point apparatus (Weiss Gallenkamp, Loughborough, UK). All new compounds were fully characterized by their analytical [melting point, elemental analysis and/or accurate mass measurement, spectroscopic data (IR, ^1^H-NMR and ^13^C-NMR, see supplementary)] and, in the case of compound **24**, also X-ray diffraction analysis. Assignments given for the NMR spectra are based on DEPT, COSY, ^1^H/^13^C single quantum correlation (gHSQC sequence) and ^1^H/^13^C multiple bond correlation (gHMBC sequence) spectra and by comparison with previous assignments for the benzyloxymethyl series. ^1^H-NMR and ^13^C-NMR spectra were recorded on a Varian Mercury 400 (400 MHz for ^1^H and 100.6 MHz for ^13^C, Varian, Palo Alto, CA, USA) spectrometer. Unless otherwise stated, the NMR spectra have been performed in CDCl_3_. Chemical shifts (δ) are reported in parts per million related to internal TMS or CDCl_3_ for ^1^H- and ^13^C-NMR, respectively. Multiplicities are reported using the following abbreviations: s, singlet; d, doublet; t, triplet; m, multiplet; br, broad; or their combinations. IR spectra were registered on a FTIR Perkin-Elmer Spectrum RX1 spectrometer (Perkin-Elmer, Seer Green, UK) using the attenuated total reflectance (ATR) technique. Absorption values are given as wavenumbers (cm^−1^), the intensity of the absorptions are given as strong (s), medium (m) or weak (w). IR and NMR spectra of all new compounds are provided in the Supplementary Materials section of this paper.High resolution mass spectra (HRMS) were carried out at the Mass Spectrometry Unity of the Centres Científics i Tecnològics of the Universitat de Barcelona (CCiTUB, Barcelona, Spain) and are reported as *m*/*z*. LC/MSD-TOF spectrometer with electrospray ionization (ESI-TOF-MS) from Agilent Technologies (Santa Clara, CA, USA) was used. The elemental analyses were carried out at the IQAC (CSIC) of Barcelona, Spain, in elemental microanalyzers (A5) model Flash 1112 series from Thermofinnigan for (C, H, N) determinations (Thermo Fisher Scientific SL, Barcelona, Spain) and in a titroprocessor Methrom model 808 (Massó Analítica, Barcelona, Spain) for the halogen determination. For the flash column chromatography, silica gel 60 AC (35–70 μm, SDS, reference 2000027) or neutral Al_2_O_3_ (50–200 μm) was used. The eluents employed are reported as volume/volume percentages. Thin-layer chromatography (TLC) was performed on aluminum-backed sheets with silica gel 60 F254 (Sigma Aldrich Química, S.L., Madrid, Spain, Merck reference 1.05554) and spots were visualized with UV light or a 1% aqueous solution of KMnO_4_. X-ray diffraction analysis of compounds 24 was performed in a Bruker D8 Venture diffractometer at the CCiTUB of the University of Barcelona (Bruker Española S.A., Madrid, Spain). The compounds and reagents were purchased to the following companies: methyl 2-oxocyclopentanecarboxylate, methyl chloromethyl ether, silica gel, 60% NaH and 30% KH, both in mineral oil, and *p*-toluenesulfonic acid to Sigma-Aldrich; anthrone and Pd(OAc)_2_ to Alfa Aesar; *N*-methylmaleimide and NaBH_4_ to TCI; 3-chloro-2-chloromethyl-1-propene to Secant Chemicals, Inc., Winchendon, MA, USA; borane THF complex, *n*-BuLi in hexanes, neutral aluminum oxide Brockman I (50–200 μm), methanesulfonyl chloride, and NaI to ACROS Organics; diisopropylamine and 35% H_2_O_2_ to Panreac; trimethylsilyl triflate, CeCl_3_·7H_2_O, and NaNO_2_ to Fluka. All of them were used without further purification.

*9-(Methoxymethoxy)anthracene* (**16**). NaH (60% in mineral oil, 3.09 g, 77 mmol) was added in portions to a cold (0 °C, ice-water bath) solution of anthrone 15 (10.0 g, 51.5 mmol) in anhydrous THF (600 mL) and the mixture was stirred for 45 min at this temperature. Methoxymethyl chloride (5.87 mL, 6.22 g, 77.3 mmol) were added at 0 °C and the mixture was stirred for 12 h at room temperature. Water (250 mL) and EtOAc (300 mL) were added, the organic phase was separated and the aqueous one was extracted with EtOAc (2 × 300 mL). The combined organic phases were washed with water (250 mL) and brine (250 mL), dried (anhydrous Na_2_SO_4_) and concentrated in vacuo to give **16** (11.6 g, 95% yield). An analytical sample of **16** (105 mg) was obtained as yellow solid by crystallization of a sample of the above product (300 mg) from a mixture of hexane (5 mL) and EtOAc (2 mL). m.p. 78–79 °C (EtOAc/hexane); ^1^H-NMR (400 MHz, CDCl_3_): δ = 3.76 (s, 3H, OCH_3_), 5.38 (s, 2H, OCH_2_O), 7.45–7.52 [complex signal, 4H, 2(7)-H and 3(6)-H], 7.98–8.01 [m, 2H, 4(5)-H], 8.26 (s, 1H, 10-H), 8.31–8.34 ppm [m, 2H, 1(8)-H]; ^13^C-NMR (100.6 MHz, CDCl_3_): δ = 58.1 (CH_3_, OCH_3_), 101.2 (CH_2_, OCH_2_O), 122.6 [CH, C1(8)], 122.7 (CH, C10), 125.0 [C, C8a(9a)], 125.3 [CH, C2(7)], 125.5 [CH, C3(6)], 128.3 [CH, C4(5)], 132.3 (C, C4a(4b)], 149.7 ppm (C, C9); IR (ATR): *ṽ* = 1675 (w), 1343 (m), 1309 (m), 1285 (m), 1157 (m), 1098 (m), 1040 (s), 1008 (m), 933 (s), 924 (s), 884 (m), 846 (m), 791 (m), 732 (s), 693 (m), 658 cm^−1^ (m); accurate mass measurement: *m*/*z* calcd. for C_16_H_14_O_2_ + H^+^: 239.1067; found: 239.1056; elemental analysis calcd. (%) for C_16_H_14_O_2_: C 80.65, H 5.92; found: C 80.65, H 5.84.

*4-(Methoxymethoxy)-2-methyl-3a,4,9,9a-tetrahydro-4,9[1′,2′]benzeno-1H-benz[f]isoindole-1,3(2H)-dione* (**17**). A solution of **16** (8.20 g, 34.2 mmol) and *N*-methylmaleimide **1** (3.80 g, 34.2 mmol) in xylene (350 mL) was heated at 140 °C for 12 h. The solution was allowed to cool to room temperature and was concentrated in vacuo to give adduct **17** as yellow solid (11.6 g, 97% yield). An analytical sample of **17** (83 mg) was obtained as yellow solid by crystallization of a sample of the above product (200 mg) from a mixture of hexane (3 mL) and EtOAc (1 mL). m.p. 168–169 °C (EtOAc/hexane); ^1^H-NMR (400 MHz, CDCl_3_): δ = 2.50 (s, 3H, *N*-C*H*_3_), 3.31 (dd, ^3^*J*_(H,H)_ = 8.4 Hz, ^3^*J*_(H,H)_ = 3.2 Hz, 1H, 9a-H), 3.44 (d, ^3^*J*_(H,H)_ = 8.4 Hz, 1H, 3a-H), 3.82 (s, 3H, OCH_3_), 4.70 (d, ^3^*J*_(H,H)_ = 3.2 Hz, 1H, 9-H), 5.53 (d, ^2^*J*_(H,H)_ = 5.4 Hz, 1H) and 5.55 (d, ^2^*J*_(H,H)_ = 5.4 Hz, 1H) (OCH_2_O), 7.12 (pseudo dt, ^4^*J*_(H,H)_ = 1.2 Hz, ^3^*J*_(H,H)_ = 7.4 Hz, 1H, 7-H), 7.17 (overlapped pseudo dt, ^4^*J*_(H,H)_ = 1.2 Hz, ^3^*J*_(H,H)_ = 7.2 Hz, 1H, 6-H), 7.20 (overlapped pseudo dt, ^4^*J*_(H,H)_ = 1.2 Hz, ^3^*J*_(H,H)_ = 7.3 Hz, 1H, 12-H), 7.21–7.26 (complex signal, 2H, 8-H and 13-H), 7.38 (dd, ^3^*J*_(H,H)_ = 7.4 Hz, ^4^*J*_(H,H)_ = 1.4 Hz, 1H, 11-H), 7.65 (dd, ^3^*J*_(H,H)_ = 7.2 Hz, ^4^*J*_(H,H)_ = 1.2 Hz, 1H, 5-H), 7.68 ppm (dd, ^3^*J*_(H,H)_ = 7.6 Hz, ^4^*J*_(H,H)_ = 1.2 Hz, 1H, 14-H); ^13^C-NMR (100.6 MHz, CDCl_3_): δ = 24.3 (CH_3_, *N*-*C*H_3_), 44.6 (CH, C9), 47.4 (CH, C3a), 47.9 (CH, C9a), 57.1 (CH_3_, OCH_3_), 81.8 (C, C4), 93.8 (CH_2_, OCH_2_O), 121.8 (CH, C5), 122.2 (CH, C14), 124.0 (CH, C11), 124.4 (CH, C8), 126.6 (CH, C13), 126.9 (CH, C12), 127.00 (CH, C7), 127.03 (CH, C6), 136.2 (C, C8a), 139.7 (C, C4a), 140.2 (C, C10), 141.6 (C, C15), 174.1 (C, C1), 176.2 ppm (C, C3); IR (ATR): *ṽ* = 1692 (s), 1457 (m), 1430 (m), 1292 (m), 1276 (m) 1163 (m), 1124 (m), 1050 (m), 1013 (m), 963 (m), 771 (m), 738 cm^−1^ (s); accurate mass measurement: *m*/*z* calcd. for C_21_H_19_NO_4_ + Na^+^: 372.1206; found: 372.1198; elemental analysis calcd. (%) for C_21_H_19_NO_4_: C 72.19, H 5.48, N 4.01; found: C 72.08, H 5.42, N 3.79.

*4-[(Methoxy)methoxy)-2-methyl-17-methylene-4,9-dihydro-4,9[1′,2′]benzeno-3a,9a-propano-1H-benz[f]isoindole-1,3(2H)-dione* (**18**). A solution of *n*-BuLi in hexanes (2.75 mL, 2.5 M, 6.87 mmol) was added dropwise to a cold (−78 °C, acetone/solid CO_2_ bath) and magnetically-stirred solution of diisopropylamine (0.97 mL, 6.9 mmol) in anhydrous THF (18 mL) under an Ar atmosphere. When *n*-BuLi addition was finished, the solution was allowed to warm to 0 °C for 1 h, it was cooled again to −78 °C, and a solution of **17** (1.00 g, 2.86 mmol) in anhydrous THF (18 mL) was added dropwise. Then, the solution was stirred a −78 °C for 15 min and allowed to warm to 0 °C for 1 h. The solution was again cooled to −78 °C and 3-chloro-2-(chloromethyl)-1-propene (0.48 mL 96% content, 498 mg, 3.98 mmol) was added dropwise. The reaction mixture was allowed to warm to room temperature and it was stirred for three days at this temperature. The mixture was made acidic with aqueous 2 N HCl (8 mL) and was extracted with Et_2_O (3 × 40 mL). The combined organic extracts were dried (anhydrous Na_2_SO_4_) and concentrated in vacuo to give a brown waxy residue (1.29 g) that was subjected to column chromatography (silica gel 35–70 μm, 65 g, hexane/EtOAc mixtures) to give an elution with hexane/EtOAc 19:1 to 9:1, product **18** (801 mg, 70% yield) as yellow solid. m.p 150–152 °C (EtOAc/hexane); ^1^H-NMR (400 MHz, CDCl_3_): δ = 1.99–2.04 (dm, ^2^*J*_(H,H)_ = 15.2 Hz, 1H, 16-H_n_), 2.25–2.31 (dm, ^2^*J*_(H,H)_ = 15.8 Hz, 1H, 18-H_n_), 2.45 (s, 3H, *N*-CH_3_), 2.59 (broad d, ^2^*J*_(H,H)_ = 15.2 Hz, 1H, 16-H_x_), 2.68 (broad d, ^2^*J*_(H,H)_ = 15.8 Hz, 1H, 18-H_x_), 3.80 (s, 3H, OCH_3_), 4.44 (s, 1H, 9-H), 4.58–4.60 (m, 1H, 17=C*H*_a_), 4.60–4.62 (m, 1H, 17=C*H*_s_), 5.37 (d, ^2^*J*_(H,H)_ = 5.6 Hz, 1H, OC*H*_a_O), 5.40 (d, ^2^*J*_(H,H)_ = 5.6 Hz, 1H, OC*H*_b_O), 7.13 (pseudo dt, ^4^*J*_(H,H)_ = 1.4 Hz, ^3^*J*_(H,H)_ = 7.2 Hz, 1H, 7-H), 7.18 (pseudo dt, ^4^*J*_(H,H)_ = 1.5 Hz, ^3^*J*_(H,H)_ = 7.6 Hz, 1H, 6-H), 7.22–7.27 (complex signal, 2H, 8-H, 12-H), 7.28 (overlapped pseudo dt, ^4^*J*_(H,H)_ = 1.6 Hz, ^3^*J*_(H,H)_ = 7.2 Hz, 1H, 13-H), 7.37–7.39 (ddm, ^3^*J*_(H,H)_ = 7.2 Hz, ^4^*J*_(H,H)_ = 1.6 Hz, 1H, 11-H), 7.74–7.77 ppm (broad d, ^3^*J*_(H,H)_ = 7.6 Hz, 2H, 5-H and 14-H); ^13^C-NMR (100.6 MHz, CDCl_3_): δ = 24.6 (CH_3_, *N*-*C*H_3_), 38.2 (CH_2_, C18), 39.0 (CH_2_, C16), 48.7 (CH, C9), 56.4 (CH_3_, OCH_3_), 64.1 (C, C3a), 64.8 (C, C9a), 87.1 (C, C4), 94.2 (CH_2_, OCH_2_O), 108.9 (CH_2_, 17=*C*H_2_), 122.7 (CH, C5), 124.3 (CH, C14), 124.8 (CH, C8), 126.3 (CH, C11), 126.9 (CH, C13), 127.0 (CH, C6), 127.20 (CH) and 127.21 (CH) (C7 and C12), 138.15 (C, C8a), 138.21 (C, C10), 139.4 (C, C4a), 139.9 (C, C15), 146.6 (C, C17), 177.2 (C, C3), 179.4 ppm (C, C1); IR (ATR): *ṽ* = 1696 (s), 1456 (m), 1428 (m), 1373 (m), 1304 (m) 1158 (m), 1092 (m), 1020 (m), 987 (m), 930 (m), 773 (m), 758 cm^−1^ (m); accurate mass measurement: *m*/*z* calcd. for C_25_H_23_NO_4_ + Na^+^: 424.1519. found: 424.1521; elemental analysis calcd. (%) for C_25_H_23_NO_4_: C 74.80, H 5.77, N 3.49; found: C 74.92, H 5.74, N 3.39.

*Syn-17-(hydroxymethyl)-4-(methoxymethoxy)-2-methyl-4,9-dihydro-4,9[1′,2′]benzeno-3a,9a-propano-1H-benz[f]isoindole-1,3(2H)-dione* (**19**) and *anti-17-(hydroxymethyl)-4-(methoxymethoxy)-2-methyl-4,9-dihydro-4,9[1’,2’]benzeno-3a,9a-propano-1H-benz[f]isoindole-1,3(2H)-dione* (**20**). A solution of the BH_3_ THF complex in anhydrous THF (26.5 mL, 1 M in THF, 26.5 mmol) was added dropwise to a cold (0 °C, ice-water bath) and magnetically-stirred solution of compound **18** (4.62 g, 11.5 mmol) in anhydrous THF (150 mL) under an Ar atmosphere, and the reaction mixture was stirred at 0 °C for 4 h. After addition of EtOH (12.7 mL), the mixture was allowed to warm to room temperature, and aqueous solutions of 35% H_2_O_2_ (9.4 mL) and 3 M NaOH (14.8 mL) were simultaneously added dropwise in 15 min, occasionally cooling with a water bath, and the reaction mixture was stirred at room temperature for 15 min. Water (50 mL) and EtOAc (100 mL) were added, the organic phase was separated and the aqueous one was extracted with EtOAc (2 × 100 mL). The combined organic phases were dried (anhydrous Na_2_SO_4_) and concentrated to dryness in vacuo to give a white solid (5.5 g) that was subjected to column chromatography (silica gel 35–70 μm, 165 g, hexane/EtOAc mixtures). Alcohol **19** (1.40 g), a 2:1 mixture of **19** and **20** (1.174 g) and alcohol **20** (330 mg) were successively eluted as white solids with hexane/EtOAc 3:2, 1:1, and 2:3, respectively (overall yield: 2.56 g, 72%, approximate ratio **19**/**20** = 2.8/1 (^1^H-NMR). 

Analytical and spectroscopic data of **19**. White solid, m.p. 216–222 °C (EtOAc/hexane); ^1^H-NMR (400 MHz, CDCl_3_): δ = 0.92 (t, ^2^*J*_(H,H)_ = ^3^*J*_(H,H)_ = 12.8 Hz, 1H, 16-H_n_), 1.14 (t, ^2^*J*_(H,H)_ = ^3^*J*_(H,H)_ = 12.8 Hz, 1H, 18-H_n_), 1.20–1.34 (broad s, OH), 1.84–1.97 (m, 1H, 17-H), 2.16 (ddd, ^2^*J*_(H,H)_ = 12.8 Hz, ^3^*J*_(H,H)_ = 6.0 Hz, ^4^*J*_(H,H)_ = 1.6 Hz, 1H, 16-H_x_), 2.28 (ddd, ^2^*J*_(H,H)_ = 12.8 Hz, ^3^*J*_(H,H)_ = 6.0 Hz, ^4^*J*_(H,H)_ = 1.2 Hz, 1H, 18-H_x_), 2.46 (s, 3H, *N*-CH_3_), 3.20 (dd, ^2^*J*_(H,H)_ = 11.2 Hz, ^3^*J*_(H,H)_ = 5.6 Hz, 1H, C*H_a_*OH), 3.23 (dd, ^2^*J*_(H,H)_ = 11.2 Hz, ^3^*J*_(H,H)_ = 5.6 Hz, 1H, C*H_b_*OH), 3.78 (s, 3H, OCH_3_), 4.40 (s, 1H, 9-H), 5.36 (d, ^2^*J*_(H,H)_ = 5.6 Hz, 1H, OCH_a_O), 5.39 (d, ^2^*J*_(H,H)_ = 5.6 Hz, 1H, OCH_b_O), 7.11 (pseudo dt, ^4^*J*_(H,H)_ = 1.6 Hz, ^3^*J*_(H,H)_ = 7.2 Hz, 1H, 7-H), 7.16 (pseudo dt, ^4^*J*_(H,H)_ = 1.6 Hz, ^3^*J*_(H,H)_ = 7.4 Hz, 1H, 6-H), 7.21 (overlapped dd, ^3^*J*_(H,H)_ = 7.2 Hz, ^4^*J*_(H,H)_ = 1.6 Hz, 1H, 8-H), 7.24 (overlapped pseudo dt, ^4^*J*_(H,H)_ = 1.2 Hz, ^3^*J*_(H,H)_ = 6.8 Hz, 1H, 12-H), 7.28 (overlapped pseudo dt,*^4^J*_(H,H)_ = 1.4 Hz, ^3^*J*_(H,H)_ = 7.3 Hz, 1H, 13-H), 7.37 (dd, ^3^*J*_(H,H)_ = 6.6 Hz, ^4^*J*_(H,H)_ = 1.4 Hz, 1H, 11-H), 7.70–7.73 (ddm, ^3^*J*_(H,H)_ = 7.4 Hz, ^4^*J*_(H,H)_ = 1.6 Hz, 1H, 5-H), 7.75–7.78 ppm (ddm, ^3^*J*_(H,H)_ = 7.6 Hz, ^4^*J*_(H,H)_ = 1.6 Hz, 1H, 14-H); ^13^C-NMR (100.6 MHz, CDCl_3_): δ = 24.6 (CH_3_, *N*-*C*H_3_), 34.4 (CH_2_, C18), 35.0 (CH_2_, C16), 42.5 (CH, C17), 48.6 (CH, C9), 56.4 (CH_3_, OCH_3_), 64.9 (C, C9a), 65.0 (CH_2_, CH_2_OH), 65.4 (C, C3a), 87.0 (C, C4), 94.1 (CH_2_, OCH_2_O), 122.7 (CH, C5), 124.3 (CH, C14), 124.7 (CH, C8), 126.4 (CH, C11), 126.7 (CH, C13), 126.9 (CH, C6), 127.1 (2CH, C7 and C12), 138.3 (C, C8a), 138.4 (C, C10), 139.6 (C, C4a), 139.9 (C, C15), 177.5 (C, C3), 179.7 ppm (C, C1); IR (ATR): *ṽ* = 3457 (m), 1767 (w), 1685 (s), 1433 (m), 1378 (m), 1299 (m), 1165 (m), 1145 (m), 1072 (m), 1053 (s), 1004 (s), 794 (m), 760 cm^−1^ (s); accurate mass measurement: *m*/*z* calcd. for C_25_H_25_NO_5_ + Na^+^ 442.1625; found: 442.1616; elemental analysis calcd. (%) for C_25_H_25_NO_5_: C 71.58, H 6.01, N 3.34; found: C 71.87, H 5.99, N 3.24. 

Analytical and spectroscopic data of **20**. White solid, m.p. 186–187 °C (EtOAc/hexane); ^1^H-NMR (400 MHz, CDCl_3_): δ = 1.09–1.20 (m, 1H, 17-H), 1.27–1.35 (broad s, 1H, OH), 1.71 (dd, ^2^*J*_(H,H)_ = 14.0 Hz, ^3^*J*_(H,H)_ = 7.6 Hz, 1H, 16-H_n_), 1.79–1.85 (overlapped dd, ^2^*J*_(H,H)_ = 14.2 Hz, ^3^*J*_(H,H)_ = 6.8 Hz, 1H, 18-H_x_), 1.83–1.89 (overlapped dd, ^2^*J*_(H,H)_ = 14.0 Hz, ^3^*J*_(H,H)_ = 6.8 Hz, 1H, 16-H_x_), 2.12 (dd, ^2^*J*_(H,H)_ = 14.2 Hz, ^3^*J*_(H,H)_ = 7.8 Hz, 1H, 18-H_n_), 2.41 (s, 3H, *N*-CH_3_), 3.19 (broad d, ^3^*J*_(H,H)_ = 5.2 Hz, 2H, C*H*_2_OH), 3.79 (s, 3H, OCH_3_), 4.43 (s, 1H, 9-H), 5.36 (d, ^2^*J*_(H,H)_ = 5.8 Hz, 1H) and 5.39 (d, ^2^*J*_(H,H)_ = 5.8 Hz) (OCH_2_O), 7.11–7.16 (overlapped pseudo dt, ^4^*J*_(H,H)_ = 1.2 Hz, ^3^*J*_(H,H)_ = 7.6 Hz, 1H, 7-H), 7.14–7.19 (overlapped pseudo dt, ^4^*J*_(H,H)_ = 1.4 Hz, ^3^*J*_(H,H)_ = 7.6 Hz, 1H, 6-H), 7.22–7.24 (overlapped dd, ^4^*J*_(H,H)_ = 1.2 Hz, ^3^*J*_(H,H)_ = 7.0 Hz, 1H, 8-H), 7.24–7.28 (overlapped pseudo dt, ^4^*J*_(H,H)_ = 1.2 Hz, ^3^*J*_(H,H)_ = 7.6 Hz, 1H, 12-H), 7.28–7.32 (overlapped pseudo dt, ^4^*J*_(H,H)_ = 1.6 Hz, ^3^*J*_(H,H)_ = 8.0 Hz, 1H, 13-H), 7.39 (dd, 4*J*_(H,H)_ = 1.4 Hz, ^3^*J*_(H,H)_ = 7.4 Hz, 1H, 11-H), 7.74 (dd, ^4^*J*_(H,H)_ = 1.6 Hz, ^3^*J*_(H,H)_ = 7.2 Hz, 1H, 5-H), 7.79 ppm (dd, ^4^*J*_(H,H)_ = 1.2 Hz, ^3^*J*_(H,H)_ = 7.6 Hz, 1H, 14-H); ^13^C-NMR (100.6 MHz, CDCl_3_): δ = 24.4 (CH_3_, *N*-*C*H_3_), 33.0 (CH_2_, C18), 35.2 (CH_2_, C16), 43.5 (CH, C17), 49.9 (CH, C9), 56.4 (CH_3_, OCH_3_), 65.4 (C, C9a), 65.5 (CH_2_, CH_2_OH), 66.5 (C, C3a), 87.5 (C, C4), 94.4 (CH_2_, OCH_2_O), 122.7 (CH, C5), 124.0 (CH, C14), 124.8 (CH, C8), 125.8 (CH, C11), 126.9 (2 CH, C6 and C13), 127.2 (CH, C7), 127.3 (CH, C12), 138.3 (C, C10), 138.4 (C, C8a), 139.1 (C, C4a), 140.0 (C, C15), 177.6 (C, C3), 179.7 ppm (C, C1); IR (ATR): *ṽ* = 3471 (m), 2946 (w), 2935 (w), 2855 (w), 1768 (w), 1691 (s), 1456 (m), n 1434 (m), 1381 (m), 1300 (m), 1162 (m), 1070 (m), 1043 (s), 1014 (m), 990 (s), 956 (s), 929 (m), 773 (m), 756 (m), 720 cm^−1^ (m); accurate mass measurement: *m*/*z* calcd. for C_25_H_25_NO_5_ + H^+^ 420.1805; found: 420.1815; elemental analysis calcd. (%) for C_25_H_25_NO_5_: C 71.58, H 6.01, N 3.34; found: C 71.32, H 5.81, N 3.11.

*Syn-4-hydroxy-17-(methanesulfonyloxymethyl)-2-methyl-4,9-dihydro-4,9[1′,2′]benzeno-3a,9a-propano-1H-benz[f]isoindole-1,3(2H)-dione* (**21**). MsCl (60 μL, 86 mg, 0.75 mmol) was added dropwise to a cold solution (0 °C, ice-water bath) of alcohol **19** (300 mg, 0.72 mmol) and anhydrous Et_3_N (0.23 mL, 166 mg, 1.65 mmol) in anhydrous CH_2_Cl_2_ (14 mL) under an Ar atmosphere, and the mixture was stirred for 4 h at 0 °C. Saturated aqueous solution of NaHCO_3_ (4 mL) and water (14 mL) were successively added to the reaction mixture. The organic phase was separated and the aqueous one was extracted with CH_2_Cl_2_ (2 × 20 mL). The combined organic phases were successively washed with aqueous 1 N HCl (3 × 15 mL), water (20 mL) and brine (20 mL), dried (anhydrous Na_2_SO_4_) and concentrated to dryness in vacuo to give a white solid (370 mg), that was subjected to column chromatography (silica gel 35–70 μm, 12 g, hexane/EtOAc mixtures) to give mesylate **21** (223 mg, 68% yield) as white solid, on elution with hexane/EtOAc 3:2 to 1:1. An analytical sample of **21** (65 mg) was obtained as white solid by crystallization of a sample of the above product (120 mg) from EtOAc (8 mL). m.p. 228–232 °C (EtOAc/hexane); ^1^H-NMR (400 MHz, CDCl_3_): δ = 1.13 (t, ^2^*J*_(H,H)_ = ^3^*J*_(H,H)_ = 12.6 Hz, 1H, 16-H_n_), 1.23 (t, ^2^*J*_(H,H)_ = ^3^*J*_(H,H)_ = 12.6 Hz, 1H, 18-H_n_), 2.09–2.21 (m, 1H, 17-H), 2.17–2.23 (ddm, ^2^*J*_(H,H)_ = 12.8 Hz, ^3^*J*_(H,H)_ = 6.0 Hz, 1H, 18-H_x_), 2.30–2.36 (ddm, ^2^*J*_(H,H)_ = 12.8 Hz, ^3^*J*_(H,H)_ = 6.0 Hz, 1H, 16-H_x_), 2.50 (s, 3H, *N*-CH_3_), 2.85 (s, 3H, CH_3_SO_3_), 3.81 (d, ^3^*J*_(H,H)_ = 5.6 Hz, 2H, C*H_2_*OMs), 4.17 (s, 1H, OH), 4.43 (s, 1H, 9-H), 7.11 (pseudo dt, ^4^*J*_(H,H)_ = 1.2 Hz, ^3^*J*_(H,H)_ = 7.2 Hz, 1H, 7-H), 7.18 (pseudo dt, ^4^*J*_(H,H)_ = 1.2 Hz, ^3^*J*_(H,H)_ = 7.6 Hz, 1H, 6-H), 7.19–7.21 (overlapped dm, ^3^*J*_(H,H)_ = 7.2 Hz, 1H, 8-H), 7.20–7.24 (overlapped pseudo dt, ^4^*J*_(H,H)_ = 1.2 Hz, ^3^*J*_(H,H)_ = 7.6 Hz, 1H, 12-H), 7.30 (pseudo dt, ^4^*J*_(H,H)_ = 1.3 Hz, ^3^*J*_(H,H)_ = 7.5 Hz, 1H, 13-H), 7.36 (dd, ^3^*J*_(H,H)_ = 7.2 Hz, ^4^*J*_(H,H)_ = 1.2 Hz, 1H, 11-H), 7.41–7.43 (dm, ^3^*J*_(H,H)_ = 7.2 Hz, 1H, 5-H), 7.68–7.71 ppm (dm, ^3^*J*_(H,H)_ = 7.2 Hz, 1H, 14-H); ^13^C-NMR (100.6 MHz, CDCl_3_): δ = 24.7 (CH_3_, *N*-*C*H_3_), 33.1 (CH_2_, C18), 35.2 (CH_2_, C16), 37.4 (CH_3_, CH_3_SO_3_), 39.2 (CH, C17), 48.0 (CH, C9), 63.6 (C, C9a), 63.9 (C, C3a), 69.4 (CH_2_, CH_2_OMs), 79.6 (C, C4), 121.0 (CH, C5), 123.1 (CH, C14), 124.5 (CH, C8), 126.1 (CH, C11), 126.9 (CH, C12), 127.0 (2 CH, C6 and C13), 127.1 (CH, C7), 137.6 (C, C10), 137.7 (C, C8a), 140.3 (C, C15), 141.4 (C, C4a), 179.5 (C, C1), 180.9 ppm (C, C3); IR (ATR): *ṽ* = 3450–3350 (w, max. at 3498 and 3385), 3028 (w), 2940 (w), 1767 (w), 1684 (s), 1454 (m), 1436 (m), 1351 (s), 1173 (s), 1072 (m), 1013 (m), 977 (s), 956 (s), 832 (m), 793 (m), 754 cm^−1^ (s); accurate mass measurement: *m*/*z* calcd. for C_24_H_23_NO_6_S + Na^+^: 476.1138; found: 476.1143; elemental analysis calcd. (%) for C_24_H_23_NO_6_S: C 63.56, H 5.11, N 3.09, S 7.07; found: C 63.26, H 5.23, N 2.80, S 6.22.

*Syn-17-(methanesulfonyloxymethyl)-4-(metoxymethoxy)-2-methyl-4,9-dihydro-4,9[1′,2′]benzeno-3a,9a-propano-1H-benz[f]isoindole-1,3(2H)-dione* (**22**). MsCl (0.23 mL, 334 mg, 2.92 mmol) was added dropwise to a cold solution (0° C, ice-water bath) of alcohol **19** (1.16 g, 2.77 mmol) and anhydrous Et_3_N (0.89 mL, 646 mg, 6.4 mmol) in anhydrous CH_2_Cl_2_ (60 mL) under an Ar atmosphere and the mixture was stirred for 4 h at 0 °C. Saturated aqueous solution of NaHCO_3_ (15 mL) and water (30 mL) were successively added to the reaction mixture. The organic phase was separated and the aqueous one was extracted with CH_2_Cl_2_ (2 × 30 mL). The combined organic phases were successively washed with aqueous 1 N HCl (3 × 30 mL), water (30 mL) and brine (30 mL), dried (anhydrous Na_2_SO_4_) and concentrated to dryness in vacuo to give a white solid (1.32 g), that was subjected to column chromatography (40 g neutral Al_2_O_3_, hexane/EtOAc mixtures) to give mesylate **22** (1.25 g, 91% yield as a white solid on elution with hexane/EtOAc 4:1 to 7:3. An analytical sample of **22** (54 mg) was obtained as white solid by crystallization of a sample of the above product (130 mg) from EtOAc (3 mL). m.p. 71–76 °C (dec) (EtOAc); ^1^H-NMR (400 MHz, CDCl_3_): δ = 0.96 (t, ^2^J_(H,H)_ = ^3^J_(H,H)_ = 12.8 Hz, 1H, 16-H_n_), 1.20 (t, ^2^J_(H,H)_ = ^3^J_(H,H)_ = 12.8 Hz, 1H, 18-H_n_), 2.03–2.15 (m, 1H, 17-H), 2.17–2.23 (ddm, ^2^J_(H,H)_ = 12.8 Hz, ^3^J_(H,H)_ = 6.0 Hz, 1H, 16-H_x_), 2.30–2.36 (ddm, ^2^J_(H,H)_ = 12.8 Hz, ^3^J_(H,H)_ = 6.0 Hz, 1H, 18-H_x_), 2.47 (s, 3H, N-CH_3_), 2.85 (s, 3H, CH_3_SO_3_), 3.79 (overlapped s, 3H, OCH_3_), 3.79–3.80 (overlapped d, 2H, CH_2_OMs), 4.41 (s, 1H, 9-H), 5.35 (d, ^2^J_(H,H)_ = 6.0 Hz, 1H, OCH_a_O), 5.37 (d, ^2^J_(H,H)_ = 6.0 Hz, 1H, OCH_b_O), 7.13 (pseudo dt, ^4^J_(H,H)_ = 1.4 Hz, ^3^J_(H,H)_ = 7.4 Hz, 1H, 7-H), 7.18 (pseudo dt, ^4^J_(H,H)_ = 1.5 Hz, ^3^J_(H,H)_ = 7.6 Hz, 1H, 6-H), 7.23 (dd, ^3^J_(H,H)_ = 7.2 Hz, ^4^J_(H,H)_ = 1.4 Hz, 1H, 8-H), 7.25–7.29 (pseudo dt, ^4^J_(H,H)_ = 1.4 Hz, ^3^J_(H,H)_ = 7.2 Hz, 1H, 12-H), 7.29–7.33 (pseudo dt, ^4^J_(H,H)_ = 1.6 Hz, ^3^J_(H,H)_ = 7.2 Hz, 1H, 13-H), 7.39 (dd, ^3^J_(H,H)_ = 7.2 Hz, ^4^J_(H,H)_ = 1.6 Hz, 1H, 11-H), 7.74–7.76 (overlapped dm, ^3^J_(H,H)_ = 7.2 Hz, 1H, 5-H), 7.76–7.78 ppm (overlapped dm, ^3^J_(H,H)_ = 7.2 Hz, 1H, 14-H); ^13^C-NMR (100.6 MHz, CDCl_3_): δ = 24.7 (CH_3_, N-CH_3_), 34.0 (CH_2_, C18), 34.7 (CH_2_, C16), 37.4 (CH_3_, CH_3_SO_3_), 39.3 (CH, C17), 48.5 (CH, C9), 56.4 (CH_3_, OCH_3_), 64.7 (C, C9a), 65.3 (C, C3a), 69.5 (CH_2_, CH_2_OMs), 87.3 (C, C4), 94.2 (CH_2_, OCH_2_O), 122.8 (CH, C5), 124.4 (CH, C14), 124.8 (CH, C8), 126.4 (CH, C11), 126.9 (CH, C13), 127.0 (CH, C6), 127.2 (CH) and 127.3 (CH) (C7 and C12), 138.0 (C, C8a), 138.2 (C, C10), 139.2 (C, C4a), 139.8 (C, C15), 177.0 (C, C3), 179.1 ppm (C, C1); IR (ATR): ṽ = 3012 (w), 2959 (w), 1769 (w), 1693 (s), 1455 (m), 1430 (m), 1378 (m), 1343 (s), 1171 (s), 1072 (m), 1050 (m), 1006 (m), 987 (s), 955 (s), 926 (s), 825 (s), 794 (m), 754 cm^−1^ (s); accurate mass measurement: m/z calcd. for C_26_H_27_NO_7_S + Na^+^: 520.1400; found: 520.1412; elemental analysis calcd. (%) for C_26_H_27_NO_7_S: C 62.76, H 5.47, N 2.82, S 6.44; found: C 62.87, H 5.48, N 2.69, S 6.26.

*Syn-17-(iodomethyl)-4-(methoxymethoxy)-2-methyl-4,9-dihydro-4,9[1′,2′]benzeno-3a,9a-propano-1H-benz[f]isoindole-1,3(2H)-dione* (**23**). A magnetically stirred solution of mesylate **22** (668 mg, 1.34 mmol) and NaI (2.03 g, 99%, 13.4 mmol) in anhydrous acetone (40 mL) was heated at reflux for 16 h under an Ar atmosphere. The mixture was allowed to cool to room temperature, the precipitate was filtered through a pad of Celite^®^, and the solid was washed with EtOAc (100 mL). The combined filtrate and washings were concentrated in vacuo, and the residue (860 mg) was subjected to column chromatography (30 g 50–200 μm silica gel, hexane/EtOAc mixtures). On elution with hexane/EtOAc 9:1, iodide **23** (617 mg, 87% yield) was obtained as yellow solid. An analytical sample of **23** (72 mg) was obtained as white solid by crystallization of a sample of the above product (100 mg) from EtOAc (0.3 mL) and hexane (0.5 mL). m.p. 176–177 °C (EtOAc/hexane); ^1^H-NMR (400 MHz, CDCl_3_): δ = 0.92 (t, ^2^*J*_(H,H)_ = ^3^*J*_(H,H)_ = 12.8 Hz, 1H, 16-H_n_), 1.15 (t, ^2^*J*_(H,H)_ = ^3^*J*_(H,H)_ = 12.8 Hz, 1H, 18-H_n_), 1.74–1.86 (m, 1H, 17-H), 2.23 (ddd, ^2^*J*_(H,H)_ = 13.0 Hz, ^3^*J*_(H,H)_ = 6.0 Hz, ^4^*J*_(H,H)_ = 1.4 Hz, 1H, 16-H_x_), 2.34 (ddd, ^2^*J*_(H,H)_ = 13.2 Hz, ^3^*J*_(H,H)_ = 6.0 Hz, ^4^*J*_(H,H)_ = 1.4 Hz, 1H, 18-H_x_), 2.47 (s, 3H, *N*-CH_3_), 2.81 (dd, ^2^*J*_(H,H)_ = 10.0 Hz, ^3^*J*_(H,H)_ = 5.6 Hz, 1H, CH*_a_*I), 2.84 (dd, ^2^*J*_(H,H)_ = 10.0 Hz, ^3^*J*_(H,H)_ = 5.6 Hz, 1H, CH*_b_*I), 3.79 (s, 3H, OCH_3_), 4.39 (s, 1H, 9-H), 5.36 (d, ^2^*J*_(H,H)_ = 6.0 Hz, 1H, OC*H_a_*O), 5.37 (d, ^2^*J*_(H,H)_ = 6.0 Hz, 1H, OC*H_b_*O), 7.12 (pseudo dt, ^4^*J*_(H,H)_ = 1.5 Hz, ^3^*J*_(H,H)_ = 7.2 Hz, 1H, 7-H), 7.17 (pseudo dt, ^4^*J*_(H,H)_ = 1.6 Hz, ^3^*J*_(H,H)_ = 7.4 Hz, 1H, 6-H), 7.22 (dd, ^3^*J*_(H,H)_ = 7.2 Hz, ^4^*J*_(H,H)_ = 1.6 Hz, 1H, 8-H), 7.27 (pseudo dt, ^4^*J*_(H,H)_ = 1.4 Hz, ^3^*J*_(H,H)_ = 7.2 Hz, 1H, 12-H), 7.31 (pseudo dt, ^4^*J*_(H,H)_ = 1.6 Hz, ^3^*J*_(H,H)_ = 7.6 Hz, 1H, 13-H), 7.39 (dd, ^4^*J*_(H,H)_ = 1.4 Hz, ^3^*J*_(H,H)_ = 7.2 Hz, 1H, 11-H), 7.73–7.76 (overlapped dm, ^3^*J*_(H,H)_ = 7.6 Hz, 1H, 5-H), 7.75–7.77 ppm (overlapped dm, ^3^*J*_(H,H)_ = 7.6 Hz, 1H, 14-H); ^13^C-NMR (100.6 MHz, CDCl_3_): δ = 8.4 (CH_2_, CH_2_I), 24.7 (CH_3_, *N*-*C*H_3_), 38.0 (CH_2_, C18), 38.7 (CH_2_, C16), 41.5 (CH, C17), 48.6 (CH, C9), 56.4 (CH_3_, OCH_3_), 64.8 (C, C9a), 65.6 (C, C3a), 87.3 (C, C4), 94.1 (CH_2_, OCH_2_O), 122.8 (CH, C5), 124.4 (CH, C14), 124.8 (CH, C8), 126.4 (CH, C11), 127.0 (CH, C6 and C13), 127.2 (CH, C7), 127.3 (CH, C12), 138.1 (C, C8a), 138.2 (C, C10), 139.3 (C, C4a), 139.8 (C, C15), 177.2 (C, C3), 179.3 ppm (C, C1); IR (ATR): *ṽ* = 2936 (w), 1769 (w), 1693 (s), 1453 (m), 1428 (m), 1376 (m), 1299 (m), 1188 (m), 1161 (m), 1093 (m), 1045 (s), 1019 (m), 1008 (m), 991 (m), 759 cm^−1^ (s); accurate mass measurement: *m*/*z* calcd. for C_25_H_24_INO_4_ + Na^+^: 552.0642; found: 552.0630; elemental analysis calcd. (%) for C_25_H_24_INO_4_: C 56.72, H 4.57, N 2.65, I 23.97; found: C 56.98, H 4.56, N 2.53, I 23.39.

(3aR*,9aR*,17S*)-17-{[(1R*)-1-(Methoxycarbonyl)-2-oxocyclopentyl]methyl}-4-(methoxymethoxy)-2-methyl-4,9-dihydro-4,9[1′,2′]benzeno-3a,9a-propano-1H-benz[f]isoindole-1,3(2H)-dione **24** *and mixture of* **24** *and its C1′ epimer* **25**. A solution of methyl 2-oxocyclopentanecarboxylate (0.47 mL, 95% content, 500 mg, 3.5 mmol) was added dropwise to a cold (0 °C, ice-water bath), magnetically-stirred suspension of NaH (117 mg, 60% content, 2.9 mmol) in anhydrous DMF (5 mL) and the mixture was stirred at this temperature for 1 h. Then, a solution of iodide **23** (1.03 g, 1.95 mmol) was added at once, and the reaction mixture was stirred at 0 °C for 2 h and then it was heated at 80 °C for 63 h. A saturated aqueous solution of NH_4_Cl (6 mL) and water (6 mL) were added, and the mixture was extracted with EtOAc (4 × 100 mL). The combined organic extracts were dried (anhydrous Na_2_SO_4_) and concentrated in vacuo. The obtained residue (1.3 g) was subjected to column chromatography (100 g 50–200 μm neutral Al_2_O_3_, hexane/EtOAc mixtures). Alkene **18** (135 mg, 17% yield) was isolated on elution with hexane/EtOAc 4:1. A stereoisomeric mixture of **24** and **25** (about 1:1 mixture of C1′-epimers, 628 mg, 59% yield) was obtained as white solid on elution with hexane/EtOAc from 7:3 to 3:2. Crystallization of part of the above product (100 mg) from EtOAc (0.2 mL)/hexane (0.2 mL) gave a mixture of **24** and **25** in a ratio **24**/**25** = 7:1 (^1^H-NMR, 22 mg). The mother liquors were concentrated in vacuo and crystallized from EtOAc (0.4 mL)/hexane (1.4 mL) gave a mixture of **24** and **25** in a ratio **24**/**25** = 7.5:1 (^1^H-NMR, 15 mg). Recrystallization of the above product (37 mg) from EtOAc (0.4 mL)/hexane (2 mL) gave two crops (16 and 13.5 mg) of pure **24** as white solid. M.p. 159–162 °C (EtOAc/hexane); ^1^H-NMR (400 MHz, CDCl_3_): δ = 0.80 (t, ^2^J_(H,H)_ = ^3^J_(H,H)_ = 12.8 Hz, 1H, 16-H_n_), 1.01 (t, ^2^J_(H,H)_ = ^3^J_(H,H)_ = 12.8 Hz, 18-H_n_), 1.16 (dd, ^2^J_(H,H)_ = 14.4 Hz, ^3^J_(H,H)_ = 6.4 Hz, 1H, C1-CH_a_), 1.57–1.71 (complex signal, 2H, 5′-H_a_ and 17-H), 1.78 (dd, ^2^J_(H,H)_ = 14.0 Hz, ^3^J_(H,H)_ = 6.8 Hz, 1H, C1-CH_b_), 1.81–1.97 (complex signal, 4′-H_a_ and 4′-H_b_), 2.07 (ddd, ^2^J_(H,H)_ = 12.8 Hz, ^3^J_(H,H)_ = 6.0 Hz, ^4^J_(H,H)_ = 1.2 Hz, 16-H_x_), 2.13–2.21 (overlapped dd, ^2^J_(H,H)_ = 19.2 Hz, ^3^J_(H,H)_ = 10.0 Hz, 3′-H_a_), 2.18–2.23 (overlapped dd, ^2^J_(H,H)_ = 12.8 Hz, ^3^J_(H,H)_ = 6.0 Hz, ^4^J(H,H) = 1.6 Hz, 18-H_x_), 2.33 (dddd, ^2^J_(H,H)_ = 19.2 Hz, ^3^J_(H,H)_ = 8.6 Hz, ^3^J_(H,H)_ = 4.8 Hz, ^4^J_(H,H)_ = 1.2 Hz, 1H, 3′-H_b_), 2.42–2.52 (m, 1H, 5′-H_b_), 2.45 (s, 3H, N-CH_3_), 3.62 (3H, COOCH_3_), 3.77 (s, 3H, OCH_3_), 4.35 (s, 1H, 9-H), 5.35 (s, 2H, OCH_2_O), 7.10 (dt, ^4^J_(H,H)_ = 1.6 Hz, ^3^J_(H,H)_ = 7.4 Hz, 1H, 7-H), 7.15 (dt, ^4^J_(H,H)_ = 1.6 Hz, ^3^J_(H,H)_ = 7.4 Hz, 6-H), 7.18–7.20 (m, 8-H), 7.24 (dt, ^4^J_(H,H)_ = 1.6 Hz, ^3^J_(H,H)_ = 7.2 Hz, 12-H), 7.29 (dt, ^4^J_(H,H)_ = 1.6 Hz, ^3^J_(H,H)_ = 7.2 Hz, 13-H), 7.33–7.36 (m, 1H, 11-H), 7.70–7.72 (m, 1H, 14-H), 7.72–7.74 ppm (m, 1H, 5-H); ^13^C-NMR (100.6 MHz, CDCl_3_): δ = 19.4 (CH_2_, C4′), 24.7 (CH_3_, N-CH_3_), 32.6 (CH_2_, C5′), 36.7 (CH, C17), 37.3 (CH_2_, C3′), 38.2 (CH_2_, C1-CH_2_), 38.4 (CH_2_, C16), 38.5 (CH_2_, C18), 48.6 (CH_,_ C9), 52.6 (CH_3_, COOCH_3_), 56.3 (CH_3_, OCH_3_), 60.1 (C, C1), 64.8 (C, C9a), 65.3 (C, C3a), 87.1 (C, C4), 94.1 (CH_2_, OCH_2_O), 122.7 (CH, C5), 124.3 (CH, C14), 124.7 (CH, C8), 126.3 (CH, C11), 126.9 (2 CH, C6 and C13), 127.1 (CH, C7), 127.2 (CH, C12), 138.2 (C, C8a), 138.3 (C, C10), 139.5 (C, C4a), 139.9 (C, C15), 170.5 (C, COOCH_3_), 177.5 (C, C3), 179.6 (C, C1), 214.0 ppm (C, C2′); IR (ATR): ṽ = 2945 (w), 1767 (w), 1746 (w), 1713 (m), 1695 (s), 1452 (m), 1439 (m), 1429 (m), 1377 (m), 1305 (m), 1256 (m), 1238 (m), 1204 (m), 1186 (m), 1165 (m), 1046 (s), 10113 (m), 997 (m), 752 cm^−1^ (m); accurate mass measurement: m/z calcd. for C_32_H_33_NO_7_ + H^+^: 544.2330; found: 544.2329; elemental analysis calcd. (%) for C_32_H_33_NO_7_: C 70.70, H 6.12, N 2.58; found: C 70.38, H 5.95, N 2.39.

Significant ^1^H-NMR data of **25** from the 1:1 mixture **24** and **25** and analytical data from this mixture: ^1^H-NMR (400 MHz, CDCl_3_): δ = 0.79 (t, ^2^*J*_(H,H)_ = ^3^*J*_(H,H_) = 12.8 Hz, 1H, 16-H_n_), 1.03 (t, ^2^*J*_(H,H)_ = ^3^*J*_(H,H)_ = 12.8 Hz, 18-H_n_), 5.34 (d, ^2^*J*_(H,H)_ = 5.2 Hz, 1H, OCH_a_O), 5.37 (d, ^2^*J*_(H,H)_ = 5.2 Hz, 1H, OCH_b_O); accurate mass measurement: *m*/*z* calcd. for C_32_H_33_NO_7_ + H^+^: 544.2330; found: 544.2346; elemental analysis calcd. (%) for C_32_H_33_NO_7_: C 70.70, H 6.12, N 2.58; found: C 70.45, H 6.13, N 2.46.

(3aR*,9aR*,17S*)-17-{[(1′R*)-1-(Methoxycarbonyl)-2-oxocyclopent-3-en-1-yl]methyl}-4-(methoxymethoxy)-2-methyl-4,9-dihydro-4,9[1′,2′]benzeno-3a,9a-propano-1H-benz[f]isoindole-1,3(2H)-dione (**26**). Trimethylsilyl trifluoromethanesulfonate (80 μL, 98% content, 96 mg, 0.43 mmol) was added at once to a cold (0 °C, ice-water bath) solution of keto ester **24** (101 mg, 0.19 mmol) and anhydrous Et_3_N (0.13 mL, 94 mg, 0.93 mmol) in anhydrous CH_2_Cl_2_ (1 mL), under an Ar atmosphere, and the mixture was stirred at room temperature for 30 min. The solution was cooled to 0 °C (ice-water bath), a saturated aqueous solution of NaHCO_3_ (1 mL) was added, the organic phase was separated, and the aqueous one was extracted with CH_2_Cl_2_ (2 × 4 mL). The combined organic phases were dried (anhydrous Na_2_SO_4_) and concentrated in vacuo to give a brown oily residue, of the corresponding trimethylsilyl enol ether (118 mg), which was used as such in the next step. Pd(OAc)_2_ (43.5 mg, 98% content, 0.19 mmol) was added to a solution of the above enol ether (118 mg) in anhydrous DMSO (5 mL) and the mixture was stirred for 16 h at room temperature. The suspension was filtered through a pad of Celite^®^, and the solid was washed with EtOAc (10 mL). The combined filtrate and washings were concentrated in vacuo, the residue was taken in EtOAc (15 mL) and was washed with water (15 mL). The aqueous phase was extracted with EtOAc (2 × 20 mL). The combined organic phase and extracts were washed with brine (2 × 25 mL), dried (anhydrous Na_2_SO_4_) and concentrated in vacuo. The brown oily residue (120 mg) was subjected to column chromatography (10 g, 50–200 μm basic Al_2_O_3_, hexane/EtOAc mixtures). On elution with hexane/EtOAc from 9:1 to 3:2, enone **26** (69 mg, 68% yield from **24**) was obtained as white solid. m.p. 160–162 °C (EtOAc/hexane); ^1^H-NMR (400 MHz, CDCl_3_): δ = 0.86 (t, ^2^*J*_(H,H)_ = ^3^*J*_(H,H)_ = 12.8 Hz, 1H, 16-H_n_), 1.04 (t, ^2^*J*_(H,H)_ = ^3^*J*_(H,H)_ = 12.8 Hz, 1H, 18-H_n_), 1.33 (dd, ^2^*J*_(H,H)_ = 14.4 Hz, ^3^*J*_(H,H)_ = 6.4 Hz, 1H, C1′-CH_a_), 1.55–1.68 (m, 1H, 17-H), 1.84 (dd, ^2^*J*_(H,H)_ = 14.4 Hz, ^3^*J*_(H,H)_ = 6.4 Hz, 1H, C1′-CH_b_), 2.07 (ddd ^2^*J*_(H,H)_ = 12.8 Hz, ^3^*J*_(H,H)_ = 5.8 Hz, ^4^*J*_(H,H)_ = 1.6 Hz, 1H, 16-H_x_), 2.19 (ddd, ^2^*J*_(H,H)_ = 13.2 Hz, ^3^*J*_(H,H)_ = 6.0 Hz, ^4^*J*_(H,H)_ = 1.3 Hz, 1H, 18-H_x_), 2.42 (overlapped dt, ^2^*J*_(H,H)_ = 19.2 Hz, ^3^*J*_(H,H)_ = 2.4 Hz, 1H, 5′-H_a_), 2.44 (overlapped s, 3H, *N*-CH_3_), 3.22 (dt, ^2^*J*_(H,H)_ = 19.2 Hz, ^3^*J*_(H,H)_ = 2.6 Hz, 1H, 5′-H_b_), 3.62 (s, 3H, COOCH_3_), 3.77 (s, 3H, OCH_3_), 4.35 (s, 1H, 9-H), 5.34 (s, 2H, OCH_2_O), 6.09 (dt, ^3^*J*_(H,H)_ = 5.6 Hz, ^3^*J*_(H,H)_ = 2.0 Hz, 1H, 3′-H), 7.11 (dt, ^3^*J*_(H,H)_ = 7.2 Hz, ^4^*J*_(H,H)_ = 1.6 Hz, 1H, 7-H), 7.15 (dt, ^3^*J*_(H,H)_ = 7.2 Hz, ^4^*J*_(H,H)_ = 1.6 Hz, 1H, 6-H), 7.19 (ddm, ^3^*J*_(H,H)_ = 6.8 Hz, ^4^*J*_(H,H)_ = 1.6 Hz, 1H, 8-H), 7.25 (dt, ^3^*J*_(H,H)_ = 7.2 Hz, ^4^*J*_(H,H)_ = 1.6 Hz, 1H, 12-H), 7.28 (dt, ^3^*J*_(H,H)_ = 7.2 Hz, ^4^*J*_(H,H)_ = 1.6 Hz, 1H, 13-H), 7.35 (ddm, ^3^*J*_(H,H)_ = 6.4 Hz, ^4^*J*_(H,H)_ = 1.6 Hz, 1H, 11-H) 7.69–7.74 ppm (complex signal, 3H, 4′-H, 5-H and 14-H); ^13^C-NMR (100.6 MHz, CDCl_3_): δ = 24.7 (CH_3_, *N*-*C*H_3_), 36.8 (CH, C17), 38.3 (CH_2_, C1′-*C*H_2_), 38.6 (CH_2_, C16), 38.7 (CH_2_, C18), 38.9 (CH_2_, C5′), 48.6 (CH, C9), 52.8 (CH_3_, COO*C*H_3_), 56.4 (CH_3_, OCH_3_), 57.6 (C, C1′), 64.7 (C, C9a), 65.3 (C, C3a), 87.2 (C, C4), 94.1 (OCH_2_O), 122.7 (CH, C5), 124.3 (CH, C14), 124.7 (CH, C8), 126.3 (CH, C11), 126.89 (CH) and 126.91 (CH) (C6 and C13), 127.1 (CH, C7), 127.2 (CH, C12), 131.6 (CH, C3′), 138.20 (C) and 138.23 (C) (C8a and C10), 139.5 (C, C4a), 139.8 (C, C15), 163.9 (CH, C4′), 170.3 (*C*OOCH_3_), 177.4 (C, C3), 179.6 (C, C1), 204.8 ppm (C, C2′); IR (ATR): *ṽ* = 2940 (w), 1767 (w), 1741 (m), 1695 (s), 1454 (m), 1423 (m), 1240 (m), 1186 (m), 1168 (m), 1054 (m), 1031 (m), 1013 (m), 984 (s), 760 (s), 749 cm^−1^ (m); accurate mass measurement: *m*/*z* calcd. for C_32_H_31_NO_7_ + Na^+^: 564.1993; found: 564.1993; elemental analysis calcd. (%) for C_32_H_31_NO_7_·0.25H_2_O: C 70.38, H 5.81, N 2.56; found: C 70.28, H 5.73, N 2.39.

*Stereoisomeric mixture of* **26** *and its C1′ epimer* (**27**). Trimethylsilyl trifluoromethanesulfonate (310 μL, 98% content, 372 mg, 1.68 mmol) was added at once to a cold (0 °C, ice-water bath) solution of a stereoisomeric mixture of keto esters **24** and **25** (approximate ratio **24**/**25** = 1:1, 400 mg, 0.74 mmol) and anhydrous Et_3_N (0.51 mL, 370 mg, 3.7 mmol) in anhydrous CH_2_Cl_2_ (3 mL) under an Ar atmosphere, and the mixture was stirred at room temperature for 30 min. The solution was cooled to 0 °C (ice-water bath), a saturated aqueous solution of NaHCO_3_ (3 mL) was added, the organic phase was separated and the aqueous one was extracted with CH_2_Cl_2_ (2 × 6 mL). The combined organic phases were dried (anhydrous Na_2_SO_4_) and concentrated in vacuo to give a brown oily residue, mixture of the corresponding trimethylsilyl enol ethers (412 mg), which was used as such in the next step. Accurate mass measurement: *m*/*z* calcd. for C_35_H_41_NO_7_Si + H^+^: 616.2725; found: 616.2731.

Pd(OAc)_2_ (169 mg, 98% content, 0.74 mmol) was added to a solution of the above stereoisomeric mixture of enol ethers (412 mg) in anhydrous DMSO (15 mL) and the mixture was stirred at room temperature for 16 h. The suspension was filtered through a pad of Celite^®^, and the solid was washed with EtOAc (20 mL). The combined filtrate and washings were concentrated in vacuo, the residue was taken in EtOAc (20 mL) and was washed with water (20 mL). The aqueous phase was extracted with EtOAc (2 × 20 mL). The combined organic phase and extracts were washed with brine (2 × 25 mL), dried (anhydrous Na_2_SO_4_) and concentrated in vacuo to give a brown oily residue (390 mg), which was subjected to column chromatography (10 g, 50–200 μm basic Al_2_O_3_, hexane/EtOAc mixtures). On elution with hexane/EtOAc 3:2, a stereoisomeric mixture of enones **26** and **27** in an approximate ratio **26**/**27** = 1:1 (301 mg, 75% yield from **24** + **25**) was obtained as white solid. m.p. 76–86 °C (EtOAc/hexane); accurate mass measurement: *m*/*z* calcd. for C_32_H_31_NO_7_ + H^+^: 542.2173; found: 542.2179; elemental analysis calcd. (%) for C_32_H_31_NO_7_ 1.5H_2_O: C 67.59, H 6.03, N 2.46; found: C 67.88, H 5.78, N 2.29.

*Methyl 2-methyl-1,3-dioxo-2,3,5,6-tetrahydro-1H,4H-3a,7,8-(epiprop[2]ene[1,1,3]triyl)-5,8a-methanocyclo hepta[c]pyrrole-7(8H)-carboxylate* **13**.

(a) NaBH_4_ (67 mg, 1.72 mmol) was added portionwise to a cold (−40 °C) solution of a diastereomeric mixture of enones **26** and **27** (233 mg, 0.43 mmol) and CeCl_3_·7H_2_O (417 mg, 1.12 mmol) in a mixture of THF (4.5 mL) and MeOH (5 mL) and the mixture was stirred at this temperature for 1 h. A saturated aqueous solution of NaHCO_3_ (2 mL) and water (2 mL) were added and the mixture was extracted with CH_2_Cl_2_ (3 × 15 mL). The combined organic phases were dried (anhydrous Na_2_SO_4_) and concentrated in vacuo to give a complex diastereoisomeric mixture of cyclopentenols **28** (172 mg) as white solid, that was used as such in the next step.
(b) *p*-TsOH H_2_O (16.4 mg, 0.09 mmol) was added to a solution of the above cyclopentenols **28** (172 mg) in benzene (15 mL) and the solution was heated under reflux for 14 h with azeotropic elimination of water with a Dean-Stark equipment. Then, the solution was allowed to cool to room temperature and was treated with solid K_2_CO_3_ (about 100 mg). The suspension was filtered and the filtrate was concentrated in vacuo to give cyclopentadiene alcohol **29** as light brown solid (180 mg) that was used as such in the next step.
(c) A suspension of 30% KH in mineral oil (150 mg, about 1.1 mmol) was placed in a three-necked flask, provided with a low temperature thermometer and an Ar atmosphere. The mineral oil was removed by washing with anhydrous THF (5 × 5 mL) and after the last washing anhydrous THF (12 mL) was added. The mixture was cooled to 0 °C with an ice-water bath and a solution of the above product **29** (180 mg) in anhydrous THF (12 mL) was added dropwise with magnetic stirring and the mixture was stirred at room temperature for 1 h. The excess KH was destroyed by careful addition of 2 N HCl (1 mL) plus 5 N HCl (0.5 mL) under an Ar atmosphere. The solution was dried (anhydrous MgSO_4_, about 3 g), filtered under vacuum and concentrated under reduced pressure to give an oily residue (263 mg) that was subjected to column chromatography (20 g, 35–70 μm silica gel, hexane/EtOAc mixtures). Compound **13** (37 mg, 30% yield from **26** + **27**) was obtained as a light yellow solid, on elution with hexane/EtOAc 4:1. The ^1^H-NMR data of this compound coincide with those previously described.

*7a-(Methoxycarbonyl)-4-(methylcarbamoyl)-3a,4,5,6,7,7a-hexahydro-1H-1,4,6-(epiethane[1,1,2]triyl)indene-9-carboxylic acid* (**30**). KOH (236 mg, 4.2 mmol) was added to a solution of compound **13** (121 mg, 0.42 mmol) in a mixture of THF (2 mL) and MeOH (2 mL) and the mixture was stirred at room temperature for 15 h. Water (8 mL) was added and the mixture was extracted with EtOAc (3 × 10 mL). The aqueous phase was made acidic with 1N HCl (6 mL) and it was extracted with EtOAc (3 × 10 mL). The combined organic phases were washed with water (2 × 10 mL), dried (anhydrous Na_2_SO_4_) and concentrated in vacuo to give amide acid (**30**) (96 mg, 75% yield) as yellow solid. An analytical sample of (**30**) (13 mg) was obtained as yellow solid by crystallization of a sample (19 mg) of the above product in MeOH (0.5 mL). m.p. 193–194 °C (MeOH); ^1^H-NMR (CD_3_OD): δ = 1.81 (d, ^3^*J*_(H,H)_ = 2.8 Hz, 2H, 7-H_a_ and 7-H_b_), 1.90 (dd, ^2^*J*_(H,H)_ = 11.6 Hz, ^4^*J*_(H,H)_ = 2.4 Hz, 1H) and 1.94 (dd, ^2^*J*_(H,H)_ = 11.6 Hz, ^4^*J*_(H,H)_ = 2.4 Hz, 1H) (5-H_anti_ and 8-H_anti_), 2.23 [dd, ^2^*J*_(H,H)_ = 11.6 Hz, ^3^*J*_(H,H)_ = 4.0 Hz, 1H, 5-H_syn_), 2.41 [dd, ^2^*J*_(H,H)_ = 11.6 Hz, ^3^*J*_(H,H)_ = 4.0 Hz, 1H, 8-H_syn_), 2.46–2.49 (m, 1H, 6-H), 2.67 [s, 3H, *N*-CH_3_], 2.96–2.98 [m, 2H, 1-H and 3a-H], 3.58 [s, 3H, OCH_3_], 6.19 (ddd, ^3^*J*_(H,H)_ = 5.6 Hz, ^3^*J*_(H,H)_ = 2.8 Hz, ^4^*J*_(H,H)_ = 1.2 Hz, 1H, 2-H), 6.34 ppm (ddd, ^3^*J*_(H,H)_ = 5.6 Hz, ^3^*J*_(H,H)_ = 2.8 Hz, ^4^*J*_(H,H)_ = 1.2 Hz, 1H, 3-H); ^13^C-NMR (CD_3_OD): δ = 26.7 [CH_3_, *N*-CH_3_], 35.7 (CH, C6), 36.2 (CH_2_, C7), 44.3 (CH_2_, C5), 44.6 (CH_2_, C8), 52.2 (CH_3_, OCH_3_), 59.16 (CH, C3a), 59.20 (CH, C1), 63.4 (C, C9), 65.3 (C, C4), 68.8 (C, C7a), 136.0 (CH, C3), 138.8 (CH, C2), 176.3 (C, CON), 176.5 (C, *C*OOCH_3_), 177.7 (C, COOH); IR (ATR): *ṽ* = 3409 (m), 3200–2500 [broad band, max. at 2981 (w), 2949 (w), 2930 (w), 2847 (w)], 1731 (s), 1709 (s), 1632 (s), 1531 (s), 1418 (m), 1294 (m), 1280 (m), 1258 (s), 1246 (s), 1209 (m), 1188 (s), 1137 (m), 1105 (m), 1034 (m), 988 (m), 829 (m), 734 (m), 707 (s), 619 cm^−1^ (m); accurate mass measurement: *m*/*z* calcd. for C_16_H_19_NO_5_ − H^−^ 304.1190; found: 304.1188; elemental analysis calcd. (%) for C_16_H_19_NO_5_ 0.25H_2_O: C 62.03, H 6.34, N 4.52; found: C 62.07, H 6.27, N 4.60.

*Methyl 1,3-dioxo-5,6-dihydro-1H,3H,4H-3a,7,8-(epiprop[2]ene[1,1,3]triyl)-5,8a-methanocyclohepta[c] furan-7(8H)-carboxylate* (**31**). Solid NaNO_2_ (305 mg, 4.4 mmol) was added portionwise in 2 h to a cold (0 °C, ice-water bath) solution of amide acid **30** (67 mg, 0.22 mmol) in Ac_2_O (2.75 mL) and AcOH (1.4 mL) and the mixture was stirred at room temperature for 16 h. The mixture was cooled to 0 °C (ice-water bath), water (5 mL) was added and the solution was extracted with CH_2_Cl_2_ (3 × 8 mL). The combined organic extracts were washed with 10% aqueous solution of Na_2_CO_3_ (3 × 8 mL) and water (8 mL), dried (anhydrous Na_2_SO_4_) and concentrated in vacuo to give anhydride **31** (63.8 mg) as white solid. Sublimation of the above product (120 °C/0.2–0.5 torr) gave the analytical sample of **31** (41.5 mg, 70% yield) as white solid. m.p. 184–185 °C (EtOAc); ^1^H-NMR (CDCl_3_, 400 MHz): δ = 1.92–1.93 (dm, ^3^*J*_(H,H)_ = 2.4 Hz, 2H, 6-H_2_), 2.11–2.15 [dm, ^2^*J*_(H,H)_ = 12.0 Hz, 2H] and 2.18–2.22 [dm, ^2^*J*_(H,H)_ = 12.0 Hz, 2H] [4(12)-Hsyn and 4(12)-Hanti], 2.71–2.75 [m, 1H, 5-H], 3.34 [t, ^3^*J*_(H,H)_ = ^4^*J*_(H,H)_ = 2.0 Hz, 2H, 8(11)-H], 3.62 [s, 3H, OCH_3_], 6.38 ppm [t, ^3^*J*_(H,H)_ = ^4^*J*_(H,H)_ = 2.0 Hz, 2H, 9(10)-H]; ^13^C-NMR (CDCl_3_, 100.6 MHz): δ = 35.0 (CH_2_, C6), 38.4 (CH, C5), 39.3 [CH_2_, C4(12)], 52.2 (CH_3_, OCH_3_), 56.3 [CH, C8(11)], 61.3 [C, C3a(8a)], 72.7 (C, C7), 136.4 [CH, C9(10)], 171.4 [C, C1(3)], 172.5 ppm (C, *C*OOCH_3_); IR (ATR): *ṽ* = 2950 (w), 2918 (w), 1834 (w), 1770 (s), 1731 (s), 1696 (w), 1284 (m), 1237 (s), 1174 (m), 941 (m), 921 (vs), 899 (s), 750 (m), 741 (m), 717 cm^−1^ (s); accurate mass measurement: *m*/*z* calcd. for C_15_H_14_O_5_ + H^+^ 275.0914; found: 275.0920; elemental analysis calcd. (%) for C_15_H_14_O_5_: C 65.69, H 5.14. Found: C 65.62, H 5.12.

*7a-(Methoxycarbonyl)-3a,4,5,6,7,7a-hexahydro-1H-1,4,6-(epiethane[1,1,2]triyl)indene-4,9-dicarboxylic acid* (**32**). A mixture of anhydride (**31**) (17 mg, 62 μmol) in water (2 mL) was heated under reflux for 16 h. The solution was allowed to cool to room temperature and was concentrated in vacuo to give diacid **32** (18 mg, 99% yield) as white solid. m.p. 174–177 °C (water); ^1^H-NMR (CD_3_OD): δ = 1.80 (d, ^3^*J*_(H,H)_ = 2.8 Hz, 2H, 7-H_2_), 1.92 [d, ^2^*J*_(H,H)_ = 11.2 Hz, 2H, 5(8)-H_anti_], 2.35 [dd, ^2^*J*_(H,H)_ = 11.6 Hz, ^3^*J*_(H,H)_ = 4.0 Hz, 2H, 5(8)-H_syn_], 2.44–2.46 (m, 1H, 6-H), 2.96 [t, ^3^*J*_(H,H)_ = ^4^*J*_(H,H)_ = 2.0 Hz, 2H, 1(3a)-H], 3.57 [s, 3H, OCH_3_], 4.85 (s, 12H, 2 COOH and CD_3_OH), 6.24 ppm [d, ^3^*J*_(H,H)_ = ^4^*J*_(H,H)_ = 2.0 Hz, 2H, 2(3)-H]; ^13^C-NMR (CD_3_OD): δ = 35.6 (CH, C6), 36.2 (CH_2_, C7), 44.1 [CH_2_, C5(8)], 52.2 (CH_3_, OCH_3_), 59.3 [CH, C1(3a)], 63.8 [C, C4(9)], 68.8 (C, C7a), 137.4 [CH, C2(3)], 176.6 (C, COOH), 177.2 ppm (C, *C*OOCH_3_); IR (ATR): *ṽ* = 3200–2800 [broad band, max. at 2977 (w)], 1725 (m), 1688 (s), 1407 (m), 1303 (m), 1272 (m), 1253 (s), 1243 (s), 1190 (m), 1102 (m), 1017 (m), 935 cm^−1^ (m); accurate mass measurement: *m*/*z* calcd. for C_15_H_16_O_6_ − H^−^ 291.0874; found: 291.0878; elemental analysis calcd. (%) for C_15_H_16_O_6_: C 61.64, H 5.52.

*X-ray Crystal-structure determination of compound* **24**. A colorless prism-like specimen of C_32_H_33_NO_7_, approximate dimensions 0.050 mm × 0.080 mm × 0.467 mm, was used for the X-ray crystallographic analysis. The X-ray intensity data were measured on a D8 Venture system equipped with a multilayer monochromator and a Mo microfocus (λ = 0.71073 Å). The frames were integrated with the Bruker SAINT software package [14,15] using a narrow-frame algorithm. The integration of the data using an orthorhombic unit cell yielded a total of 20021 reflections to a maximum θ angle of 26.41° (0.80 Å resolution), of which 5444 were independent (average redundancy 3.678, completeness = 99.7%, R_int_ = 11.85%, R_sig_ = 12.14%) and 3454 (63.45%) were greater than 2σ(F^2^). The final cell constants of *a* = 8.2651(3) Å, *b* = 11.0782(6) Å, *c* = 29.0497(15) Å, volume = 2659.9(2) Å^3^, are based upon the refinement of the XYZ-centroids of reflections above 20 σ(I). Data were corrected for absorption effects using the multi-scan method (SADABS) [16]. The calculated minimum and maximum transmission coefficients (based on crystal size) are 0.6511 and 0.7454. The structure was solved and refined using the Bruker SHELXTL software package [17], using the space group P 21 21 21, with Z = 4 for the formula unit, C_32_H_33_NO_7_. The final anisotropic full-matrix least-squares refinement on F^2^ with 364 variables converged at *R*_1_ = 5.60%, for the observed data and *wR*_2_ = 10.62% for all data. The goodness-of-fit was 1.016. The largest peak in the final difference electron density synthesis was 0.270 e Å^−3^ and the largest hole was −0.244 e Å^−3^ with an RMS deviation of 0.060 e Å^−3^. On the basis of the final model, the calculated density was 1.357 g cm^−3^ and F(000), 1152 e.

## 4. Conclusions

An alternative synthesis of polycycle **13** has been developed by using methoxymethyl, instead of benzyloxymethyl, as a hydroxyl-protecting group. The overall yield of both synthetic sequences is of the same order. The advantage of the new synthetic sequence derives from the fact that alcohols **19** and **20** could be separated by silica gel column chromatography and the rest of the synthesis could be carried out with the main *syn*-stereoisomer **19**. One of the diastereomers (**24**) formed in the reaction of iodide **23** with methyl 2-oxocyclopentanecarboxylate could be isolated and its structure established by X-ray diffraction analysis. Additionally, the selective hydrolysis of the imide function of **13** to the corresponding diacid has been achieved through a non-standard procedure, whose key-step consists of the conversion of amide acid **30** to anhydride **31** on reaction with NaNO_2_ in a 1:1 mixture of AcOH/Ac_2_O. We hope these products may serve as new scaffolds for the synthesis of potentially active compounds.

## Supplementary Materials

Supplementary materials are available online. IR and NMR spectra of all new compounds.
