# Supplementary material for: Alternative Access to Functionalized 2,8-Ethanonoradamantane Derivatives
Source: Molecules. 2017 May 31;22(6):906. doi: 10.3390/molecules22060906 (PMC6152640; doi:10.3390/molecules22060906)

Alternative access to functionalized 2,8-ethanonoradamantane derivatives

Pelayo Camps [1, *], Tània Gómez [2], Ane Otermin [2] and Mercè Font-Bardia [2,3]

[1] Laboratori de Química Farmacèutica (Unitat Associada al CSIC), Facultat de Farmàcia i Ciències de la Alimentació and Institut de Biomedicina (IBUB), Universitat de Barcelona, Av. Joan XXIII 27–31, Barcelona 08028, Spain; tania_gomeznadal@hotmail.com (T.G.); aneotermin@gmail.com (A. O.)

[2] Departament de Mineralogia, Petrologia i Geologia Aplicada, Universitat de Barcelona, Martí Franquès s/n, Barcelona 08028, Spain

[3] Unitat de Difracció de RX, Centres Científics i Tecnològics de la Universitat de Barcelona (CCiTUB), Solé i Sabarís 1–3, Barcelona 08028, Spain; mercef@ccit.ub.edu

* Correspondence: camps@ub.edu; Tel.: +34-934-024-536

SUPPORTING INFORMATION

Content

- NMR and IR spectra of compound **16** 1
- NMR and IR spectra of compound **17** 6
- NMR and IR spectra of compound **18** 11
- NMR and IR spectra of compound **19** 16
- NMR and IR spectra of compound **20** 21
- NMR and IR spectra of compound **22** 26
- NMR and IR spectra of compound **21** 31
- NMR and IR spectra of compound **23** 36
- NMR and IR spectra of compound **24** and **25** 41
- NMR and IR spectra of compound **24** 46
- NMR and IR spectra of compound **26** 51
- NMR and IR spectra of compound **30** 56
- NMR and IR spectra of compound **31** 61
- NMR and IR spectra of compound **32** 66

NMR and IR spectra of compound **16**

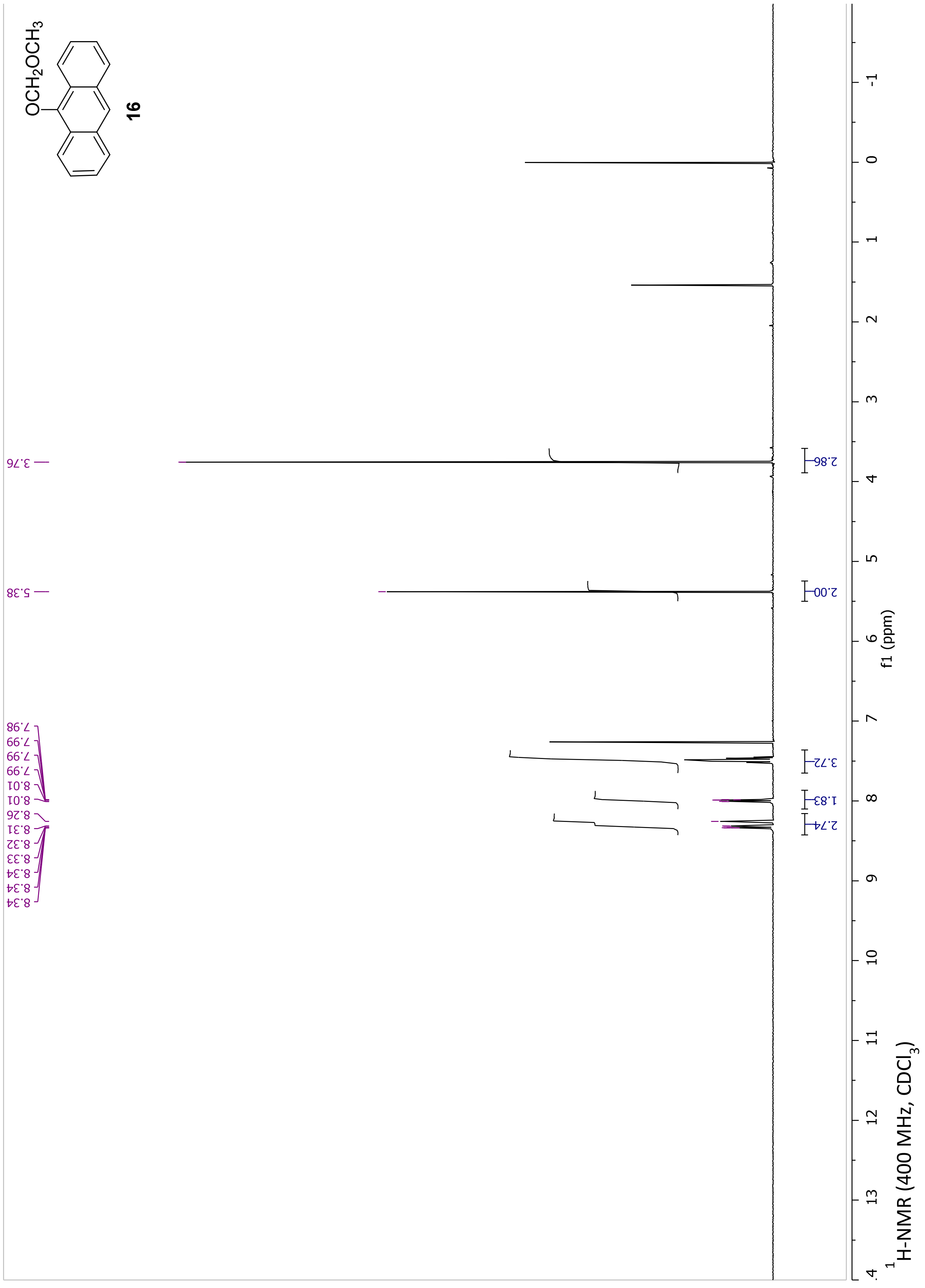

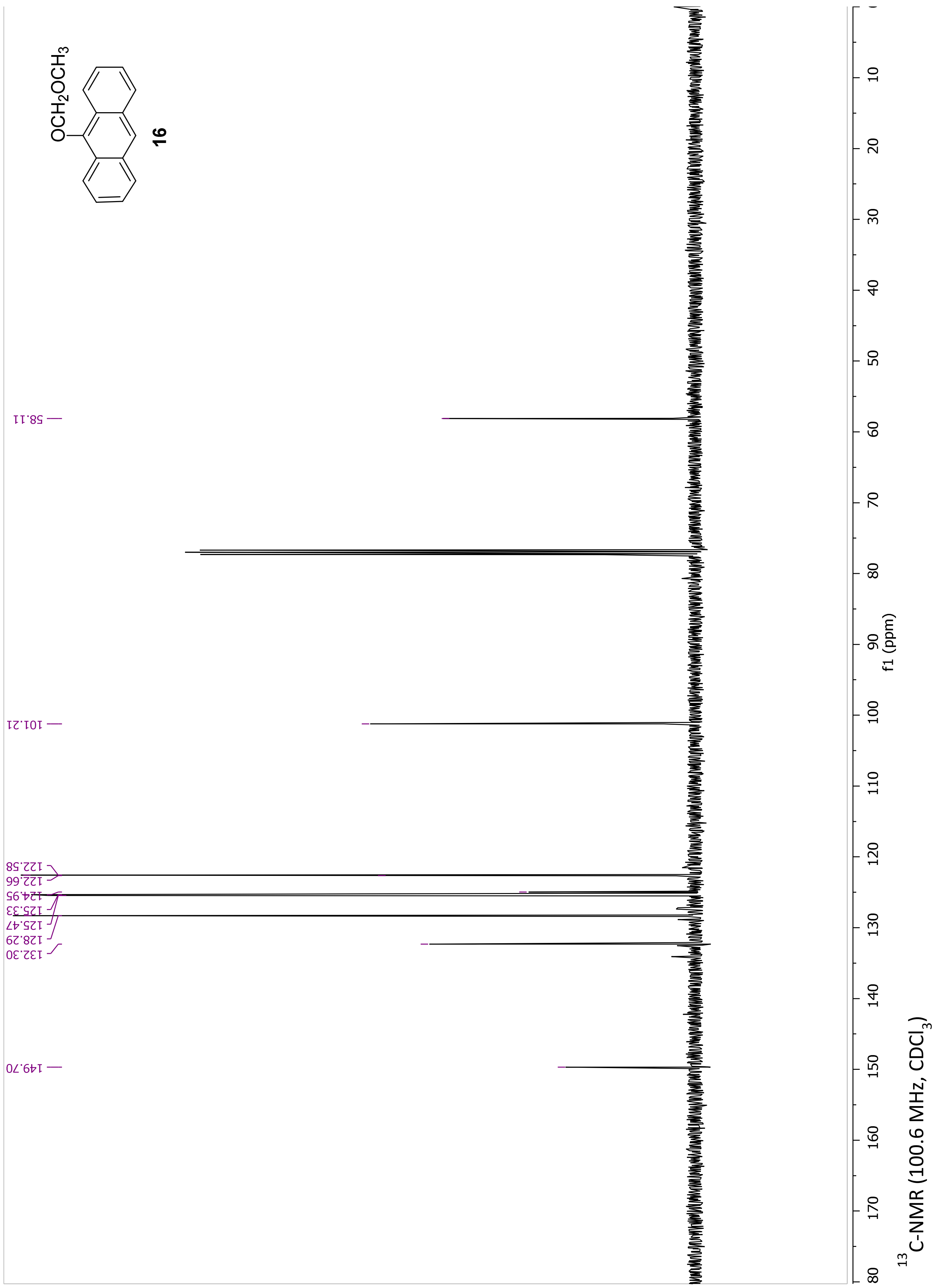
OCH2OCH3
16
58.11
101.21
122.58
122.66
124.95
125.33
125.47
128.29
132.30
149.70
f1 (ppm)
13C-NMR (100.6 MHz, CDCl3)

NMR and IR spectra of compound **16**

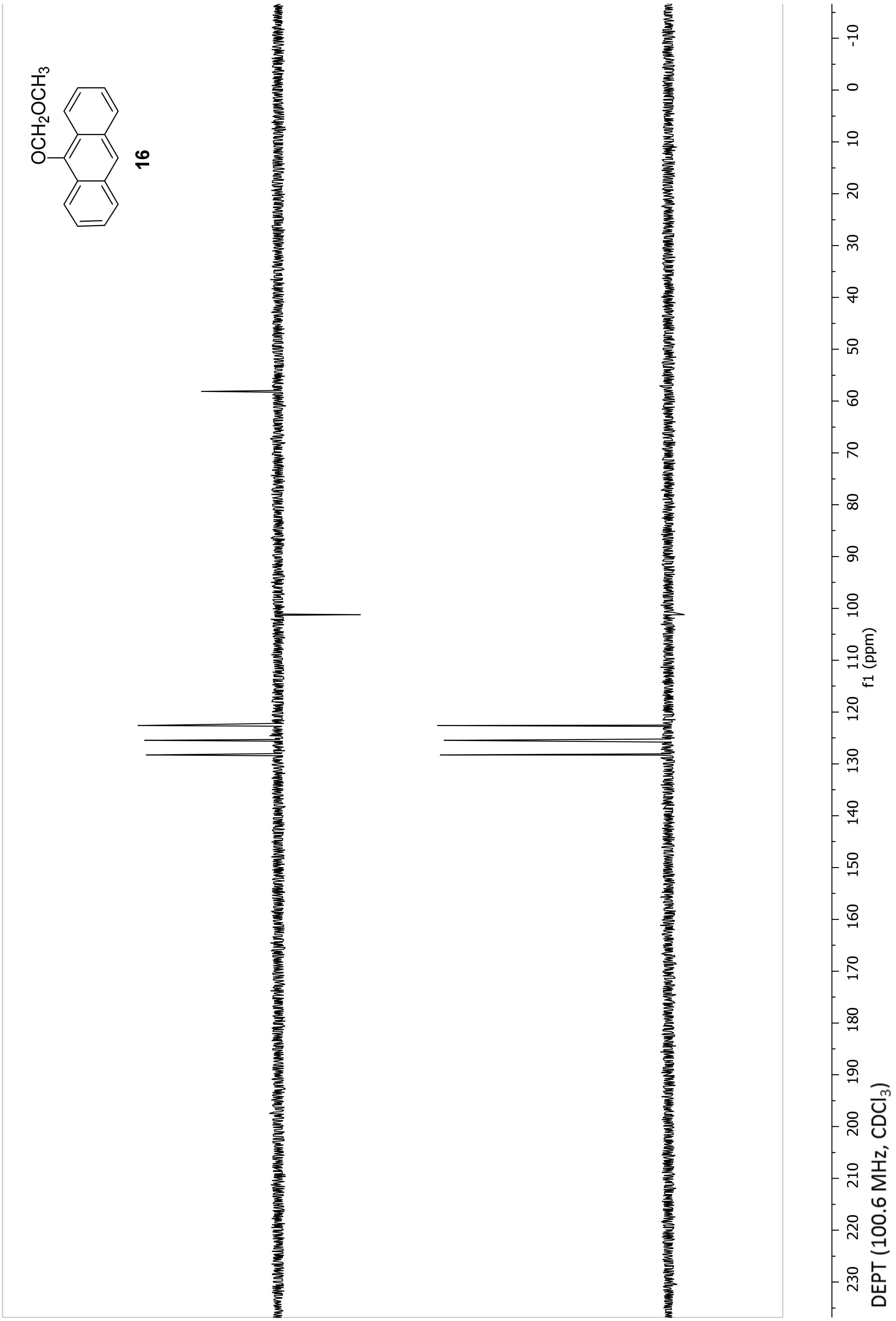

NMR and IR spectra of compound **16**

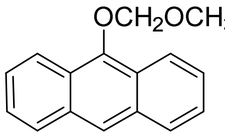

16

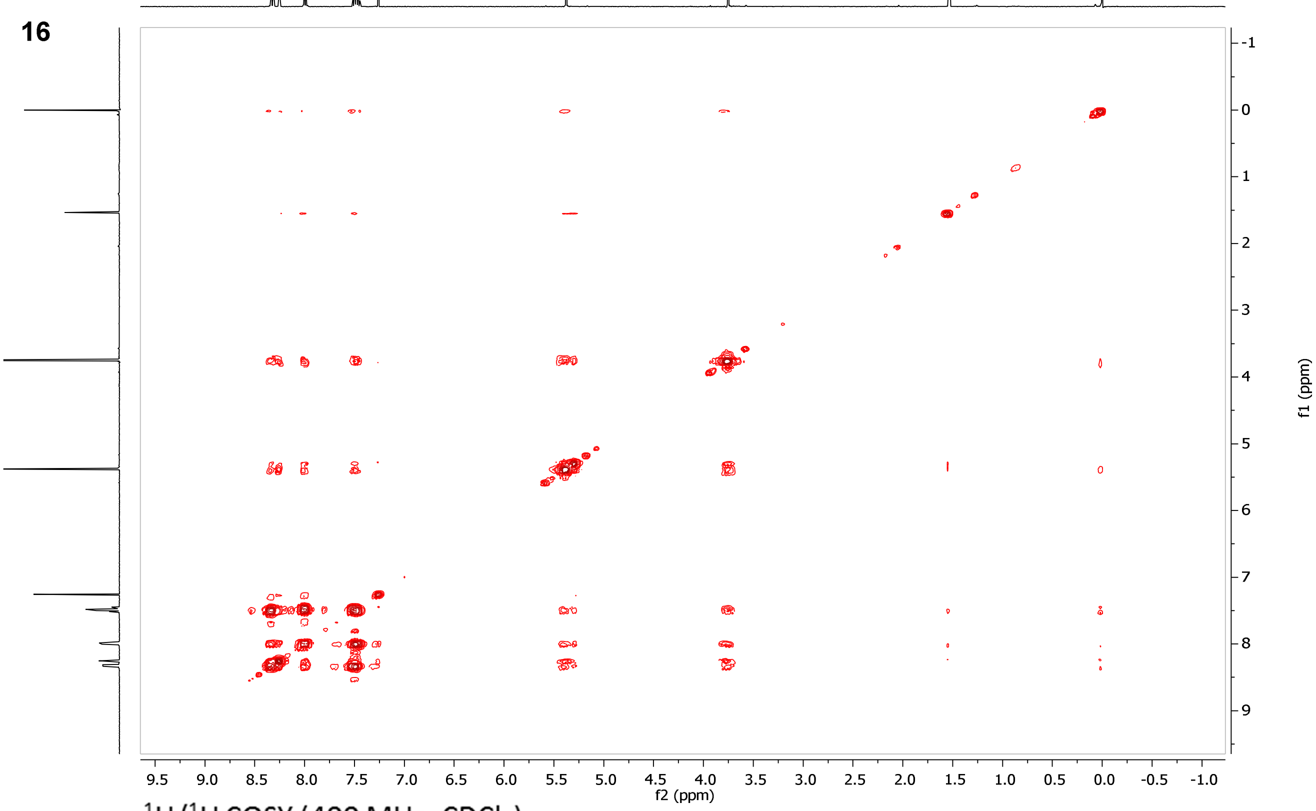

^{1}H/^{1}H COSY (400 MHz, $CDCl_3$)

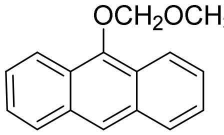

16

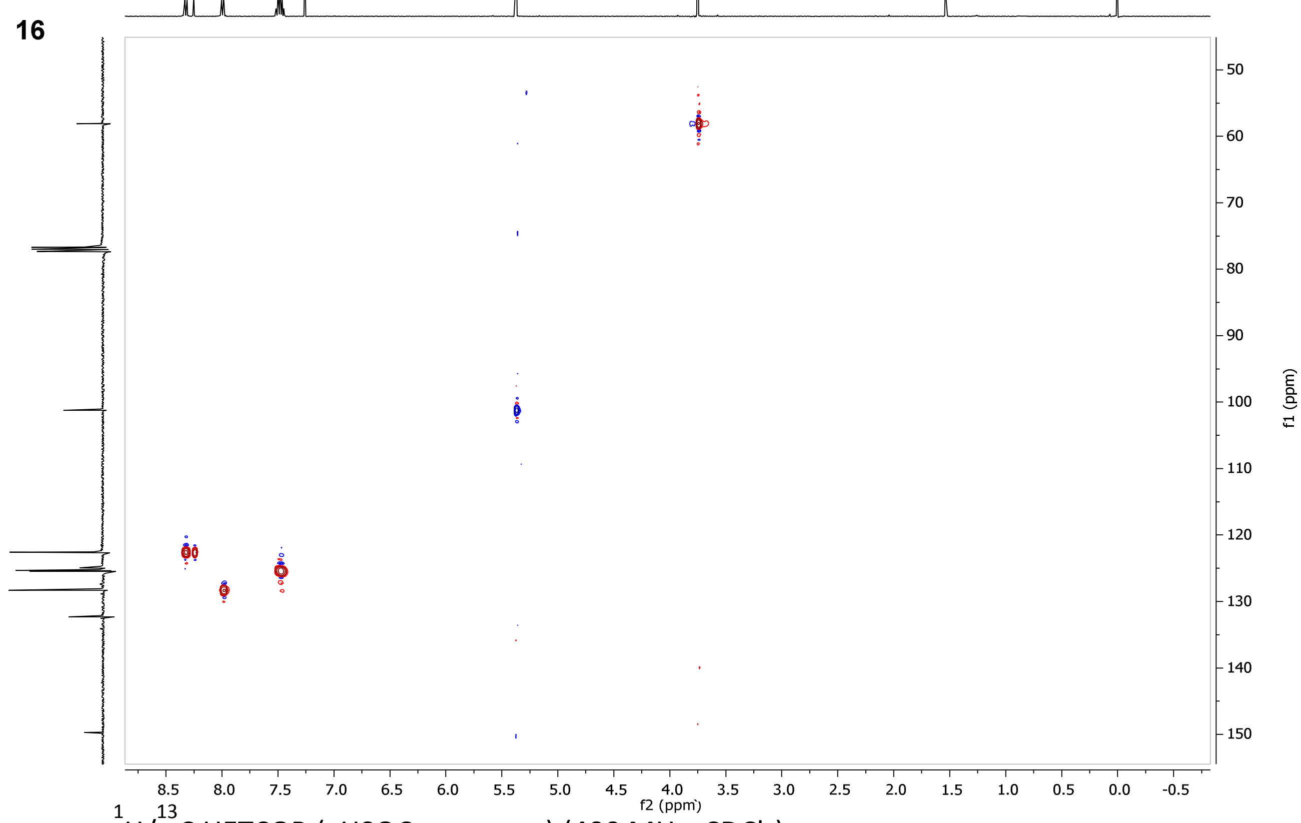

^{1}H/^{13}C HETCOR (gHSQC sequence) (400 MHz, $CDCl_3$)

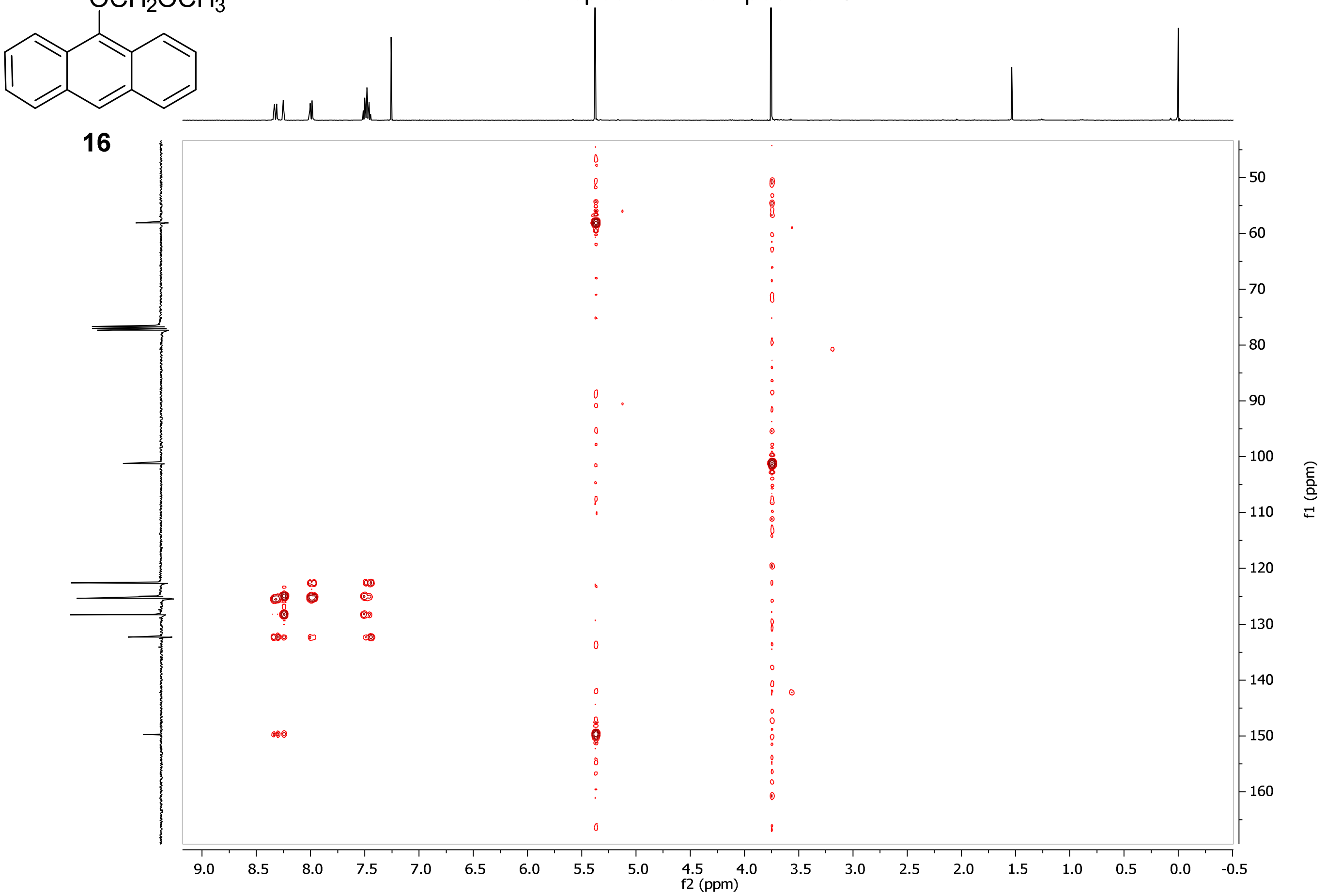

NMR and IR spectra of compound **16**

$^{1}H/^{13}C$ HETCOR (gHMBC sequence) (400 MHz, $CDCl_3$)

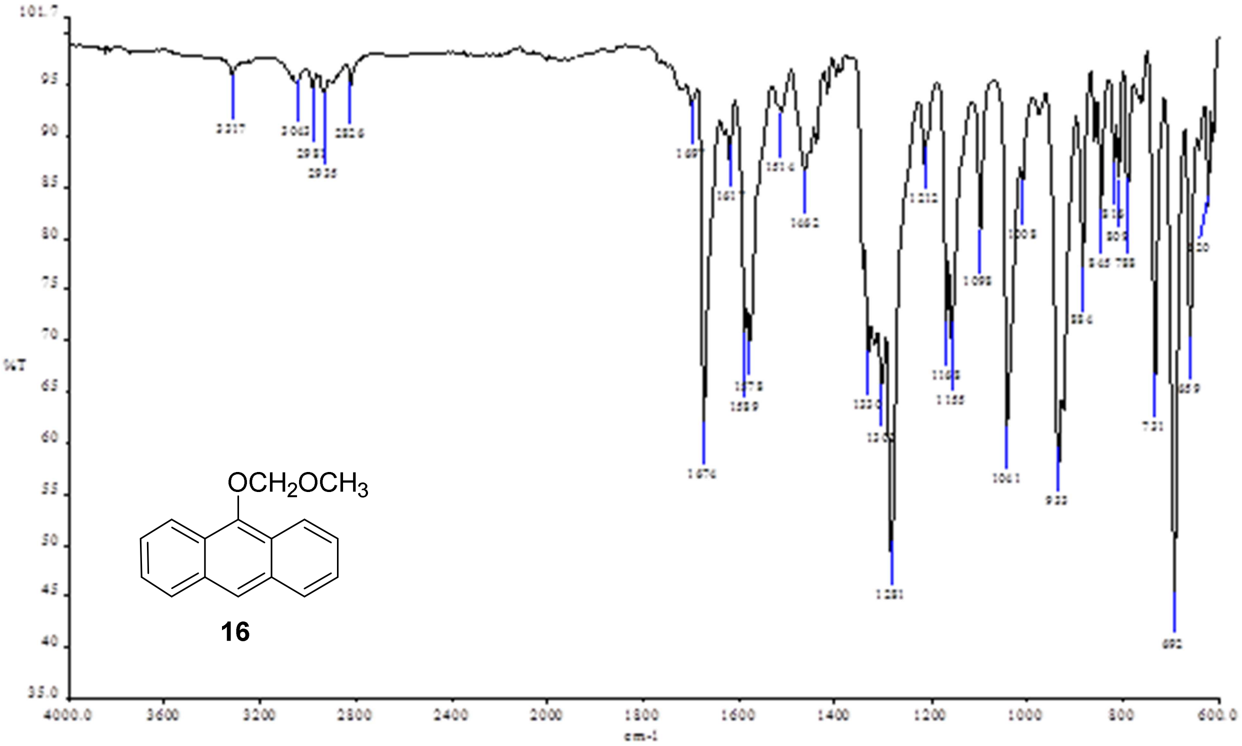

IR (ATR)

NMR and IR spectra of compound **17**

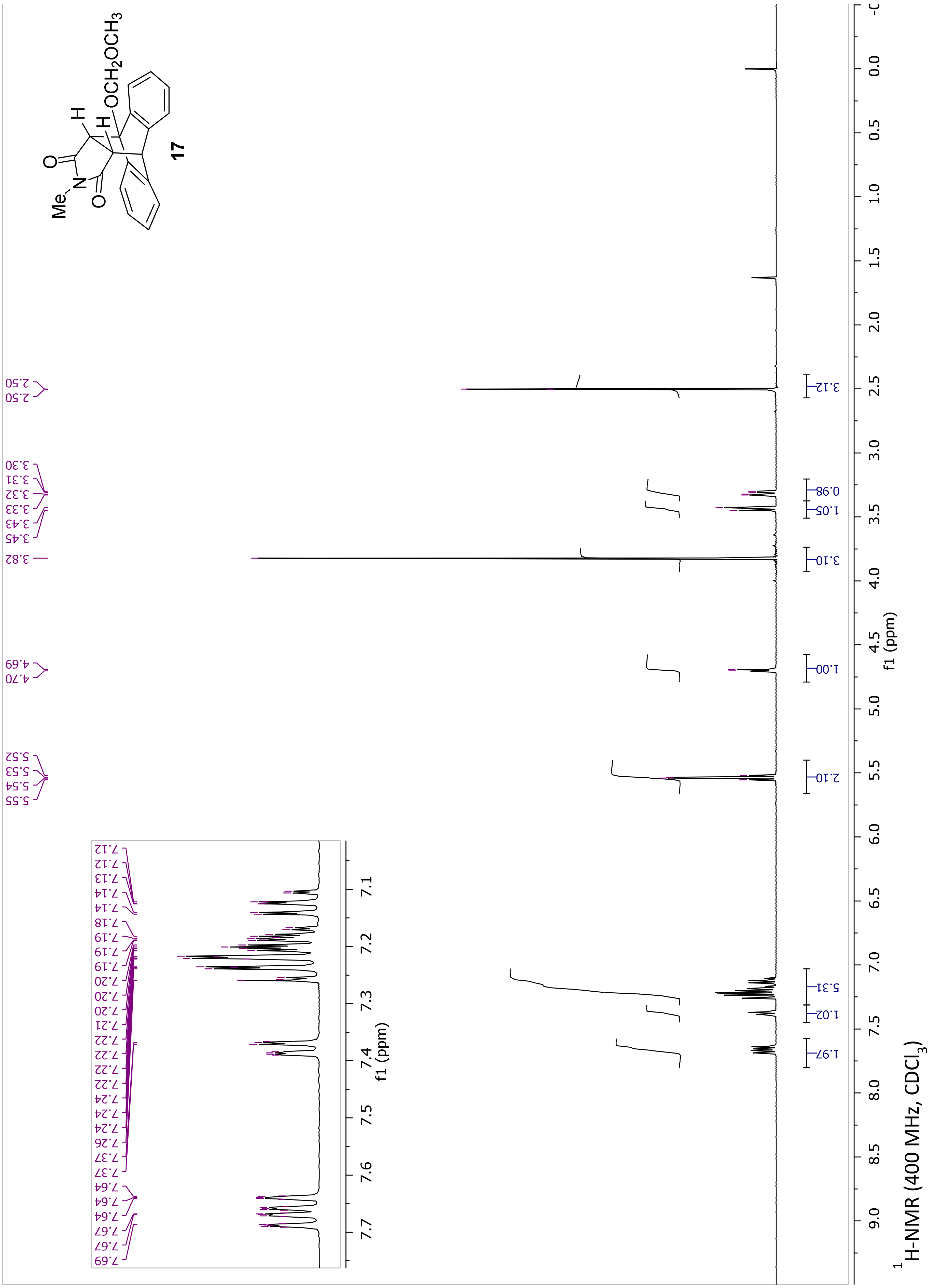

NMR and IR spectra of compound **17**

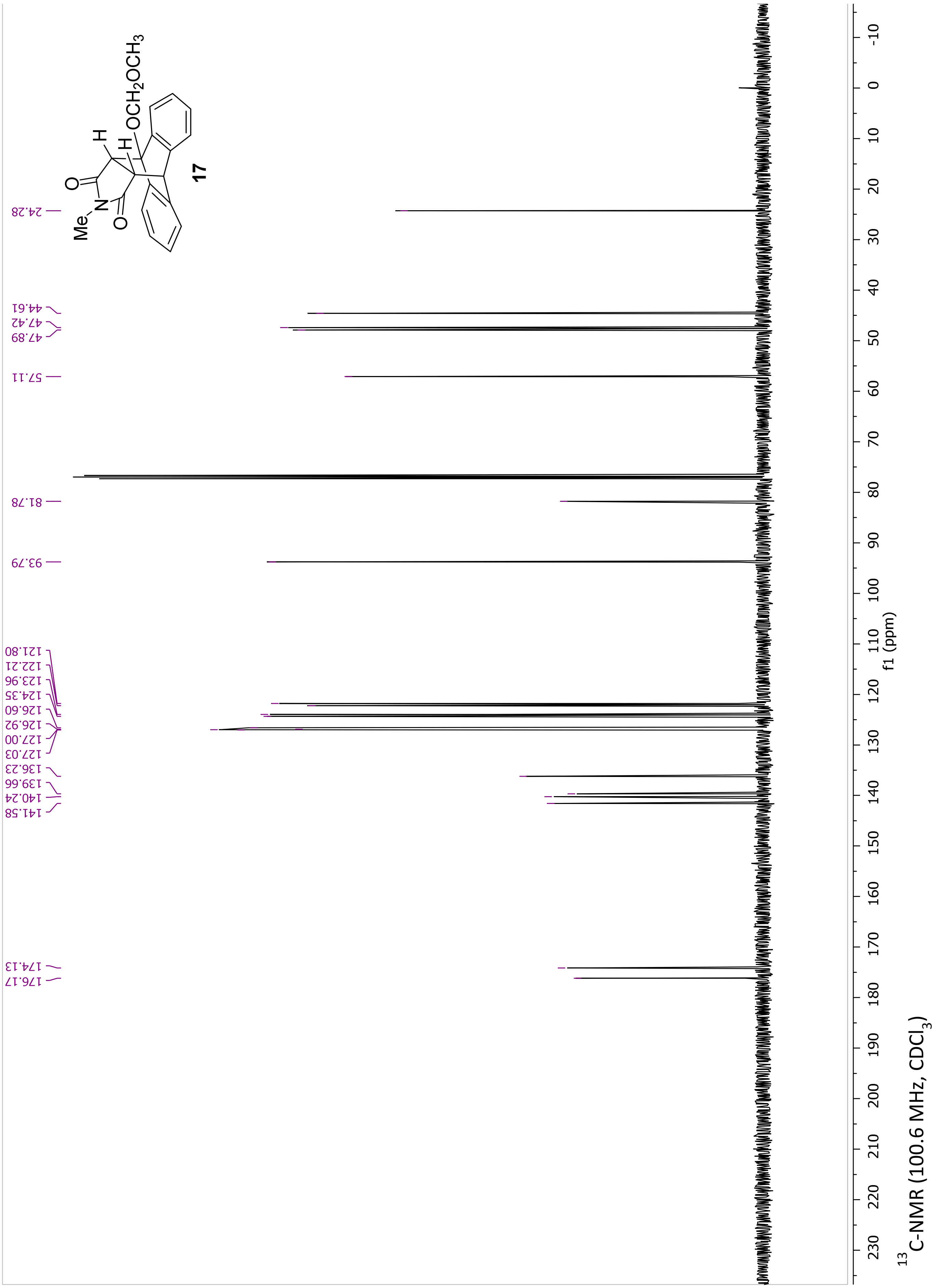

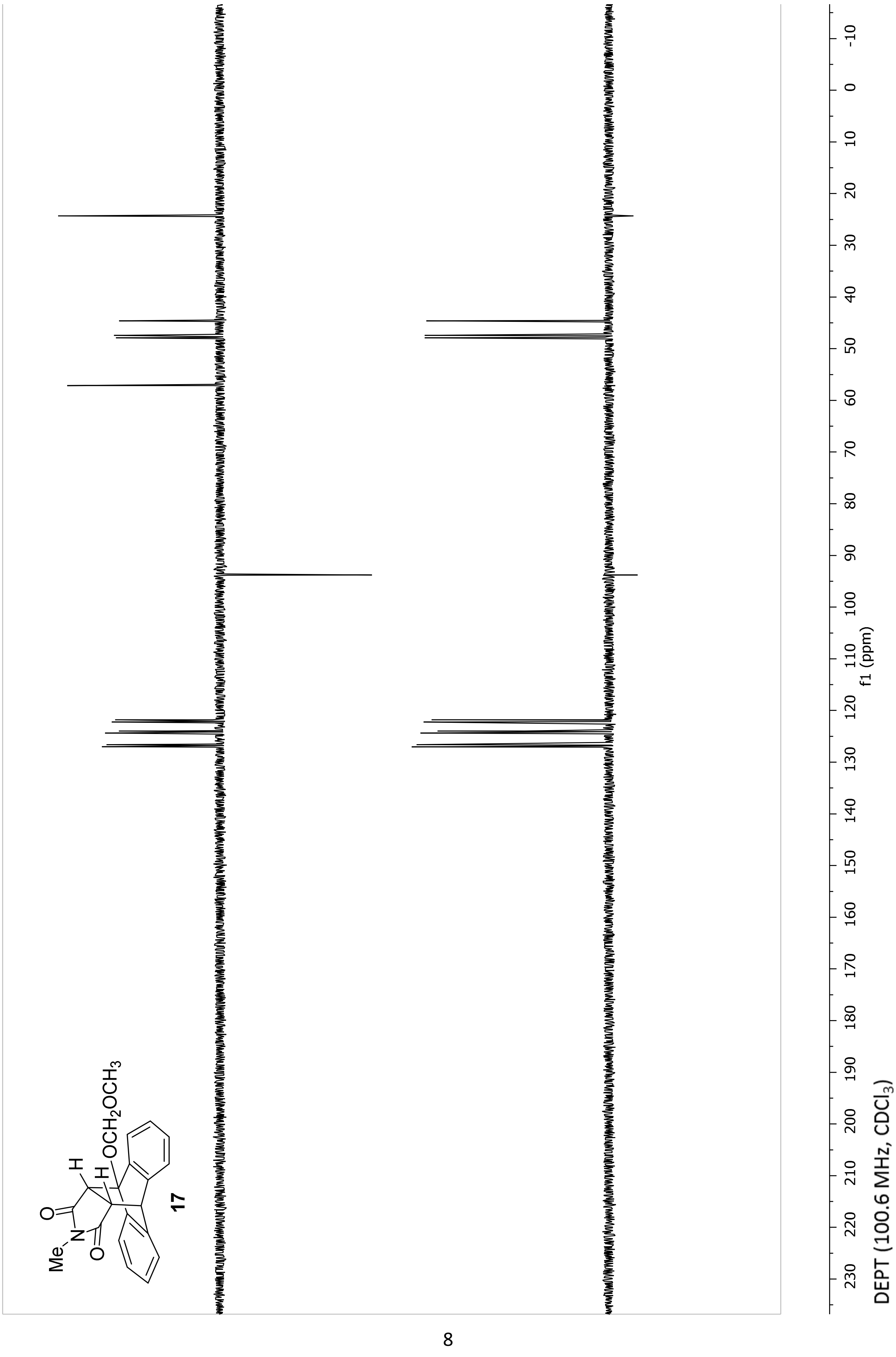

DEPT (100.6 MHz, $CDCl_3$)

NMR and IR spectra of compound **17**

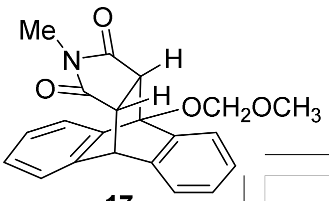

17

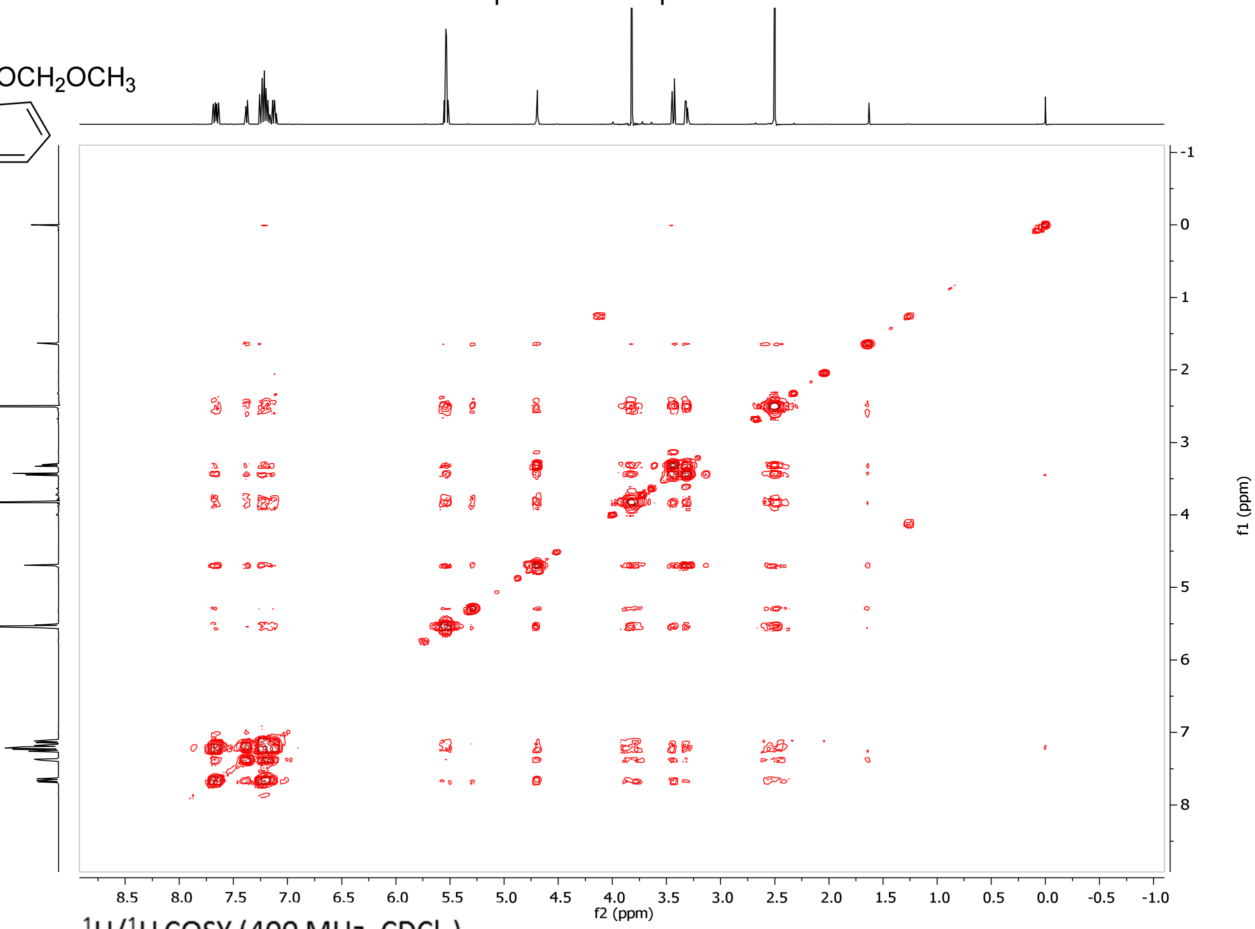

$^{1}H/^{1}H$ COSY (400 MHz, $CDCl_3$)

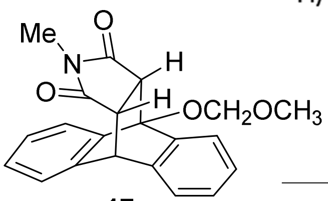

17

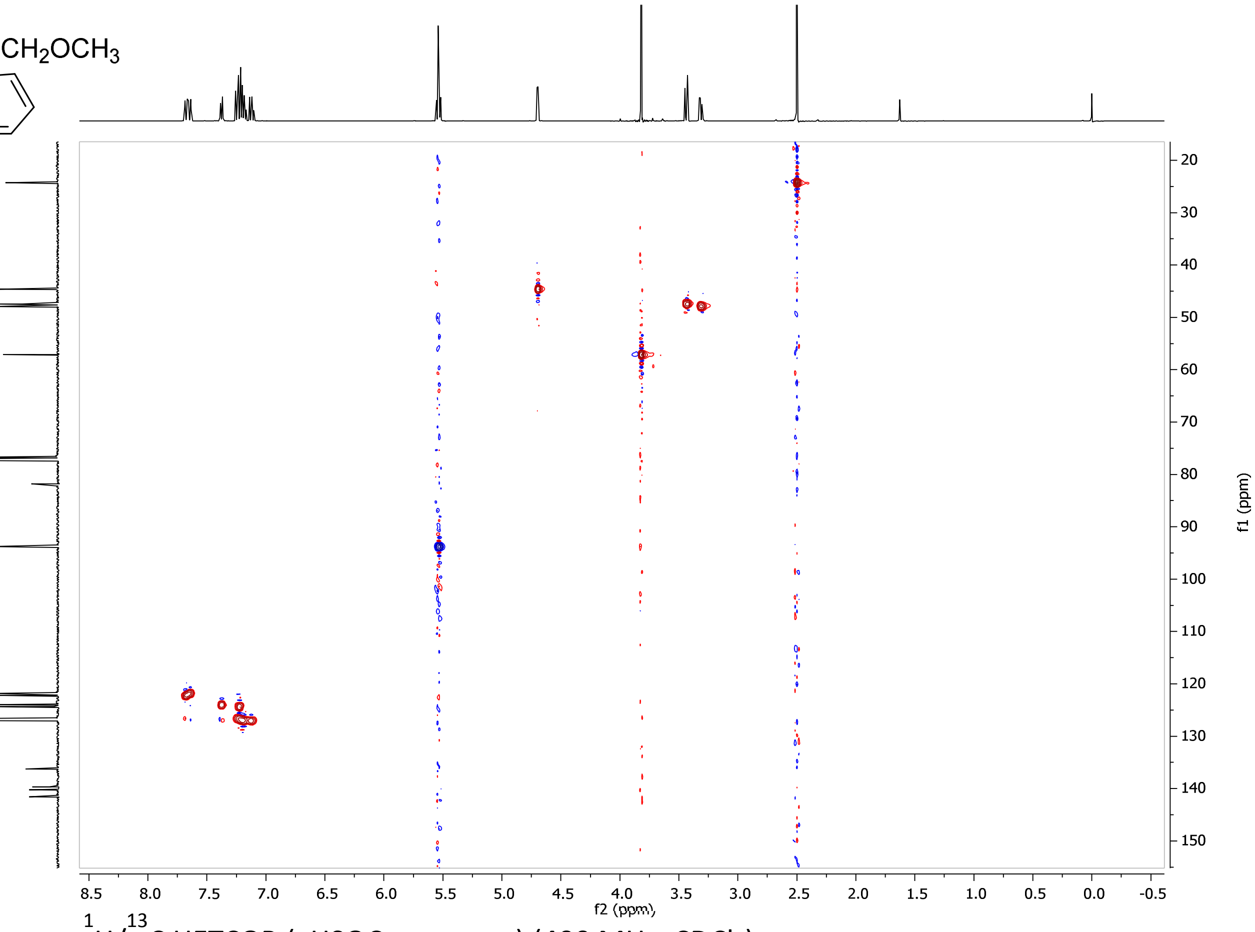

$^{1}H/^{13}C$ HETCOR (gHSQC sequence) (400 MHz, $CDCl_3$)

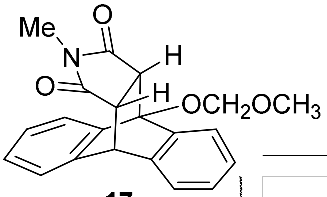

17

NMR and IR spectra of compound **17**

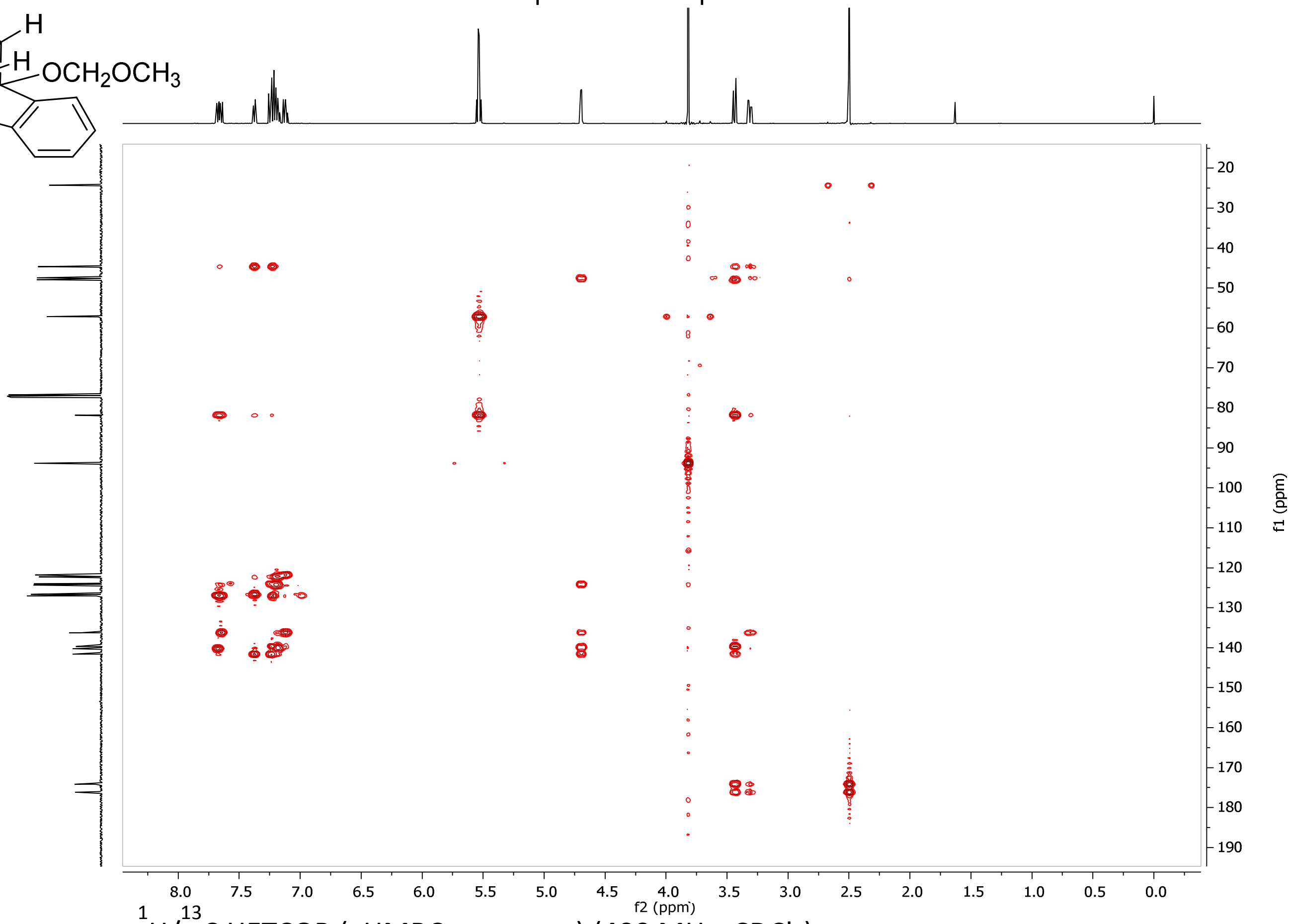

$^{1}H/^{13}C$ HETCOR (gHMBC sequence) (400 MHz, $CDCl_3$)

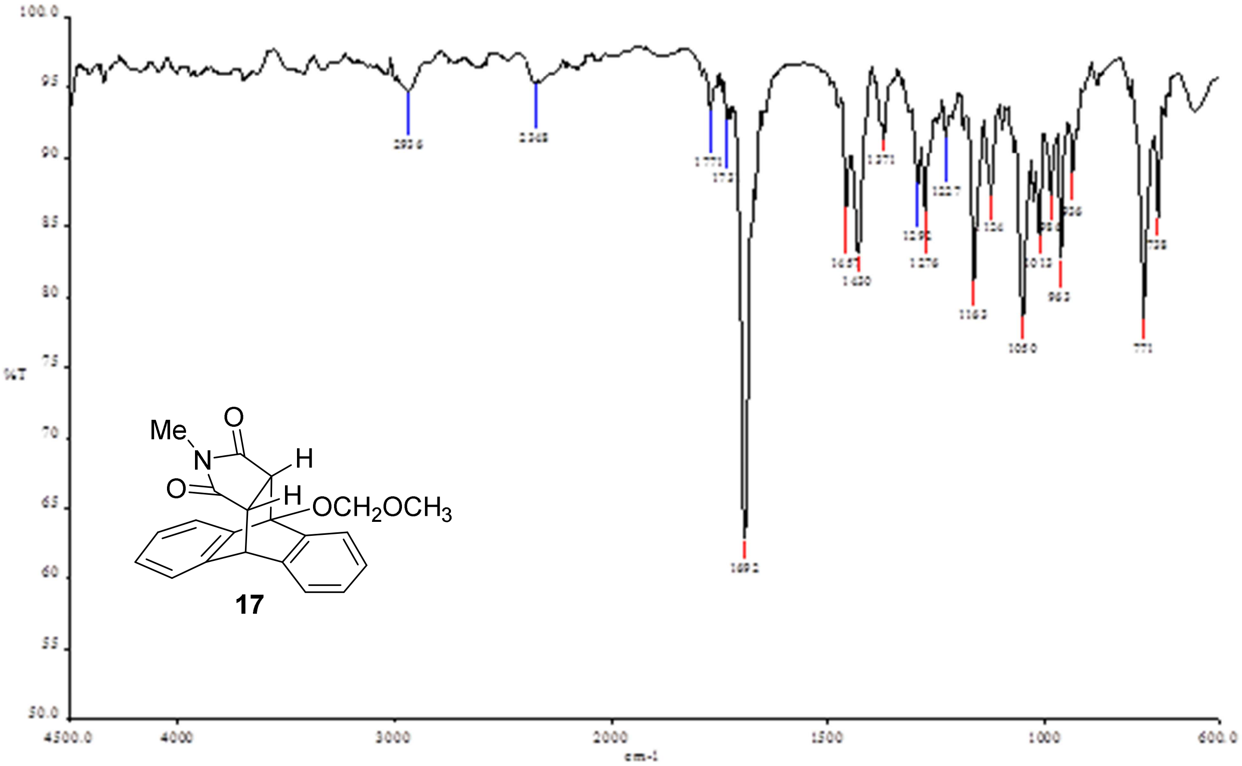

IR (AATR)

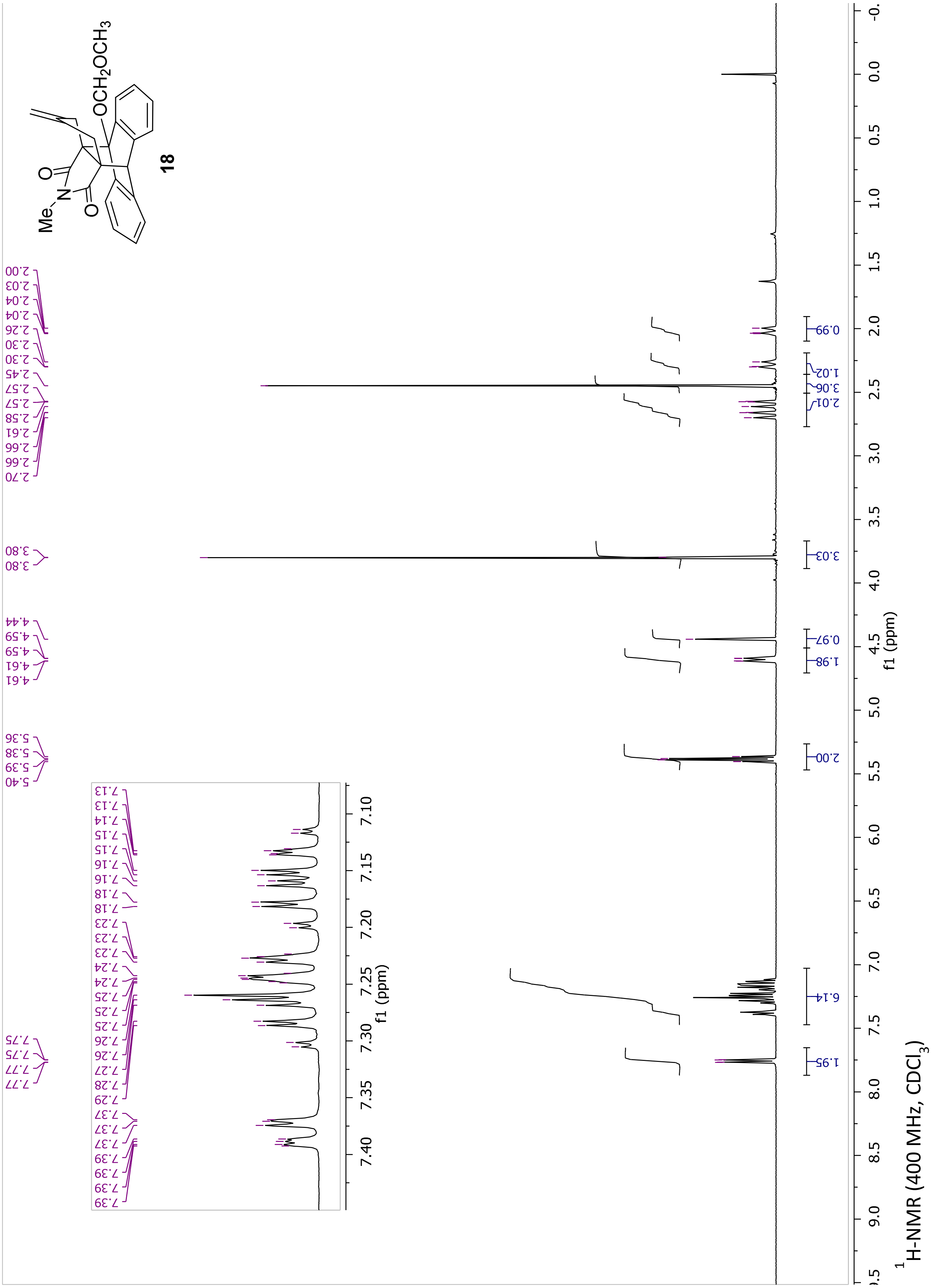
18
f1 (ppm)
1H-NMR (400 MHz, CDCl3)

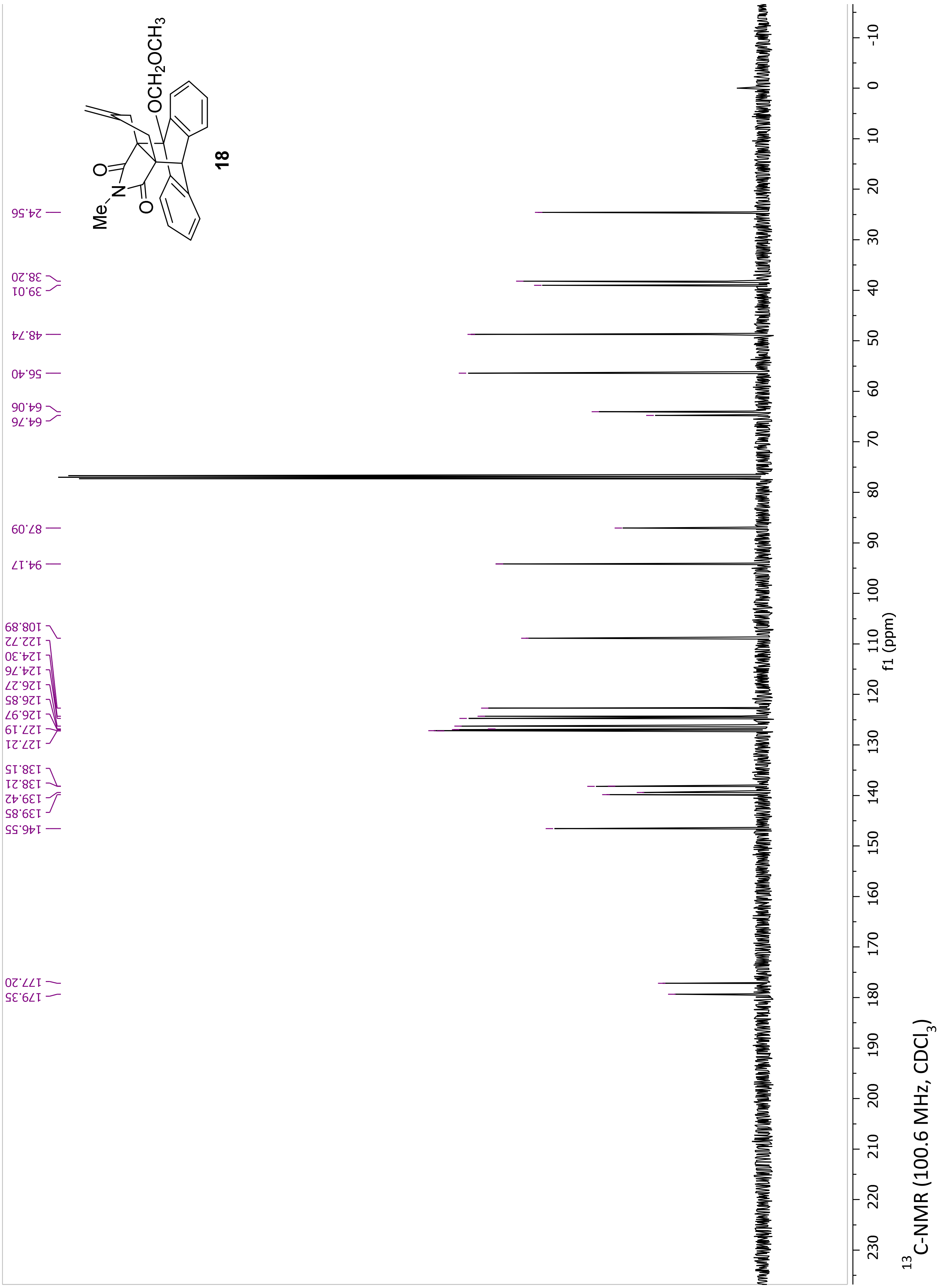
24.56
38.20
39.01
48.74
56.40
64.06
64.76
87.09
94.17
108.89
122.72
124.30
124.76
126.27
126.85
126.97
127.19
127.21
138.15
138.21
139.42
139.85
146.55
177.20
179.35
OCH2OCH3
Me
N
O
18
f1 (ppm)
13C-NMR (100.6 MHz, CDCl3)

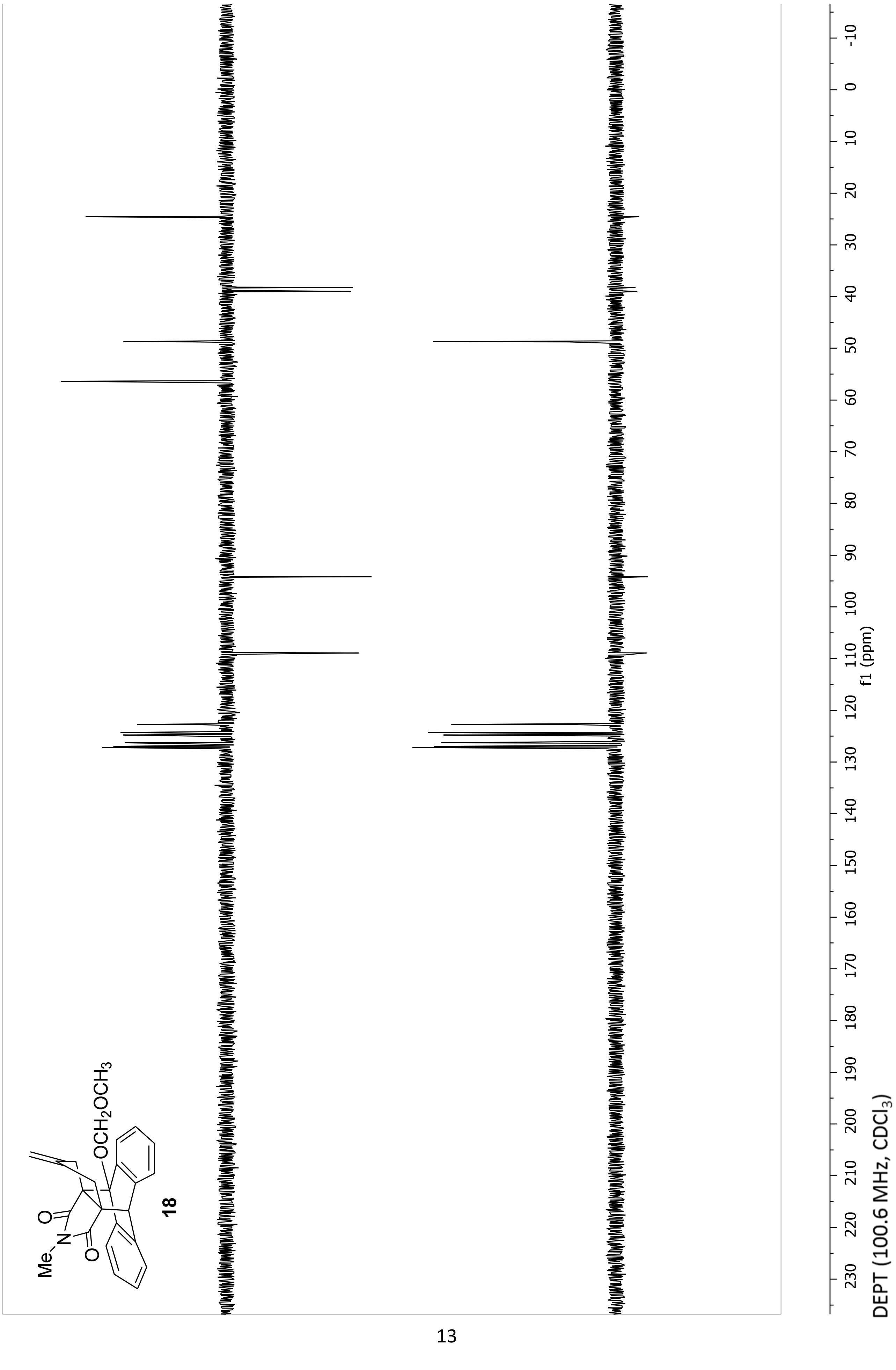
OCH2OCH3
Me
N
O
O
18
f1 (ppm)
DEPT (100.6 MHz, CDCl3)

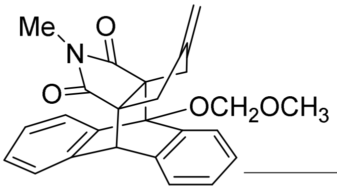

18

NRM and IR spectra of compound **18**

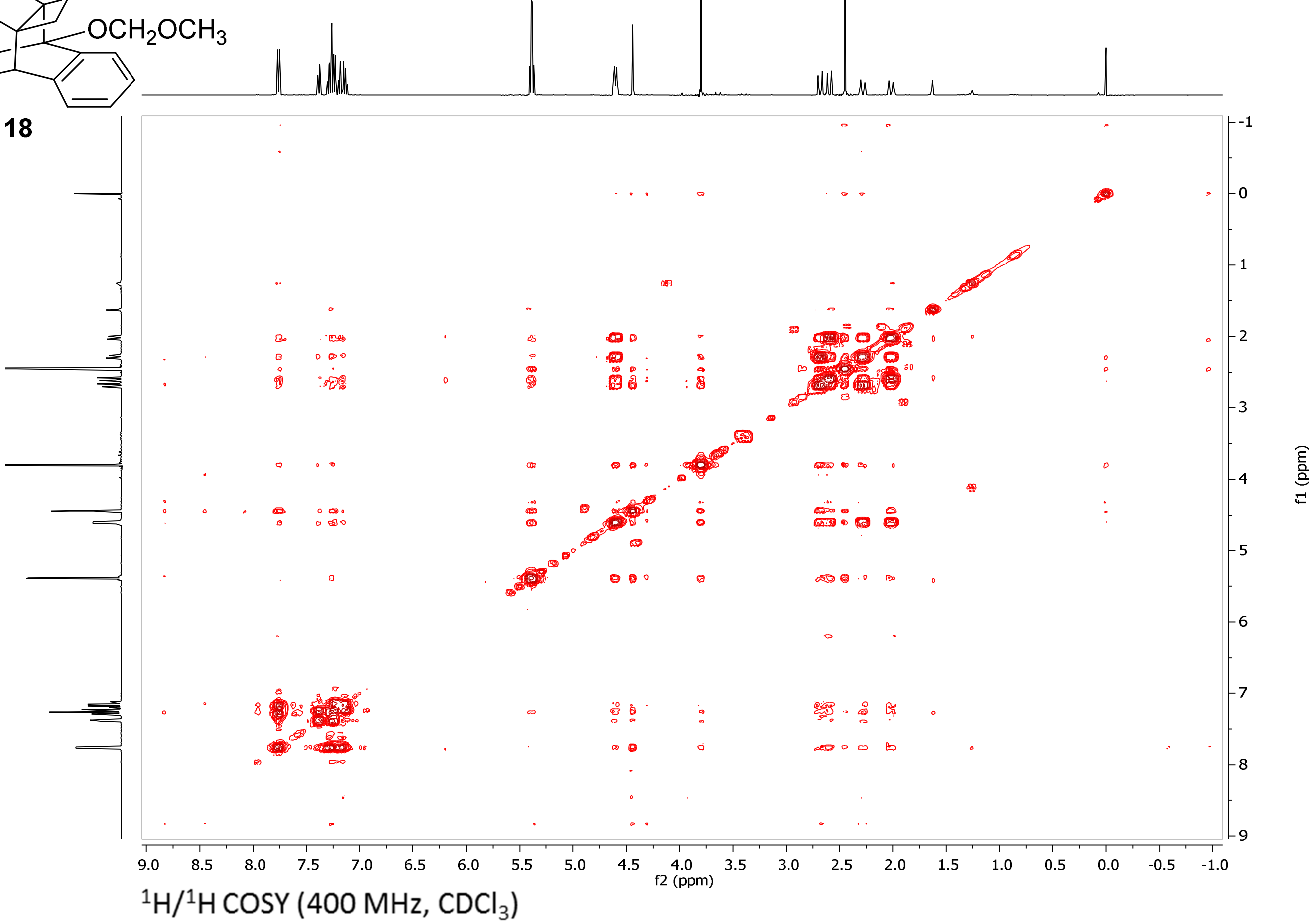

$^1H/^1H$ COSY (400 MHz, $CDCl_3$)

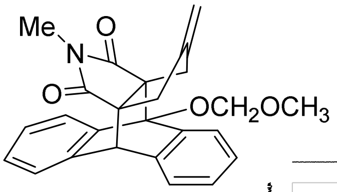

18

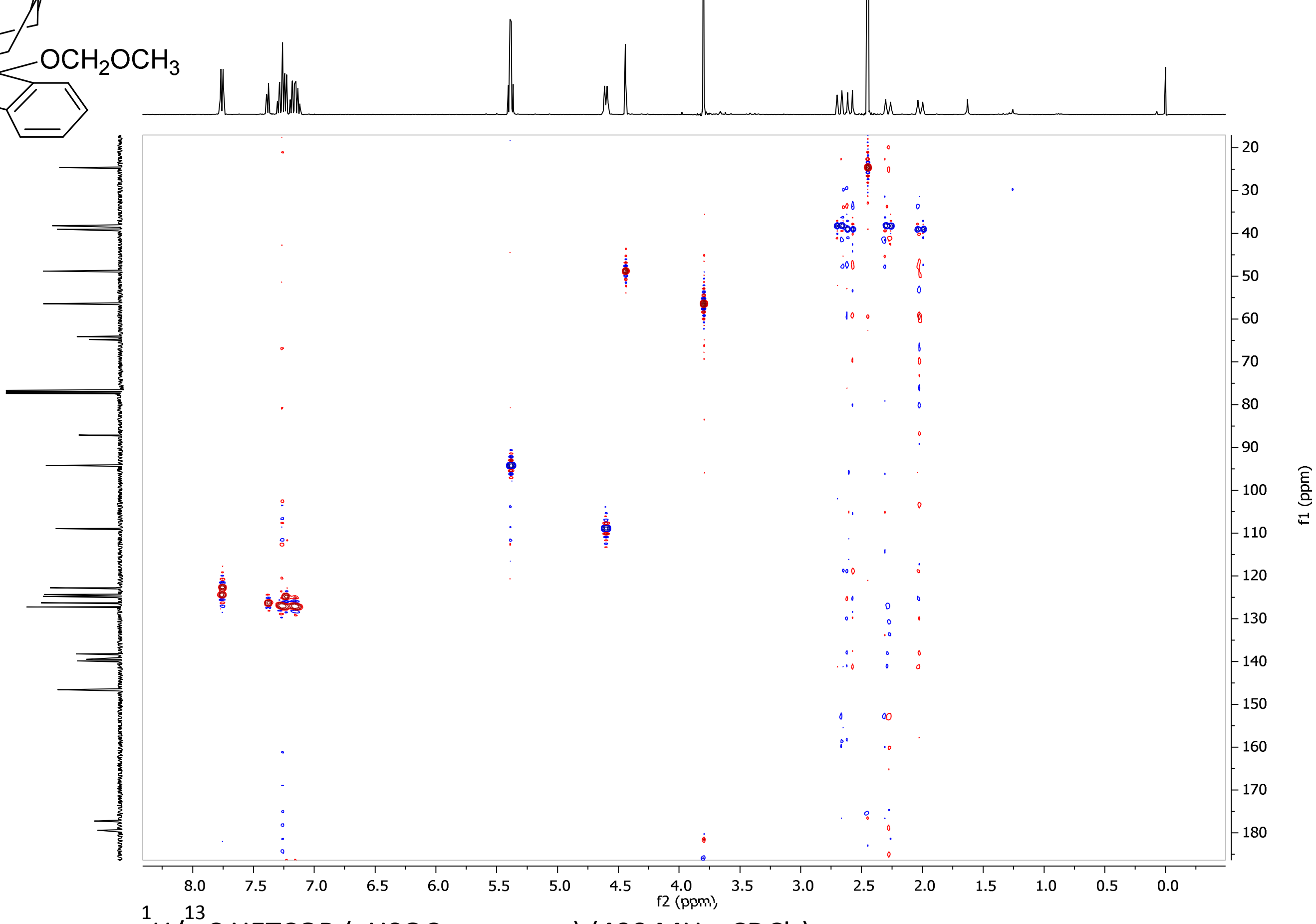

$^1H/^{13}C$ HETCOR (gHSQC sequence) (400 MHz, $CDCl_3$)

NRM and IR spectra of compound **18**

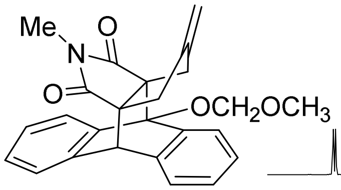

18

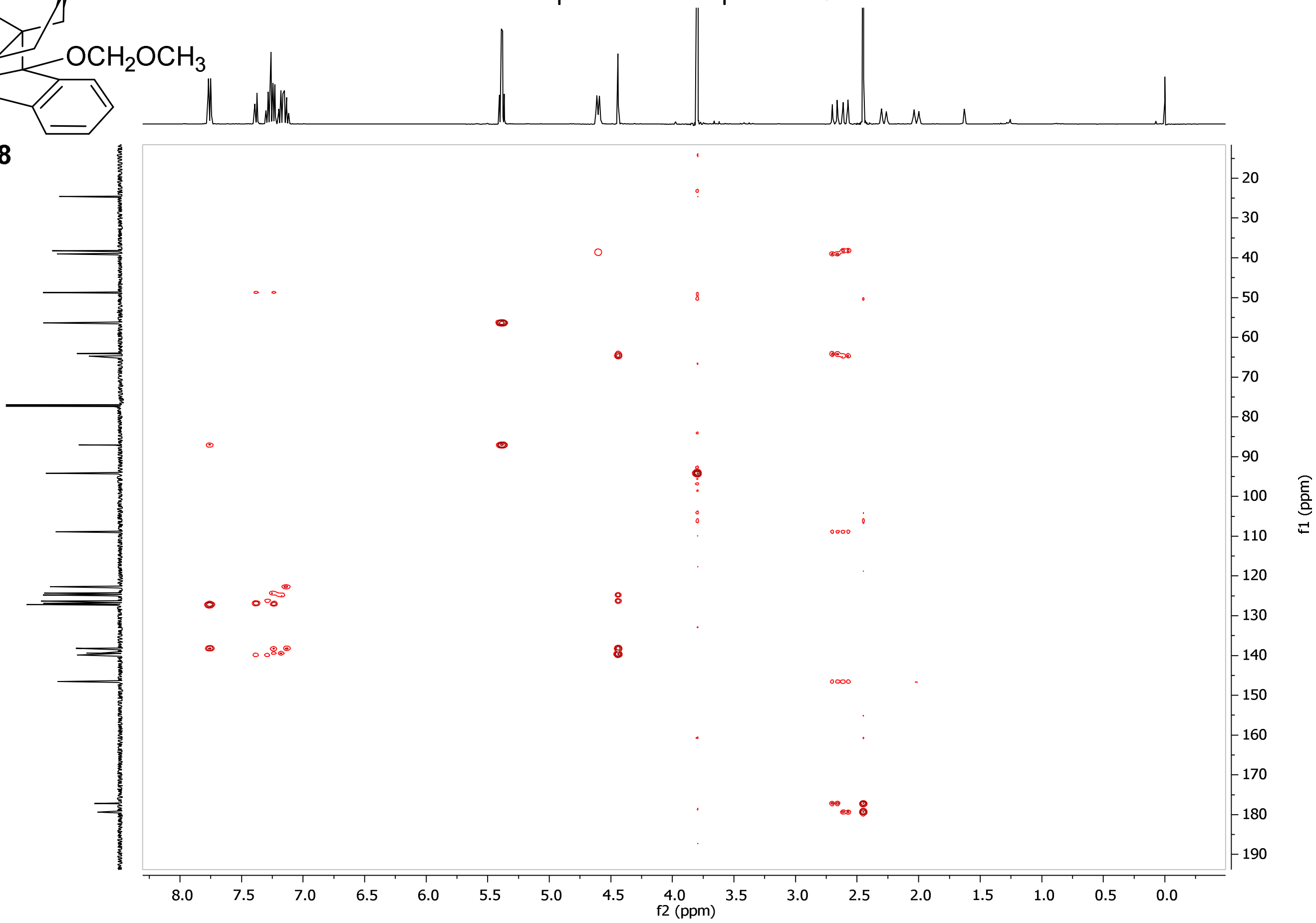

$^{1}H/^{13}C$ HETCOR (gHMBC sequence) (400 MHz, $CDCl_3$)

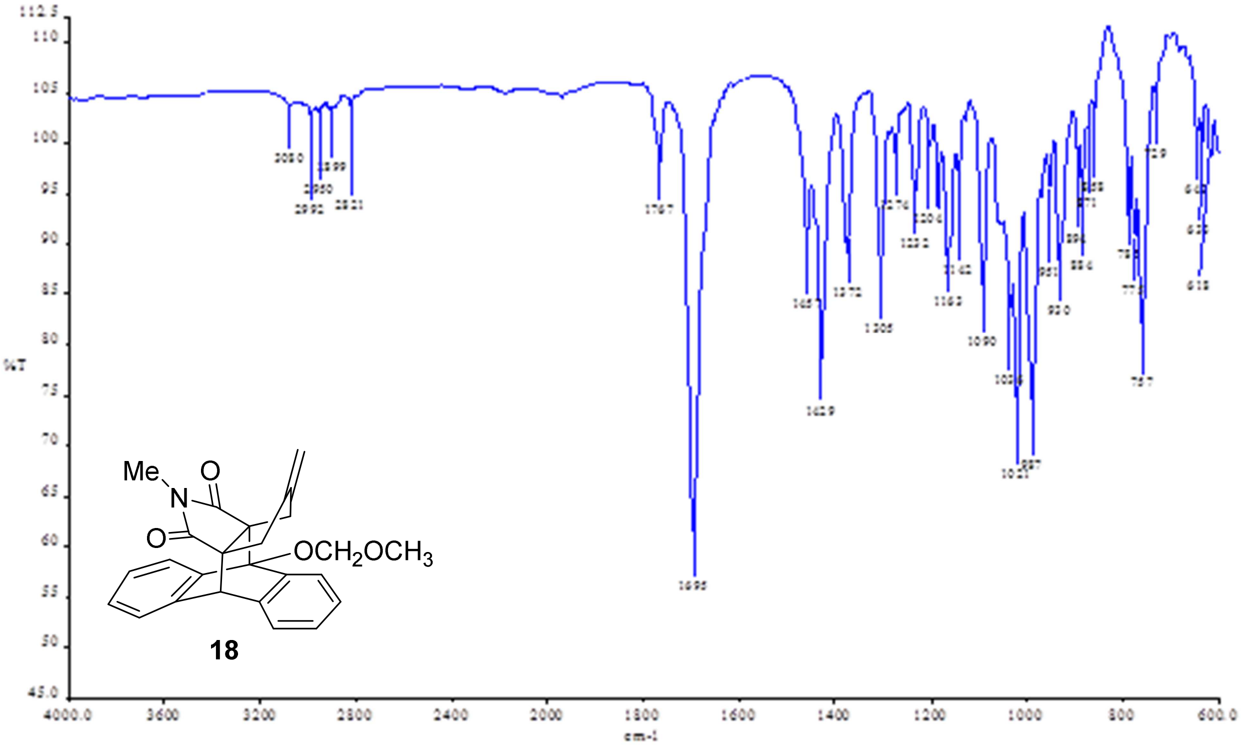

IR (ATR)

NRM and IR spectra of compound **19**

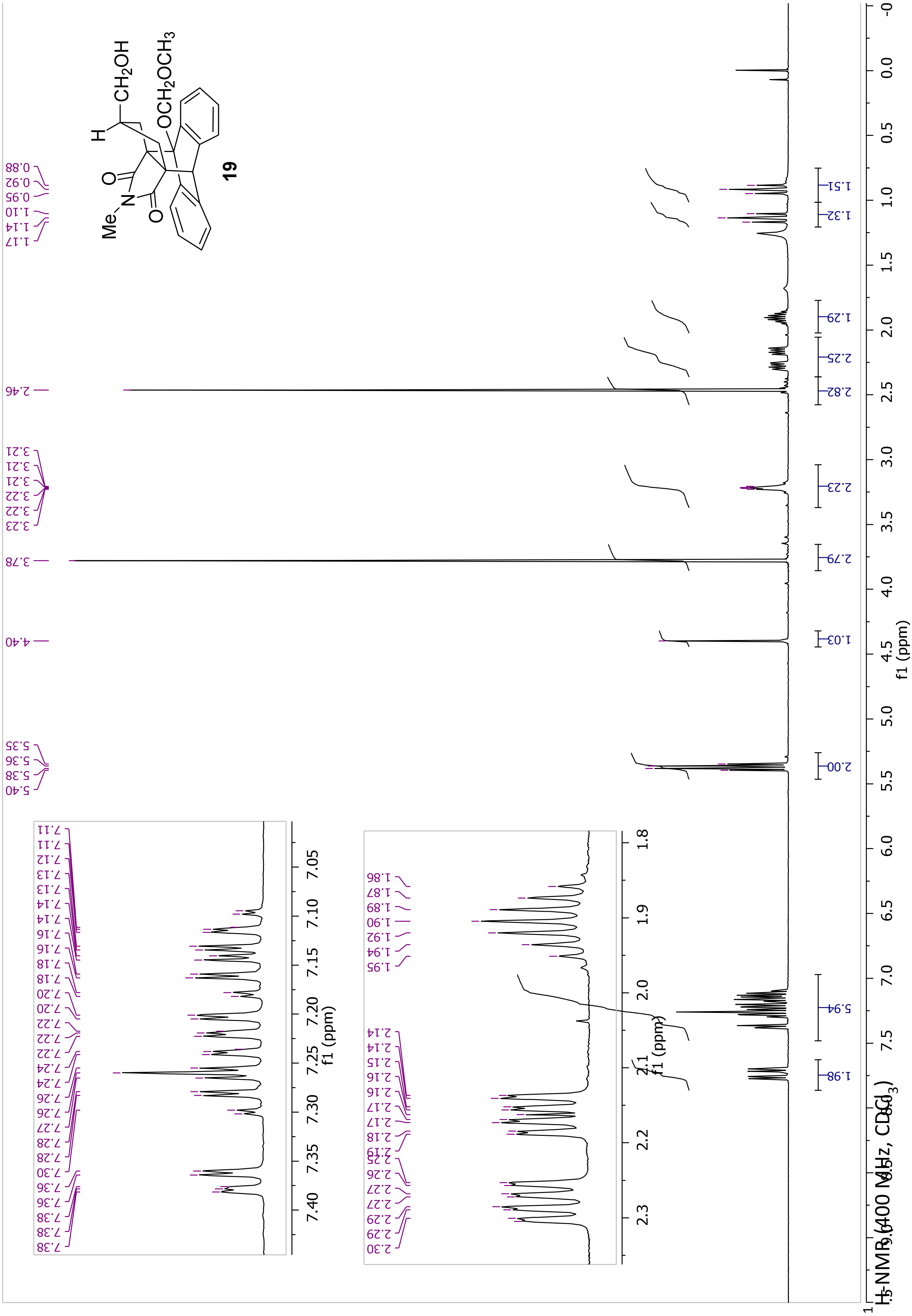

NRM and IR spectra of compound **19**

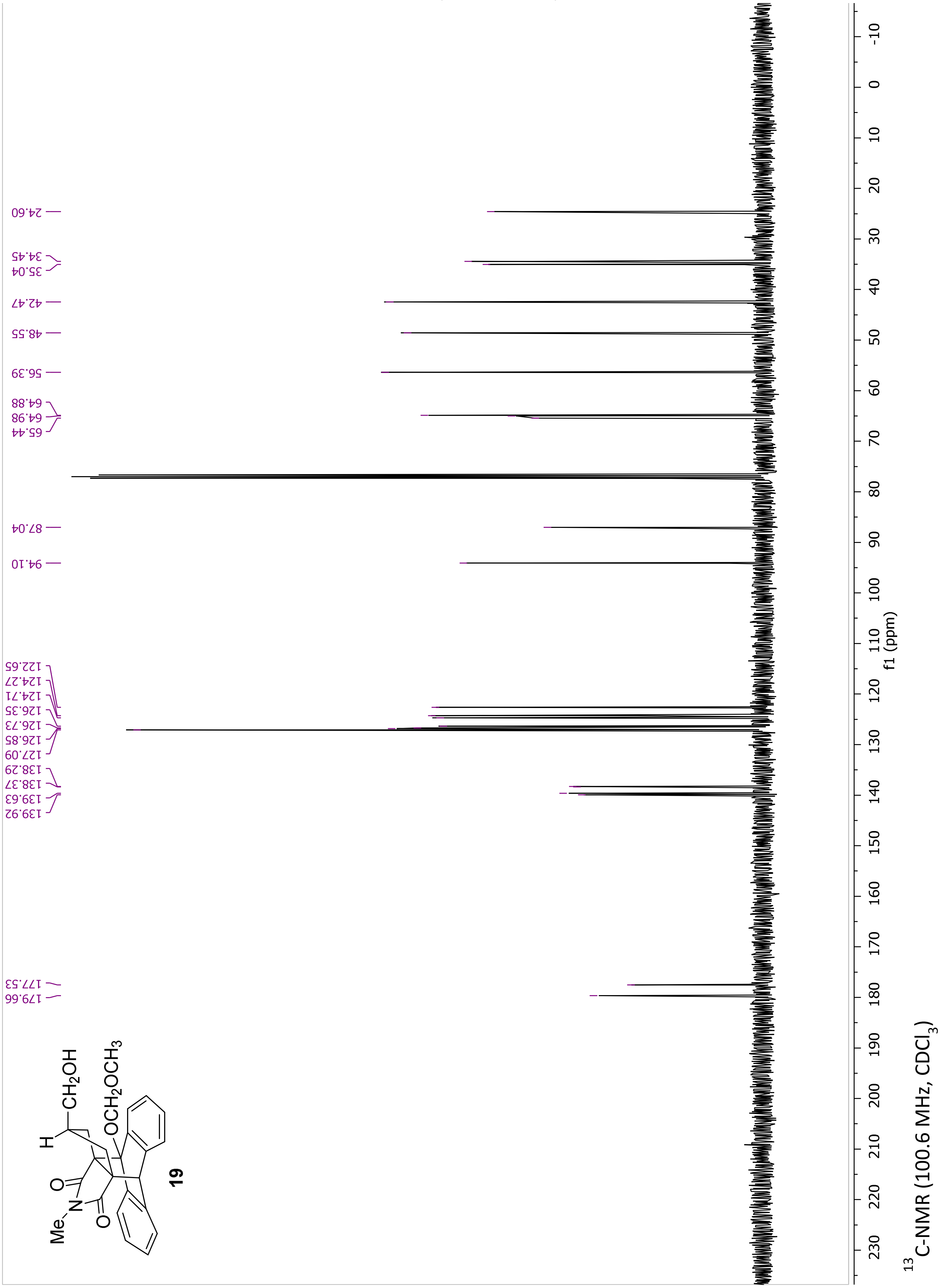

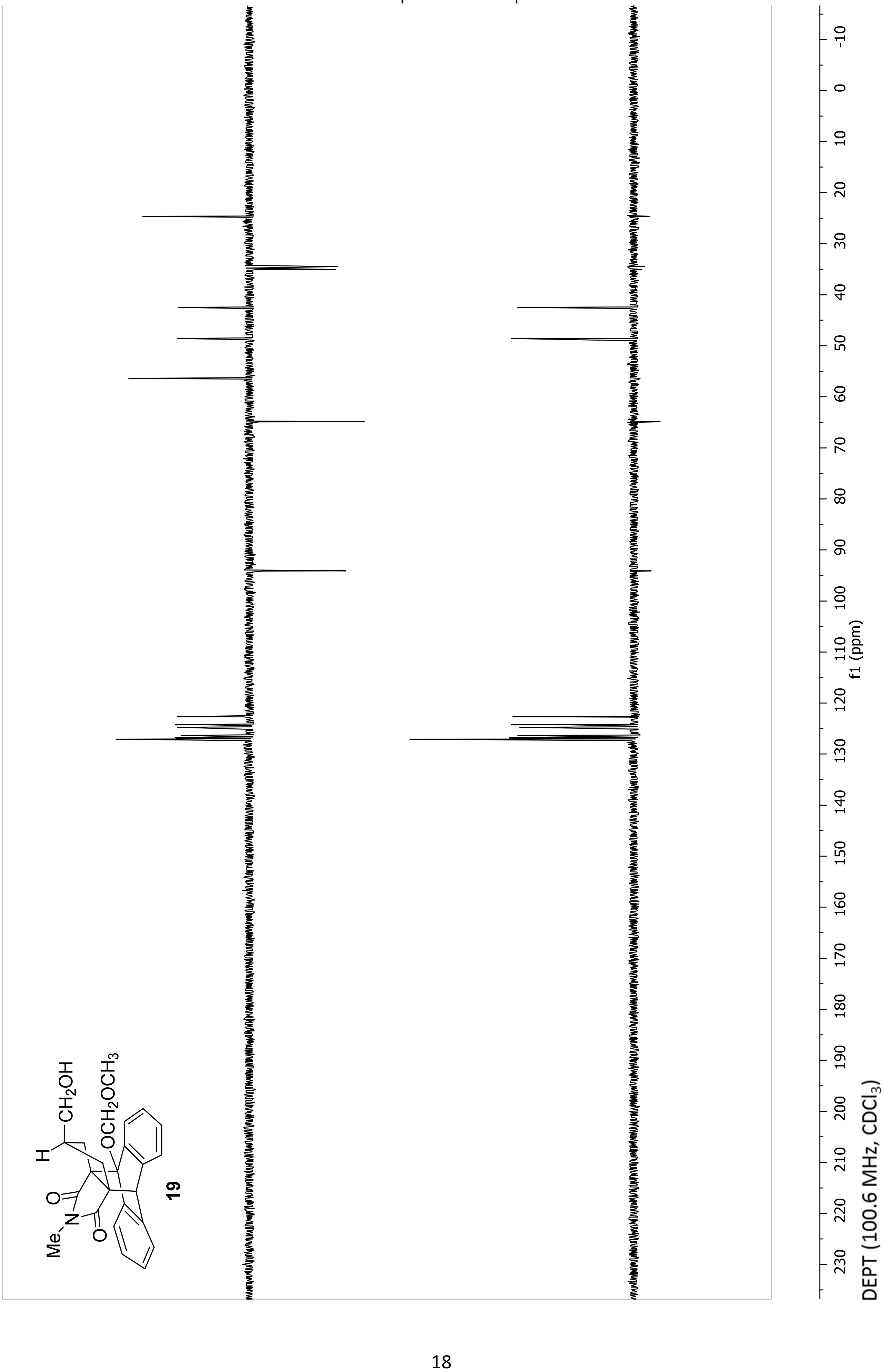

CH2OH
OCH2OCH3
H
Me
N
O
O
19
f1 (ppm)
DEPT (100.6 MHz, CDCl3)

NRM and IR spectra of compound **19**

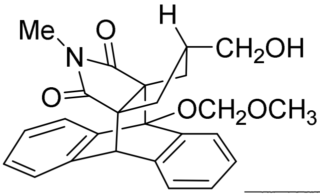

19

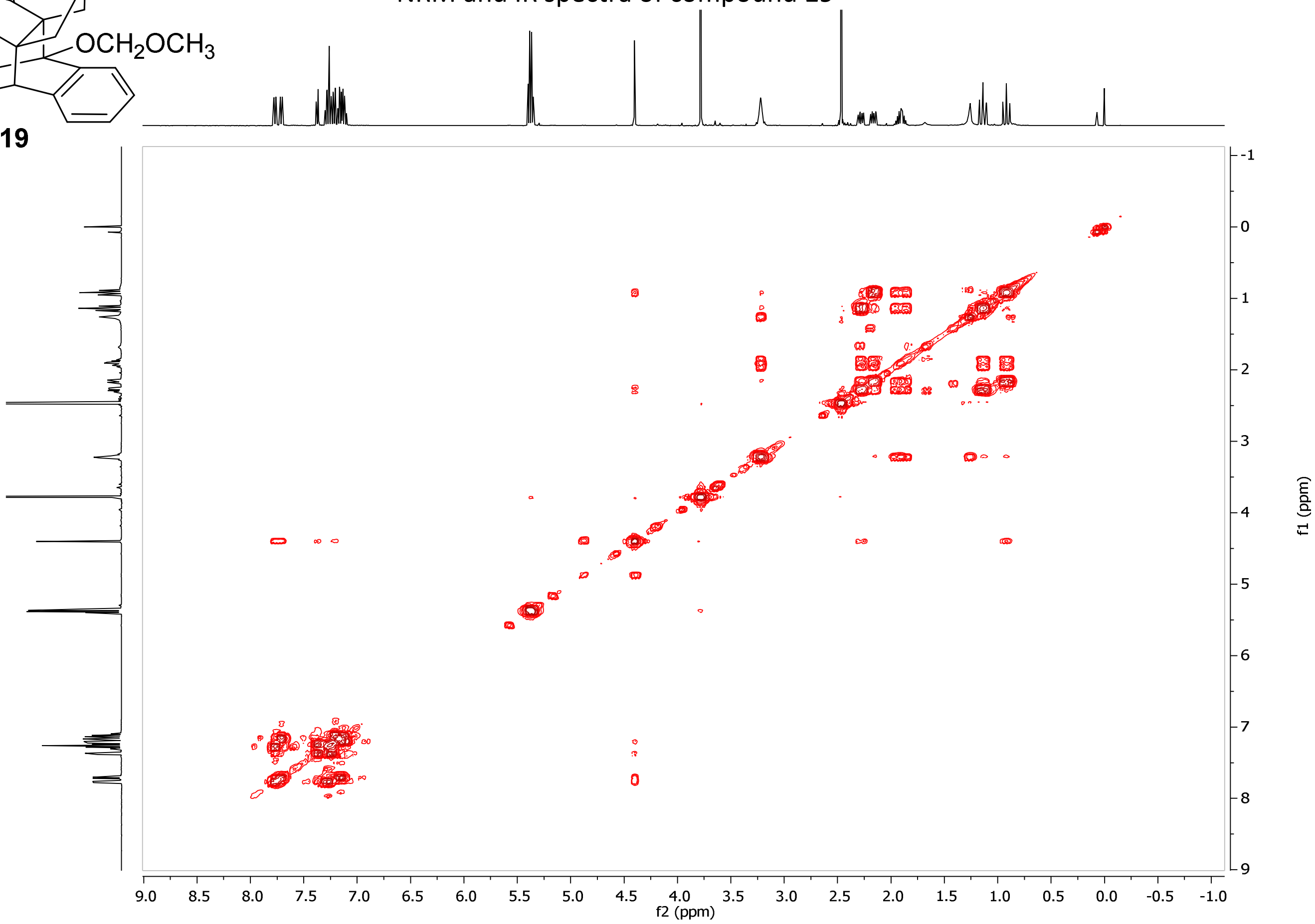

$^{1}H/^{1}H$ COSY (400 MHz, $CDCl_3$)

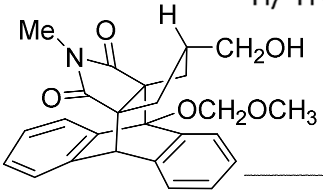

19

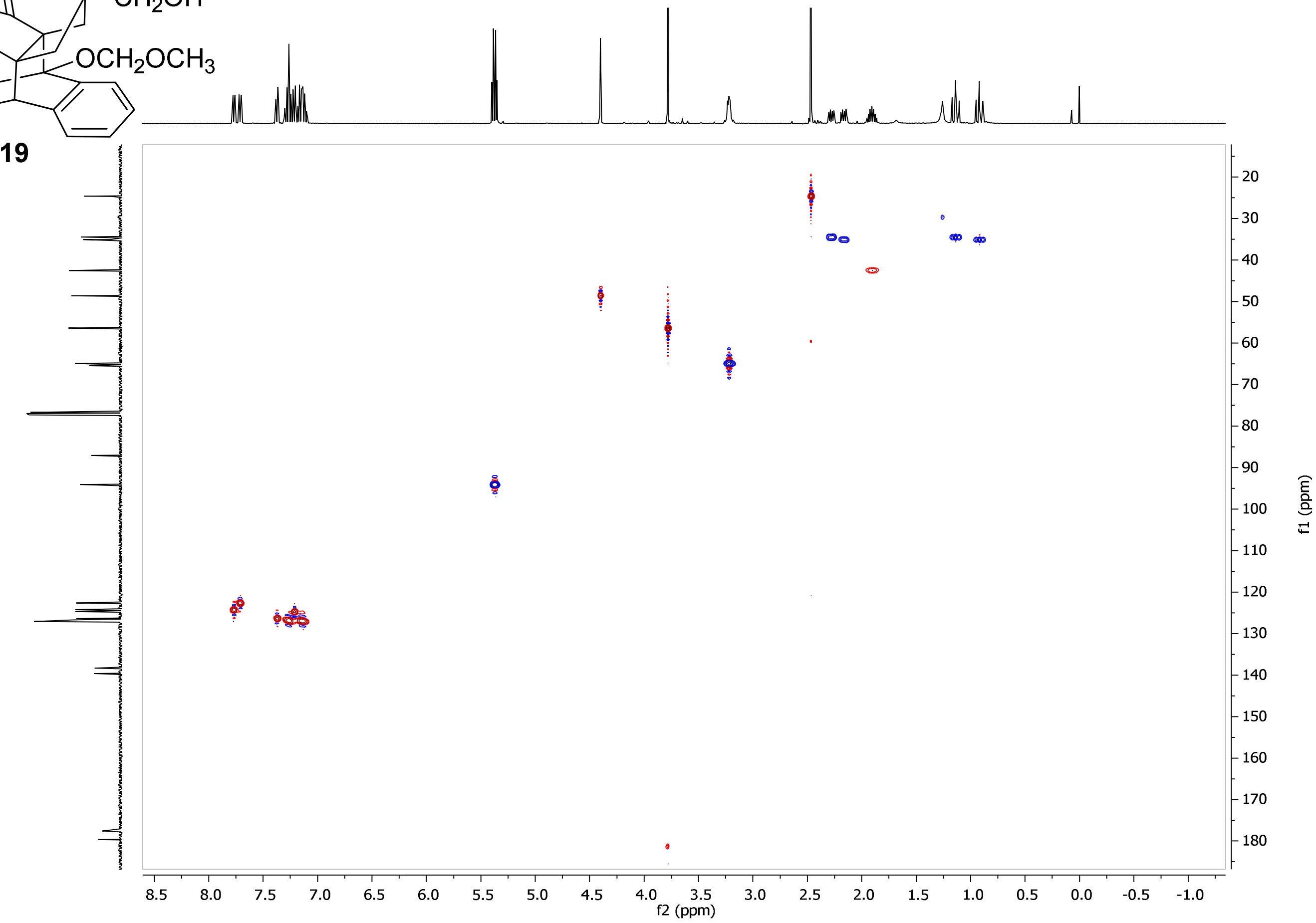

$^{1}H/^{13}C$ HETCOR (gHSQC sequence) (400 MHz, $CDCl_3$)

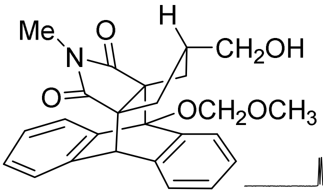

19

NRM and IR spectra of compound **19**

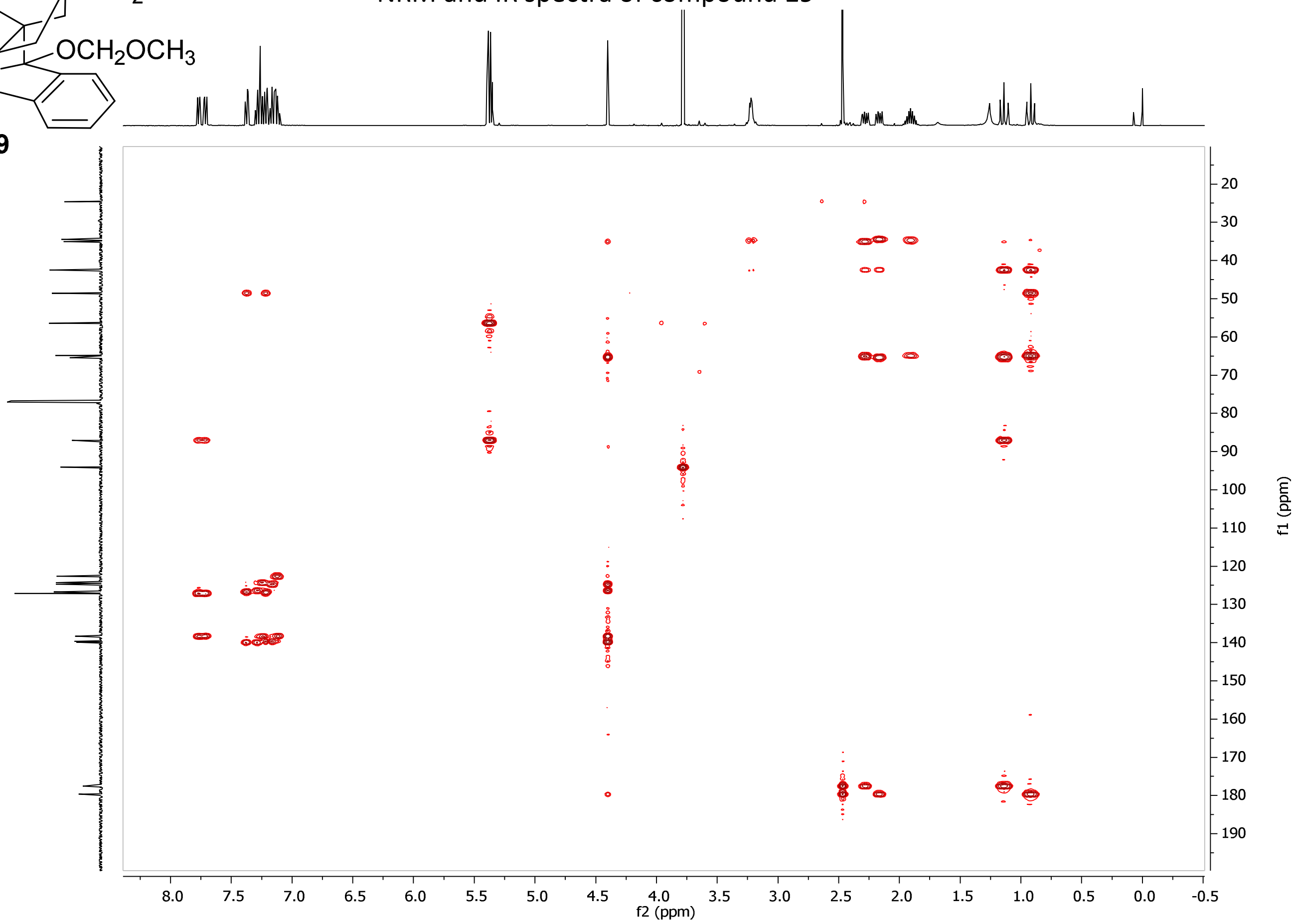

$^{1}H/^{13}C$ HETCOR (gHMBC sequence) (400 MHz, $CDCl_3$)

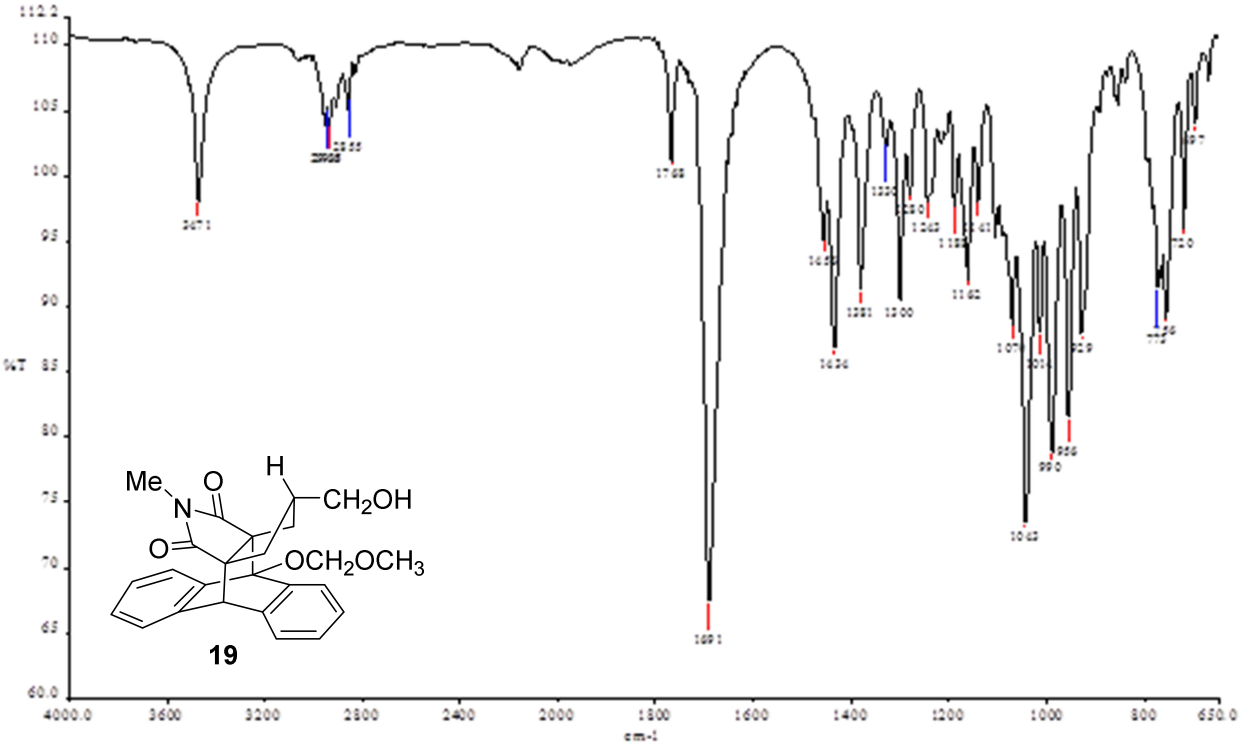

IR (ATR)

NMR and IR spectra of compound **20**

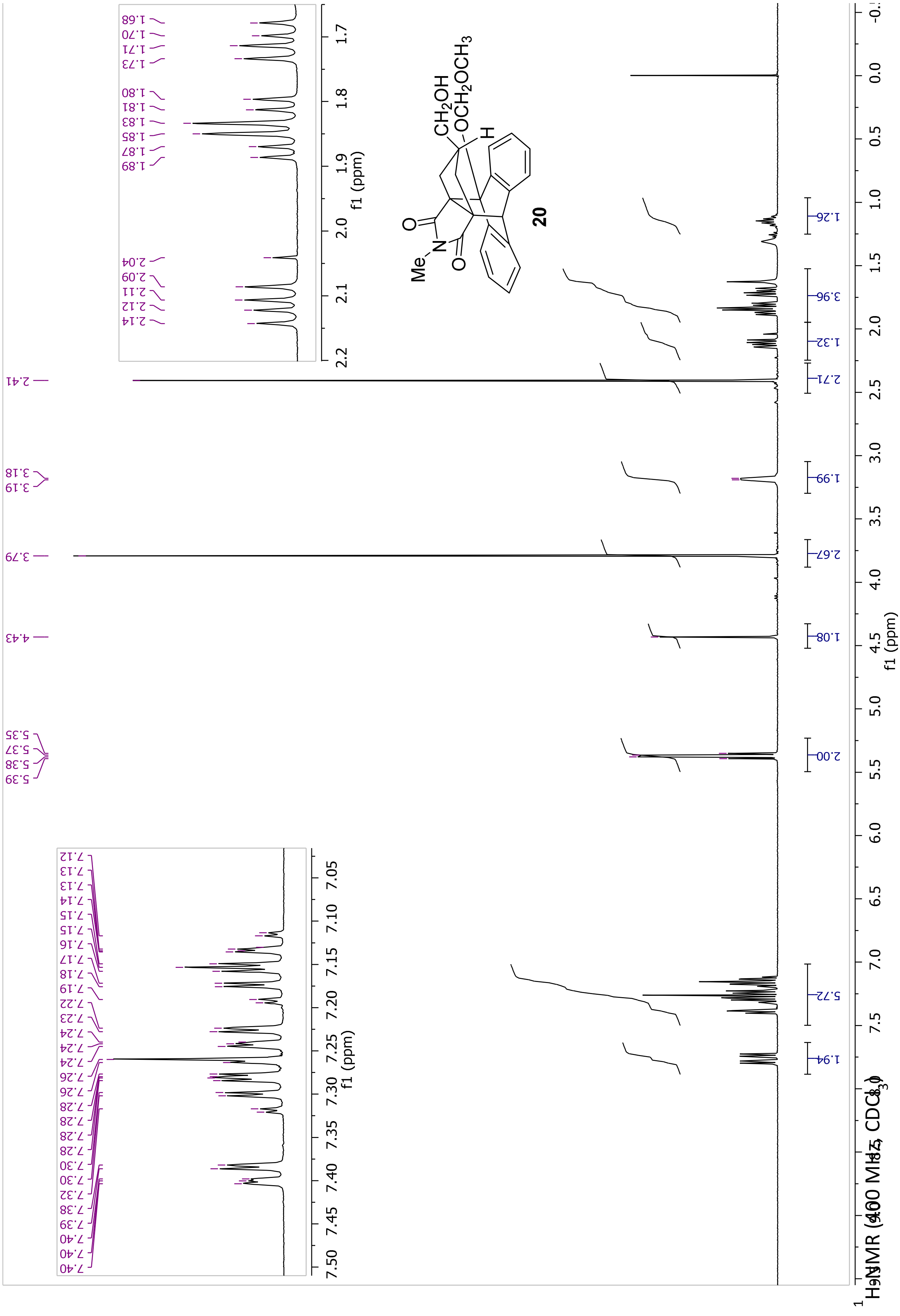

NMR and IR spectra of compound **20**

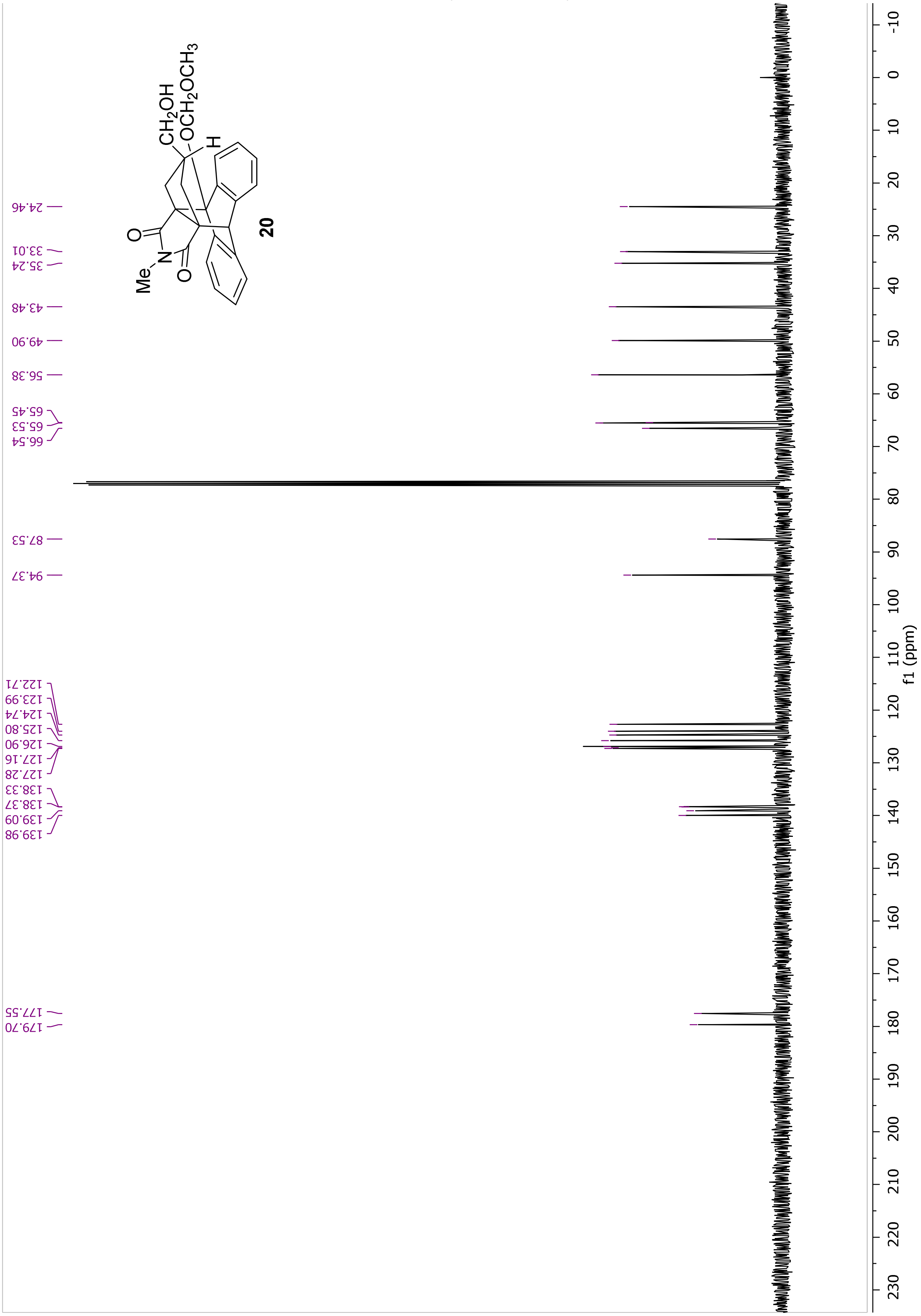

^{13}C-NMR (100.6 MHz, $CDCl_3$)

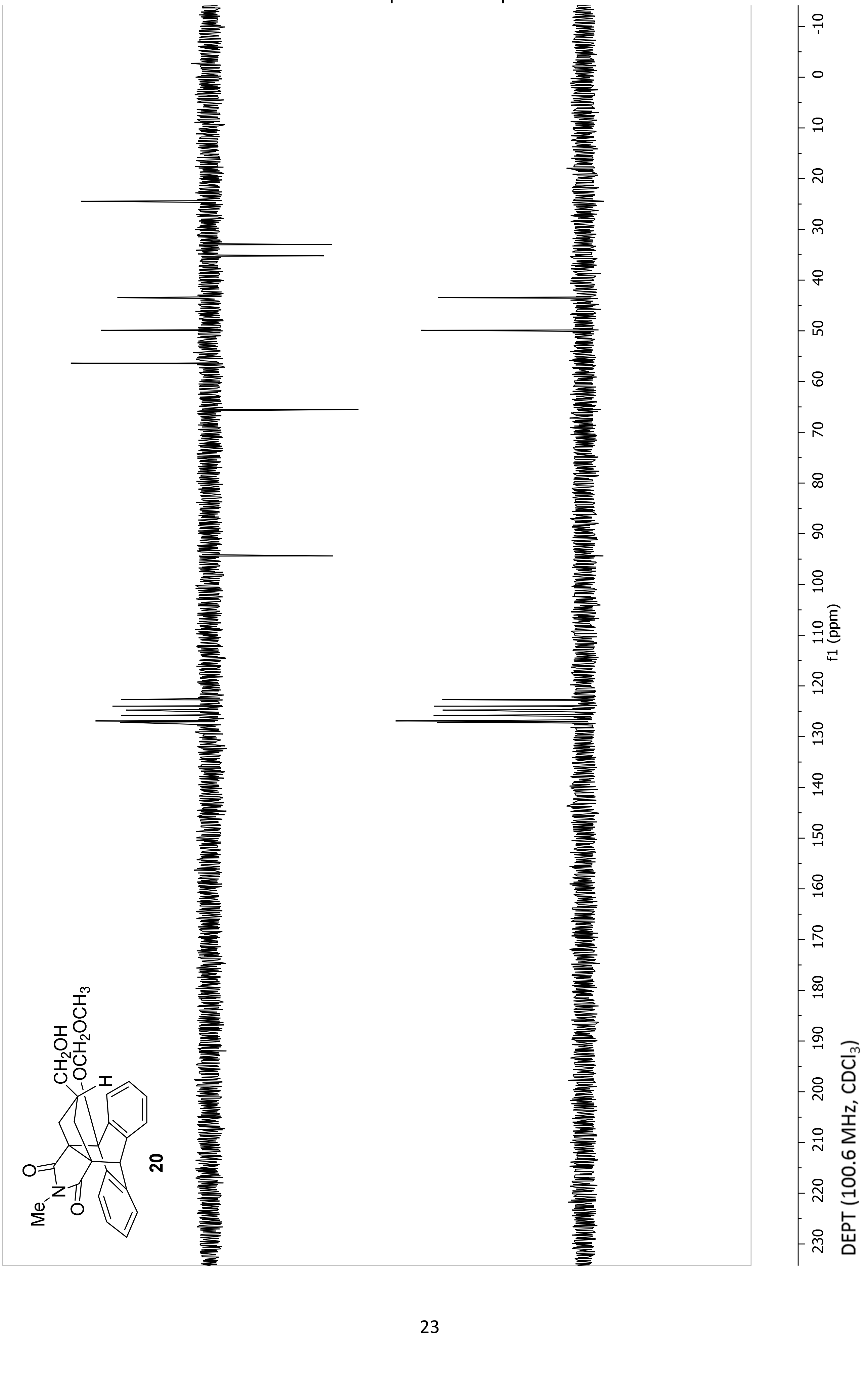
Me
N
O
O
CH2OH
OCH2OCH3
H
20
f1 (ppm)
DEPT (100.6 MHz, CDCl3)

NMR and IR spectra of compound **20**

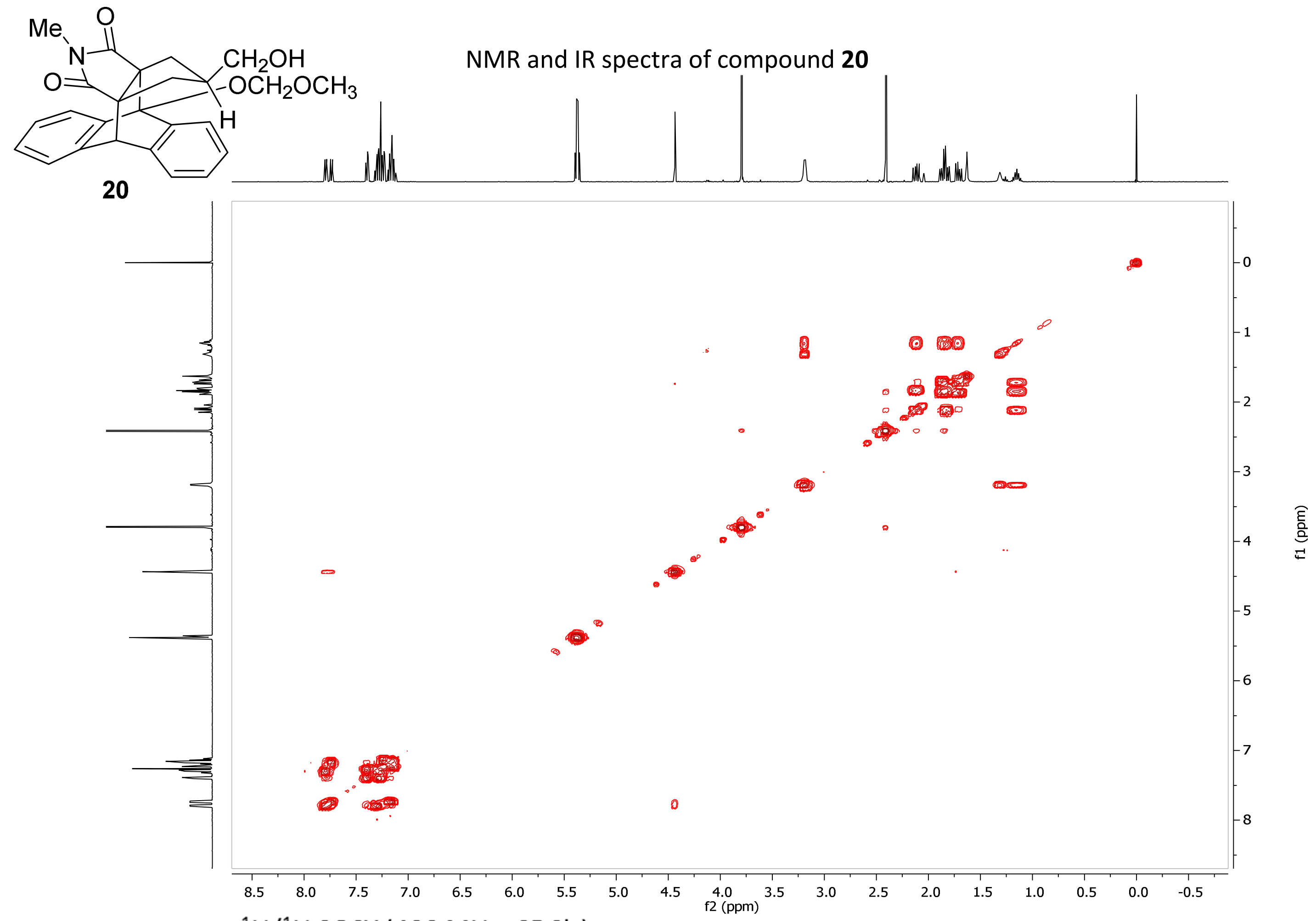

$^{1}H/^{1}H$ COSY (400 MHz, $CDCl_3$)

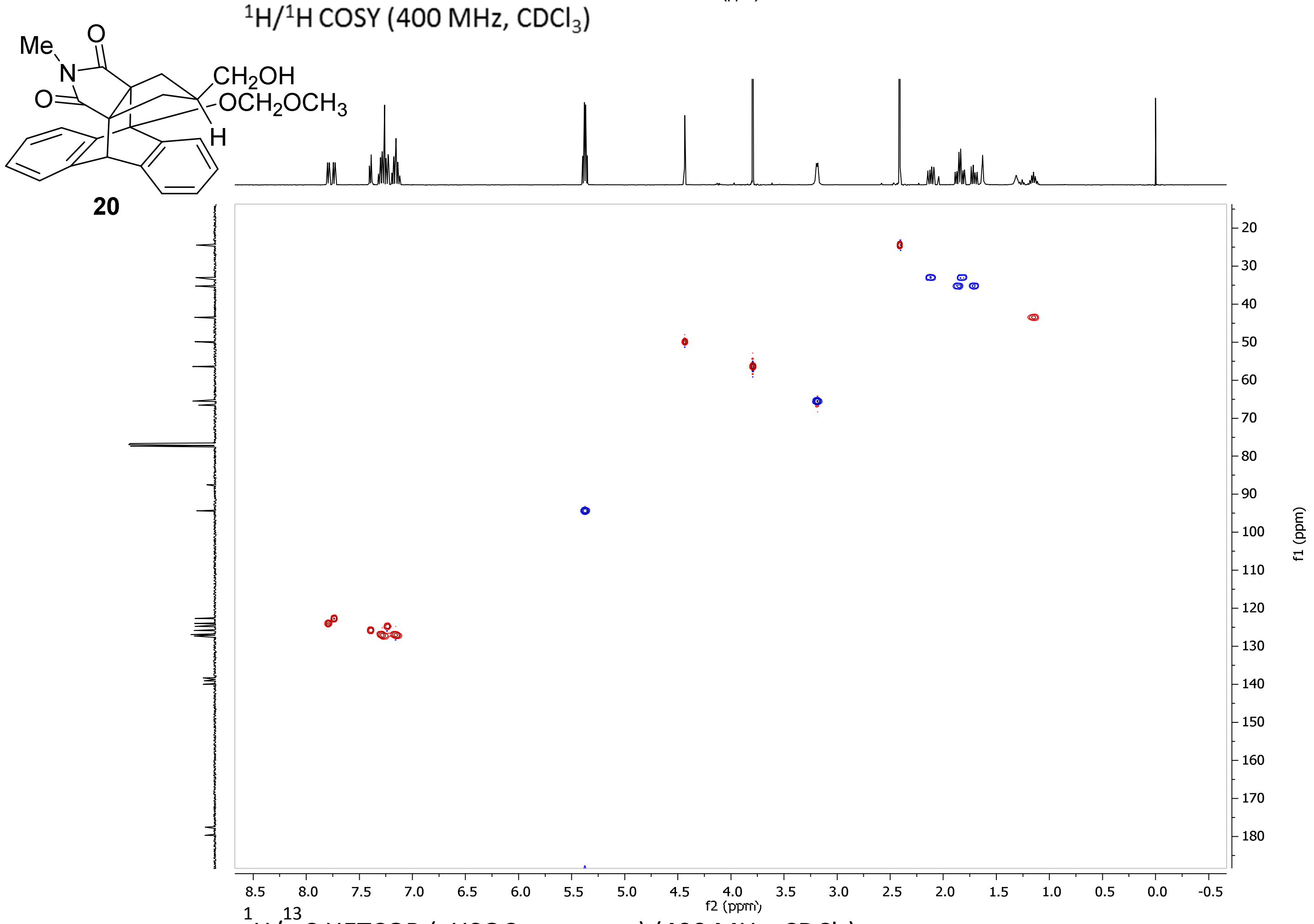

$^{1}H/^{13}C$ HETCOR (gHSQC sequence) (400 MHz, $CDCl_3$)

NMR and IR spectra of compound **20**

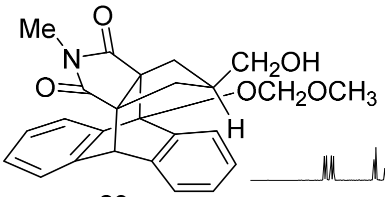

20

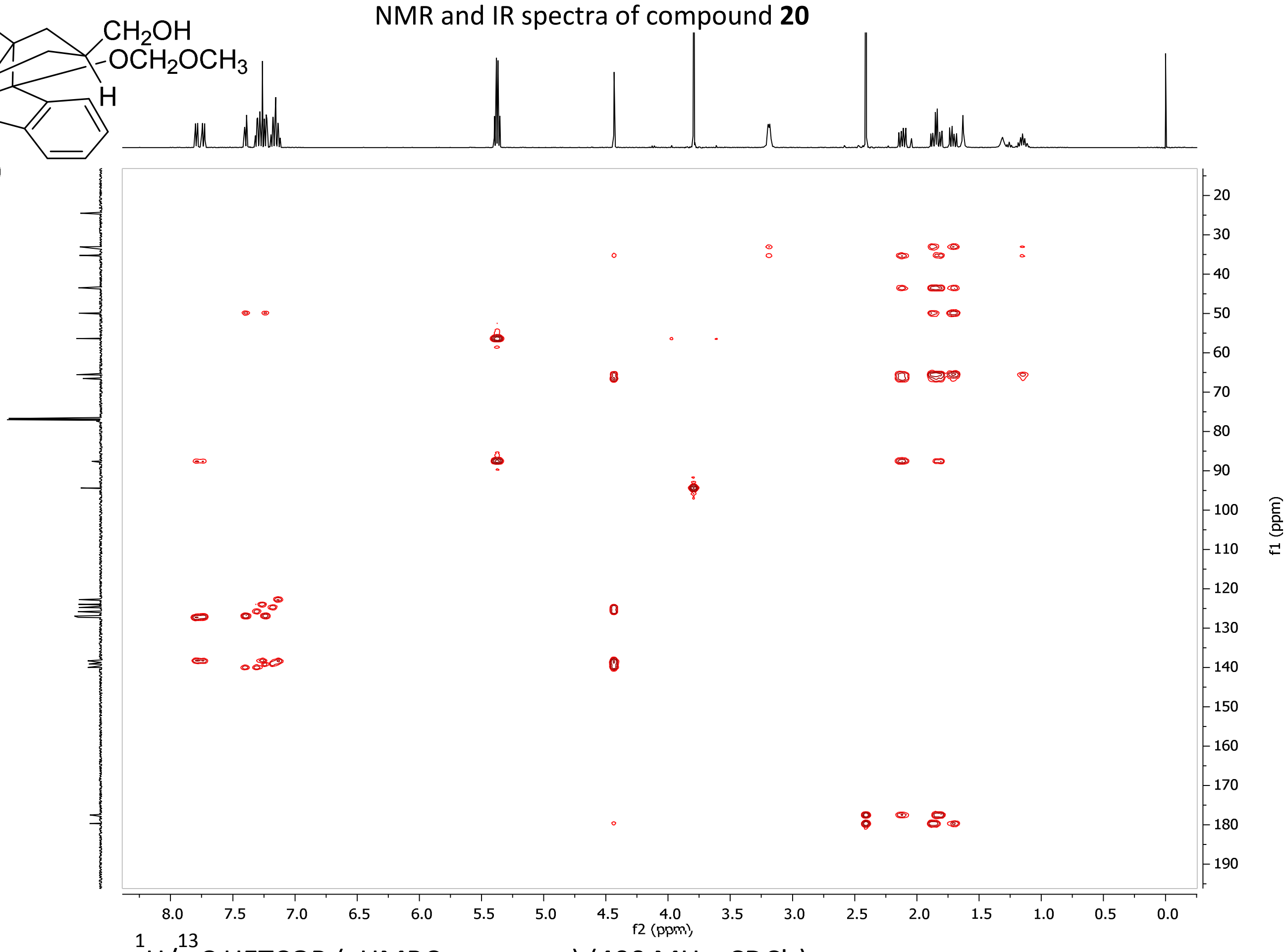

$^{1}H/^{13}C$ HETCOR (gHMBC sequence) (400 MHz, $CDCl_3$)

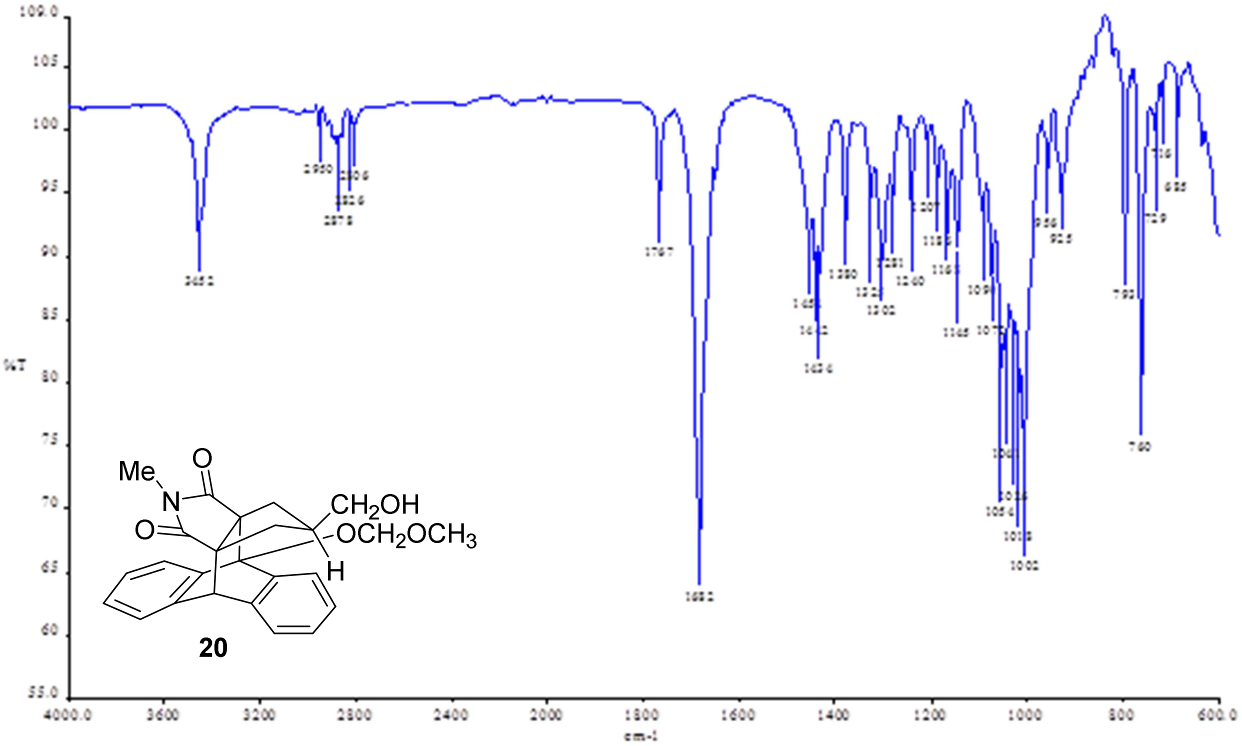

IR (ATR)

NMR and IR spectra of compound **22**

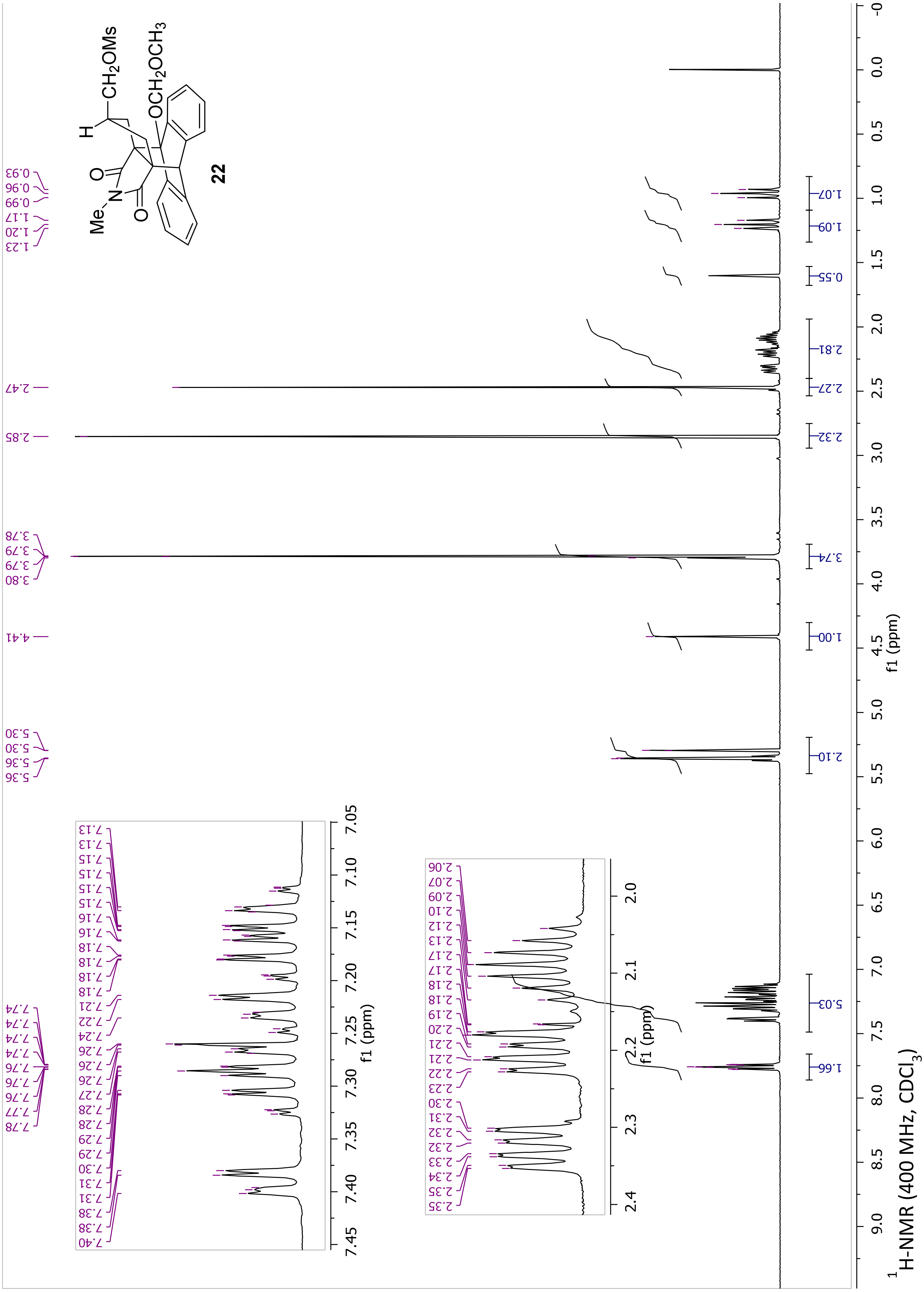

NMR and IR spectra of compound **22**

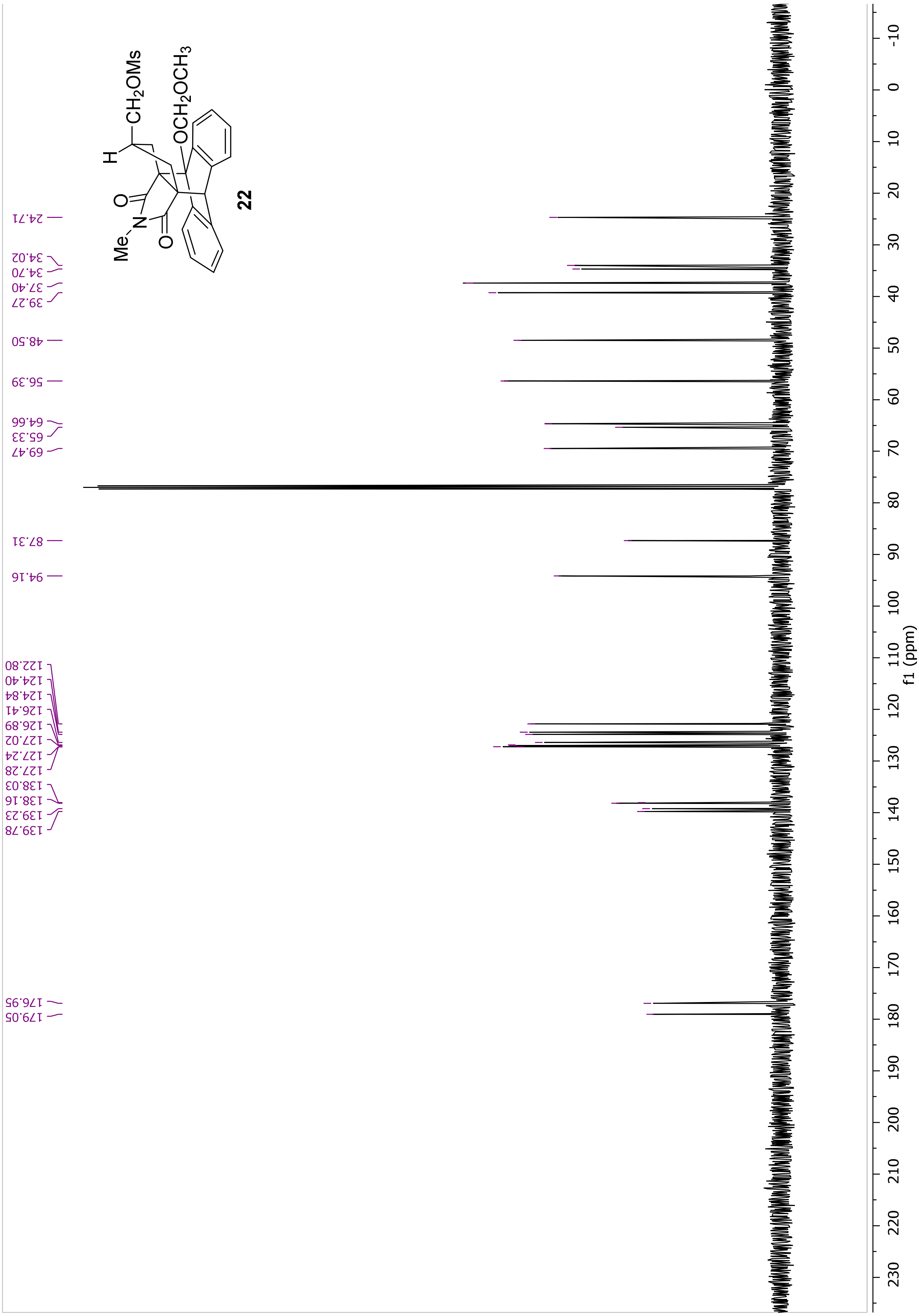

^{13}C-NMR (100.6 MHz, $CDCl_3$)

NMR and IR spectra of compound **22**

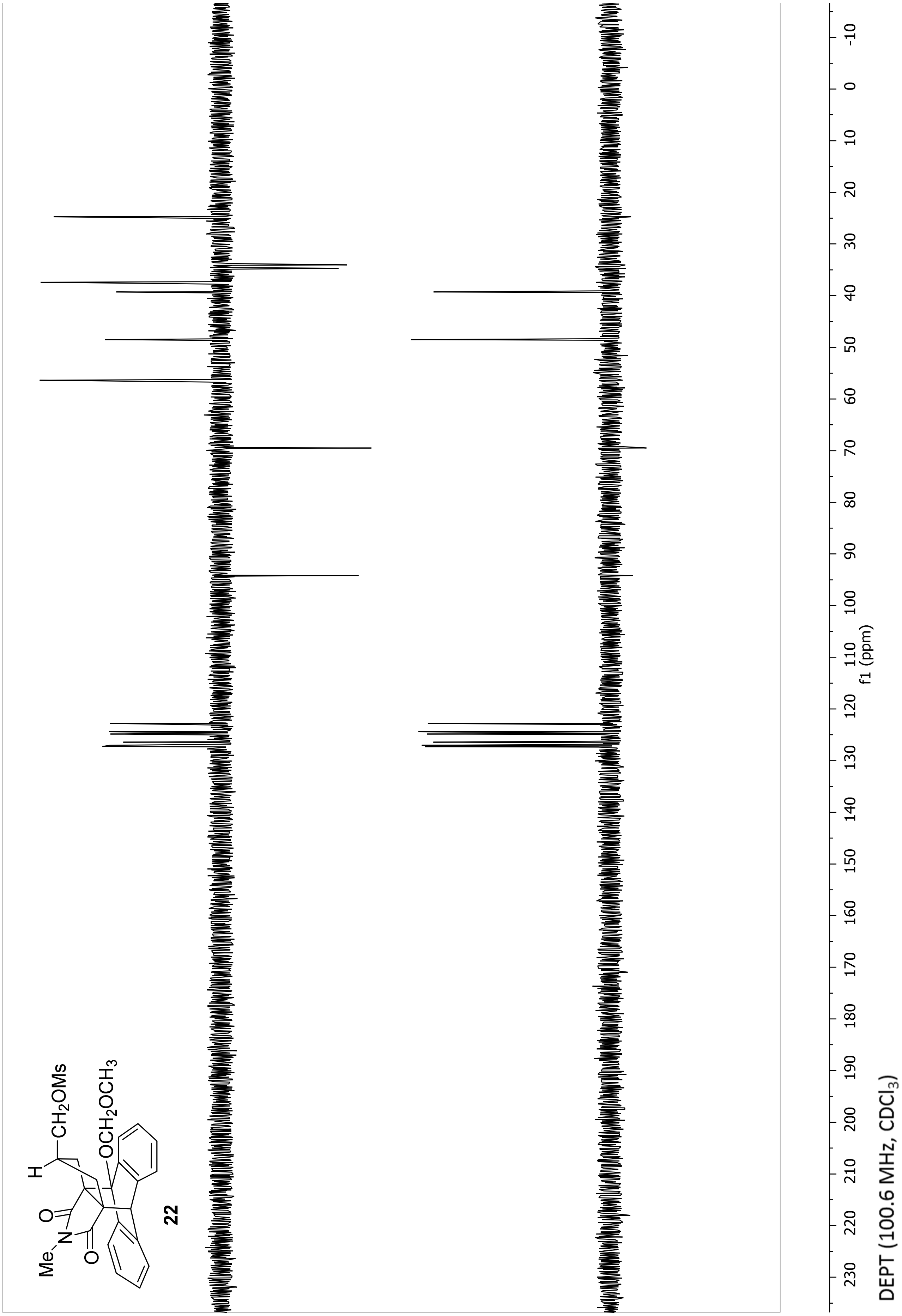

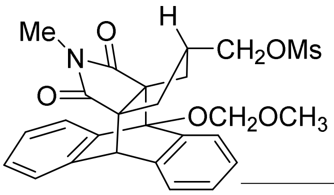

22

NMR and IR spectra of compound **22**

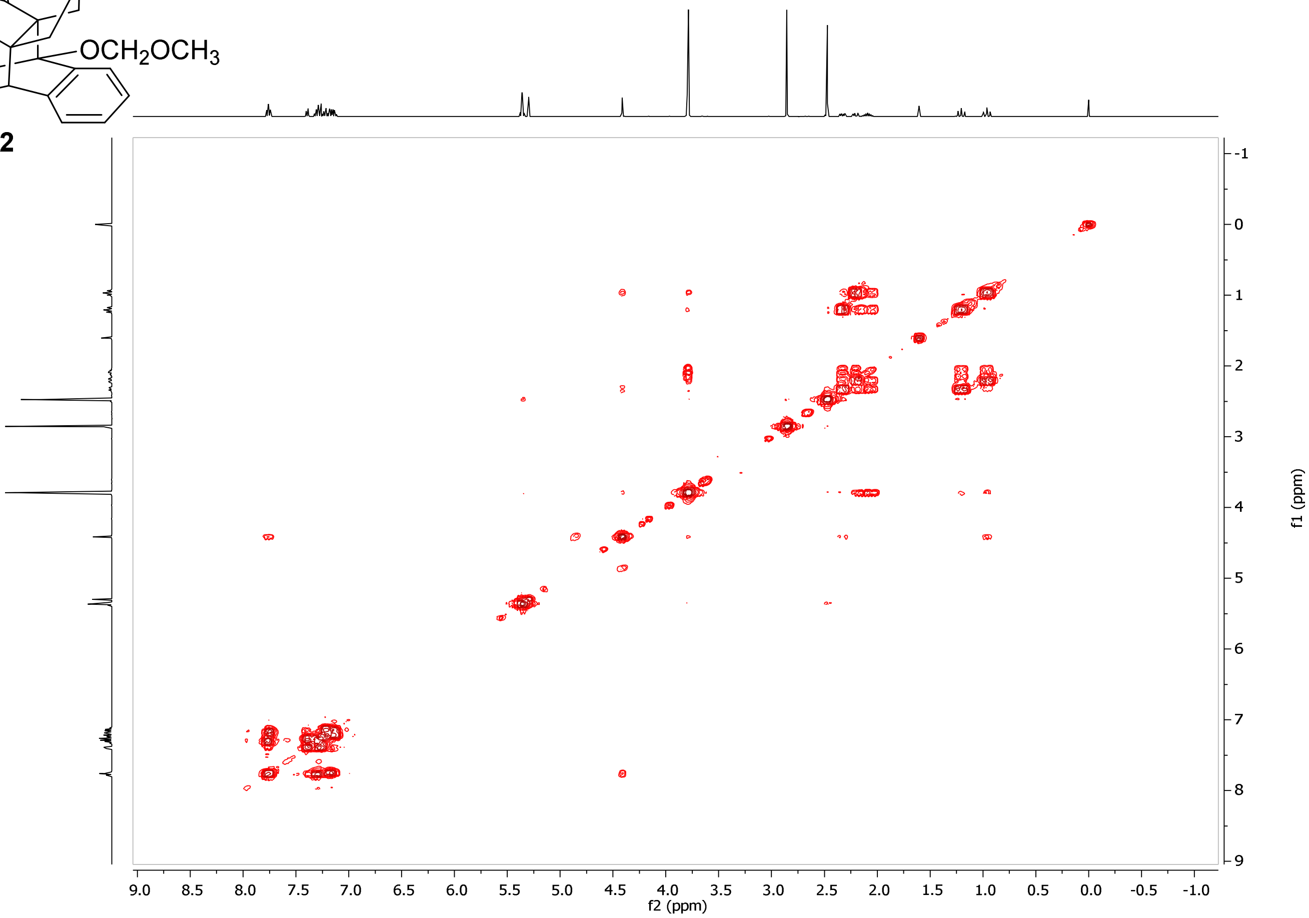

$^1H/^1H$ COSY (400 MHz, $CDCl_3$)

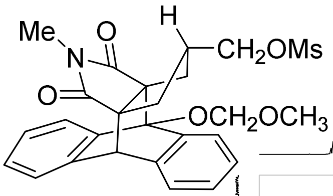

22

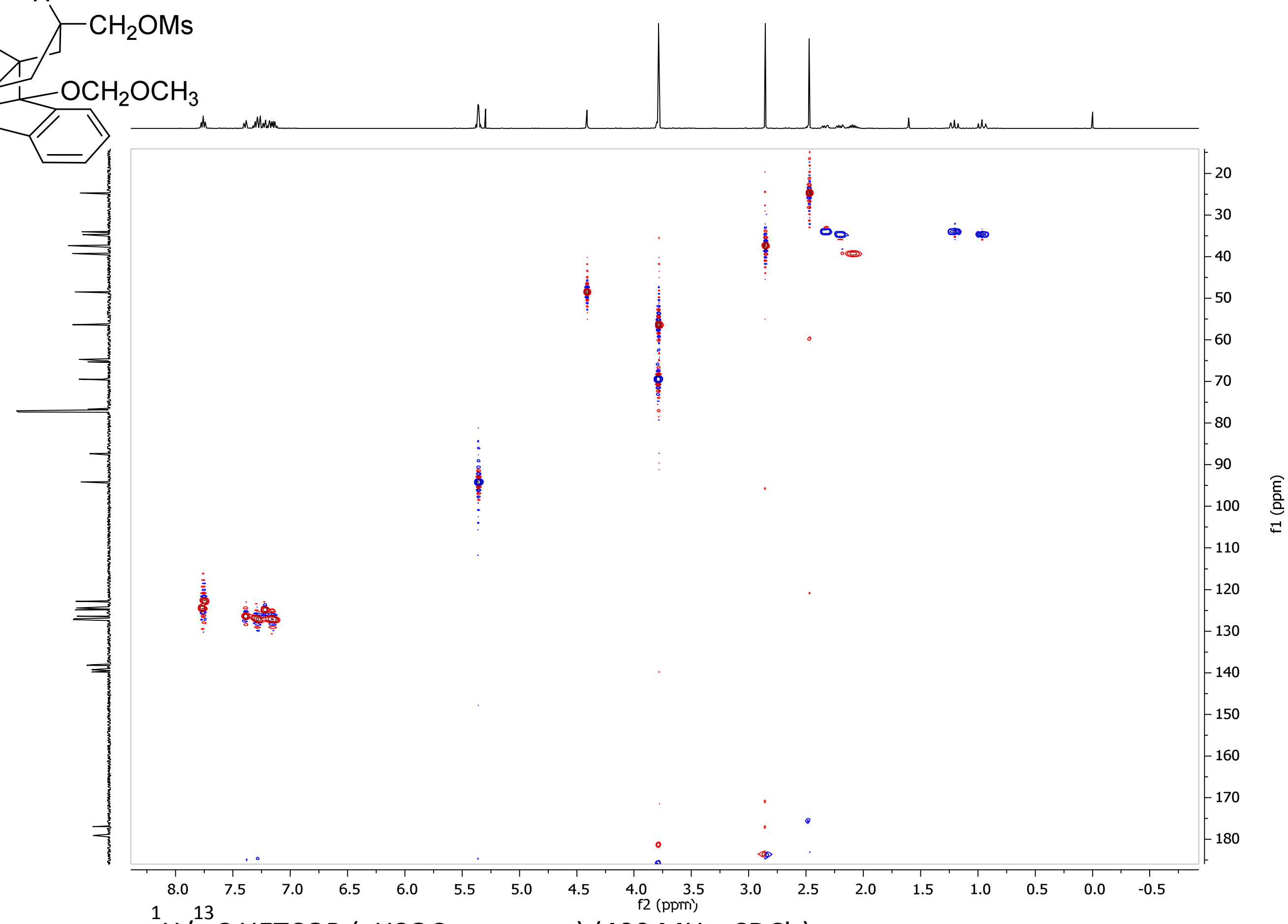

$^1H/^{13}C$ HETCOR (gHSQC sequence) (400 MHz, $CDCl_3$)

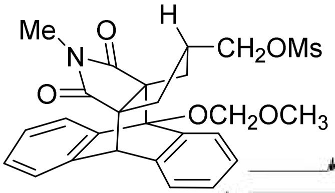

22

NMR and IR spectra of compound **22**

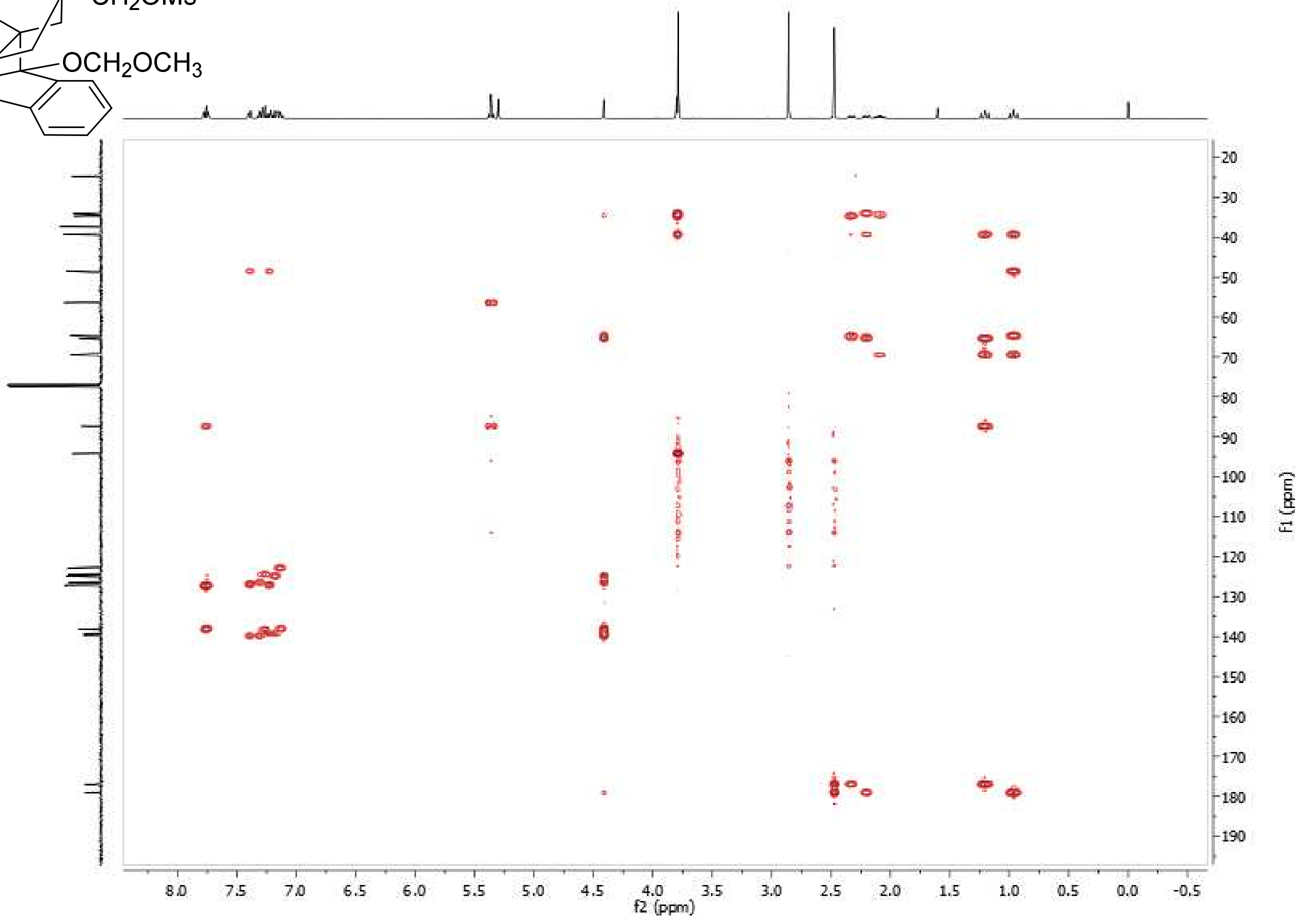

$^{1}H/^{13}C$ HETCOR (gHMBC sequence) (400 MHz, $CDCl_3$)

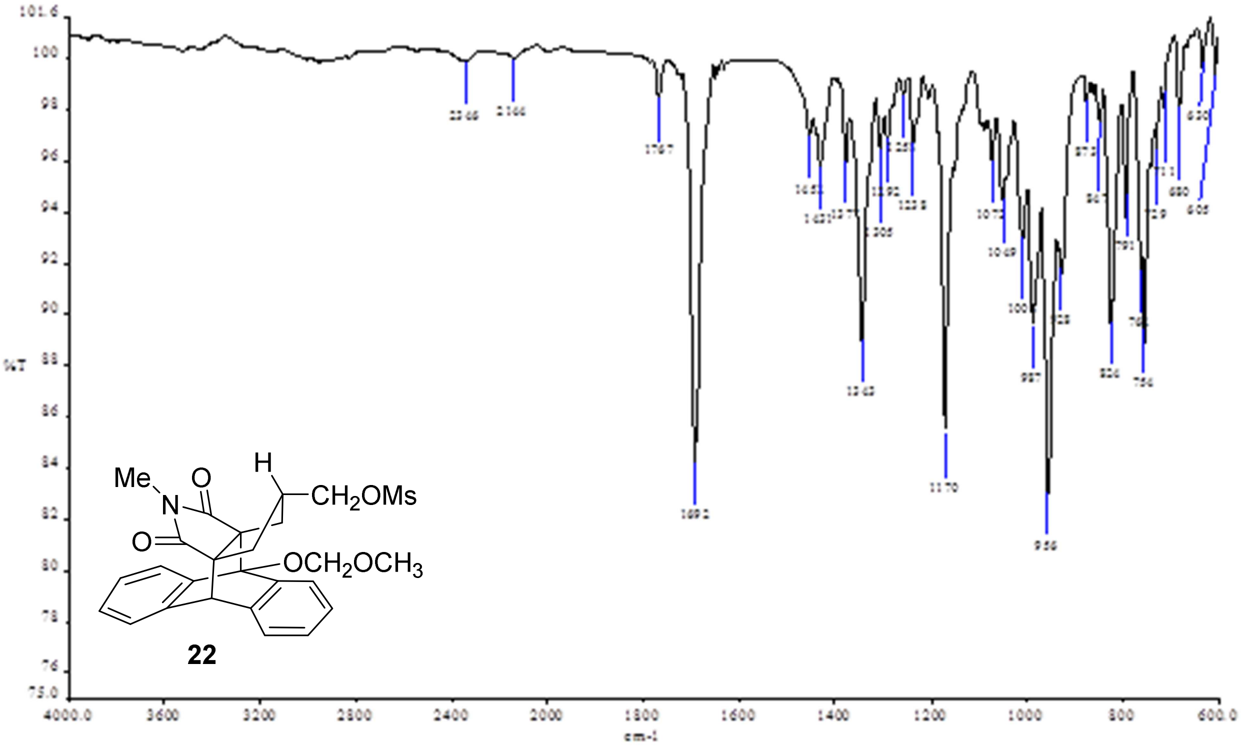

IR (ATR

NMR and IR spectra of compound **21**

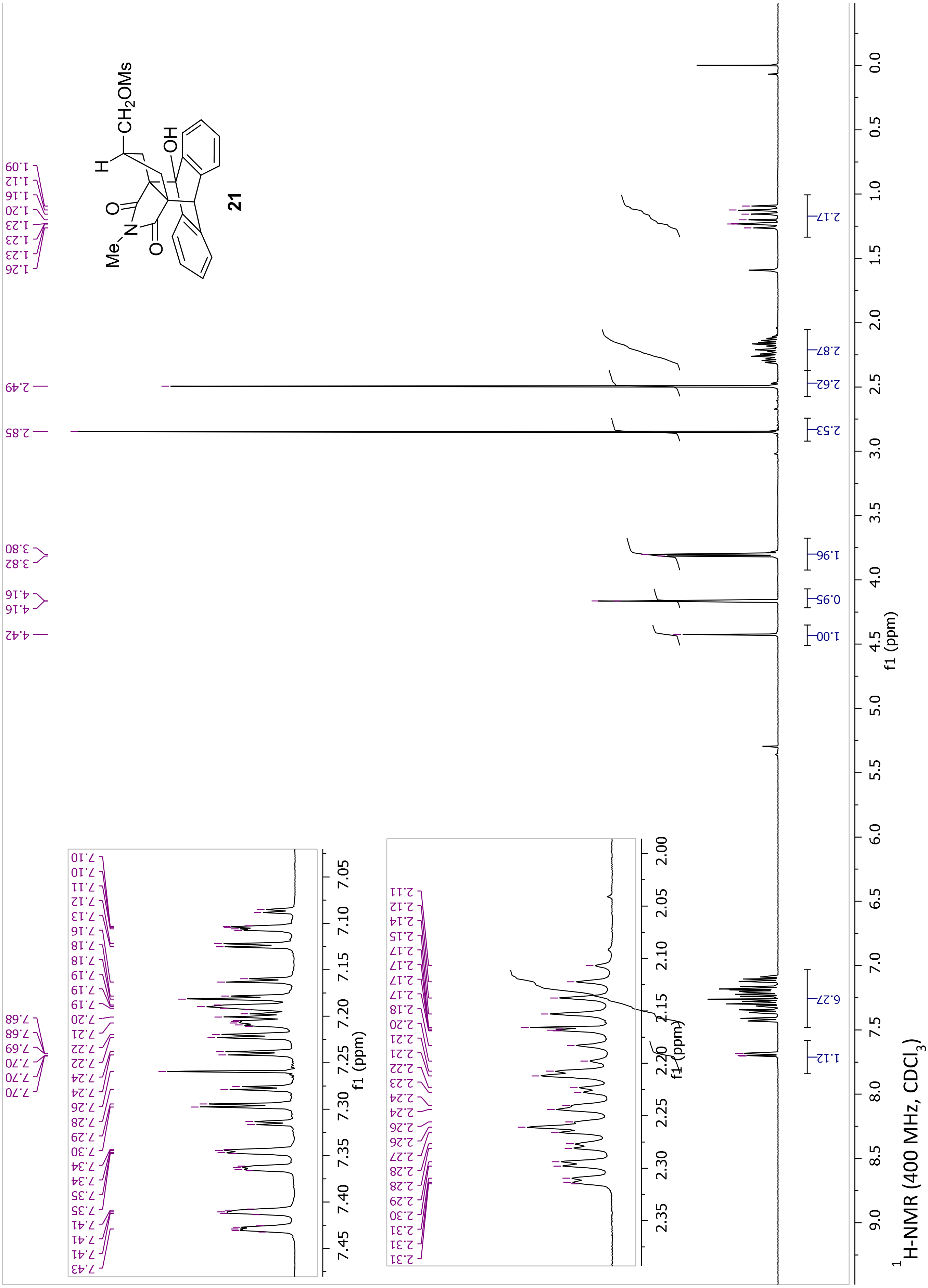

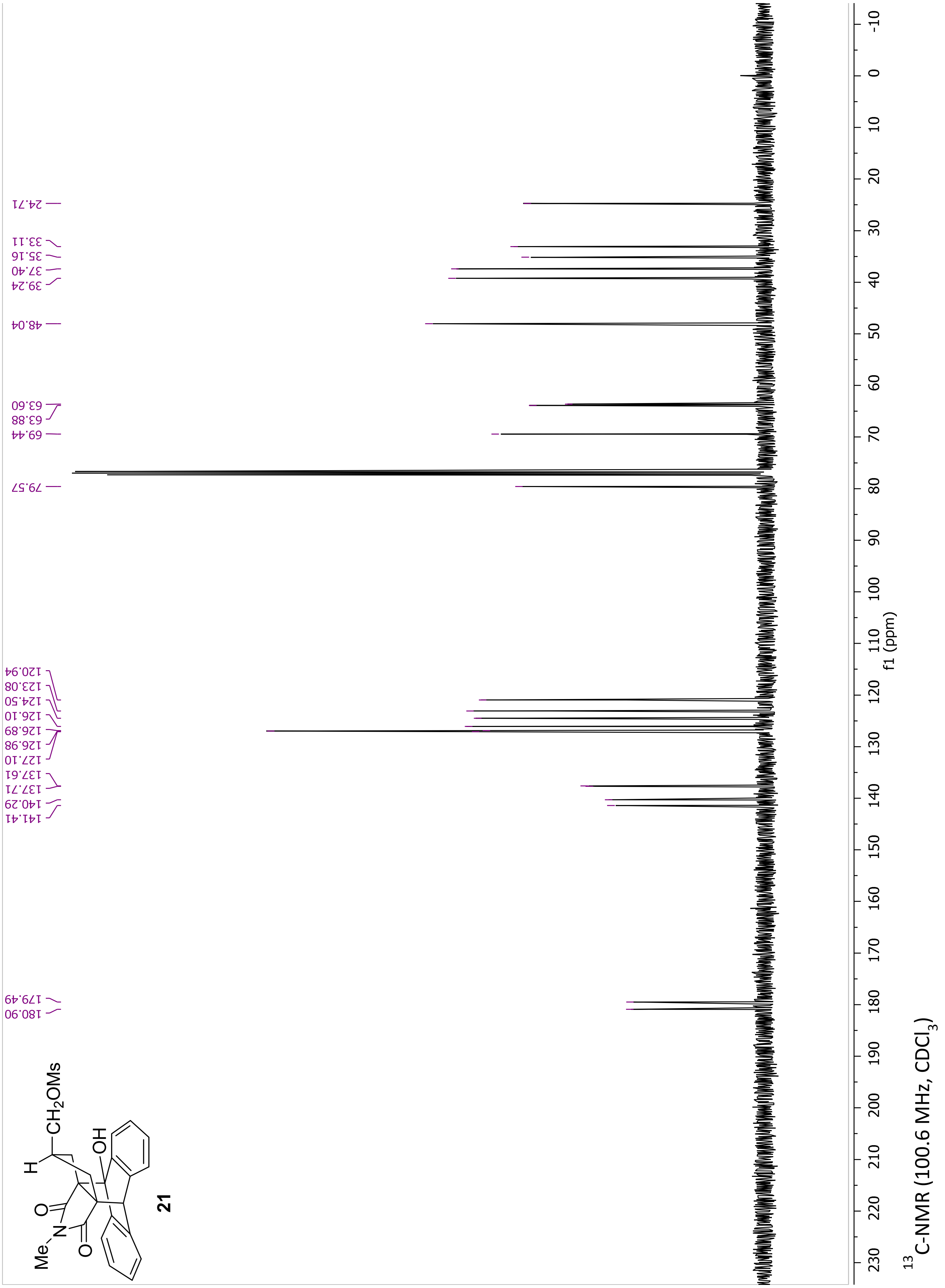
24.71
33.11
35.16
37.40
39.24
48.04
63.60
63.88
69.44
79.57
120.94
123.08
124.50
126.10
126.89
126.98
127.10
137.61
137.71
140.29
141.41
179.49
180.90
f1 (ppm)
^{13}C-NMR (100.6 MHz, $CDCl_3$)
Me
N
O
O
H
CH_2OMs
OH
21

NMR and IR spectra of compound **21**

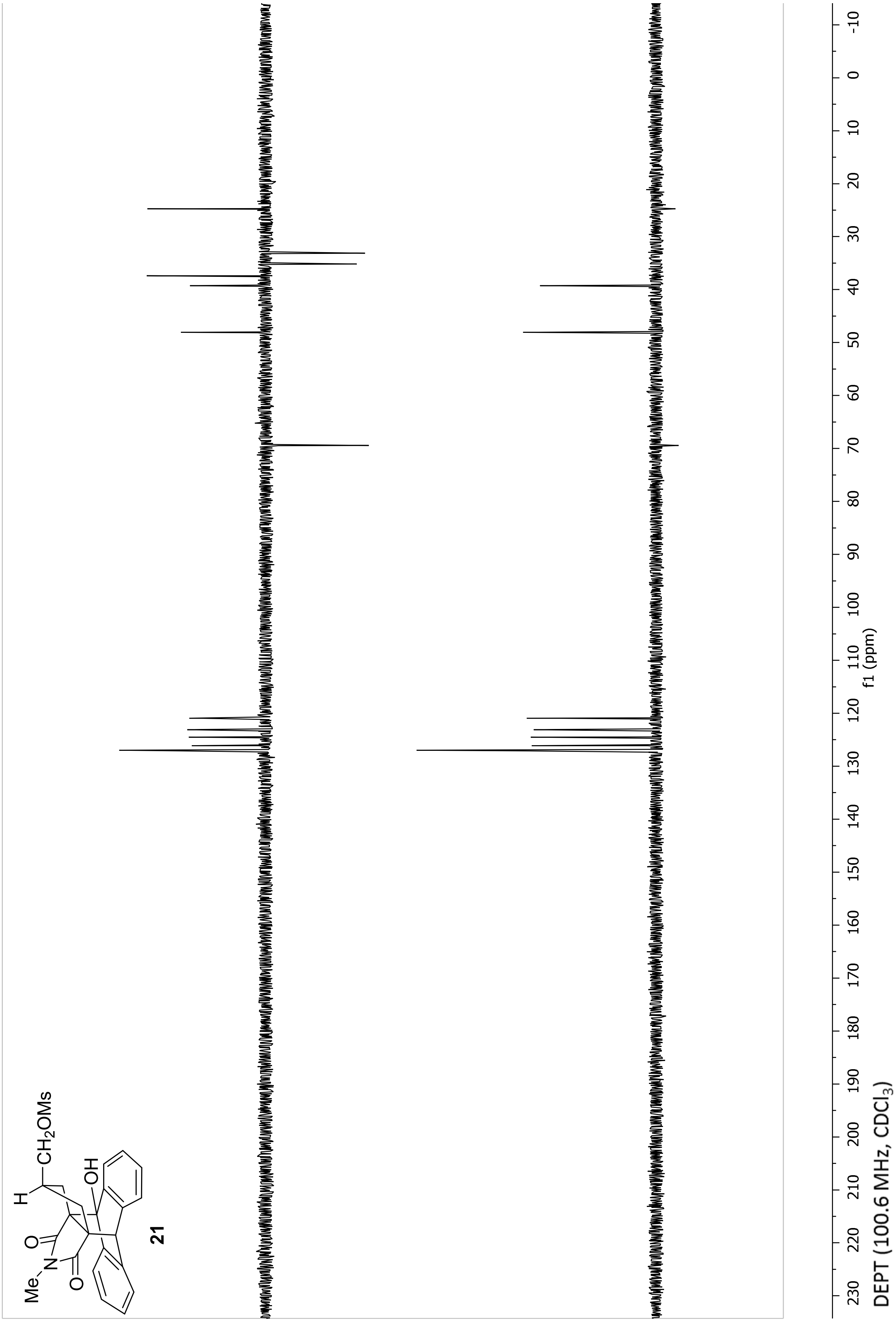

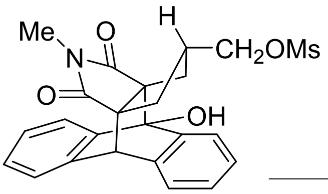

21

NMR and IR spectra of compound **21**

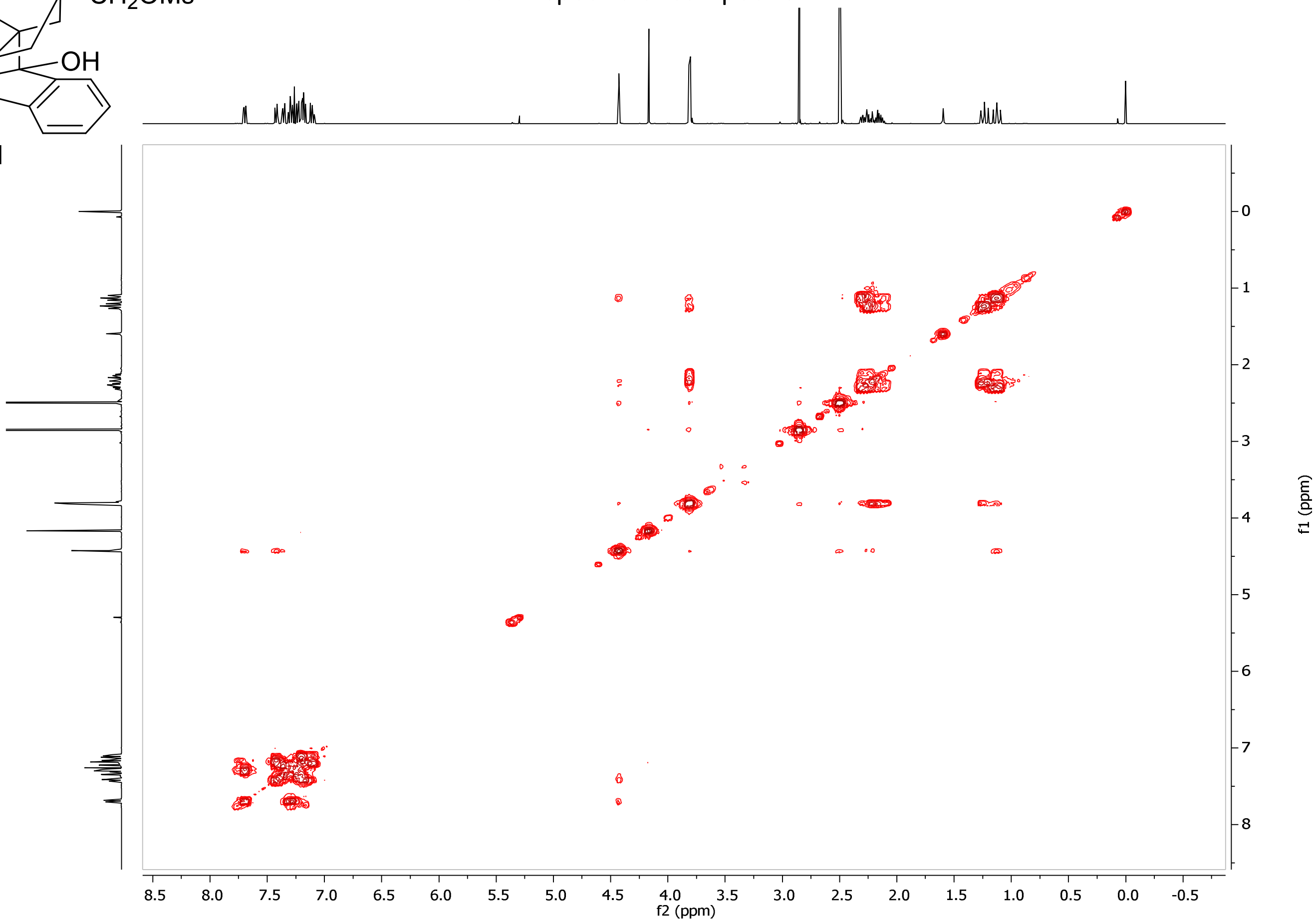

^{1}H/^{1}H COSY (400 MHz, $CDCl_3$)

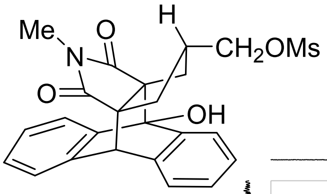

21

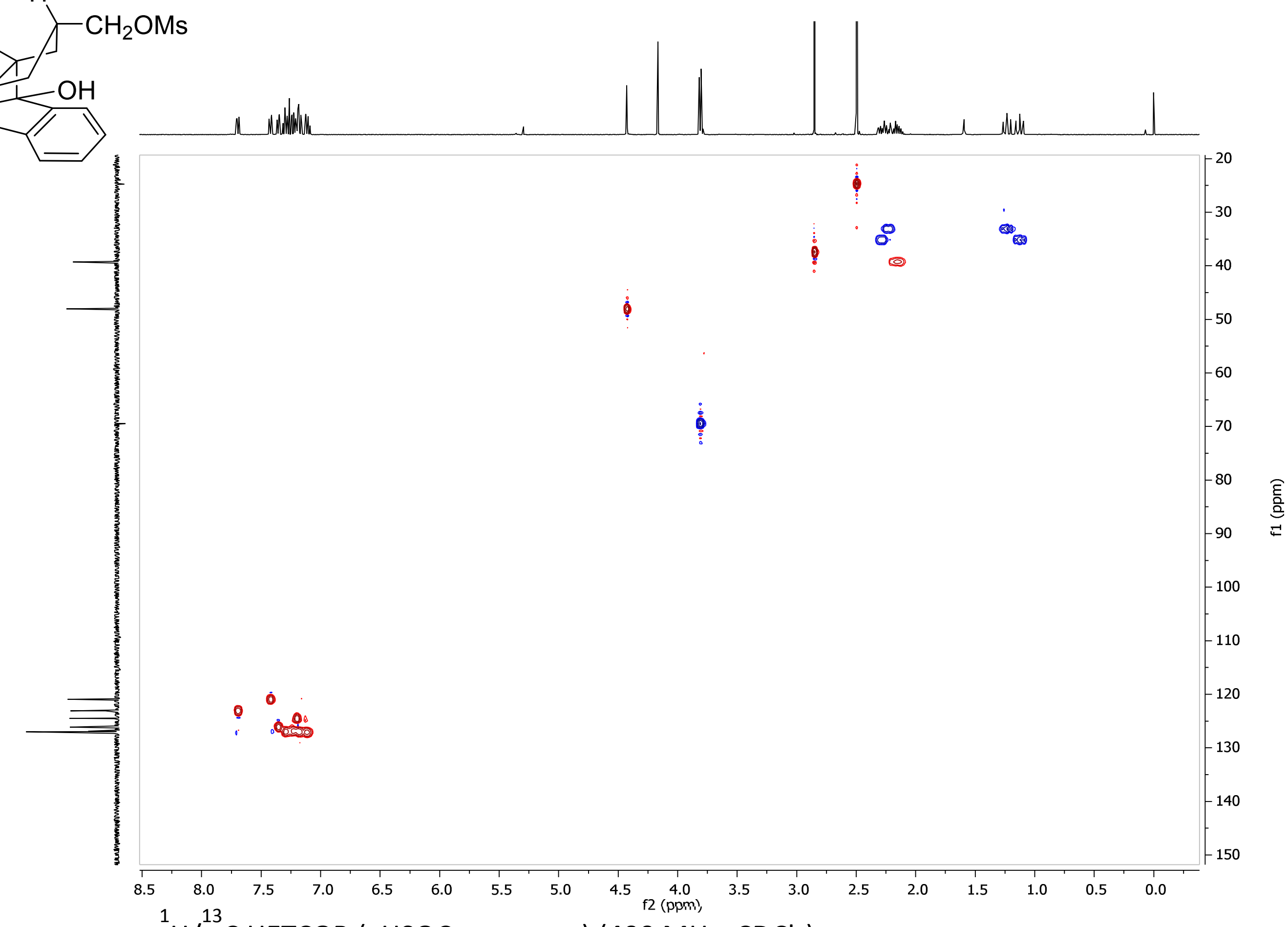

^{1}H/^{13}C HETCOR (gHSQC sequence) (400 MHz, $CDCl_3$)

NMR and IR spectra of compound **21**

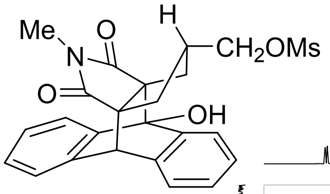

21

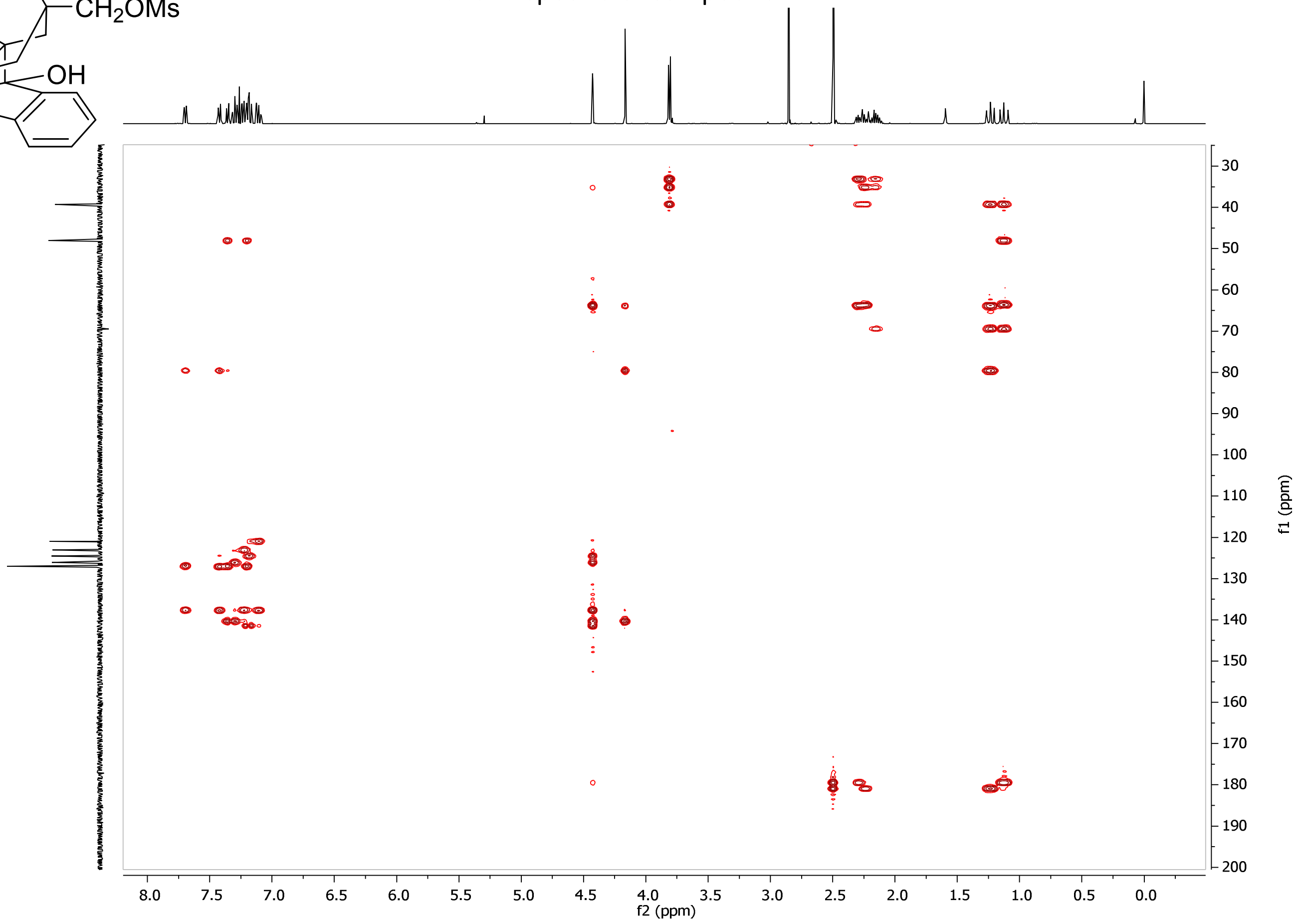

$^1H/^{13}C$ HETCOR (gHMBC sequence) (400 MHz, $CDCl_3$)

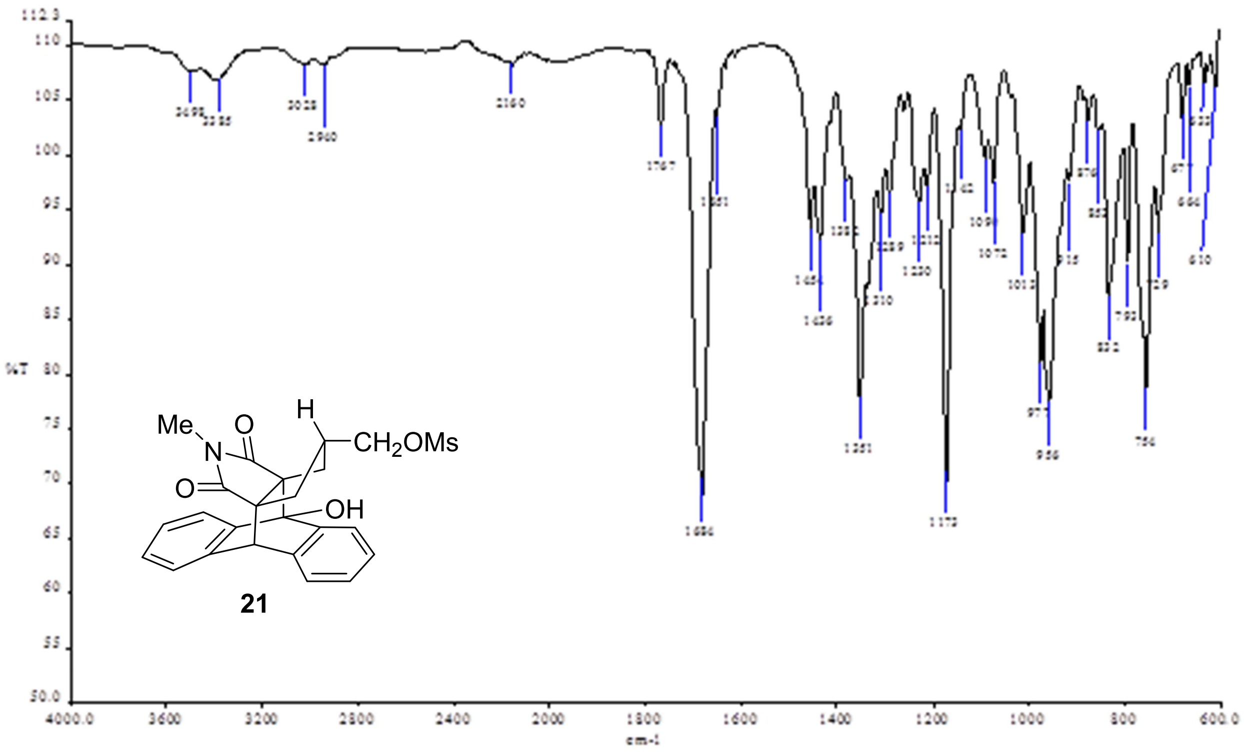

IR (ATR)

NMR and IR spectra of compound **23**

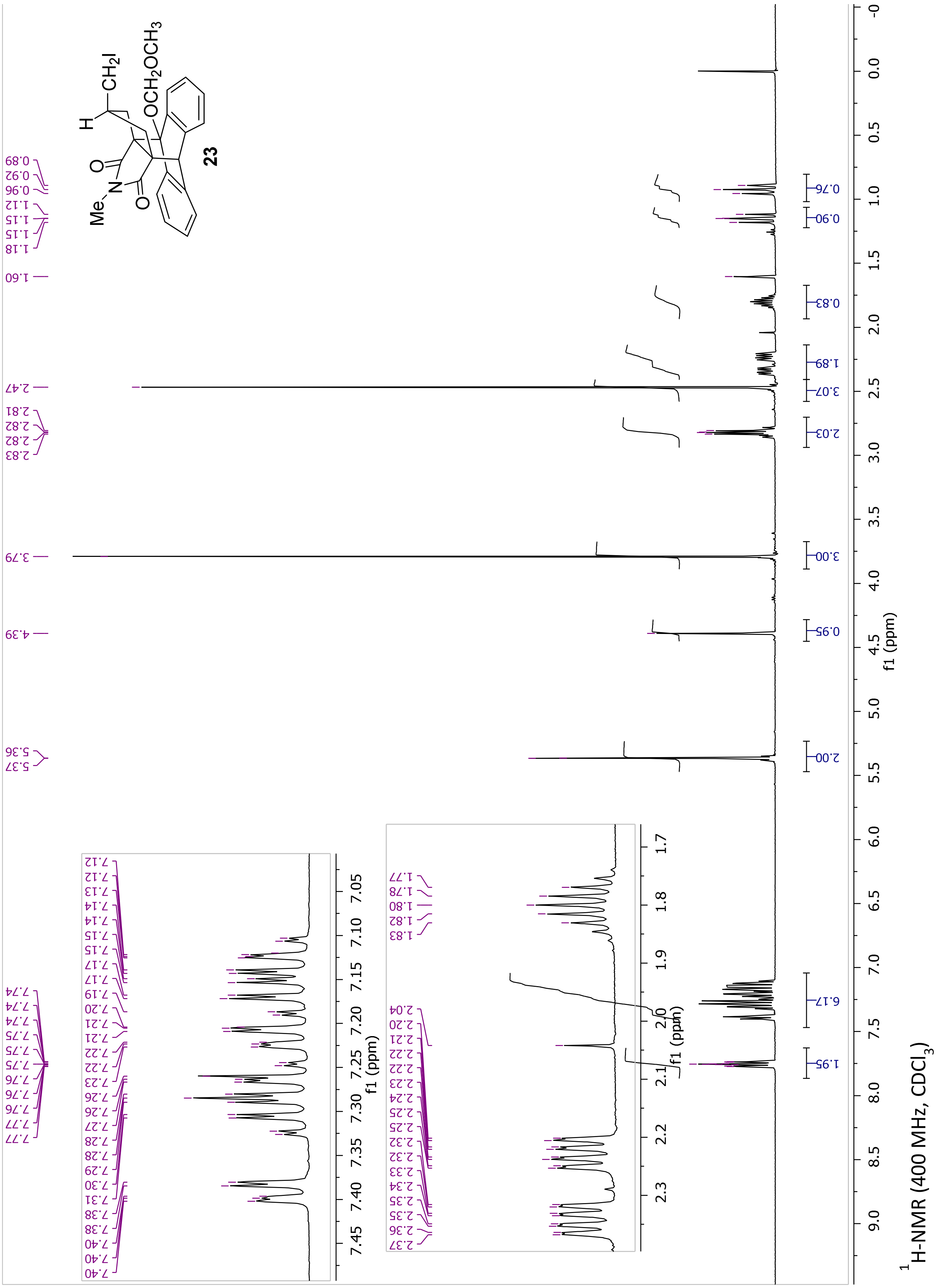

NMR and IR spectra of compound **23**

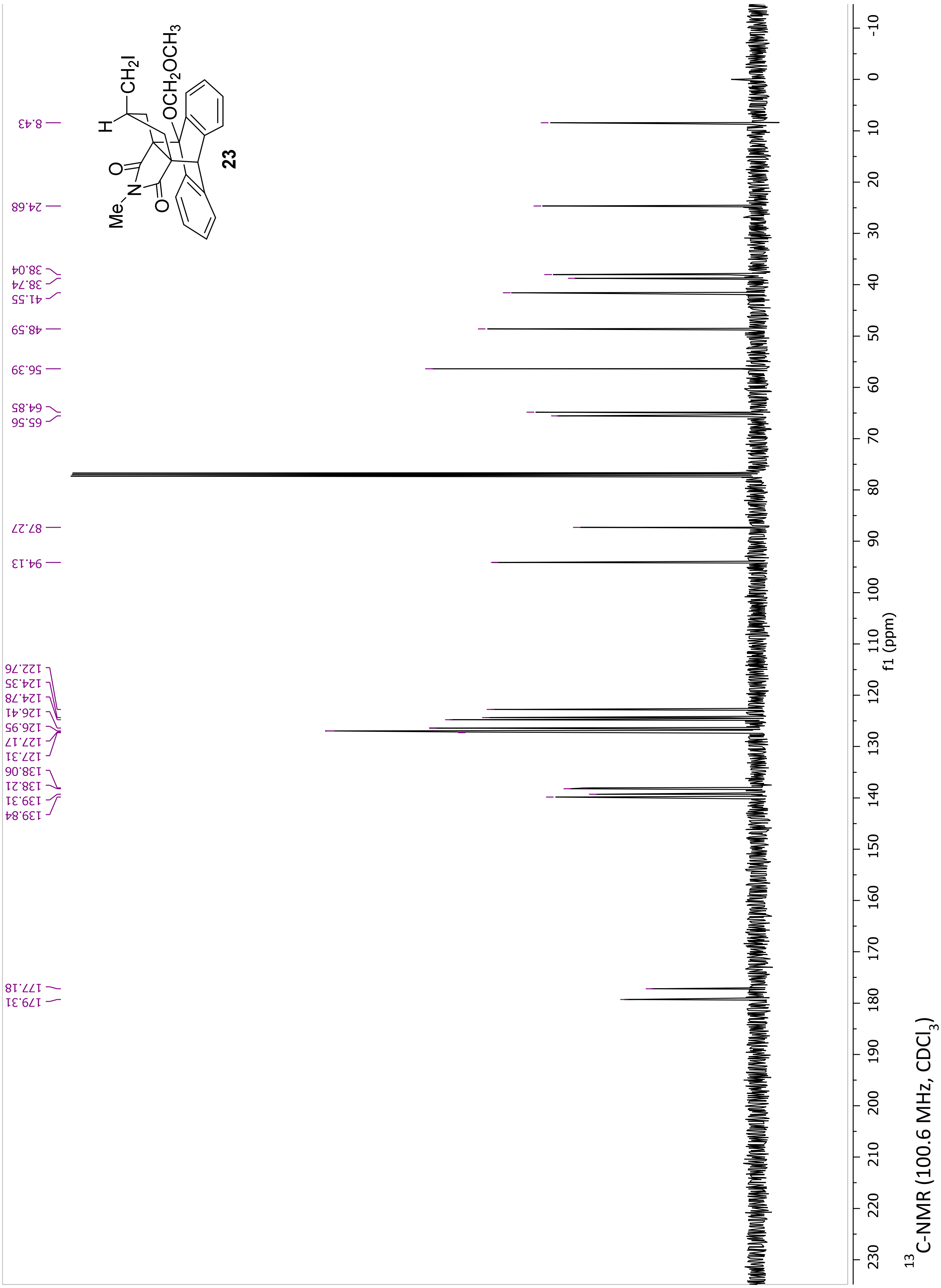

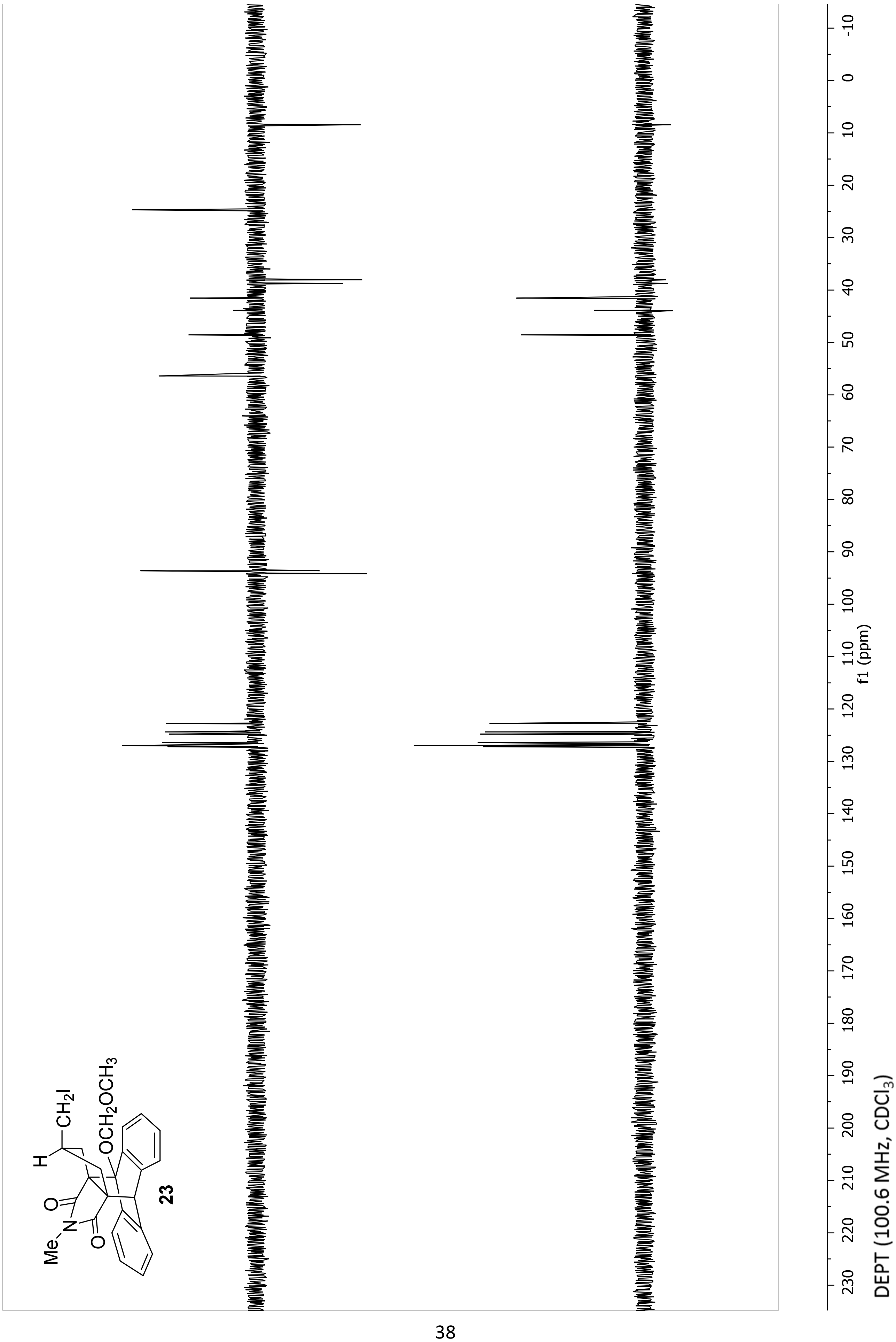
DEPT (100.6 MHz, CDCl3)
f1 (ppm)
23
Me
N
O
O
H
CH2I
OCH2OCH3

NMR and IR spectra of compound **23**

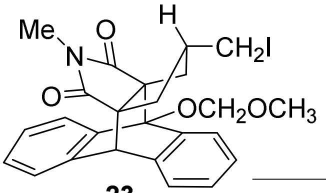

23

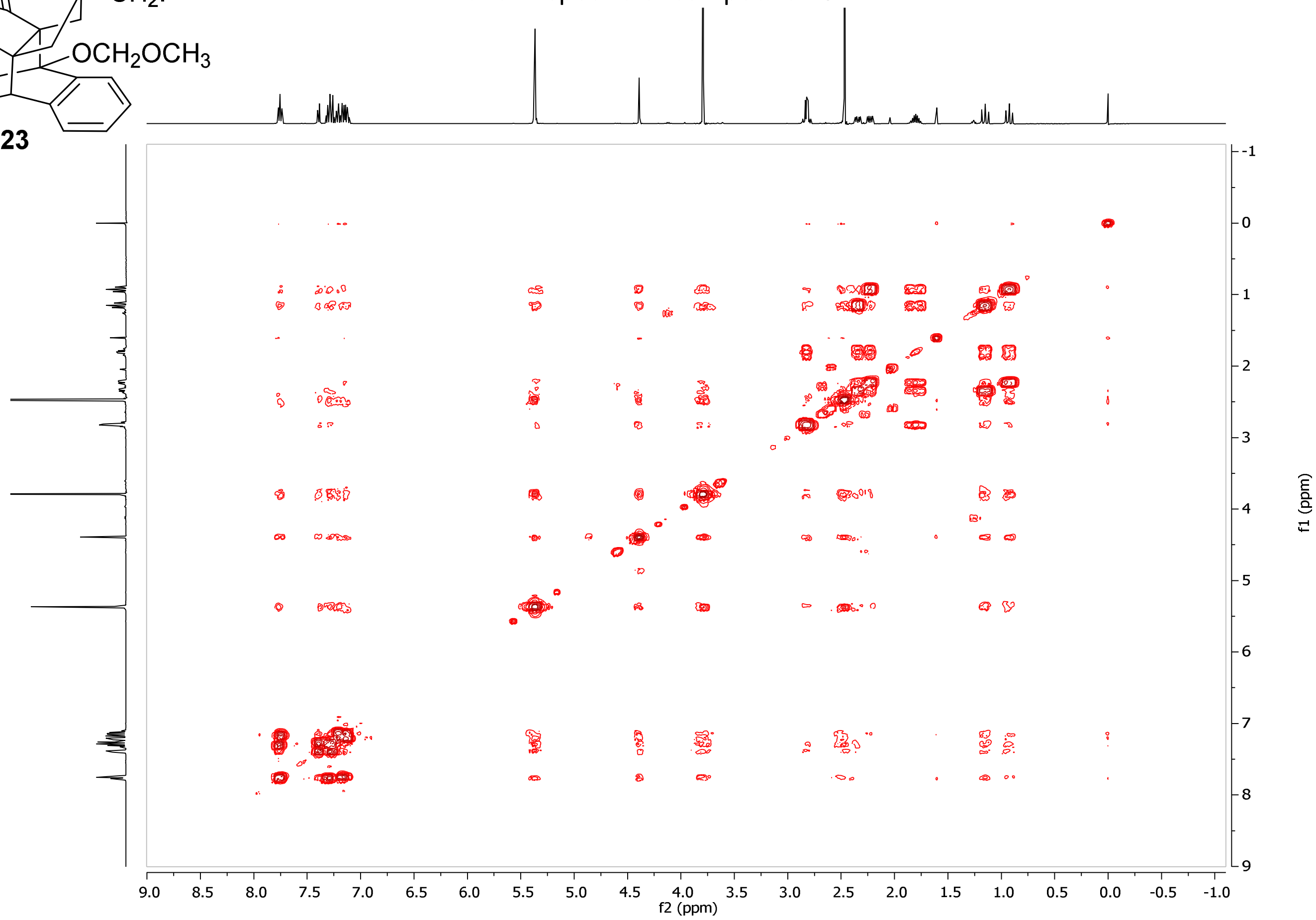

$^{1}H/^{1}H$ COSY (400 MHz, $CDCl_3$)

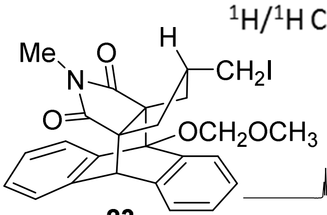

23

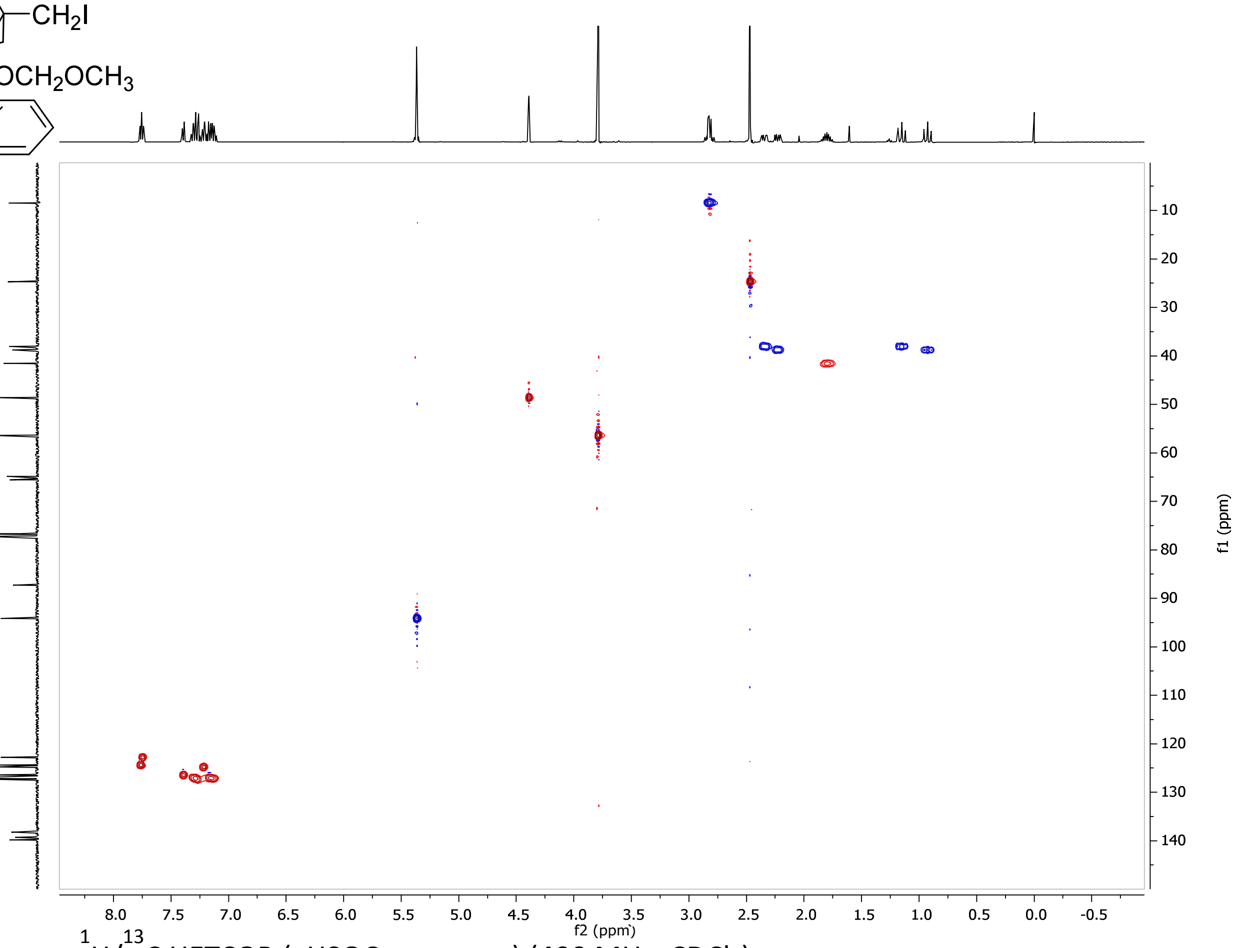

$^{1}H/^{13}C$ HETCOR (gHSQC sequence) (400 MHz, $CDCl_3$)

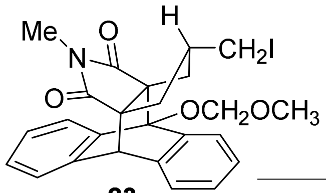

23

NMR and IR spectra of compound **23**

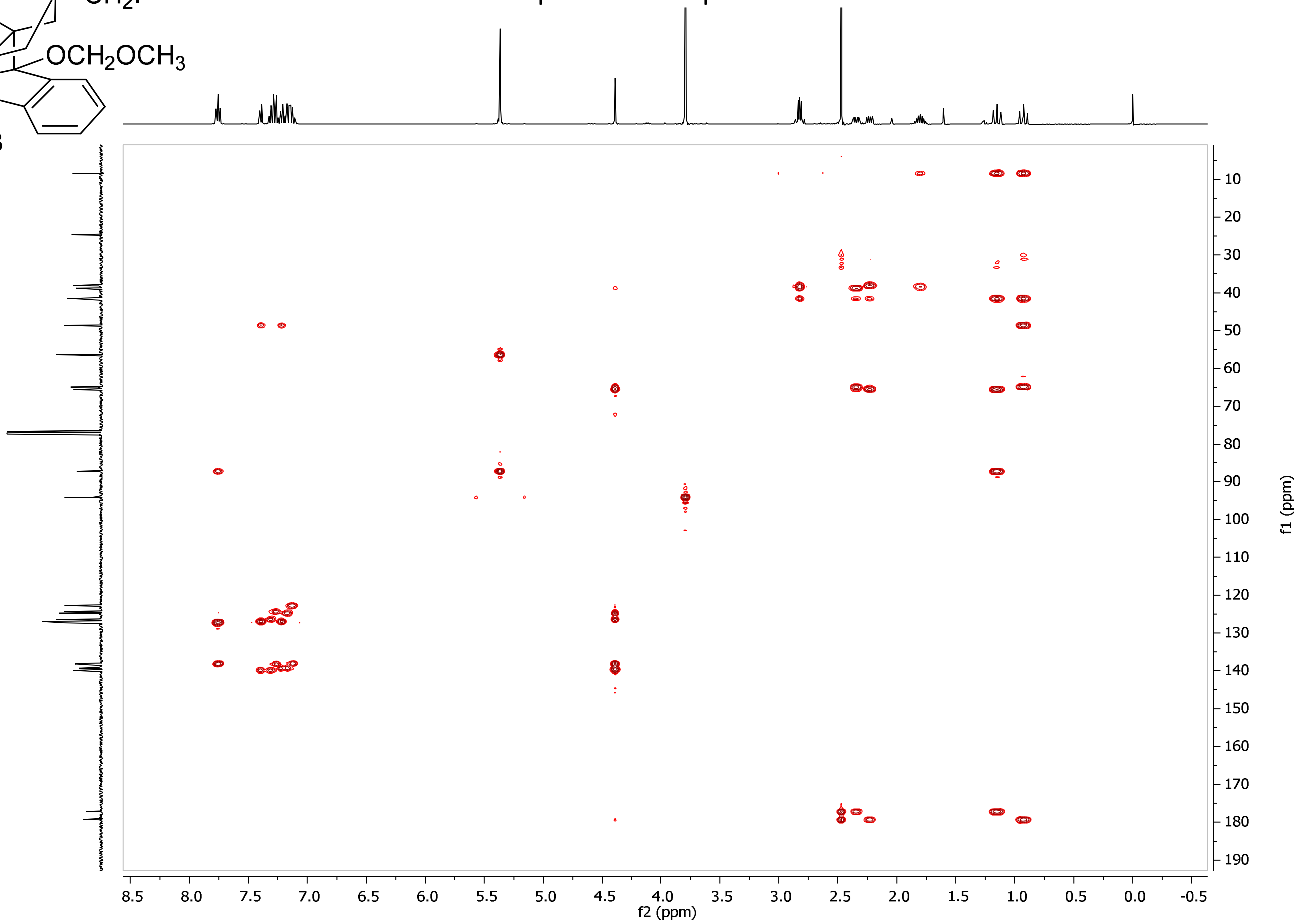

$^1H/^{13}C$ HETCOR (gHMBC sequence) (400 MHz, $CDCl_3$)

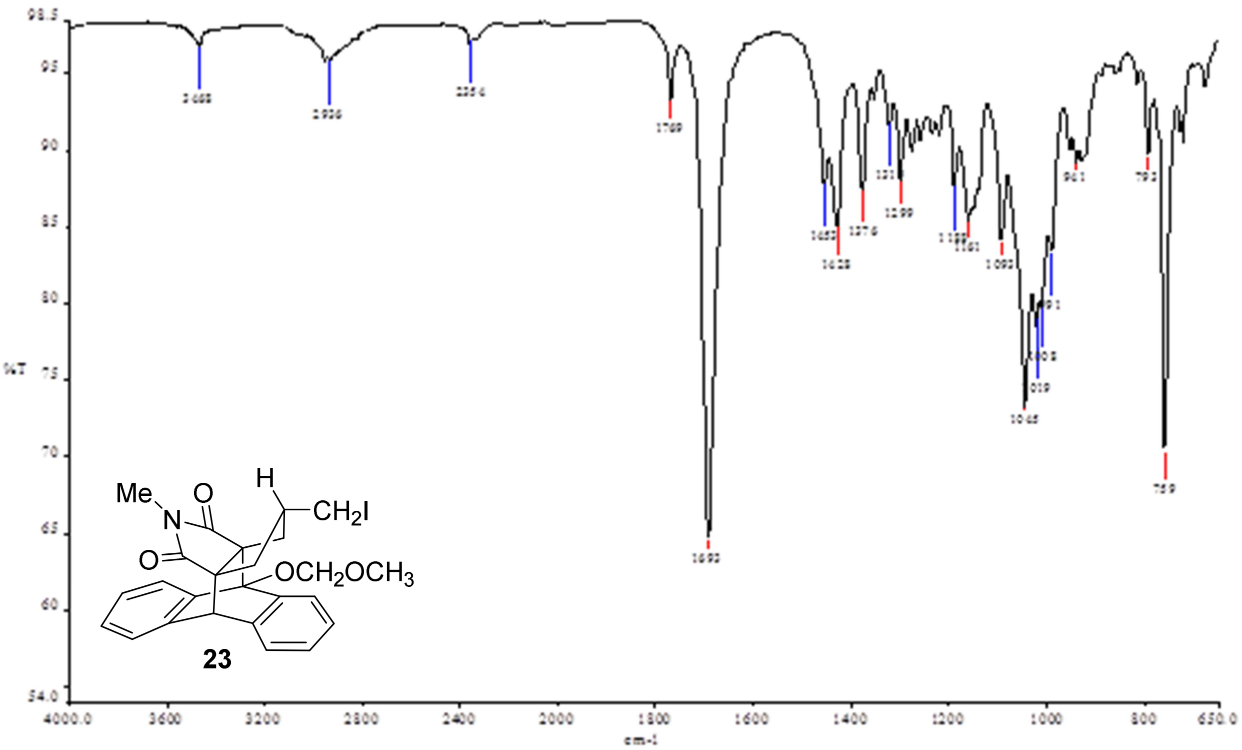

IR (ATR)

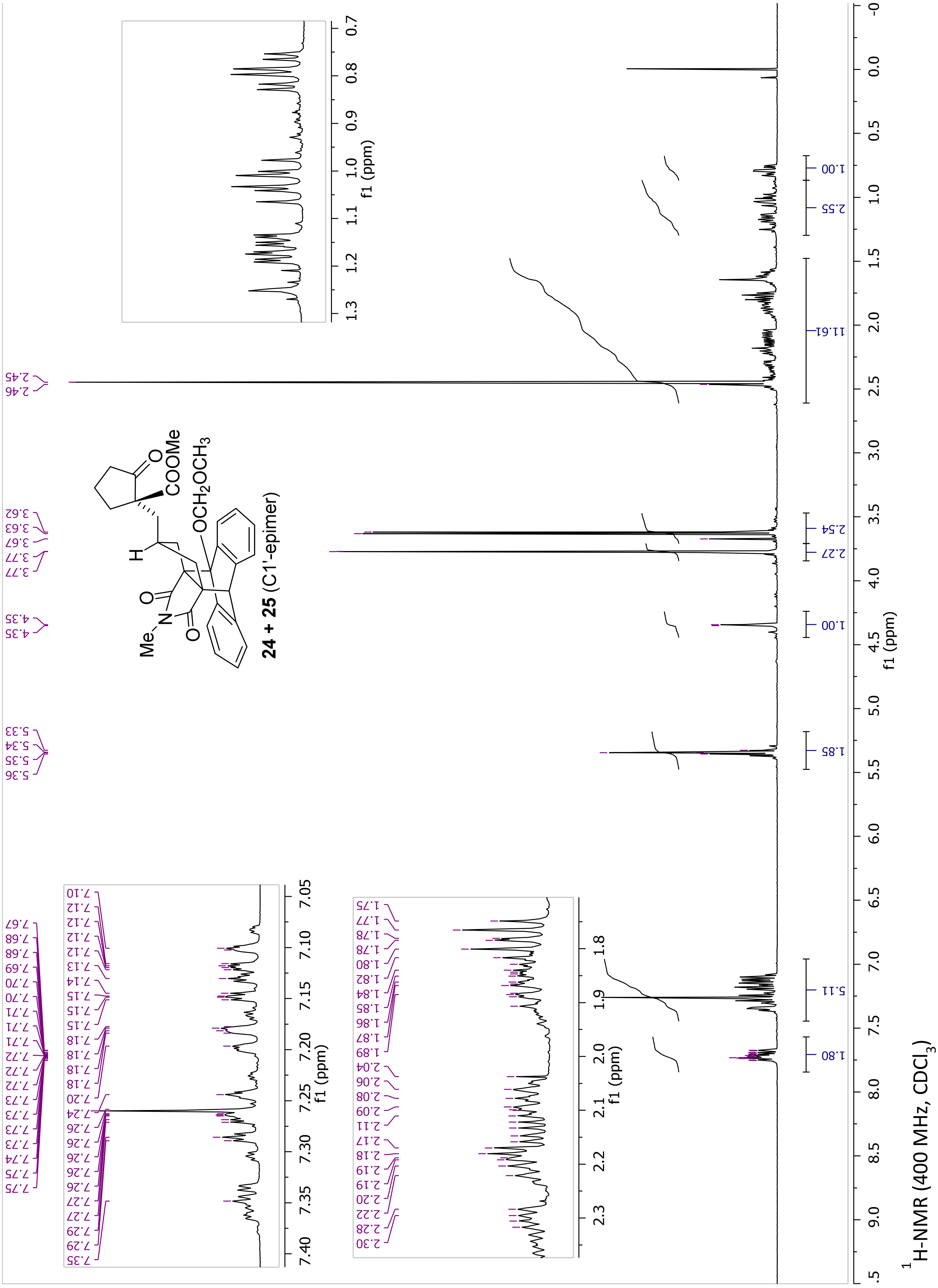
24 + 25 (C1'-epimer)
f1 (ppm)
1H-NMR (400 MHz, CDCl3)

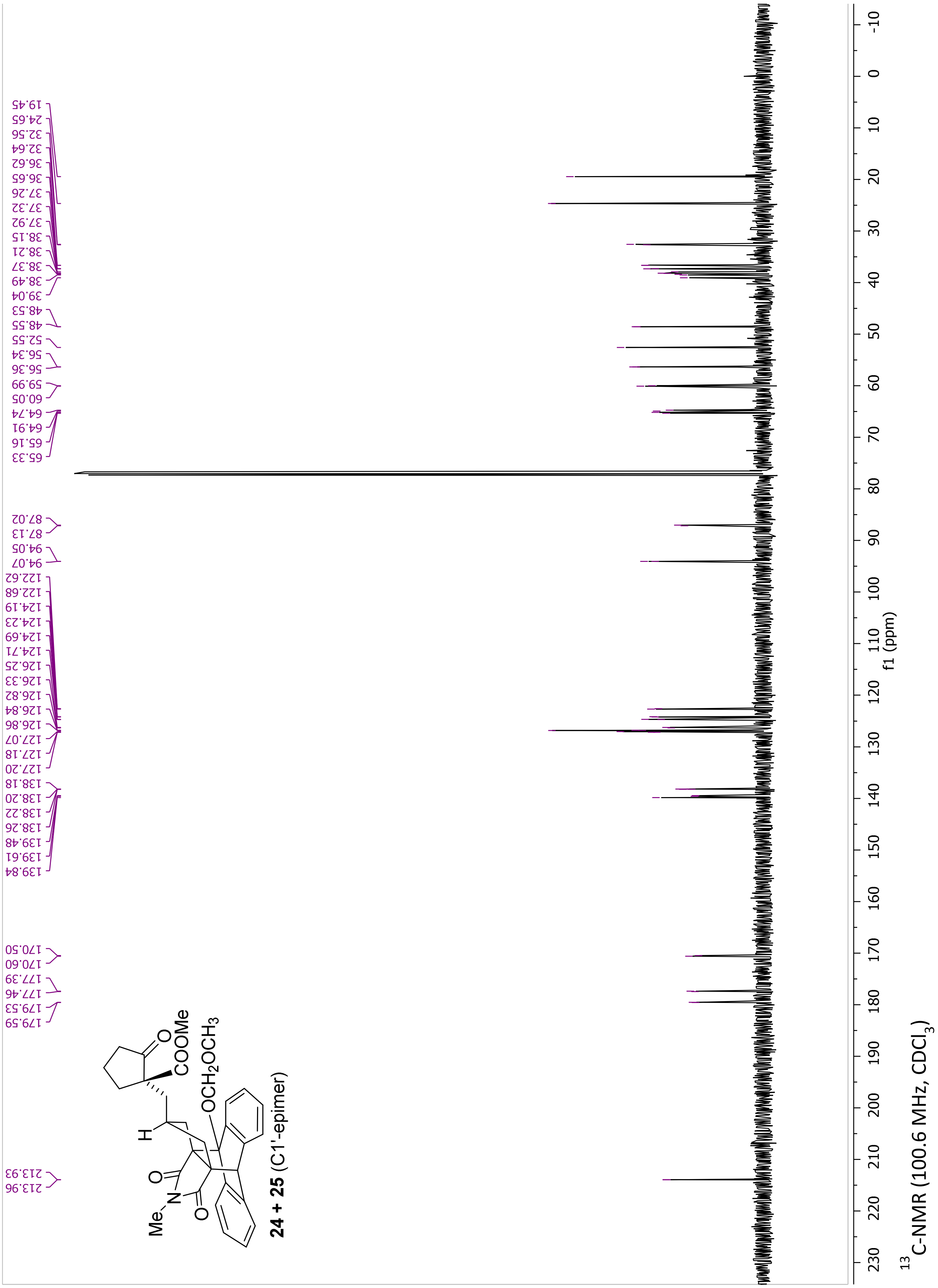
19.45
24.65
32.56
32.64
36.62
36.65
37.26
37.32
37.92
38.15
38.21
38.37
38.49
39.04
48.53
48.55
52.55
56.34
56.36
59.99
60.05
64.74
64.91
65.16
65.33
87.02
87.13
94.05
94.07
122.62
122.68
124.19
124.23
124.69
124.71
126.25
126.33
126.82
126.84
126.86
127.07
127.18
127.20
138.18
138.20
138.22
138.26
139.48
139.61
139.84
170.50
170.60
177.39
177.46
179.53
179.59
213.93
213.96
COOMe
OCH_2OCH_3
H
O
Me
N
O
O
24 + 25 (C1'-epimer)
f1 (ppm)
^{13}C-NMR (100.6 MHz, $CDCl_3$)

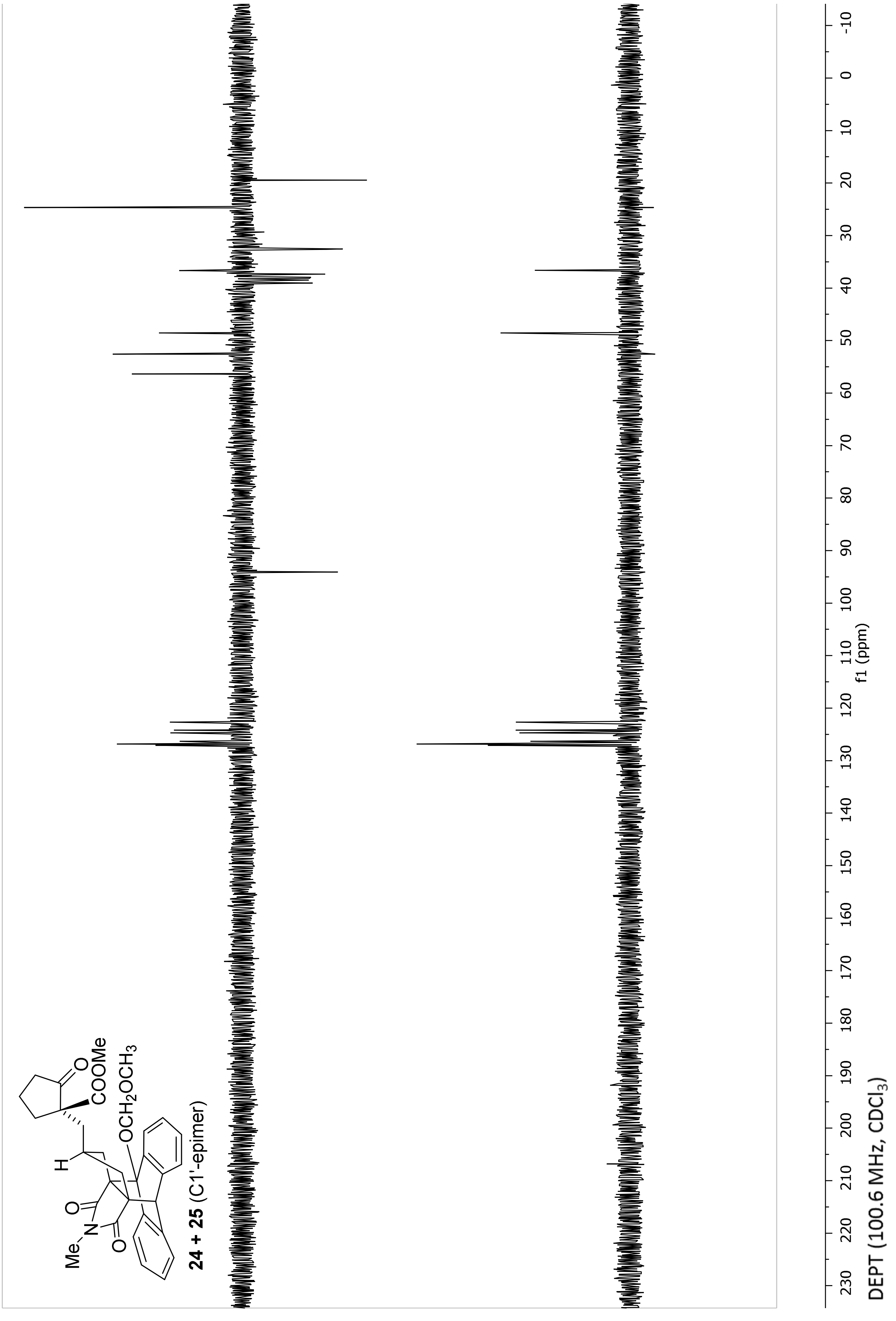
24 + 25 (C1'-epimer)
f1 (ppm)
DEPT (100.6 MHz, CDCl3)

NMR and IR spectra of compoounds **24** and **25**

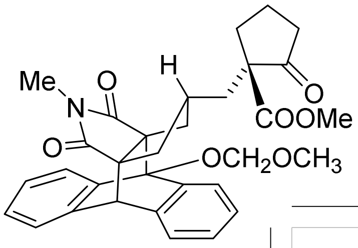

24 + 25 (C1'-epimer)

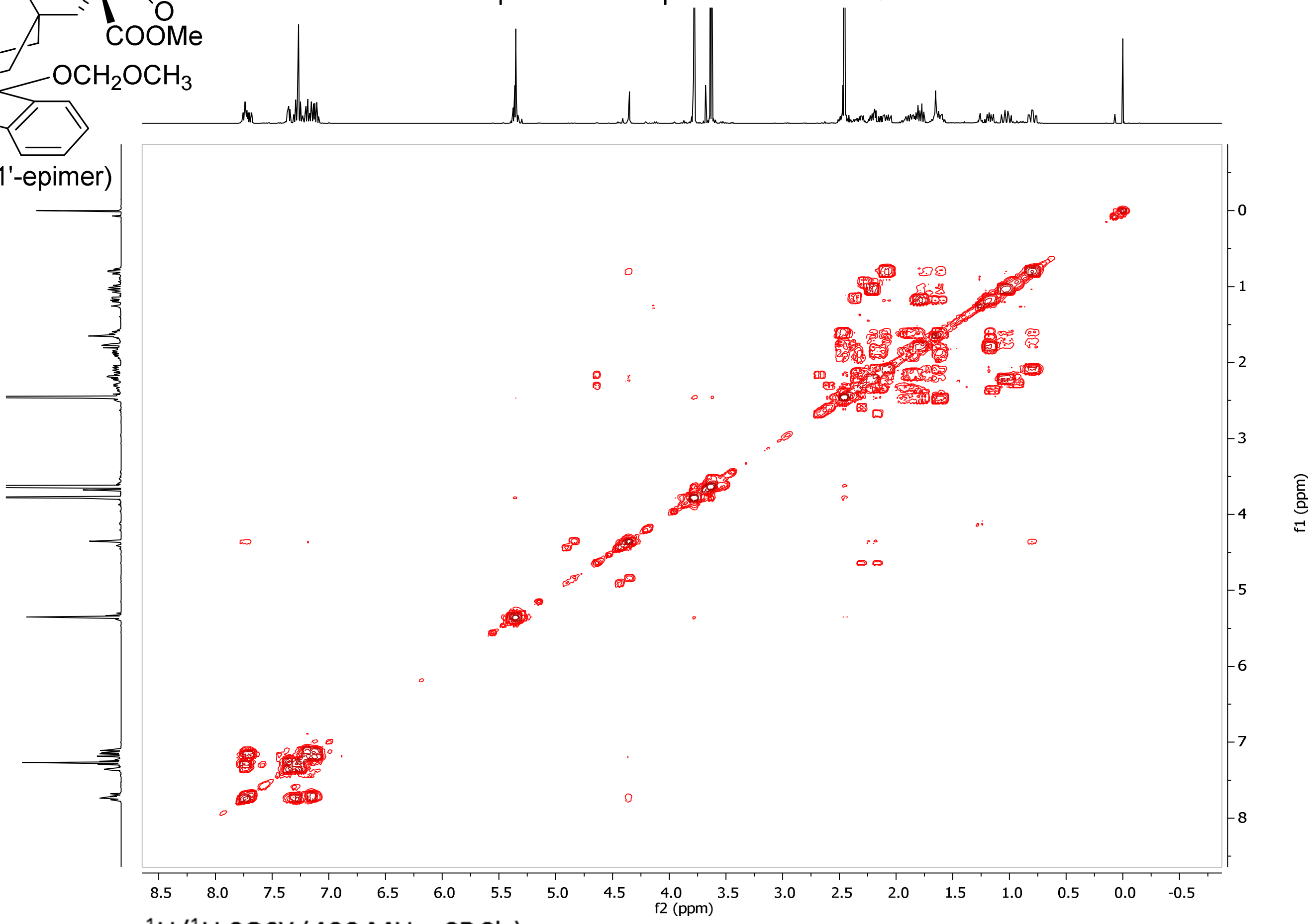

$^1H/^1H$ COSY (400 MHz, $CDCl_3$)

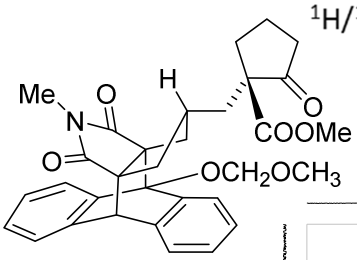

24 + 25 (C1'-epimer)

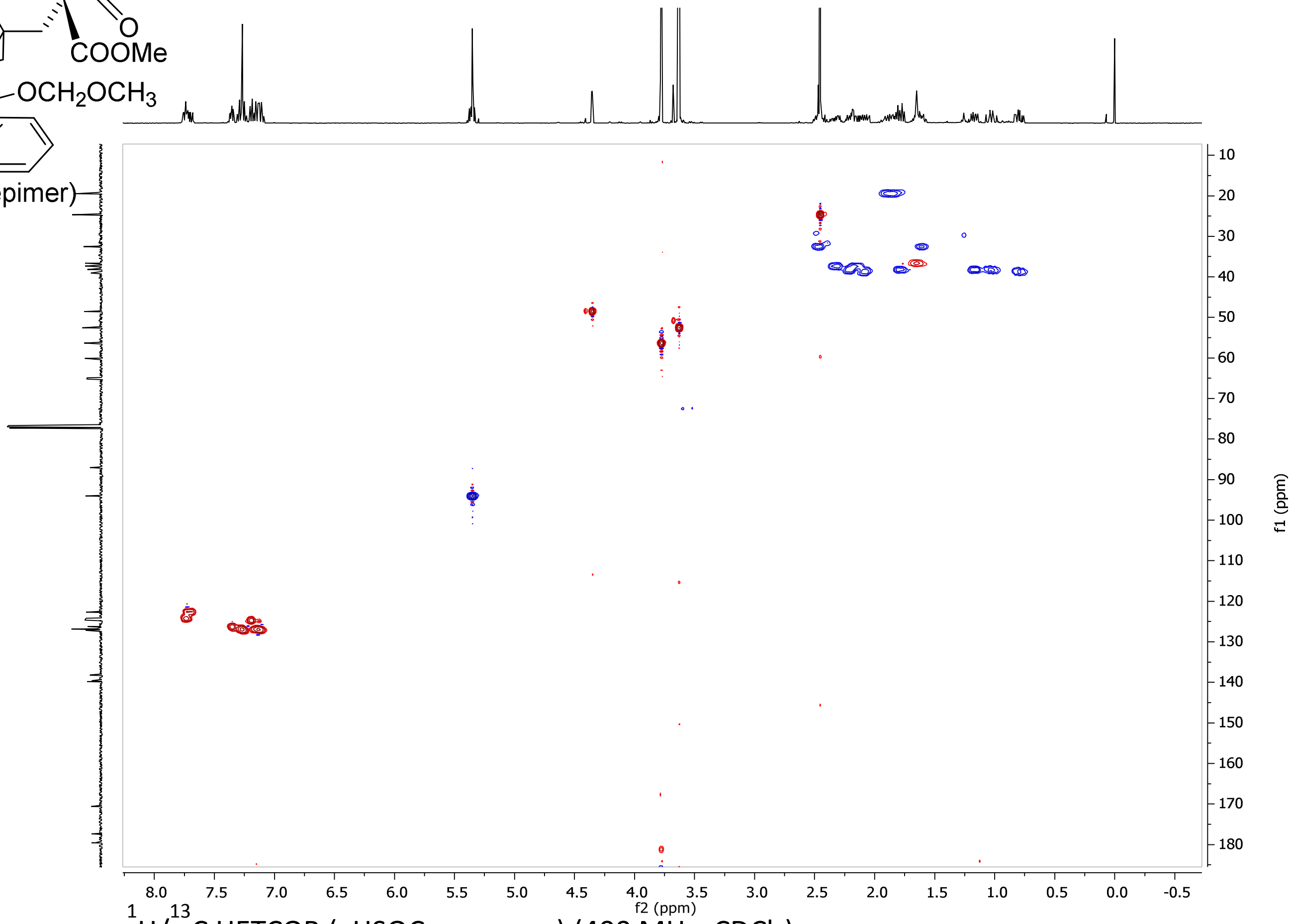

$^1H/^{13}C$ HETCOR (gHSQC sequence) (400 MHz, $CDCl_3$)

NMR and IR spectra of compoounds **24** and **25**

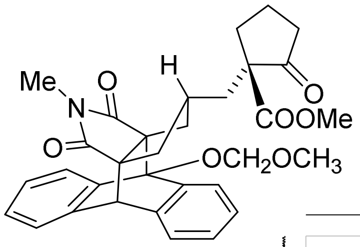

24 + 25 (C1'-epimer)

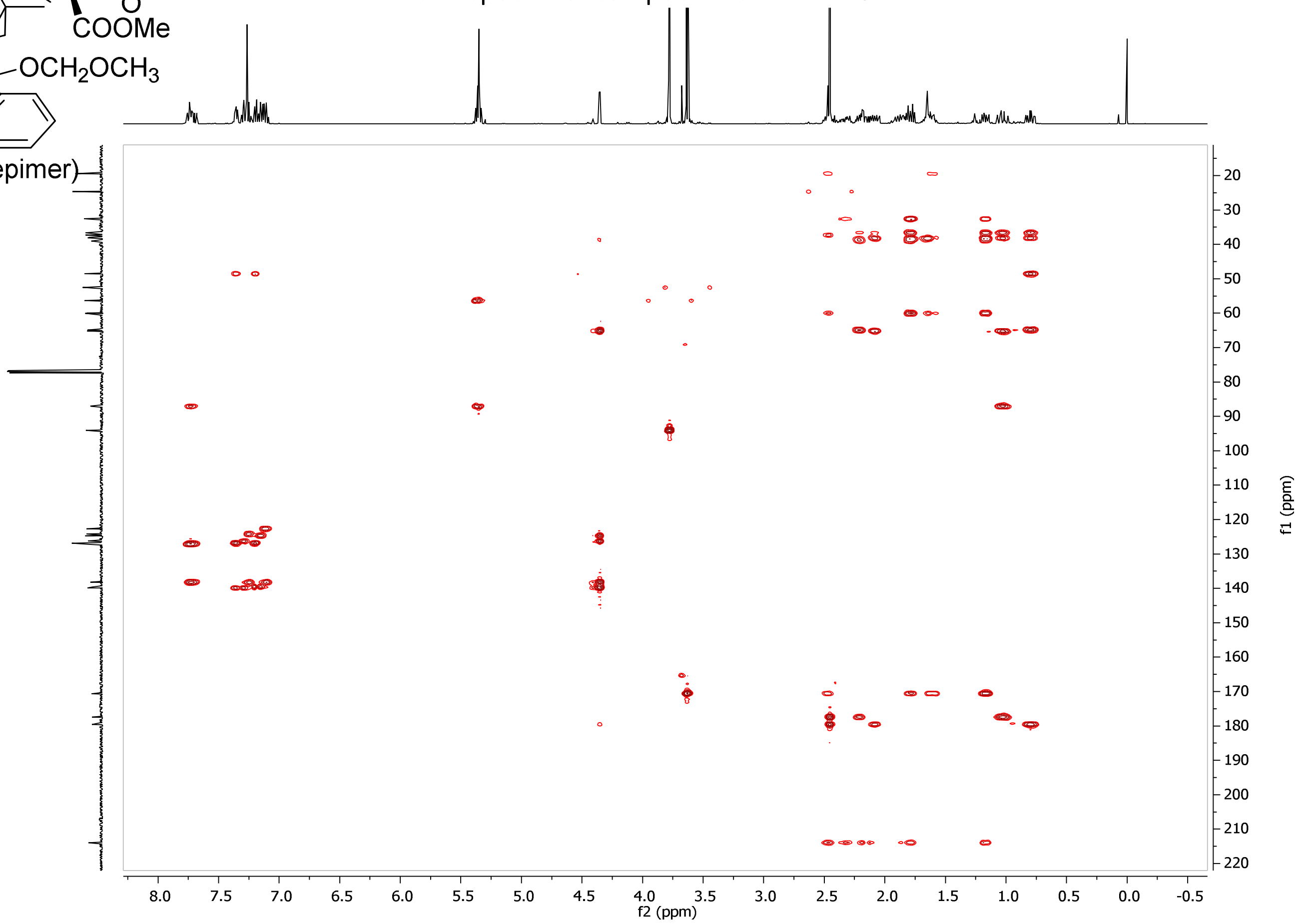

$^{1}H/^{13}C$ HETCOR (gHMBC sequence) (400 MHz, $CDCl_3$)

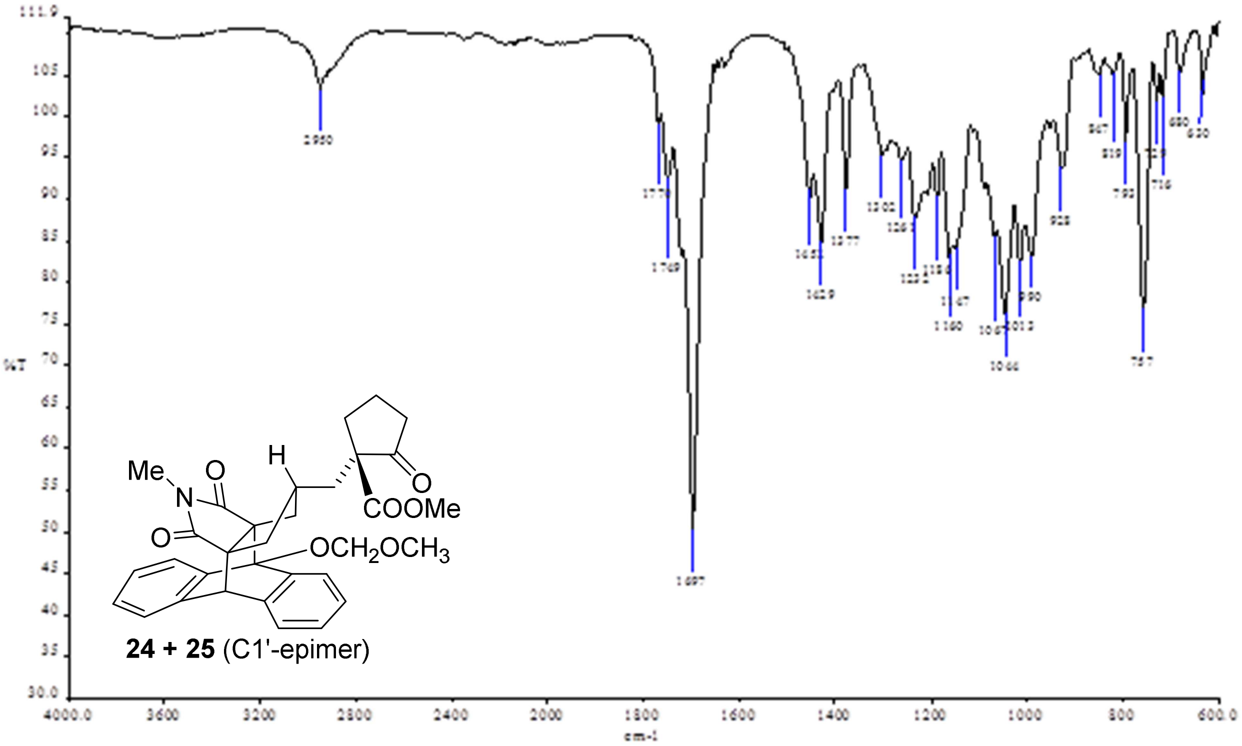

IR (ATR)

NMR and IR spectra of compound **24**

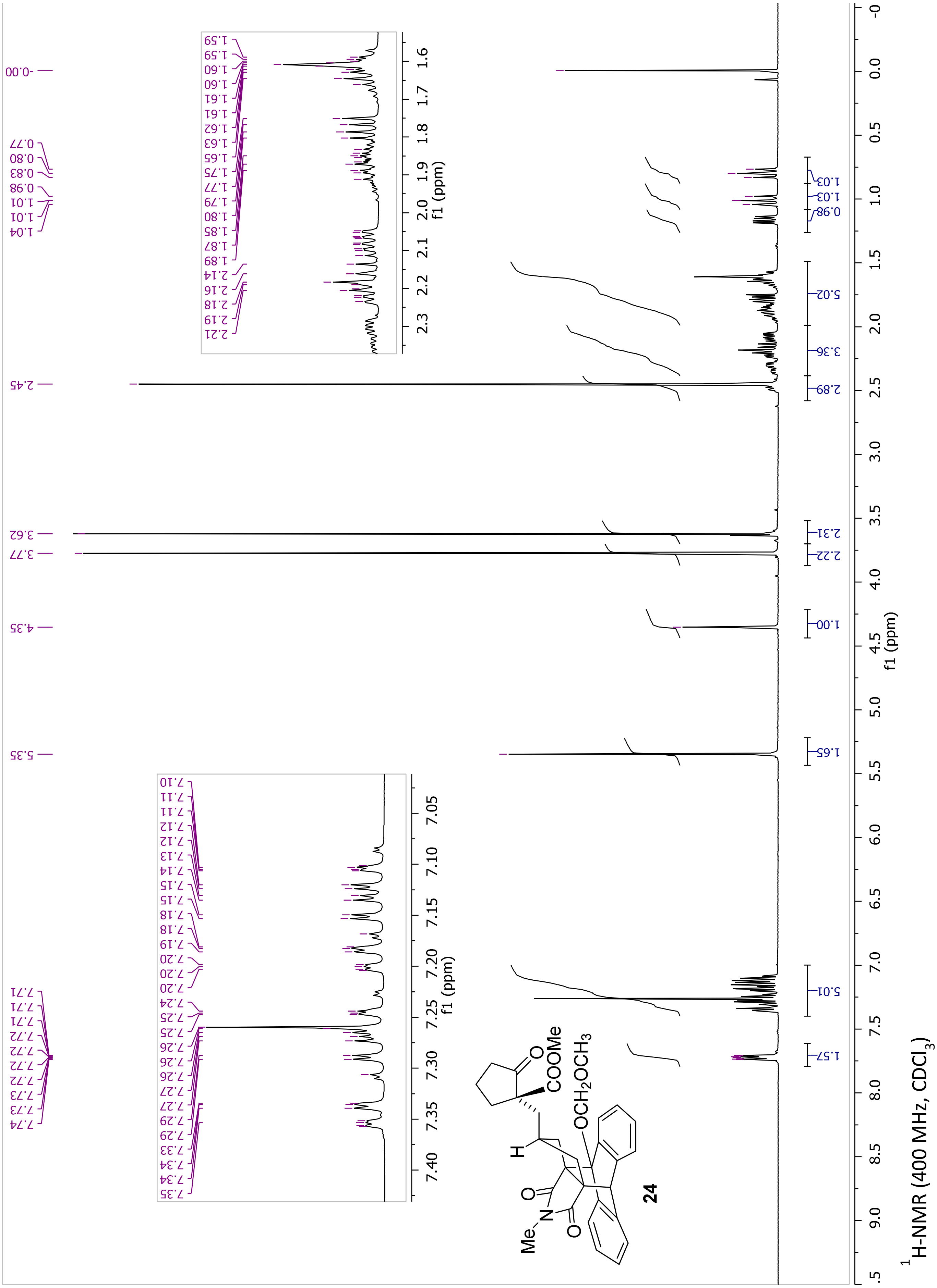

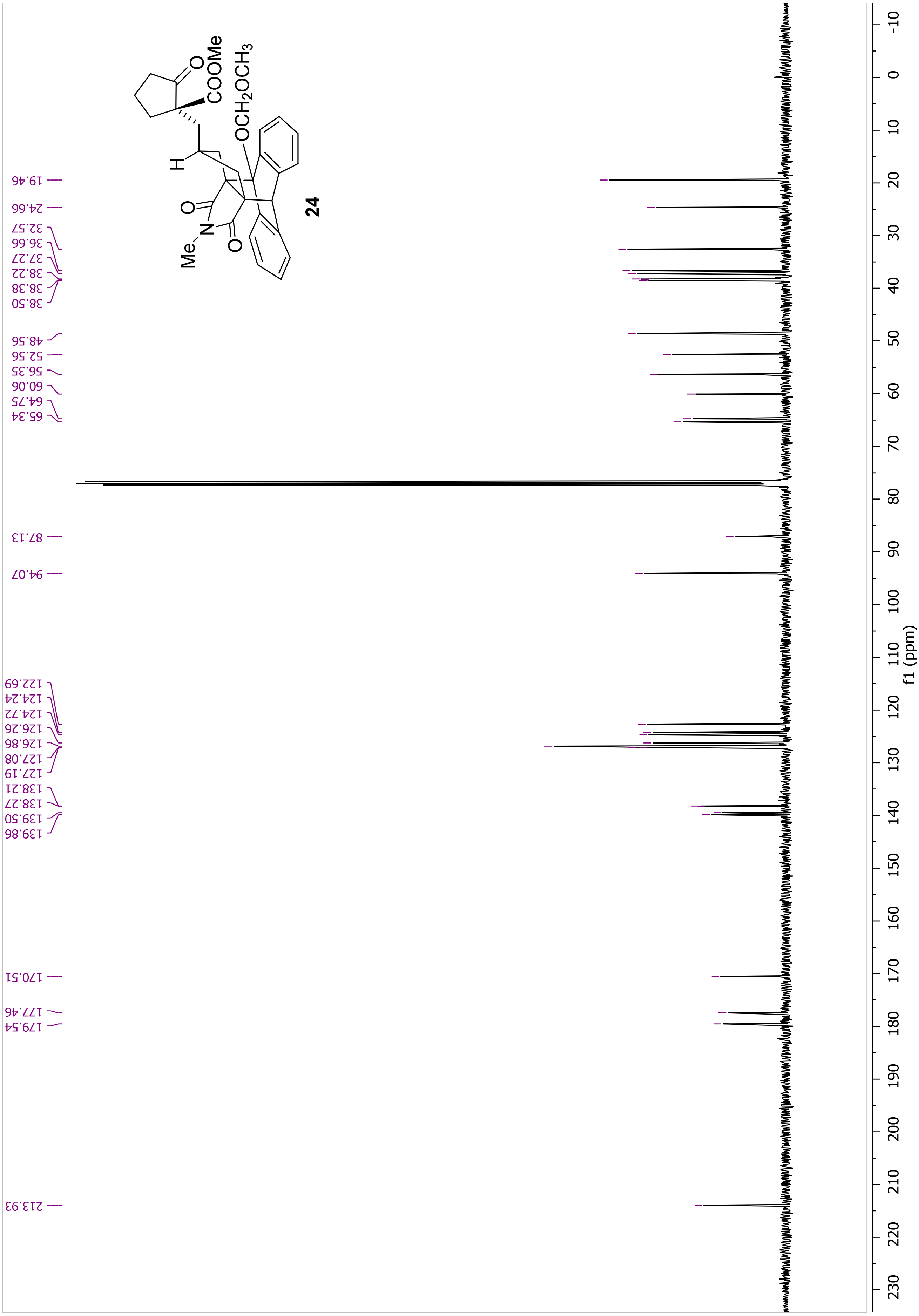

^{13}C-NMR (100.6 MHz, $CDCl_3$)

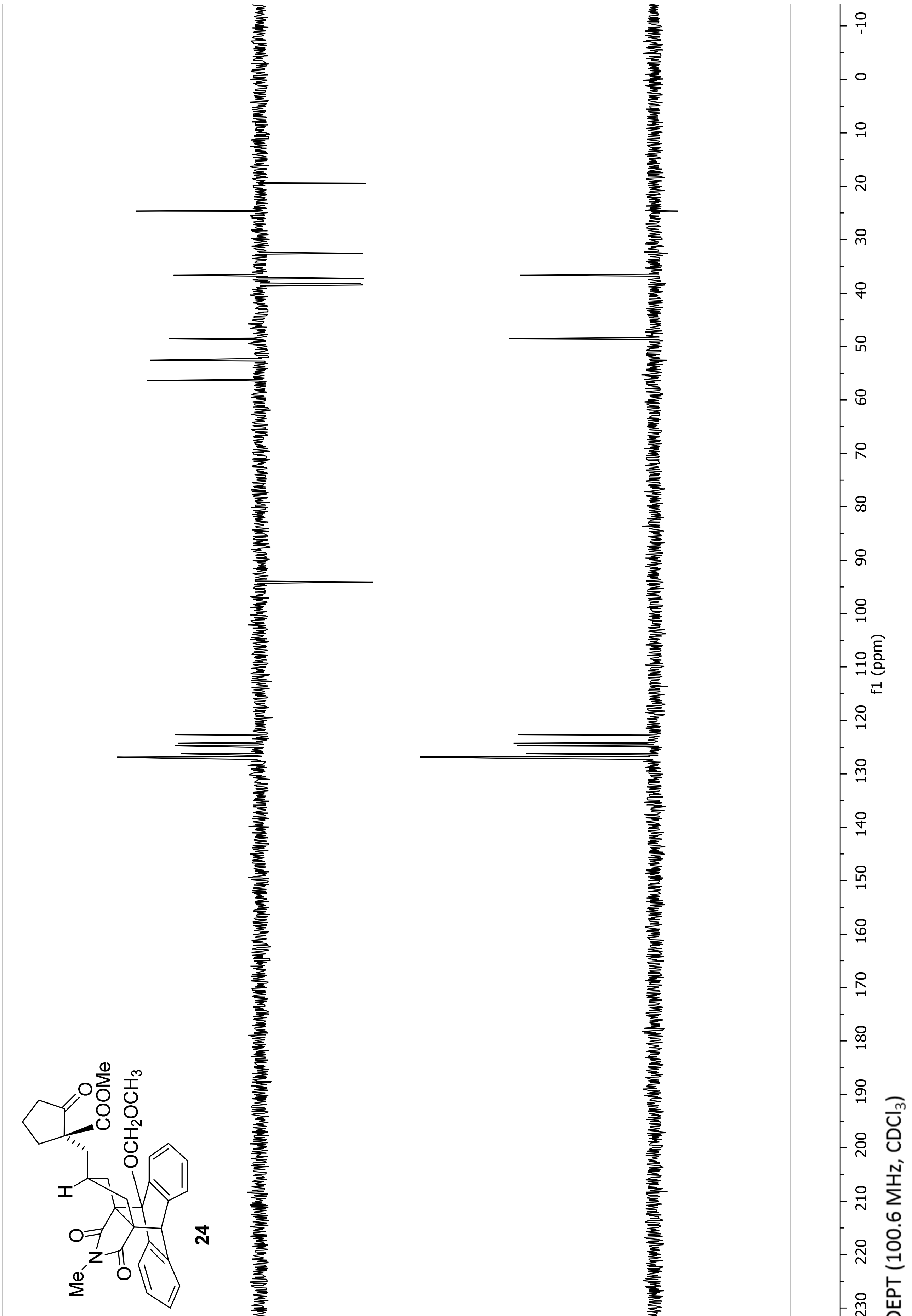
COOMe
OCH2OCH3
H
O
N
Me
O
24
f1 (ppm)
DEPT (100.6 MHz, CDCl3)

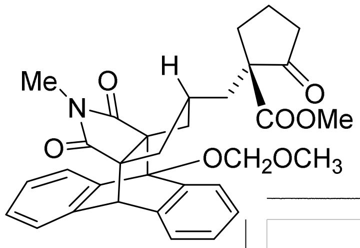

24

NMR and IR spectra of compound **24**

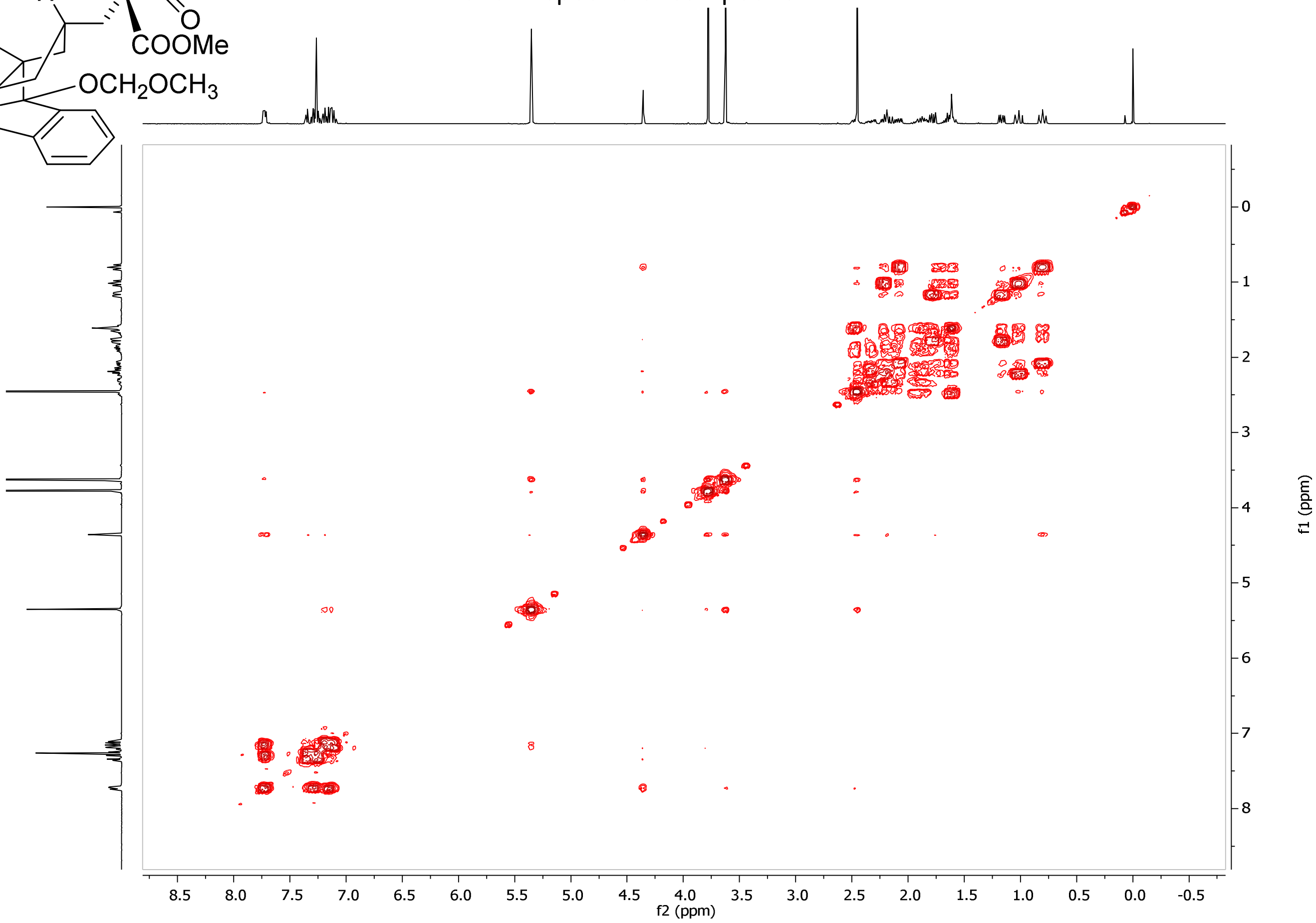

$^{1}H/^{1}H$ COSY (400 MHz, $CDCl_3$)

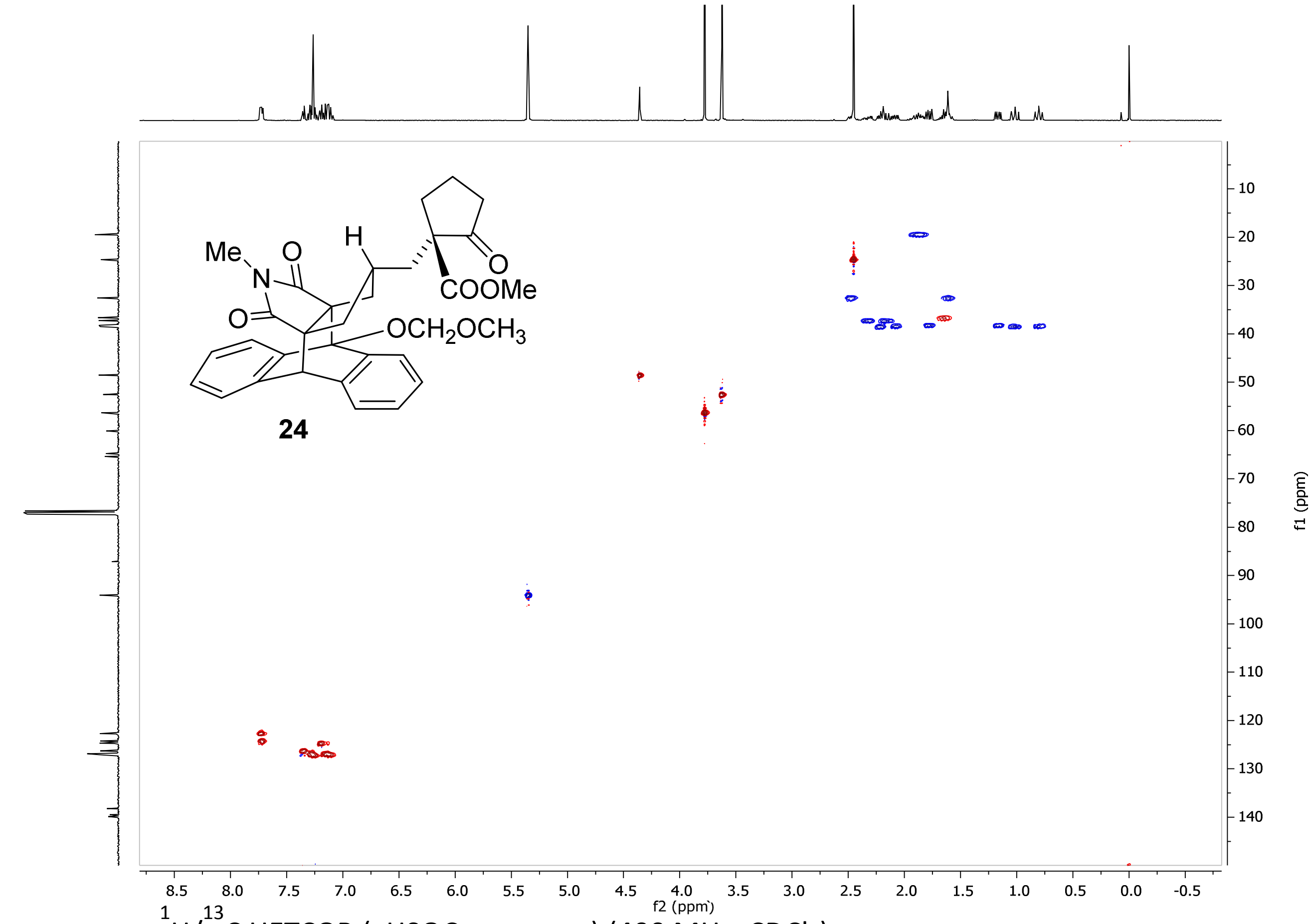

$^{1}H/^{13}C$ HETCOR (gHSQC sequence) (400 MHz, $CDCl_3$)

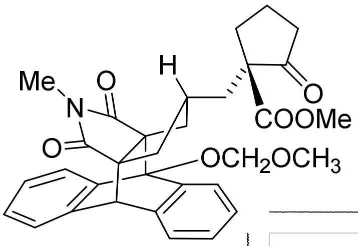

24

NMR and IR spectra of compound **24**

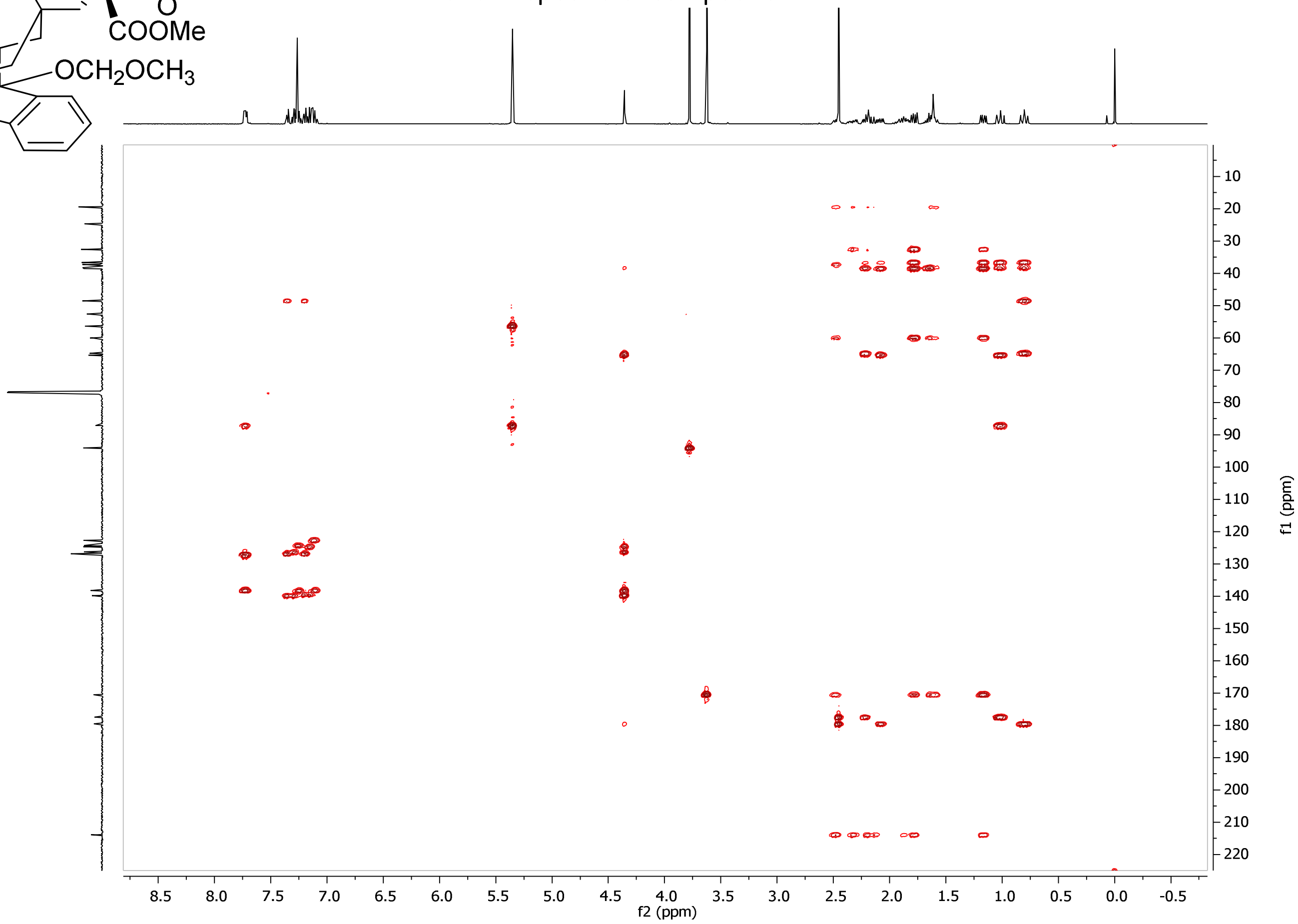

$^{1}H/^{13}C$ HETCOR (gHMBC sequence) (400 MHz, $CDCl_3$)

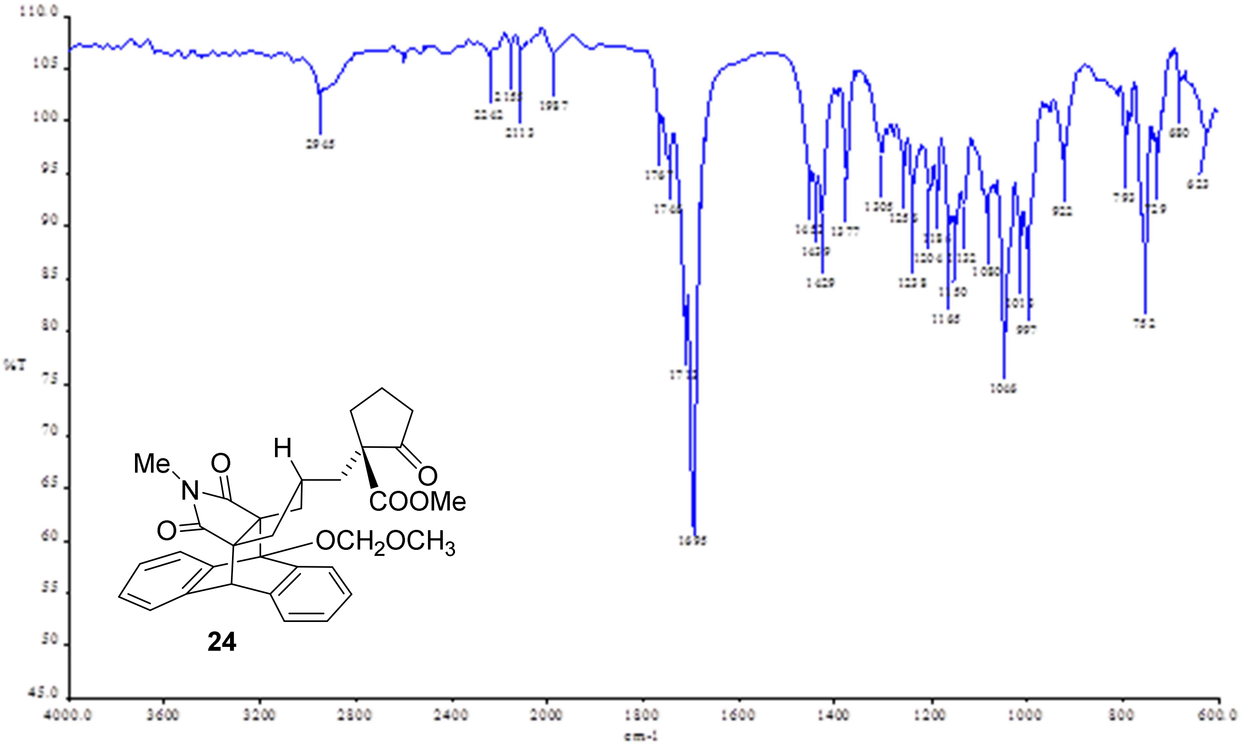

IR (ATR)

NMR and IR spectra of compound **26**

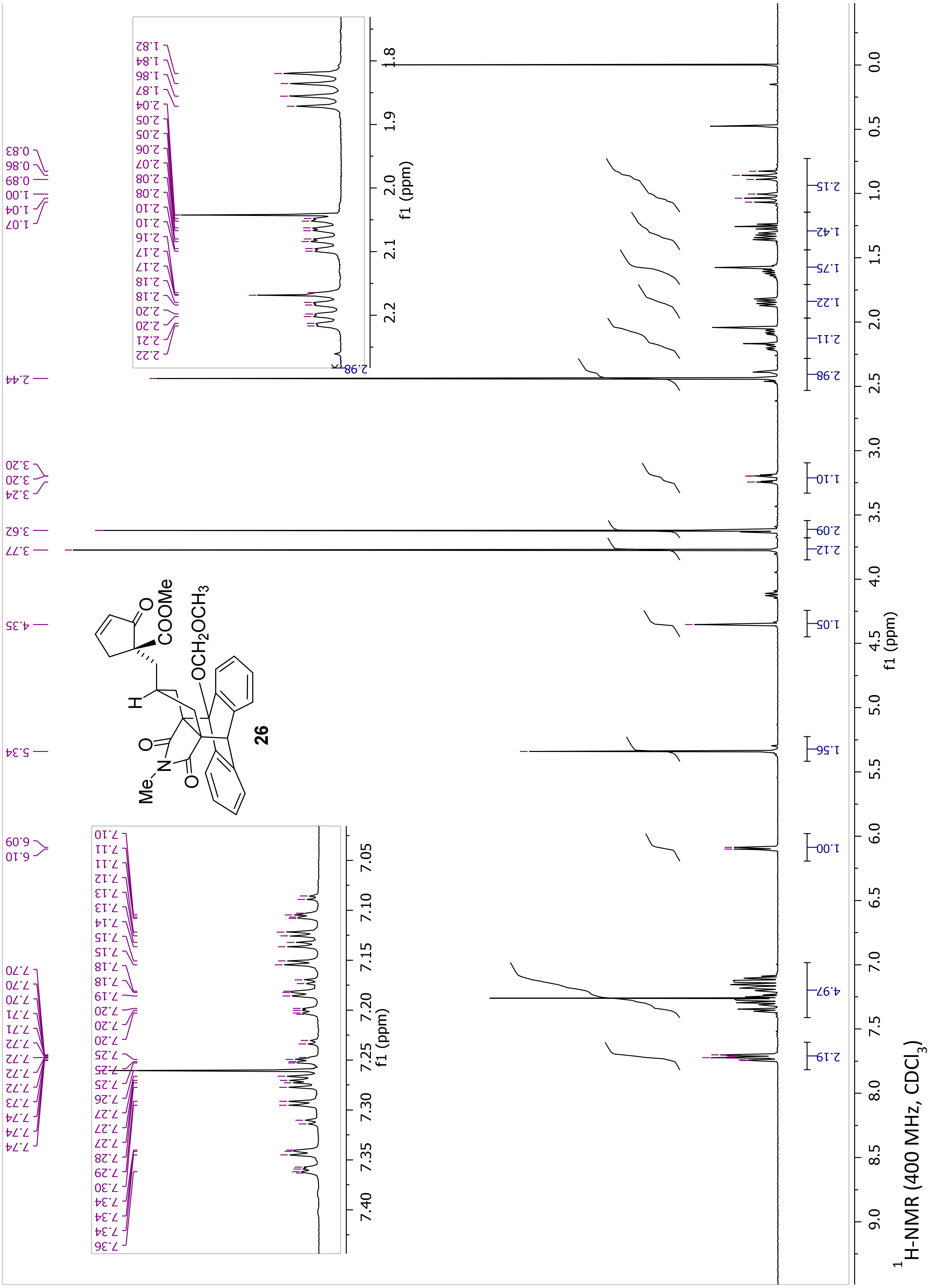

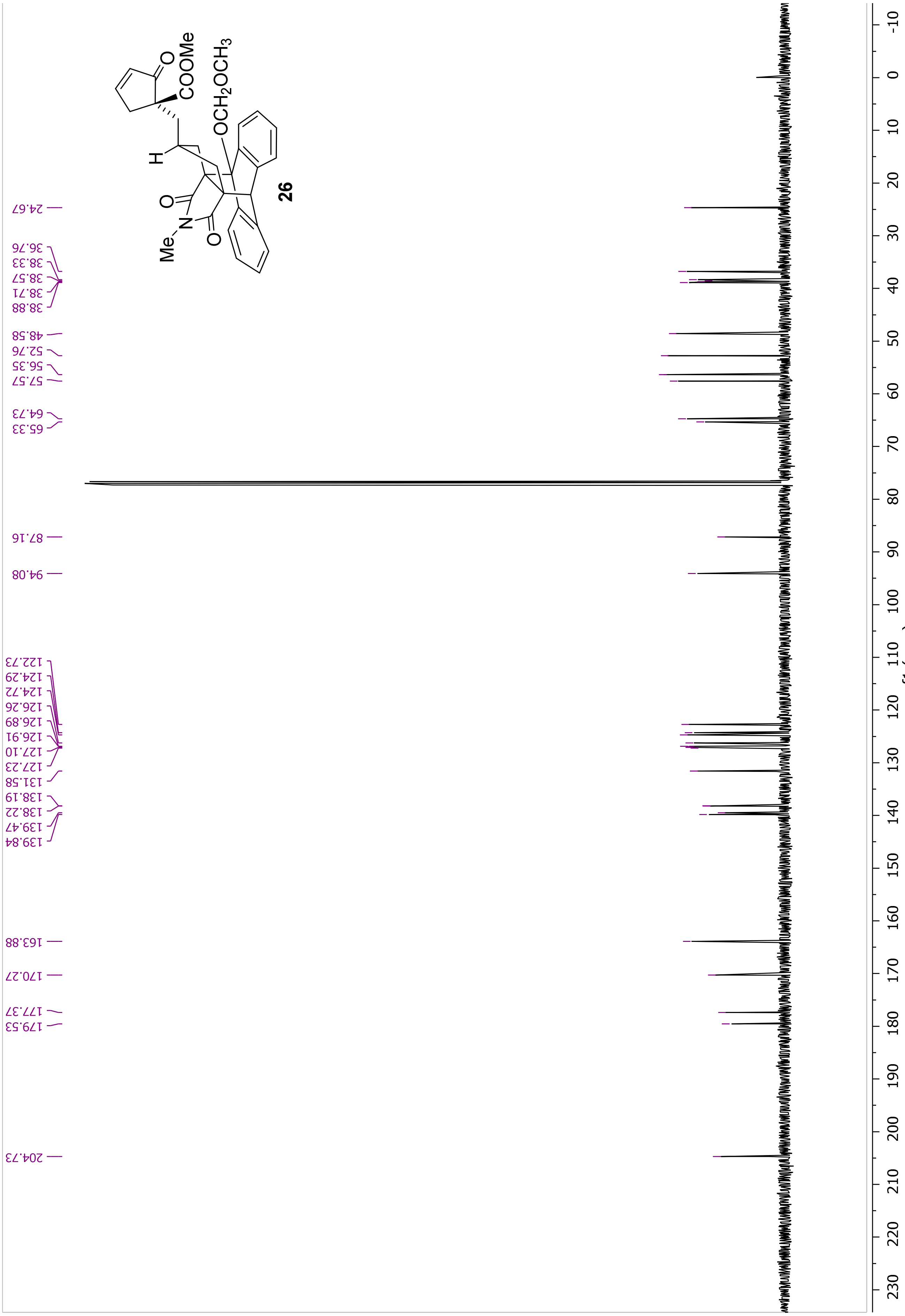

^{13}C-NMR (100.6 MHz, $CDCl_3$)

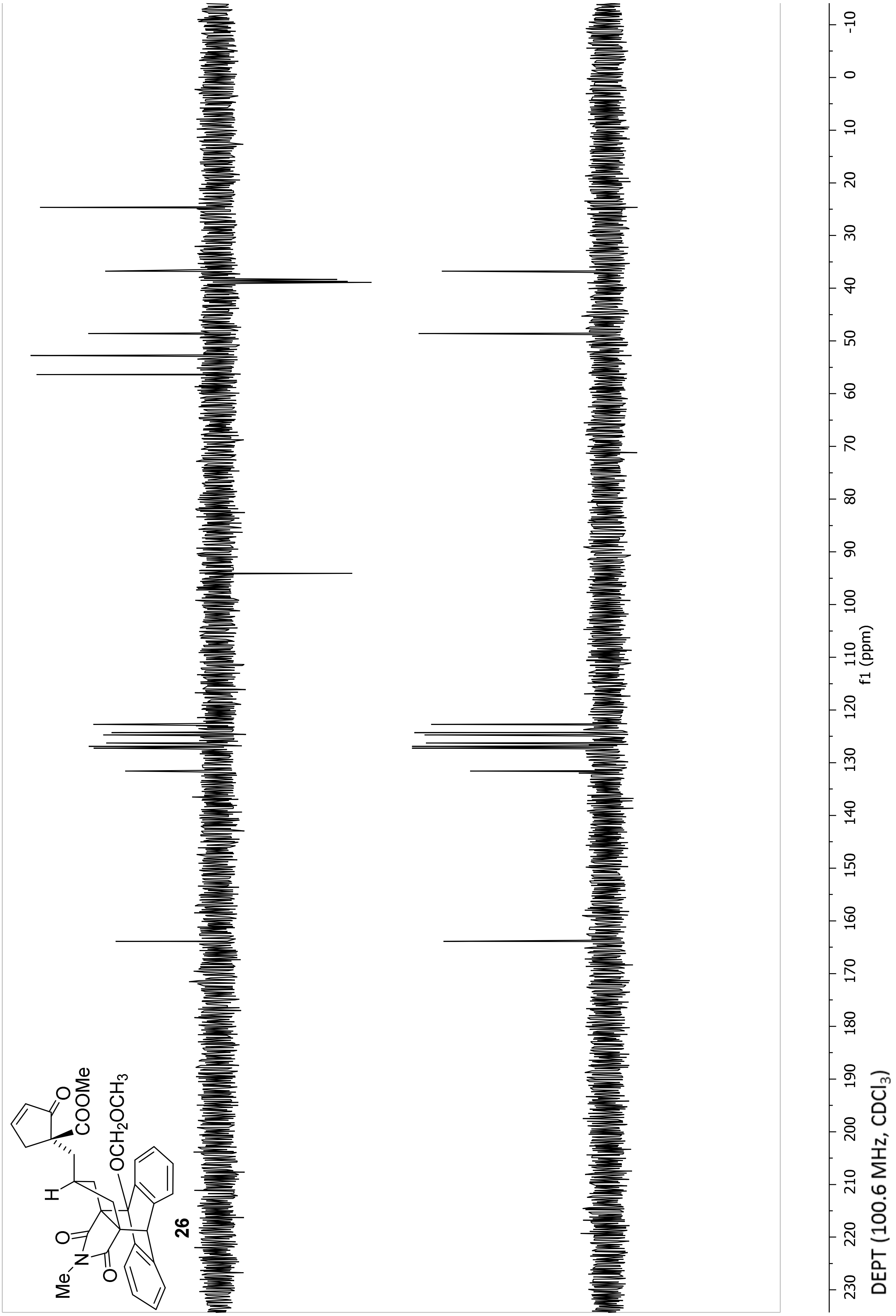
f1 (ppm)
DEPT (100.6 MHz, CDCl3)
-10
0
10
20
30
40
50
60
70
80
90
100
110
120
130
140
150
160
170
180
190
200
210
220
230
COOMe
O
OCH2OCH3
H
O
N
Me
O
26

NMR and IR spectra of compound **26**

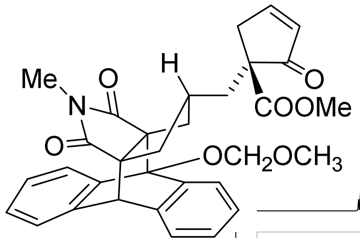

26

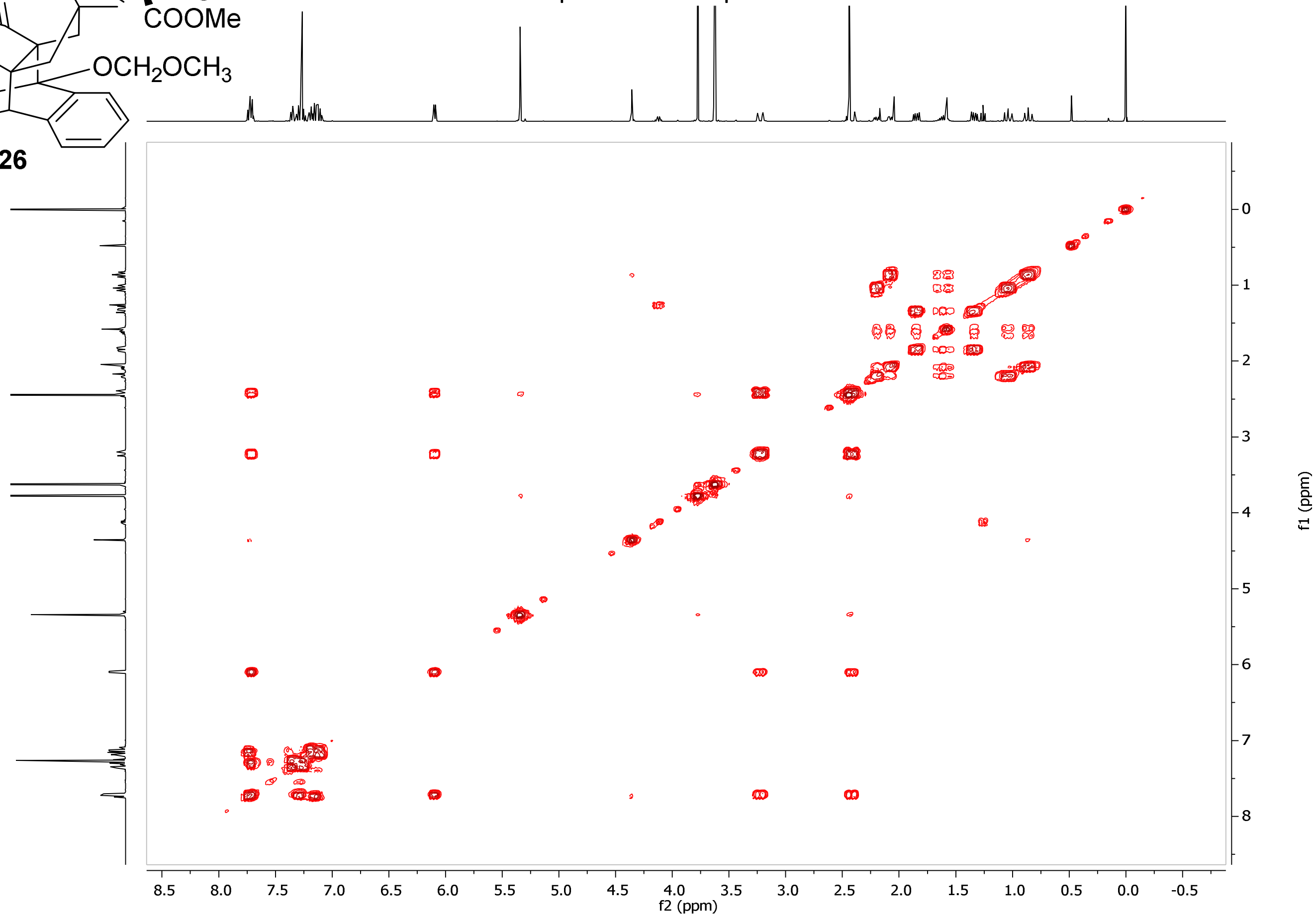

^{1}H/^{1}H COSY (400 MHz, CDCl$_3$)

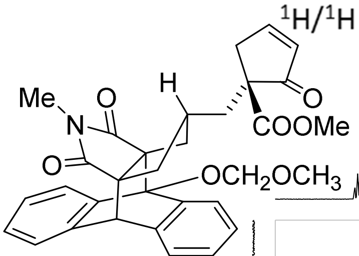

26

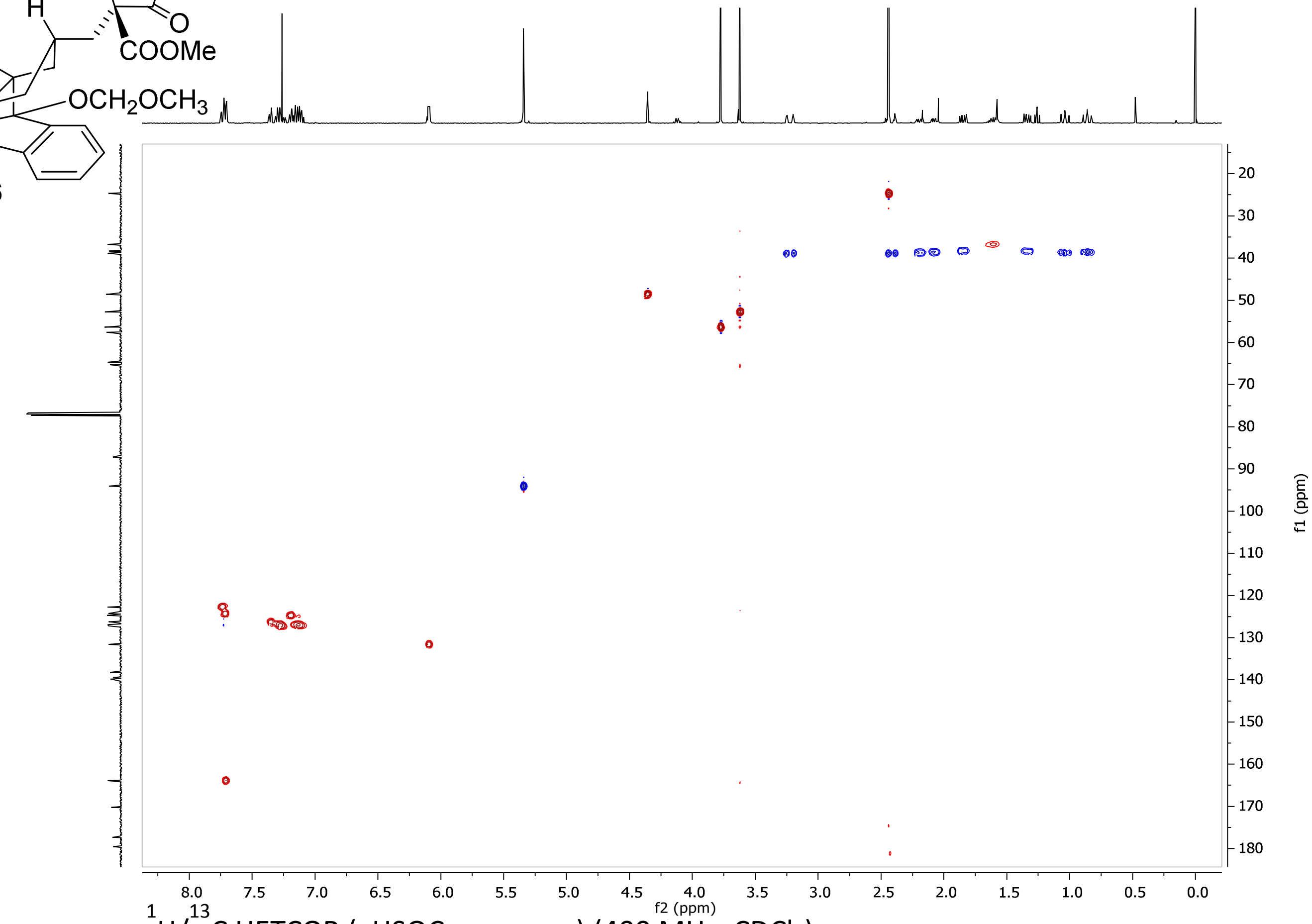

^{1}H/^{13}C HETCOR (gHSQC sequence) (400 MHz, CDCl$_3$)

NMR and IR spectra of compound **26**

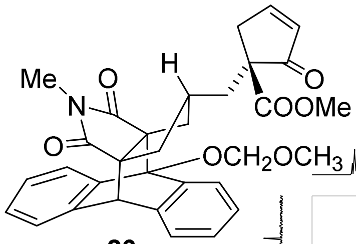

26

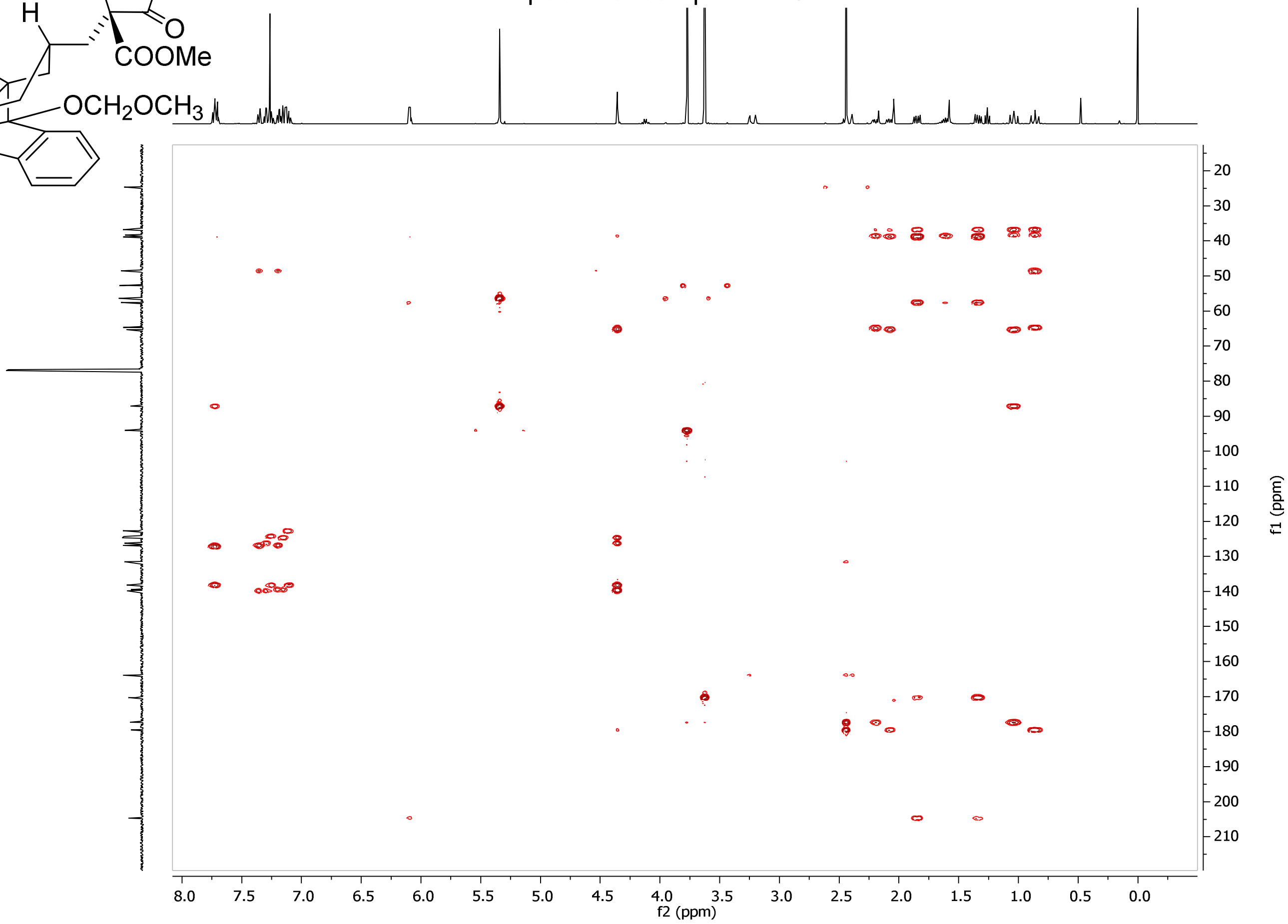

$^{1}H/^{13}C$ HETCOR (gHMBC sequence) (400 MHz, $CDCl_3$)

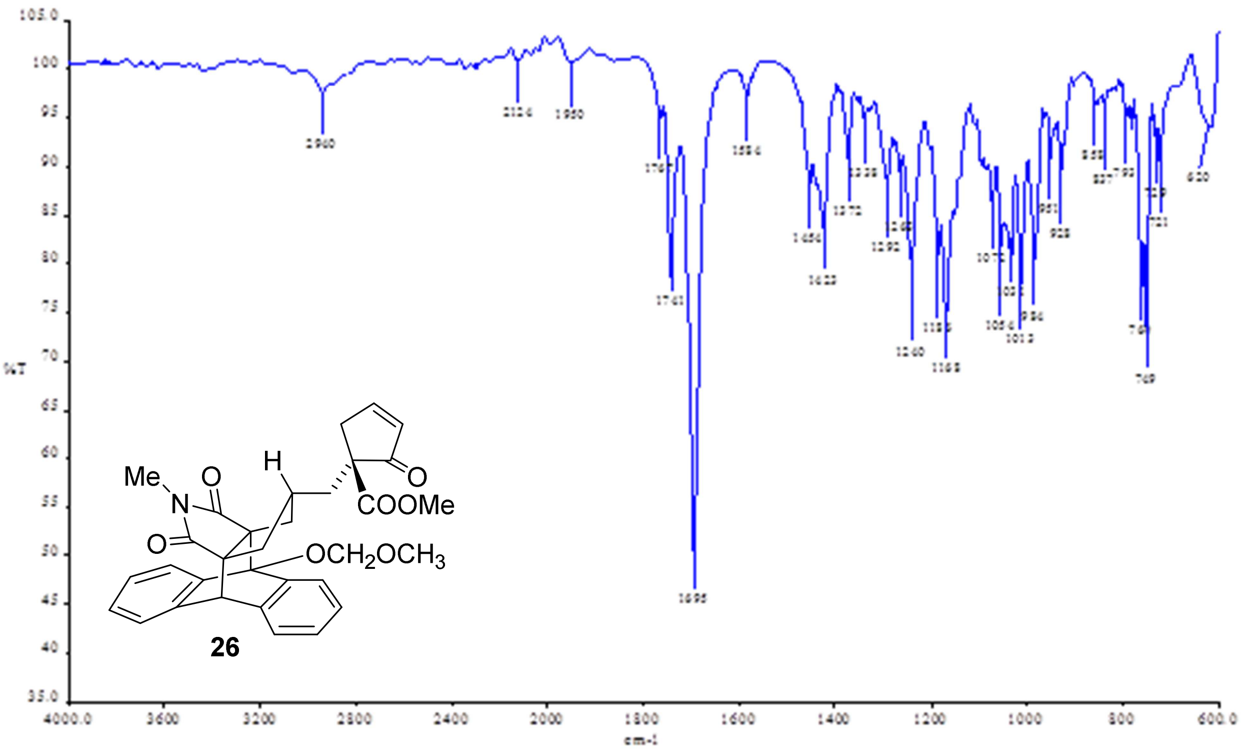

IR (ATR)

NMR and IR spectra of compound **30**

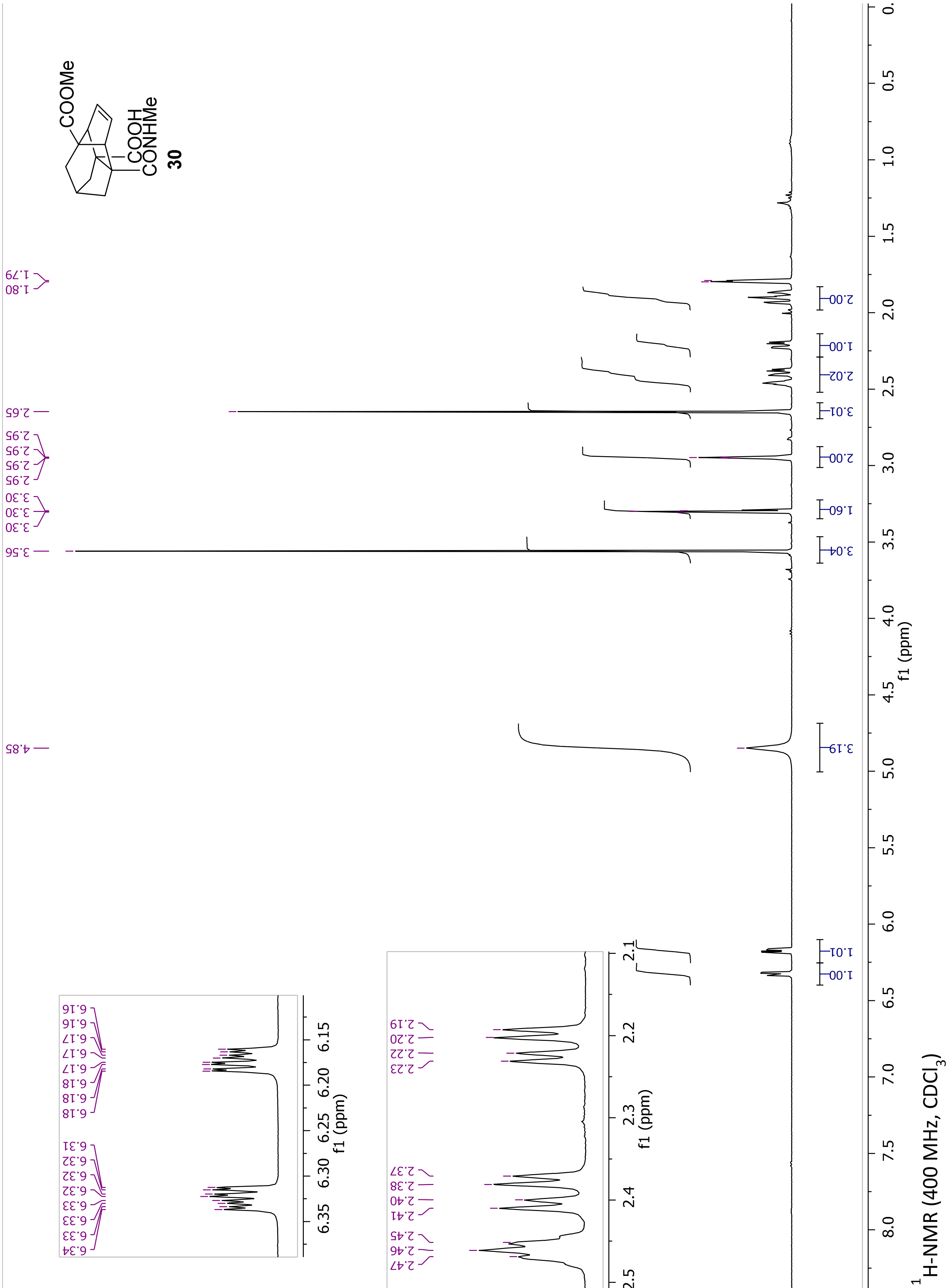

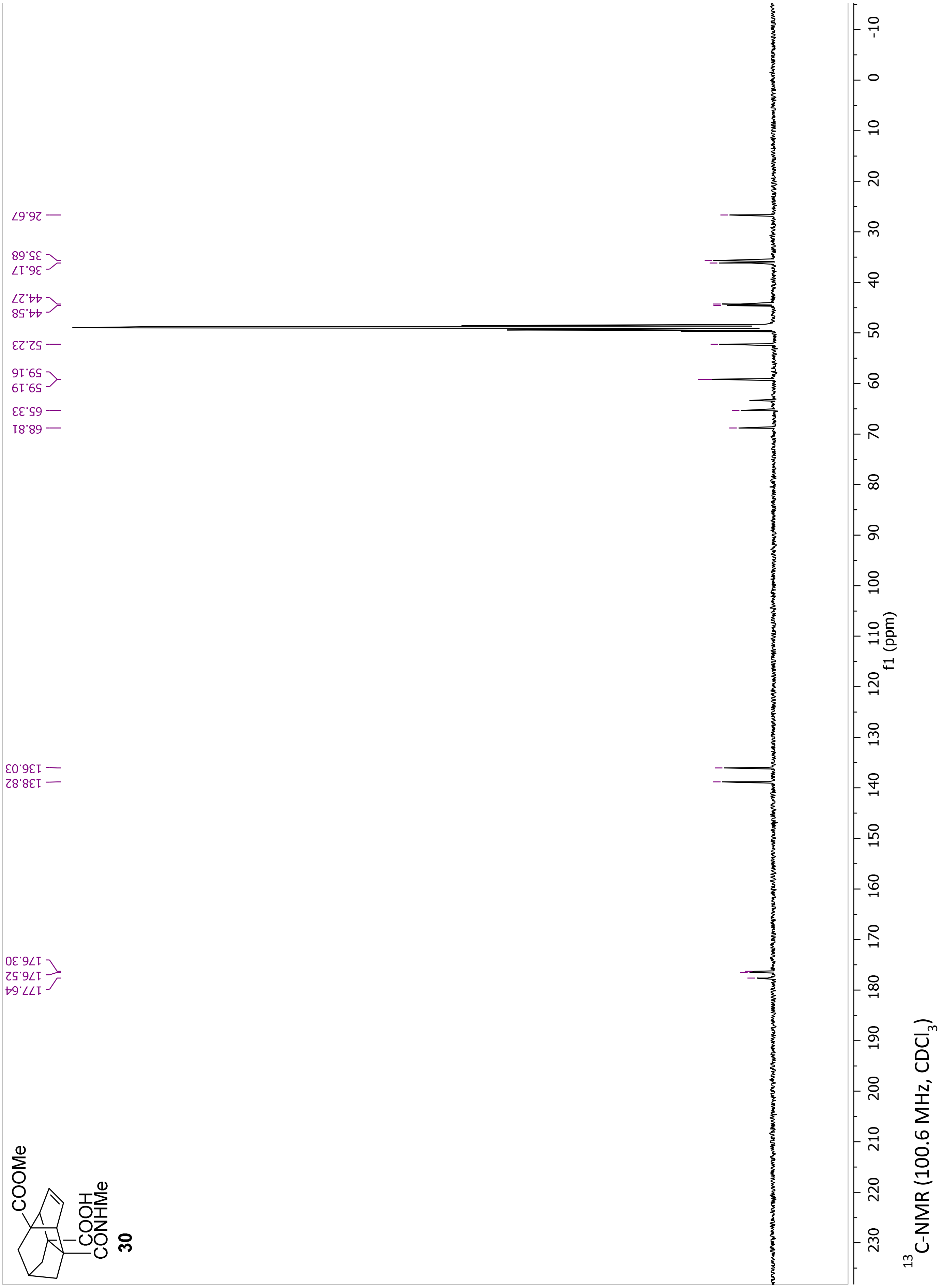
26.67
35.68
36.17
44.27
44.58
52.23
59.16
59.19
65.33
68.81
136.03
138.82
176.30
176.52
177.64
COOMe
COOH
CONHMe
30
f1 (ppm)
13C-NMR (100.6 MHz, CDCl3)

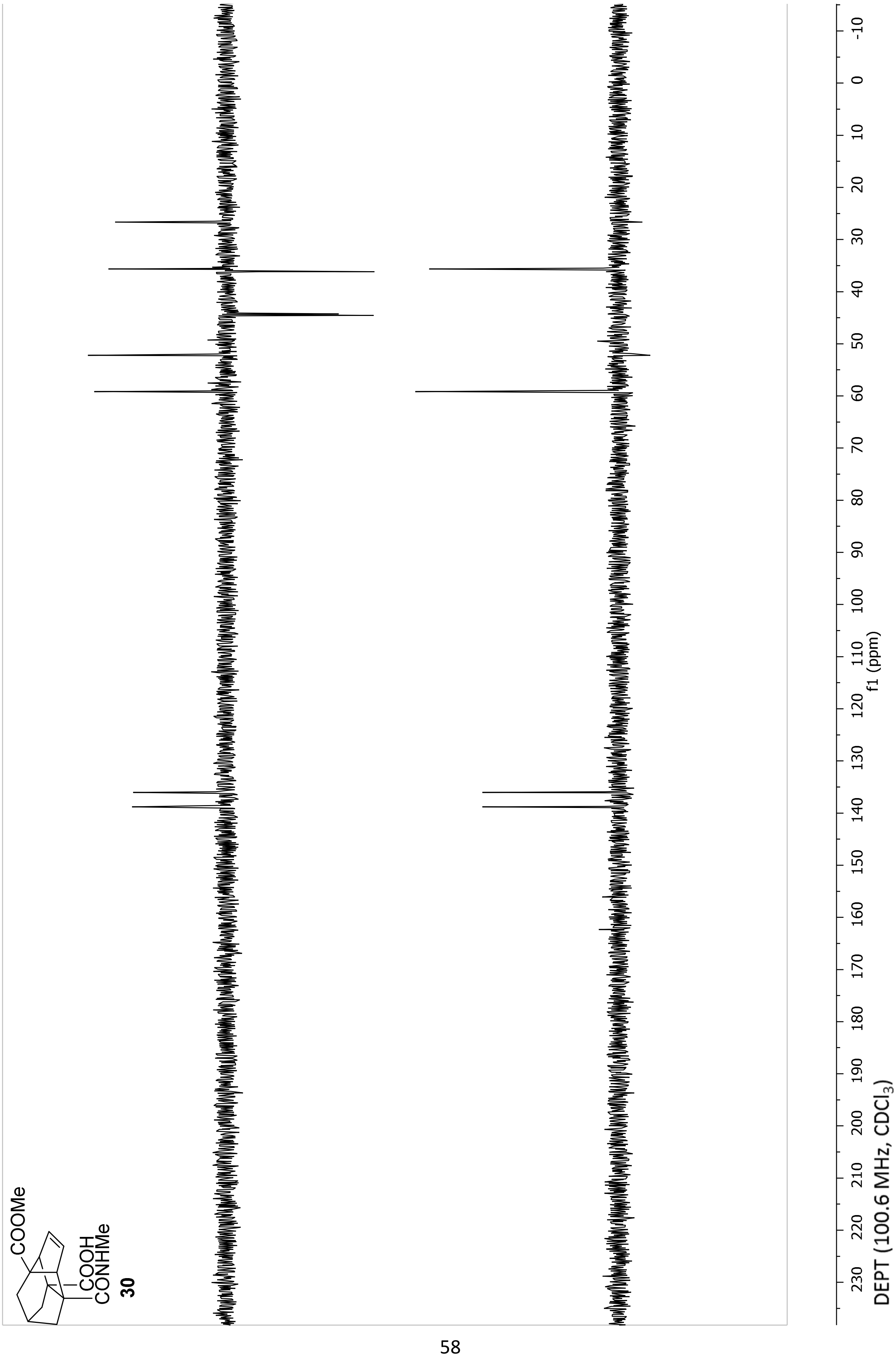
COOMe
COOH
CONHMe
30
f1 (ppm)
DEPT (100.6 MHz, CDCl3)

NMR and IR spectra of compound **30**

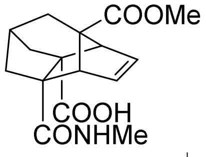

30

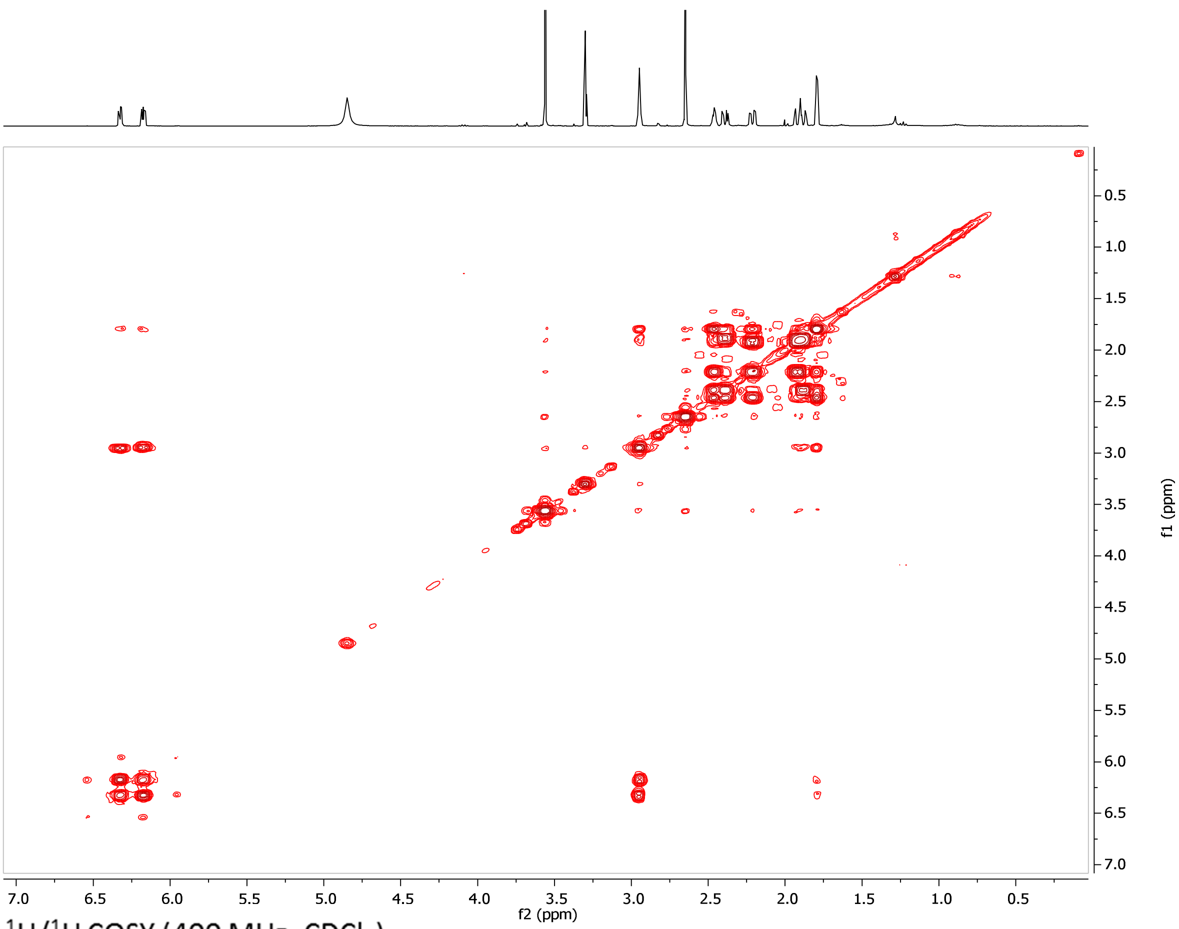

$^1H/^1H$ COSY (400 MHz, $CDCl_3$)

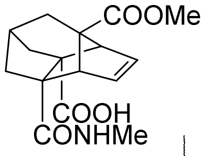

30

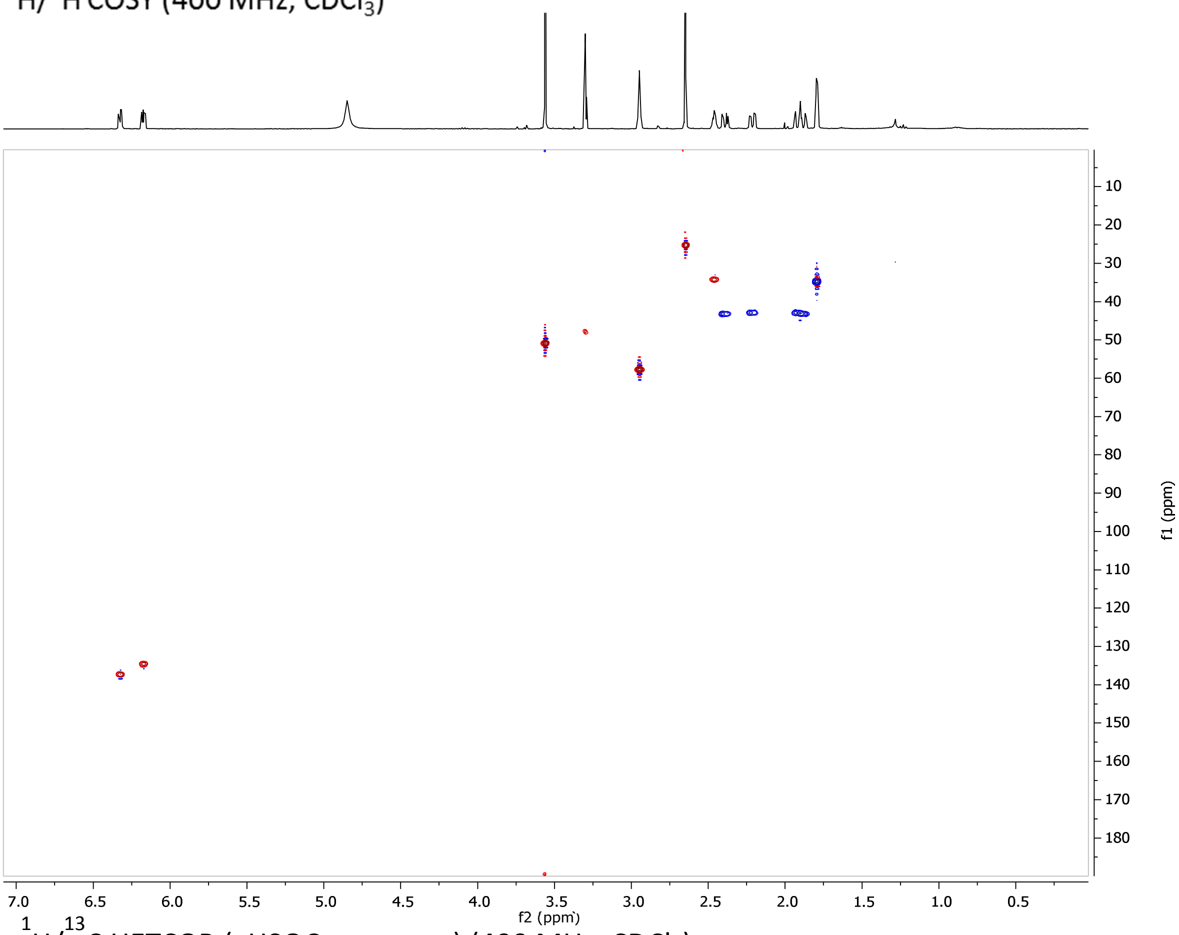

$^1H/^{13}C$ HETCOR (gHSQC sequence) (400 MHz, $CDCl_3$)

NMR and IR spectra of compound **30**

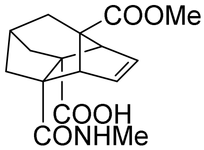

30

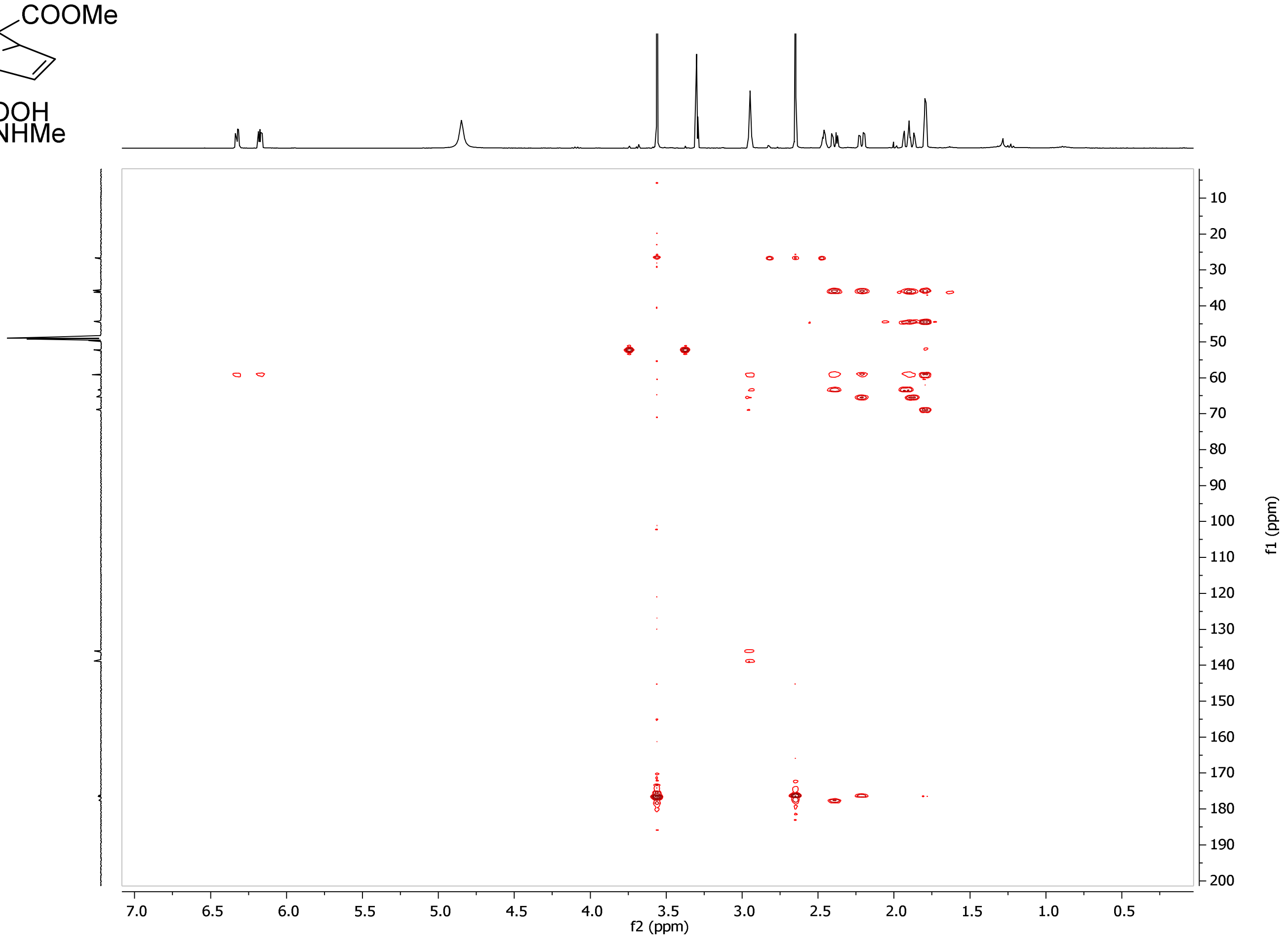

$^{1}H/^{13}C$ HETCOR (gHMBC sequence) (400 MHz, $CDCl_3$)

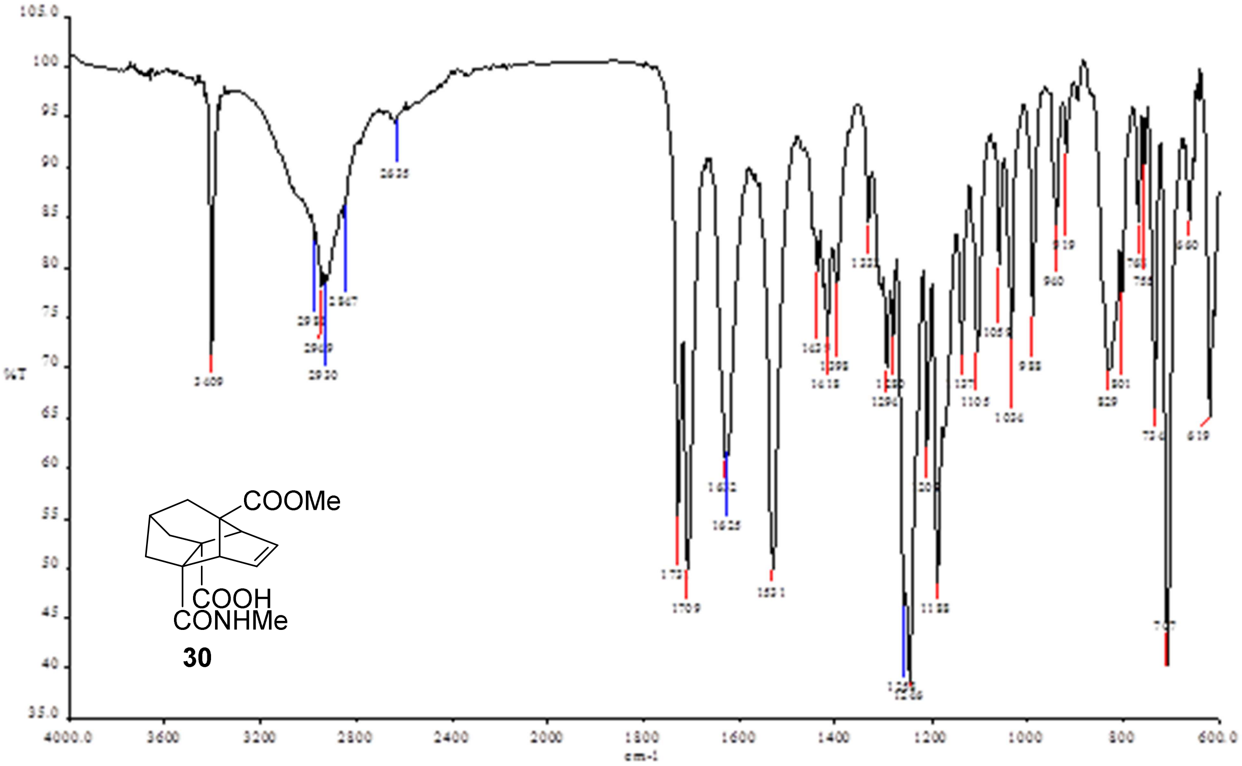

30

IR (ATR)

NMR and IR spectra of compound **31**

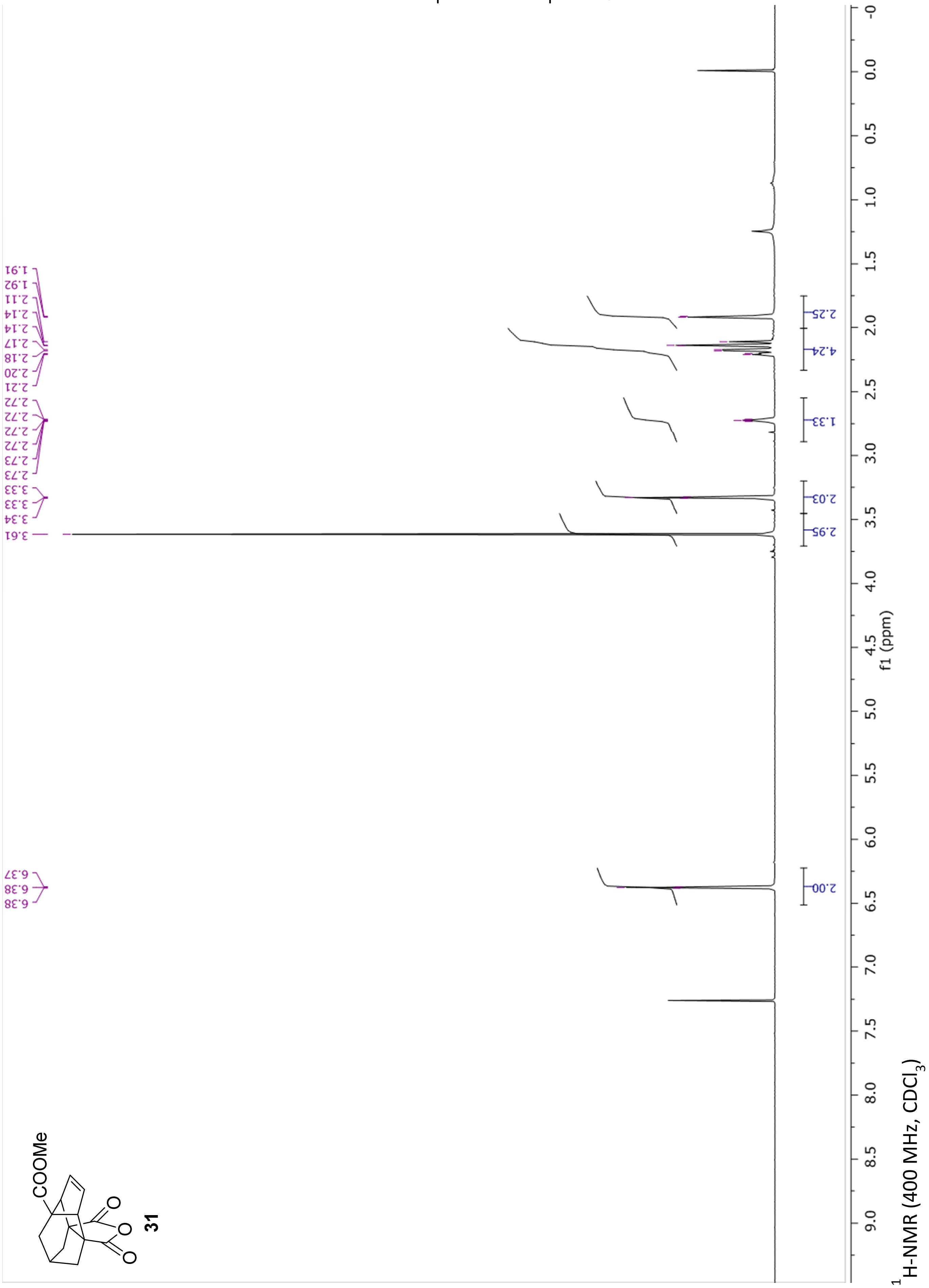

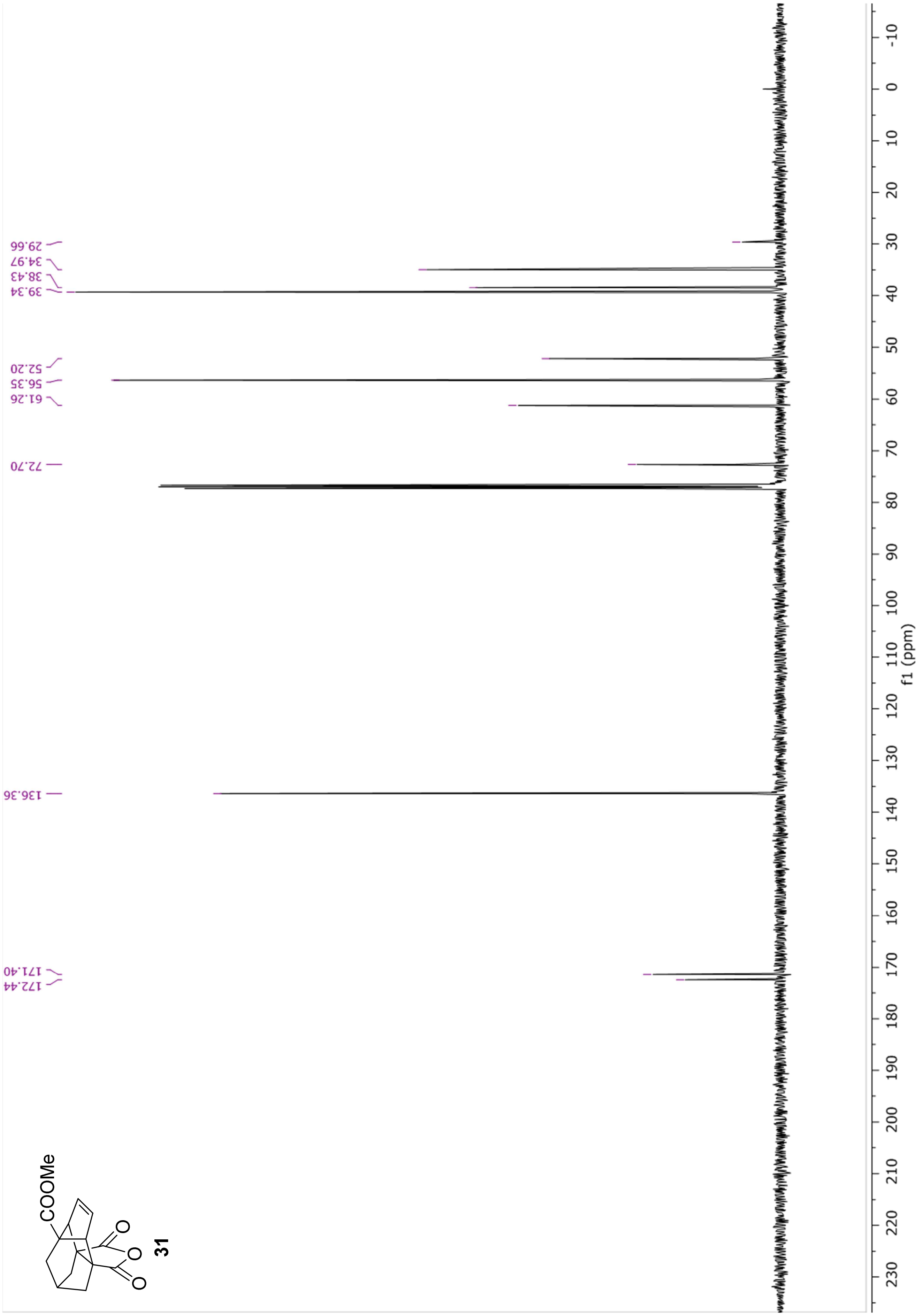

^{13}C-NMR (100.6 MHz, $CDCl_3$)

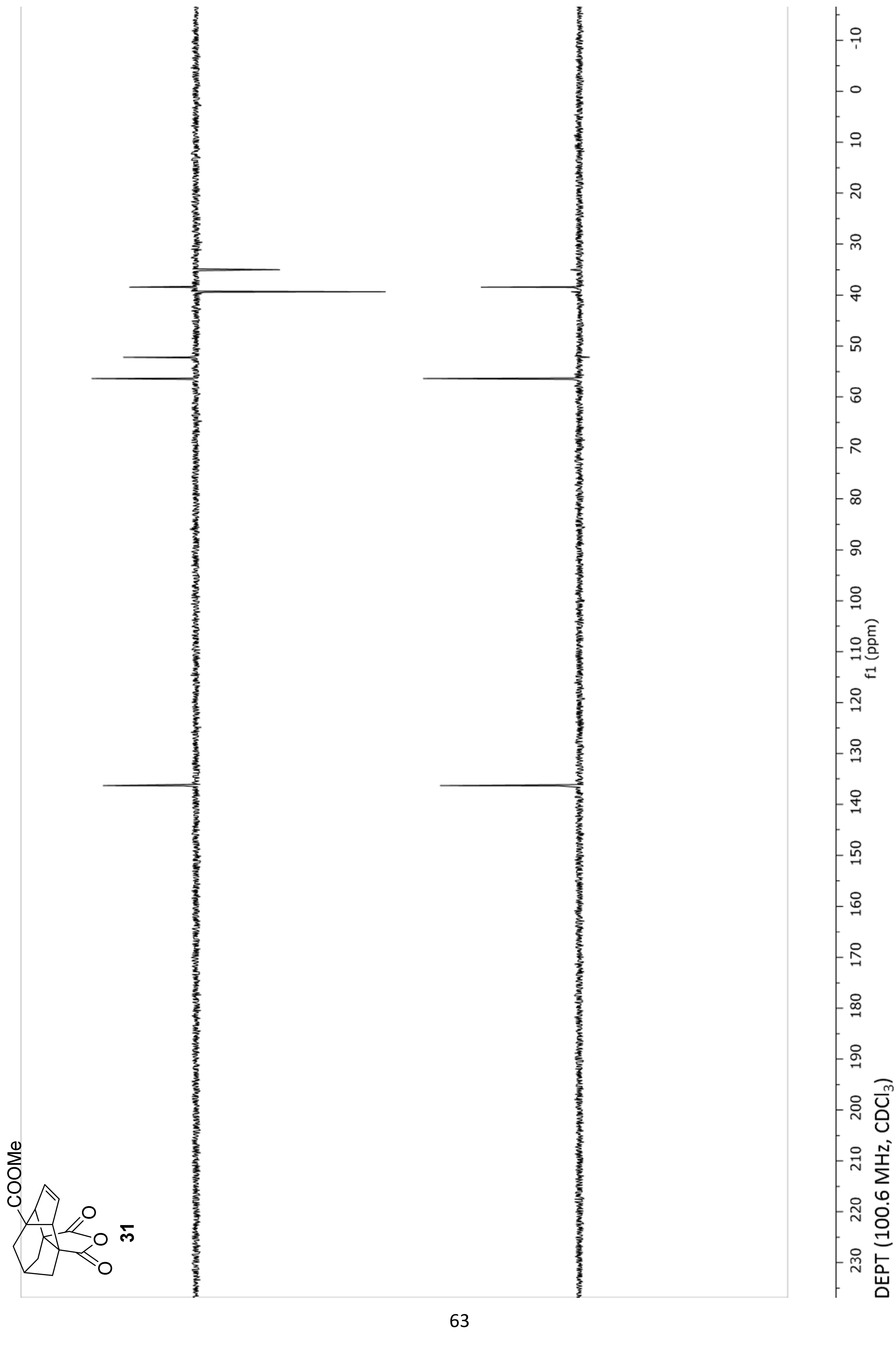
COOMe
O
O
O
31
f1 (ppm)
DEPT (100.6 MHz, CDCl3)

NMR and IR spectra of compound **31**

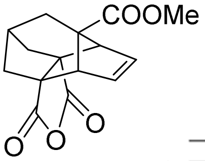

31

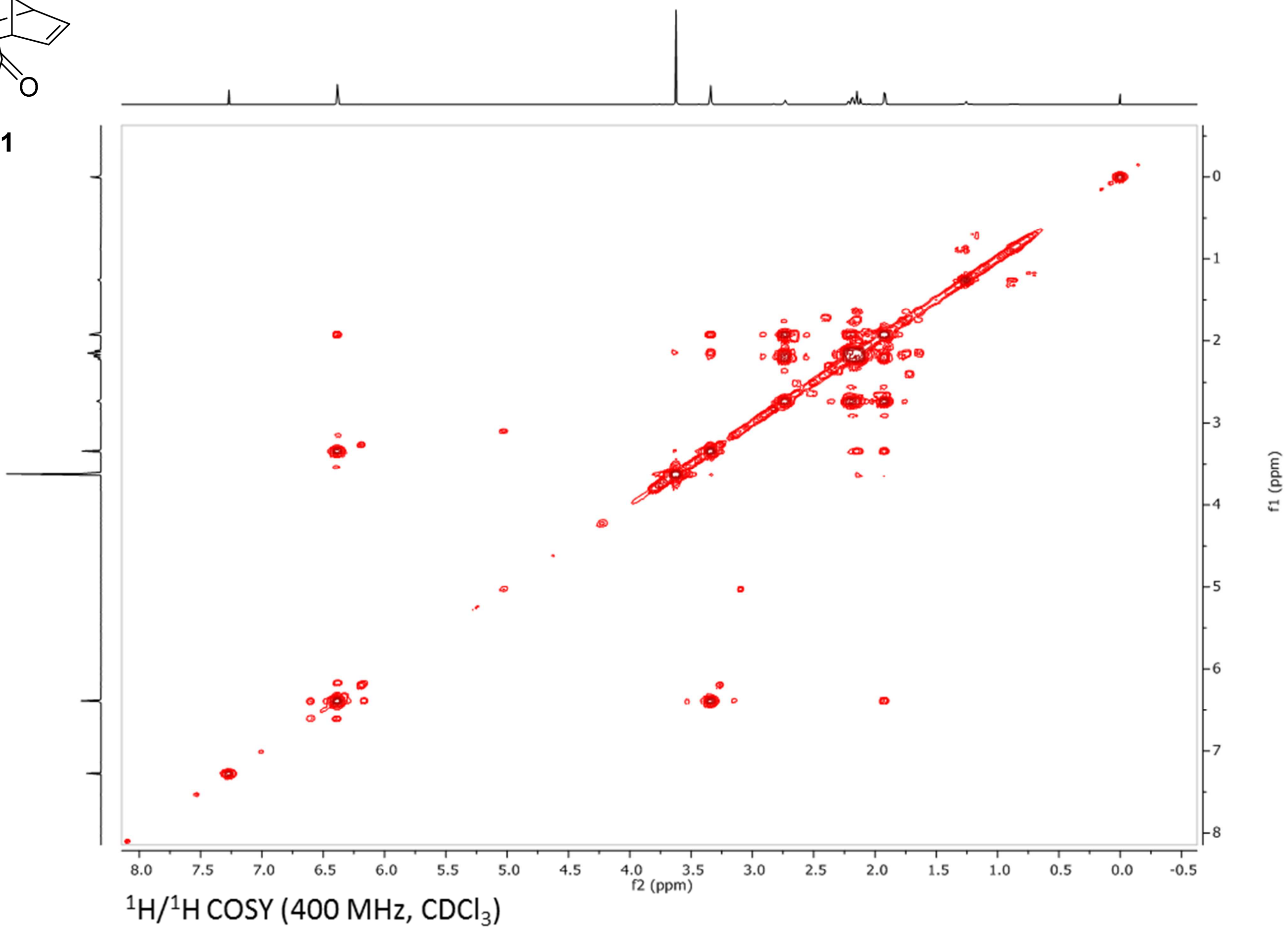

$^1H/^1H$ COSY (400 MHz, $CDCl_3$)

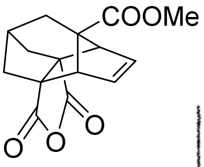

31

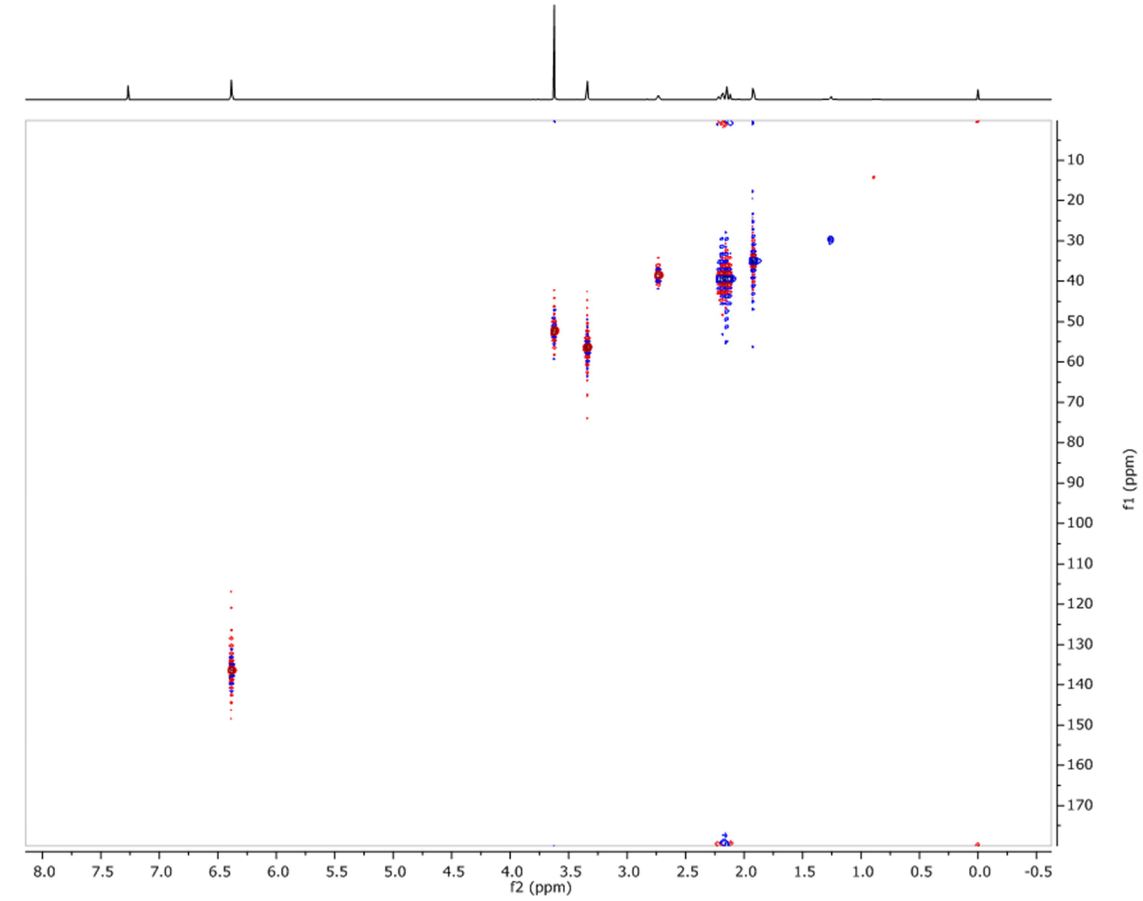

$^1H/^{13}C$ HETCOR (gHSQC sequence) (400 MHz, $CDCl_3$)

NMR and IR spectra of compound **31**

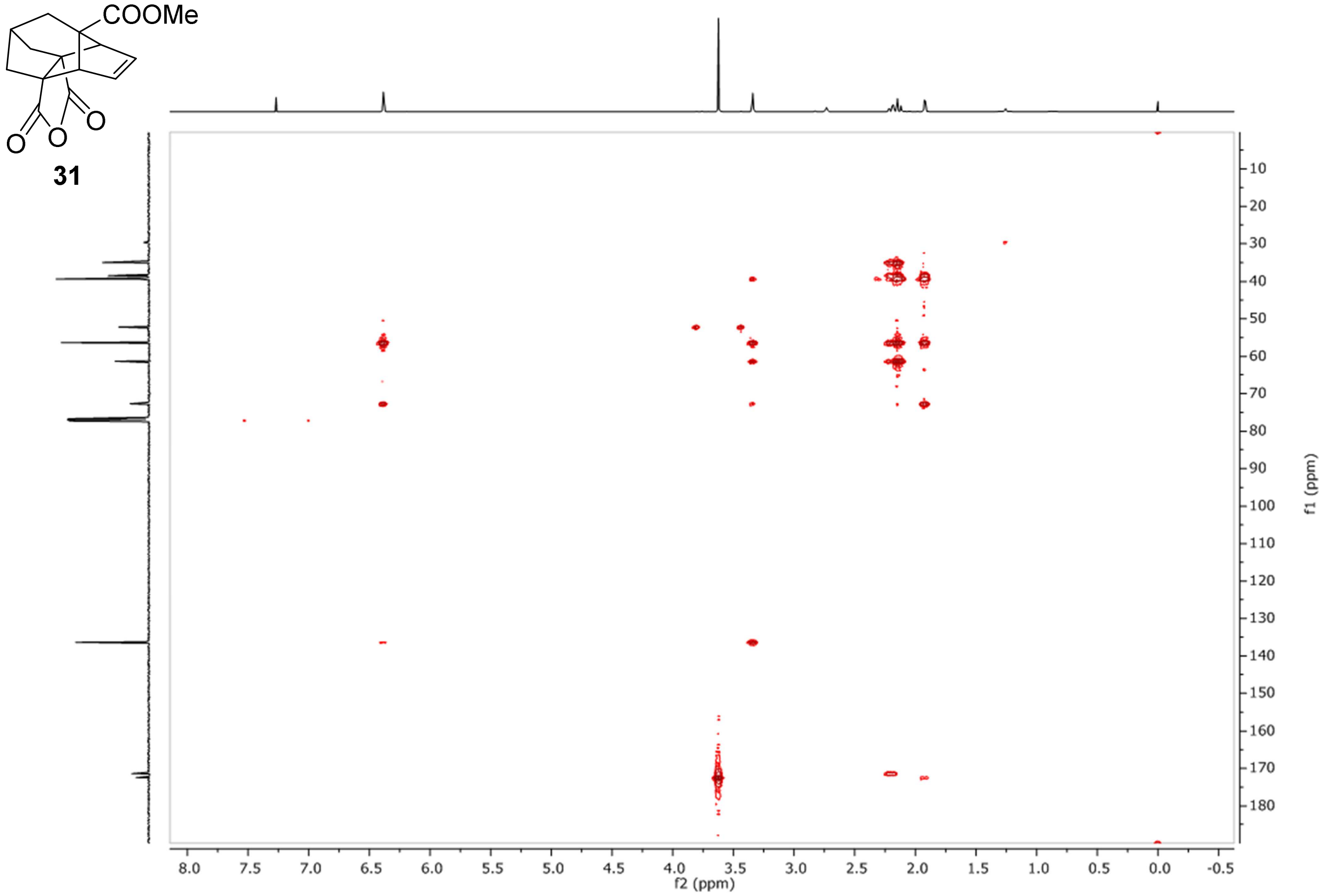

^{1}H/^{13}C HETCOR (gHMBC sequence) (400 MHz, $CDCl_3$)

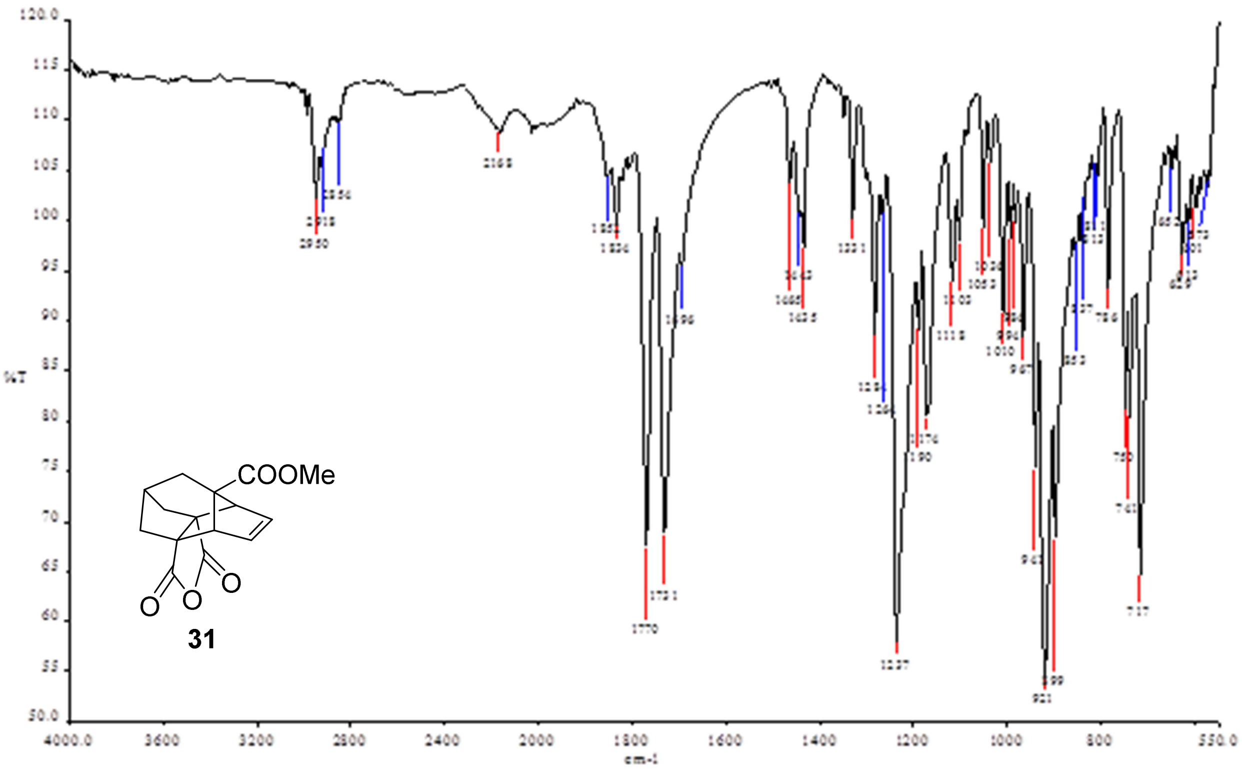

IR (ATR)

NMR and IR spectra of compound **32**

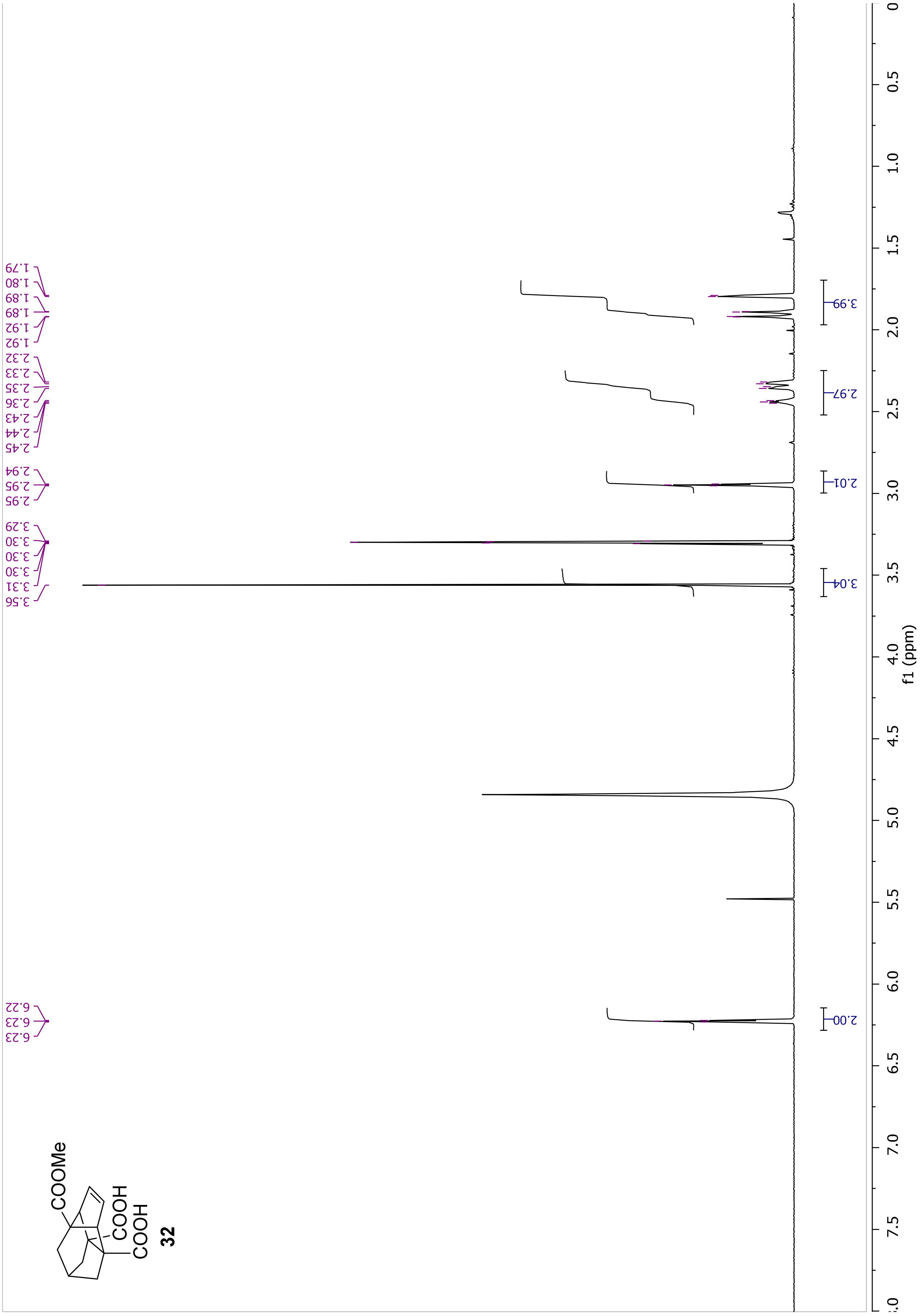

^{1}H-NMR (400 MHz, $CDCl_3$)

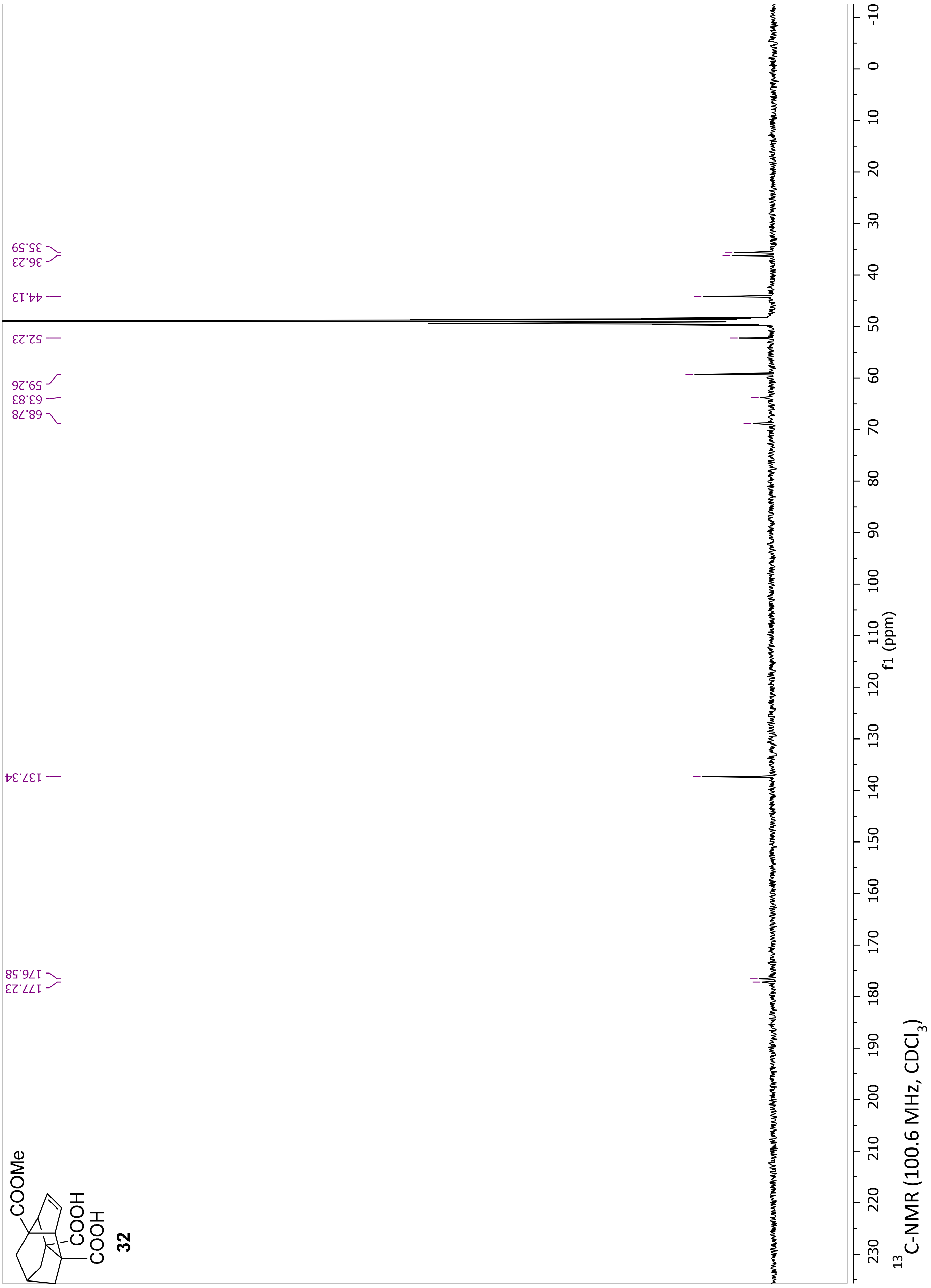
35.59
36.23
44.13
52.23
59.26
63.83
68.78
137.34
176.58
177.23
COOMe
COOH
COOH
32
f1 (ppm)
^{13}C-NMR (100.6 MHz, $CDCl_3$)

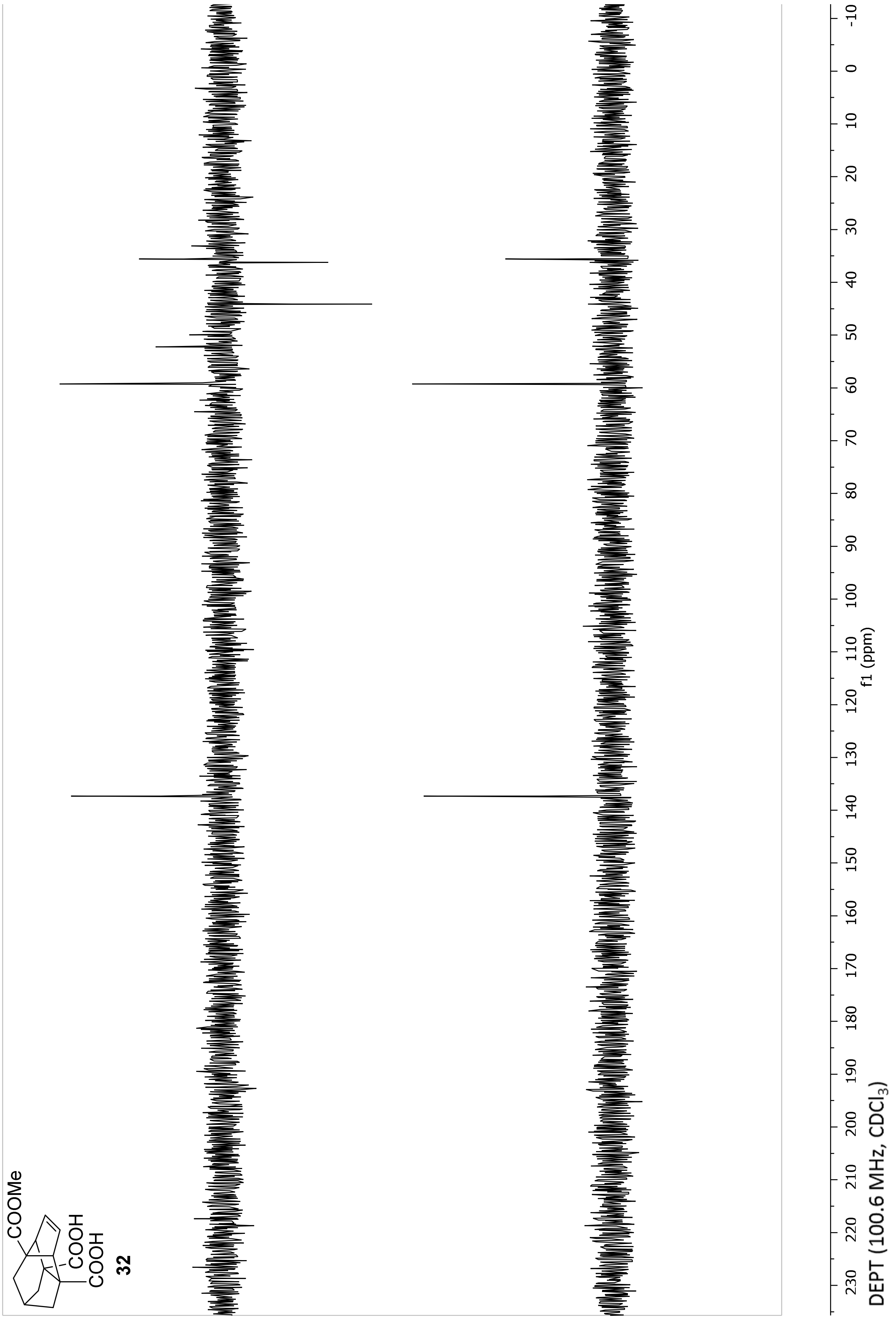
COOMe
COOH
COOH
32
f1 (ppm)
DEPT (100.6 MHz, CDCl3)

NMR and IR spectra of compound **32**

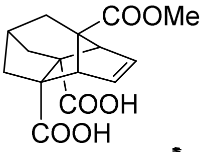

32

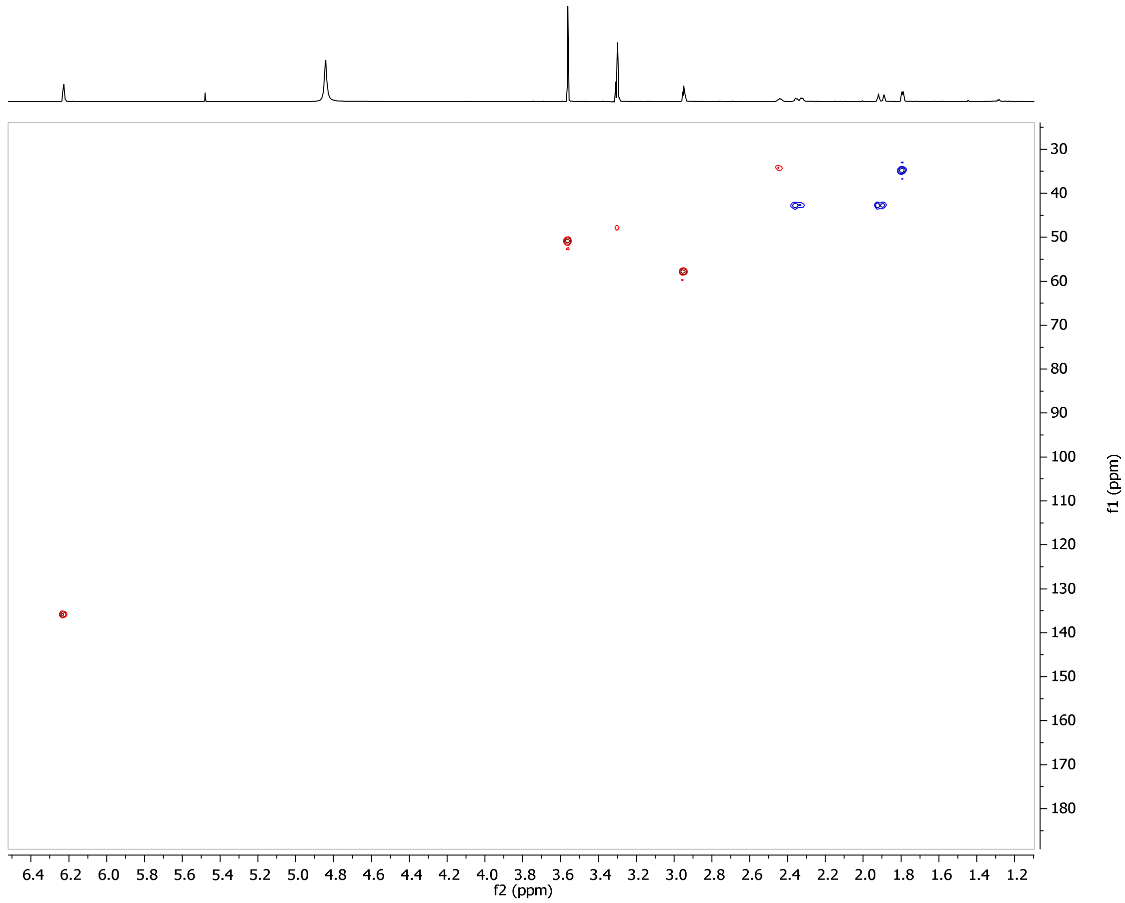

$^1H/^{13}C$ HETCOR (gHSQC sequence) (400 MHz, $CDCl_3$)

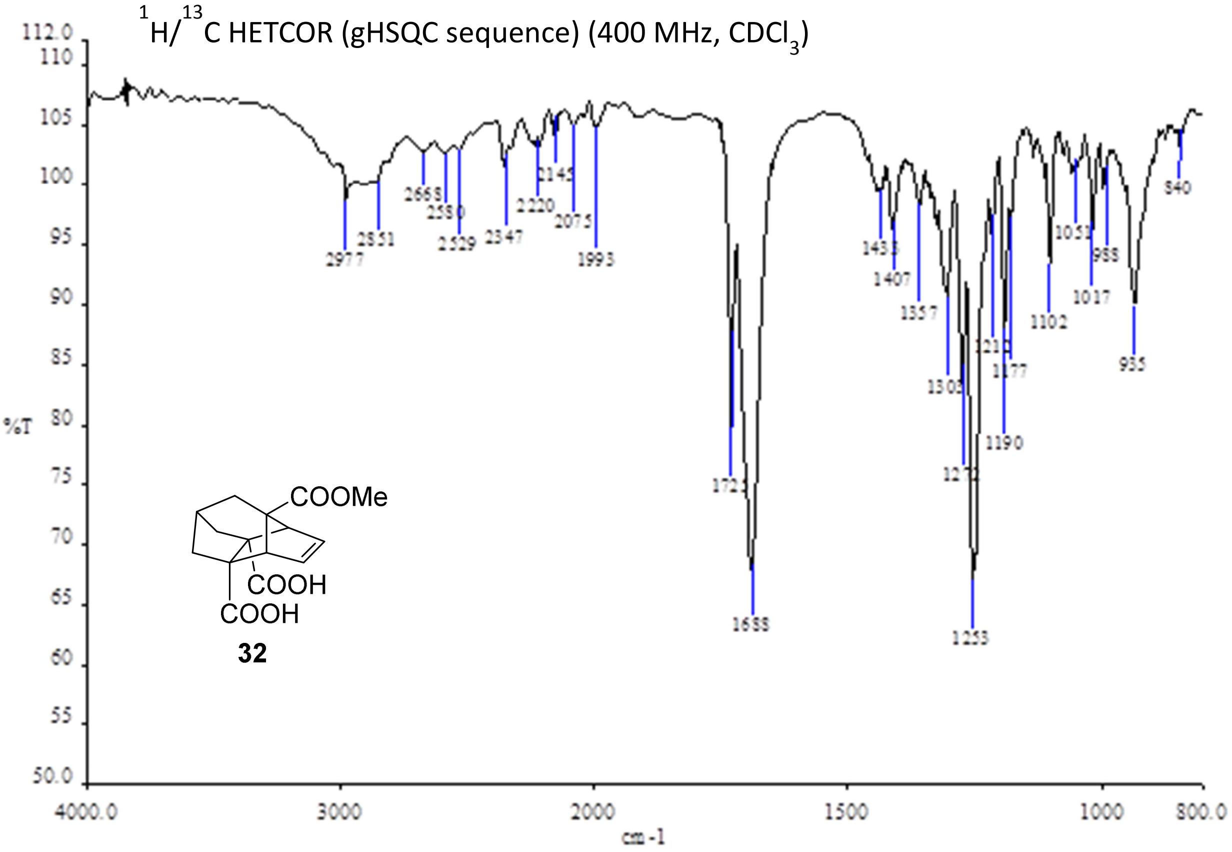

Supplement: Supplementary file 1 [file molecules-22-00906-s001.pdf]
